# Targeting Receptor Tyrosine Kinase VEGFR-2 in Hepatocellular Cancer: Rational Design, Synthesis and Biological Evaluation of 1,2-Disubstituted Benzimidazoles

**DOI:** 10.3390/molecules25040770

**Published:** 2020-02-11

**Authors:** Heba T. Abdel-Mohsen, Mona A. Abdullaziz, Ahmed M. El Kerdawy, Fatma A. F. Ragab, Keith J. Flanagan, Abeer E. E. Mahmoud, Mamdouh M. Ali, Hoda I. El Diwani, Mathias O. Senge

**Affiliations:** 1 Department of Chemistry of Natural and Microbial Products, Division of Pharmaceutical and Drug Industries Research, National Research Centre, Dokki, P.O. 12622, Cairo, Egypt; m.abdulla87@hotmail.com (M.A.A.); heldiwani@gmail.com (H.I.E.D.); 2 Department of Pharmaceutical Chemistry, Faculty of Pharmacy, Cairo University, Kasr El-Aini Street, P.O. Box 11562, Cairo, Egypt; Fatmarag@hotmail.com; 3 Department of Pharmaceutical Chemistry, Faculty of Pharmacy, New Giza University, New Giza, km 22 Cairo–Alexandria Desert Road, Cairo, Egypt; 4 Medicinal Chemistry, Trinity Translational Medicine Institute, Trinity Centre for Health Sciences, Trinity College Dublin, The University of Dublin, St. James’s Hospital, Dublin 8, Ireland; kflanaga@tcd.ie; 5 Department of Biochemistry, Division of Genetic Engineering and Biotechnology, National Research Centre, Cairo, Egypt; ab_essam@yahoo.com (A.E.E.M.); mmali1999@yahoo.com (M.M.A.)

**Keywords:** design, synthesis, 1,2-disubstituted benzimidazole, VEGFR-2, angiogenesis, HepG-2

## Abstract

In this study, a novel series of 1,2-disubstituted benzo[*d*]imidazoles was rationally designed as VEGFR-2 inhibitors targeting hepatocellular carcinoma. Our design strategy is two-fold; it aimed first at studying the effect of replacing the 5-methylfuryl moiety of the well-known antiangiogenic 2-furylbenzimidazoles with an isopropyl moiety on the VEGFR-2 inhibitory activity and the cytotoxic activity. Our second objective was to further optimize the structures of the benzimidazole derivatives through elongation of the side chains at their one-position for the design of more potent type II-like VEGFR-2 inhibitors. The designed 1,2-disubstituted benzimidazoles demonstrated potent cytotoxic activity against the HepG2 cell line, reaching IC_50_ = 1.98 μM in comparison to sorafenib (IC_50_ = 10.99 μM). In addition, the synthesized compounds revealed promising VEGFR-2 inhibitory activity in the HepG2 cell line, e.g., compounds **17a** and **6** showed 82% and 80% inhibition, respectively, in comparison to sorafenib (% inhibition = 92%). Studying the effect of **17a** on the HepG2 cell cycle demonstrated that **17a** arrested the cell cycle at the G2/M phase and induced a dose-dependent apoptotic effect. Molecular docking studies of the synthesized 1,2-disubstituted benzimidazoles in the VEGFR-2 active site displayed their ability to accomplish the essential hydrogen bonding and hydrophobic interactions for optimum inhibitory activity.

## 1. Introduction

Receptor tyrosine kinases (RTKs), a family of receptors that exist on the cell surface, play a crucial role in the cellular response to environmental signals [1]. They also mediate cellular proliferation and survival. In normal cells, RTK expression is highly regulated; however, in some pathological conditions such as cancer there is an extreme up-regulation of some RTKs [2,3]. 

In this respect, the vascular endothelial growth factor receptor (VEGFR) family, composed of VEGFR-1, VEGFR-2 and VEGFR-3 isoforms, is one of the main RTK families that play a significant role in angiogenesis and lymphogenesis [4,5,6,7]. In particular, VEGFR-2 is the main key mediator of mitogenesis and angiogenesis in endothelial cells [8]. At certain stages of cancer, signaling of VEGFR-2 is up-regulated to support tumor proliferation and migration [7]. Hence, inhibition of this signaling pathway is considered an efficient approach for hindering pathological angiogenesis and, in turn, counteracts the process of cancer growth, proliferation and metastasis [8]. 

**Figure 1:**
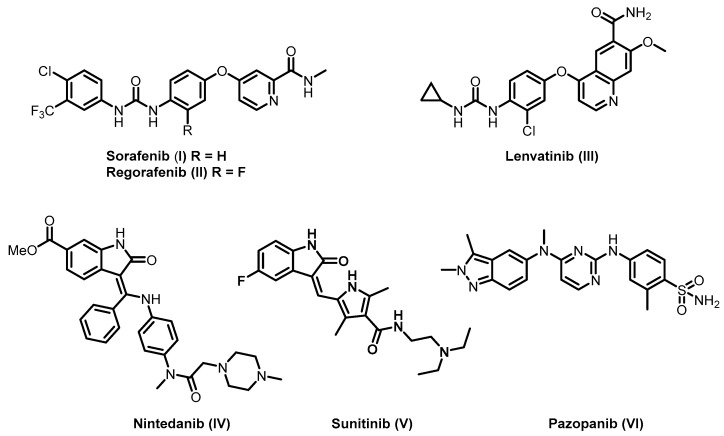
Examples of FDA-approved vascular endothelial growth factor receptor (VEGFR)-2 inhibitors.

Over the last decade, different small molecule VEGFR-2 inhibitors were developed as an adjuvant therapy for cancer chemotherapy [9,10]. For example, sorafenib (**I**), regorafenib (**II**), lenvatinib (**III**), nintedanib (**IV**), sunitinib (**V**) and pazopanib (**VI**) were clinically approved by the FDA for the treatment of different types of cancers (Figure 1) [10,11,12,13,14]. In addition, different research groups designed and synthesized several promising VEGFR-2 inhibitors for targeted cancer therapy [15,16,17,18,19,20]. 

Based on the different reported VEGFR-2 crystal structures, VEGFR-2 inhibitors can be classified into three main types. Type I inhibitors are able to block the active “DFG-in” conformation of the receptor by occupying the ATP binding region forming a hydrogen bond with the hinge region amino acid Cys919. Type II inhibitors occupy the ATP binding site and extend over the gate area into the adjacent allosteric hydrophobic back pocket of the inactive “DFG-out” conformation. Type III inhibitors accommodate the allosteric hydrophobic back pocket of VEGFR-2 in the inactive “DFG-out” conformation, blocking the receptor through hydrophobic interactions [10,17,21].

The extension into the less conservative allosteric hydrophobic back pocket promotes the affinity and selectivity of the type II inhibitors compared to type I inhibitors. Moreover, it prolongs TK suppression, as it increases their drug-target residence time [19,21,22,23,24,25]. Therefore, different strategies have been implemented to develop novel type II VEGFR-2 inhibitors. 

**Figure 2:**
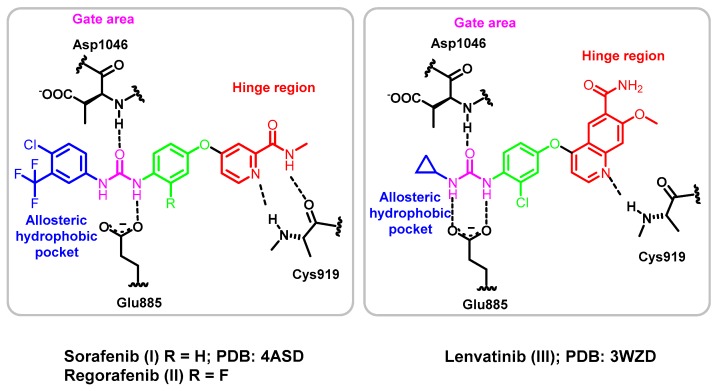
Representation of type II VEGFR-2 inhibitors **I**–**III** in the VEGFR-2 active site.

**Figure 3:**
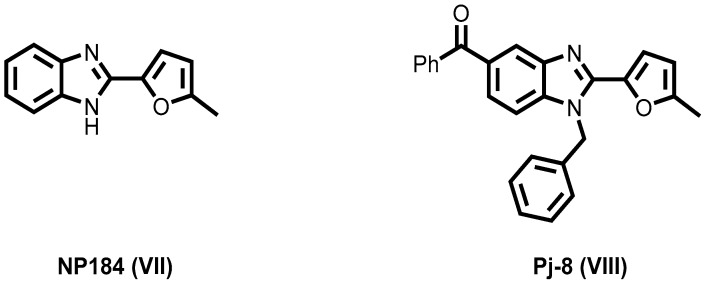
2-Furyl benzimidazoles **VII** and **VIII** of potent antiangiogenic activity.

**Figure 4:**
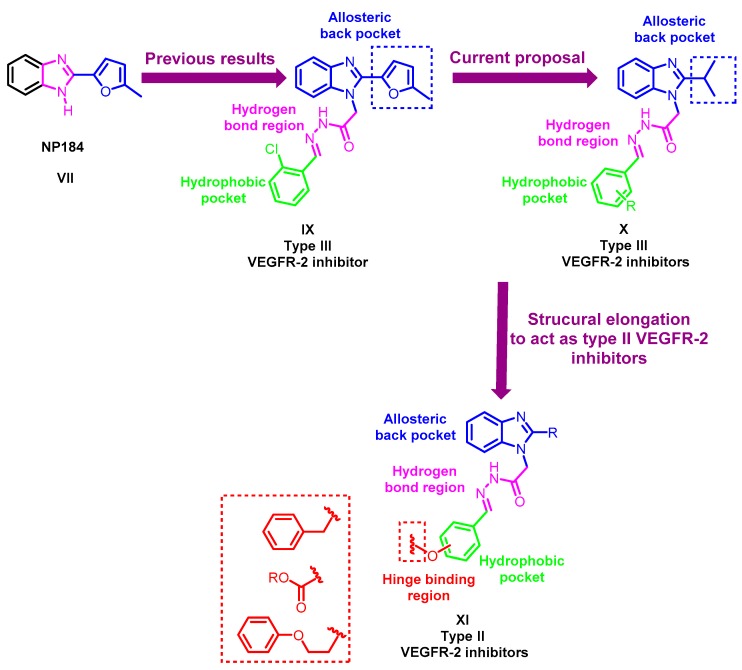
Schematic representation for the design strategy of the novel 1,2-disubstituted-benzimidazole derivatives **X** and **XI**.

The structure of type II inhibitors, e.g., sorafenib (**I**; PDB 4ASD) [26], regorafenib (**II**) and lenvatinib (**III**; 3WZD) [12], were found to share common pharmacophoric features, which are (1) a hinge region binding moiety “head”, which is a heterocycle that occupies the adenine region in the ATP binding pocket with H bond donor and/or acceptor capabilities to interact with Cys919 (colored red in Figure 2); (2) a “linker”, which is a segment of three to four chemical bonds that extends over the gatekeeper residue (colored green in Figure 2); (3) a hydrogen-bonding moiety that is required to achieve hydrogen bond interaction with the Asp1046 in the conserved DFG motif and Glu885 of the αC helix (colored purple in Figure 2) and (4) a “tail” segment typically consisting of a hydrophobic moiety that occupies the allosteric hydrophobic back pocket created by the DFG-out flip (colored blue in Figure 2) [10,27,28].

Hepatocellular adenocarcinoma (HCC) is regarded as one of the most life-threatening cancers around the world [29,30,31]. Recently, it was reported that overexpression of VEGFR-2 in HCC promotes pathological angiogenesis [32,33]. Hence, the application of VEGFR-2 inhibitors in HCC is considered one of the most successful approaches to hinder the growth and spread of hepatic cancer cells [33,34,35].

In the last few years, some of the FDA-approved VEGFR-2 inhibitors, including sorafenib (**I**), regorafenib (**II**), lenvatinib (**III**), nintedanib (**IV**), sunitinib (**V**) and pazopanib (**VI**), were employed for clinical studies either alone or in combination with some other chemotherapies for the treatment of HCC [36]. In 2007, sorafenib (**I**) was approved by the FDA for the treatment of HCC patients. Sorafenib (**I**) is a multiprotein kinase inhibitor that successfully downregulates VEGF signaling, resulting in minimization of the pathological angiogenesis. Consequently, it reduces the proliferation and migration of tumor cells; thus, it prolongs HCC patients’ survival [11,37]. Despite the progress achieved, the observed survival was found to be dependent on the patients’ individual sensitivity, and it lasts for one year at most [38,39]. In 2017, regorafenib (**II**) was approved by the FDA for the treatment of HCC patients whose therapy was not successful with sorafenib (**I**) [40,41]. In 2018, lenvatinib (**III**) was approved by the FDA as a first-line treatment for advanced and unresponsive patients with HCC [28,42]. Despite the reported progress in HCC treatment, the continuous emergence of acquired resistance by the cancer cells towards tyrosine kinase inhibitors makes the search for new scaffolds with promising antiangiogenic and cytotoxic activity a continuous demand for cancer treatment [43].

Recently, 2-furylbenzimidazole scaffold has attracted much attention because of its promising angiokinase inhibitory activity [44,45,46]. For instance, NP184 (**VII**) was identified as a potent antiangiogenic agent [44,45]. Moreover, Pj-8 (**VIII**) significantly inhibited VEGFR-2 and suppressed tumor-induced angiogenesis in vivo (Figure 3) [46].

Motivated by these previous findings, our group has recently reported the design, synthesis and antiproliferative activity of novel 2-furylbenzimidazole derivatives [17]. They were designed through structural optimization of the known VEGFR-2 inhibitor NP184 (**VII**), e.g., compound **IX** (Figure 4). The designed molecules successfully showed promising VEGFR-2 inhibitory activity in comparison to their parent compound NP184 (**VII**). In silico, molecular docking simulations showed that the 2-furylbenzimidazole scaffold occupies the allosteric hydrophobic back pocket, while the chain in position one of the benzimidazole moiety extends to the gate area stabilizing the molecule through two hydrogen bonds with the key amino acids Glu885 and Asp1046, achieving VEGFR-2 inhibition in a type III inhibitor-like binding mode [17,47].

Against this background, we pursued our research through structural optimization of the 2-furylbenzimidazole derivative **IX** by the design and synthesis of a novel series of VEGFR-2 inhibitors targeting hepatocellular carcinoma based on the benzimidazole scaffold. As can be seen in Figure 4, our goal in this work is two-fold. The first is to study the effect of replacing the 5-methylfuryl moiety at the two-position of **IX** with an isopropyl moiety in **X** on the hydrophobic interaction with the allosteric hydrophobic back pocket and further its impact on the VEGFR-2 inhibition; moreover, it could achieve a better accommodation of the benzimidazole moiety in the back pocket. The second is to further optimize the 2-substituted benzimidazole structures **IX** and **X** through extension of the side chain at the one-position in series **XI** to get in proximity to Cys919 to catch interaction with it. Hence, shift them from being type III inhibitors into the more potent type II inhibitors (Figure 4).

In this study, the 1,2-disubstituted benzimidazole derivatives **X** and **XI** were designed and synthesized. The novel benzimidazoles were screened in vitro for their cytotoxic activity against the hepatocellular carcinoma cell line (HepG2). Simultaneously, some compounds were evaluated in the National Cancer Institute (NCI) in the division of cancer treatment and diagnosis, NIH, Bethesda, Maryland, USA for their in vitro antiproliferative activity against 60 cancer cell lines at 10 µM. In addition, evaluation of VEGFR-2 inhibitory activity of the designed compounds in HepG2 cell lines was performed. Selected compound(s) were evaluated biochemically for their inhibitory activity against VEGFR-2, FGFR-1 and PDGFR-β. The most potent compound was subsequently selected to study its effect on the HepG2 cell cycle and cell apoptosis. A molecular docking study was carried out to investigate the plausible binding mode of the newly synthesized compounds in the VEGFR-2 binding site and to study their interaction with VEGFR-2 hot spots (key amino acids).

## 2. Results

### 2.1. Chemistry

**Scheme 1:**
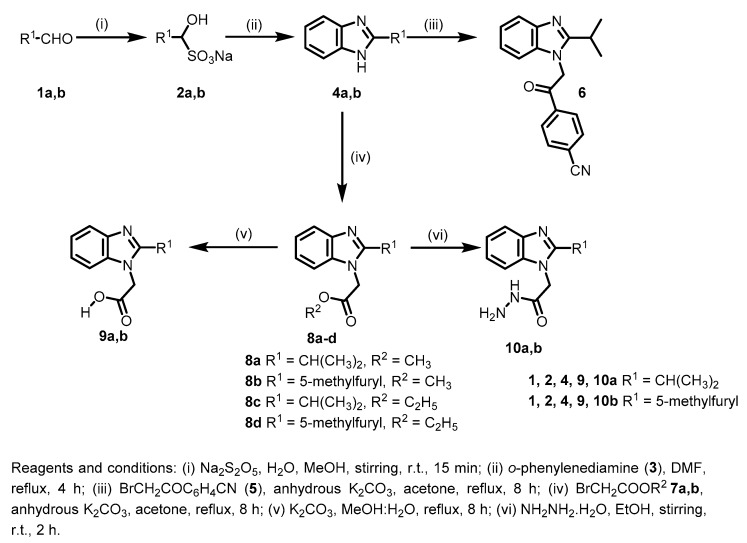
Synthesis of compounds **4,6,8**–**10**.

For the synthesis of the target compounds, 2-isopropyl-1*H*-benzo[*d*]imidazole (**4a**) and 2-(5-methylfuran-2-yl)-1*H*-benzo[*d*]imidazole (**4b**) were initially synthesized by the reaction of isobutyraldehyde (**1a**) or 5-methylfurfural (**1b**) with Na_2_S_2_O_5_ to obtain the corresponding bisulfite adducts **2a**,**b**, respectively. Subsequently, **2a,b** were reacted with 1,2-phenylenediamine (**3**) in DMF under reflux to give the corresponding 2-substituted benzimidazoles **4a** and **4b**, respectively [17]. Reaction of **4a** with 2-bromo-4′-cyanoacetophenone (**5**) afforded compound **6** in good yield. Treatment of 2-substituted benzimidazoles **4a**,**b** with either methyl or ethyl bromoacetate **7a** or **7b** gave the corresponding *N*-alkylated products **8a,c** and **8b,d**, respectively [17]. Hydrolysis of the formed esters **8a–d** was carried out in methanol-water under basic conditions to afford the corresponding acids **9a**,**b** [17]. Concurrently, reactions of **8a–d** with hydrazine hydrate in ethanol gave the corresponding acetohydrazides **10a,b** (Scheme 1).

**Scheme 2:**
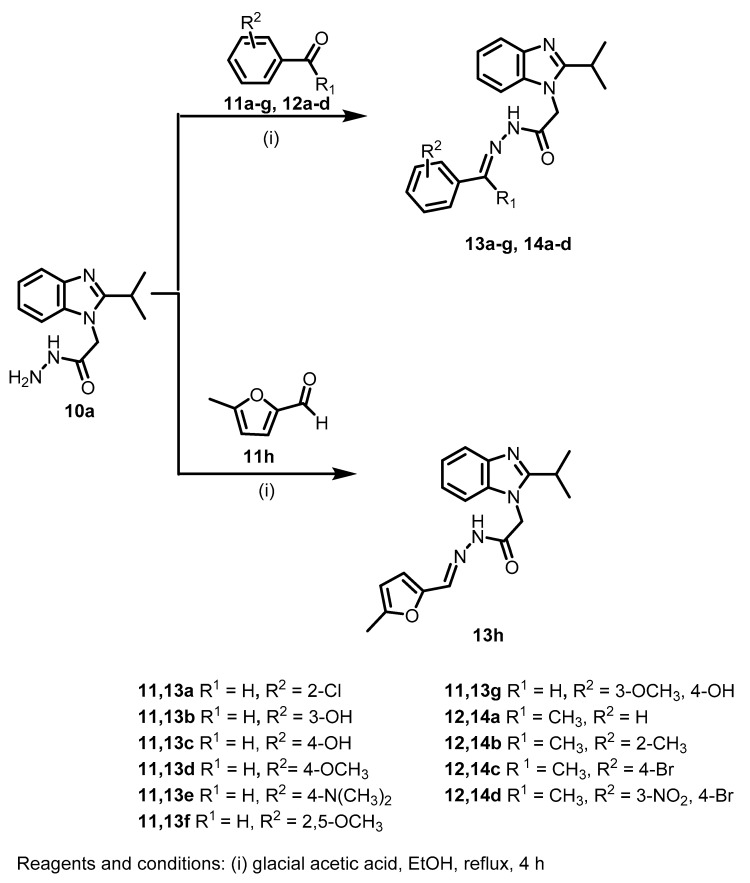
Synthesis of the target Schiff bases **13a**–**h** and **14a**–**d**.

The acetohydrazide **10a** was then reacted with different aldehydes **11a**–**h** and ketones **12a**–**d** to afford the corresponding Schiff bases **13a**–**h** and **14a**–**d**, respectively (Scheme 2).

**Figure 5:**
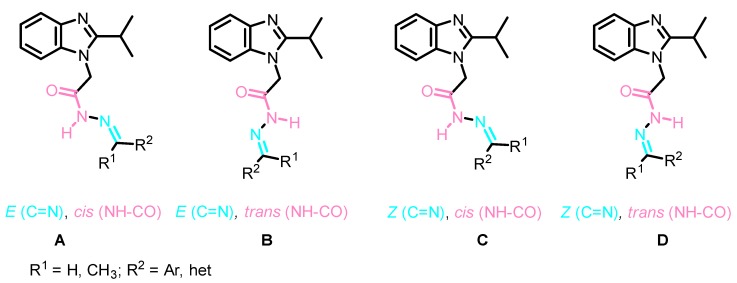
General stereochemistry of the four possible isomeric forms **A**–**D**.

**Figure 6:**
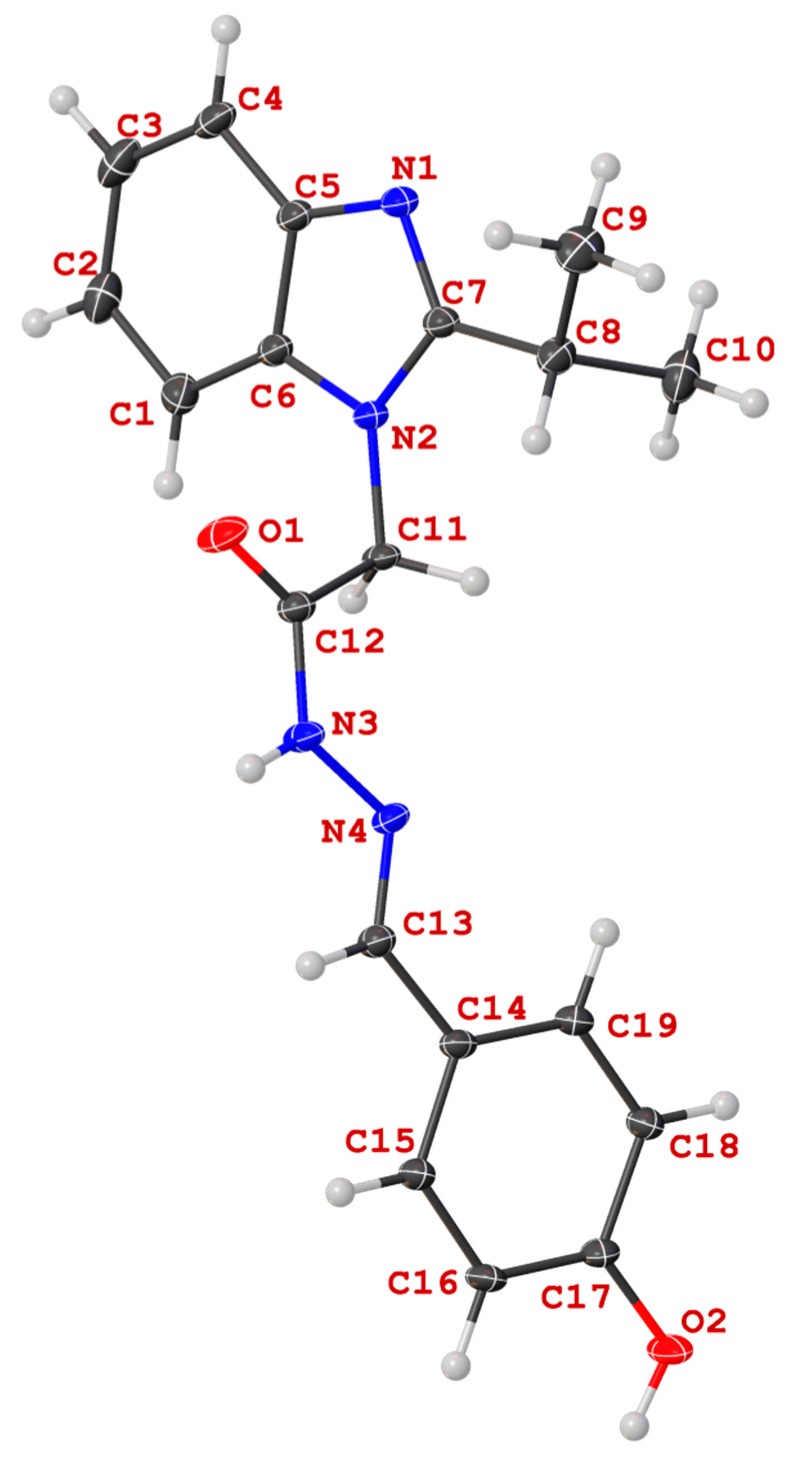
Molecular structure of *N*′-((4-hydroxyphenyl)methylidene)-2-(2-(propan-2-yl)-1*H*-benzimidazol-1-yl)acetohydrazide (**13c**) in the crystal.

**Figure 7:**
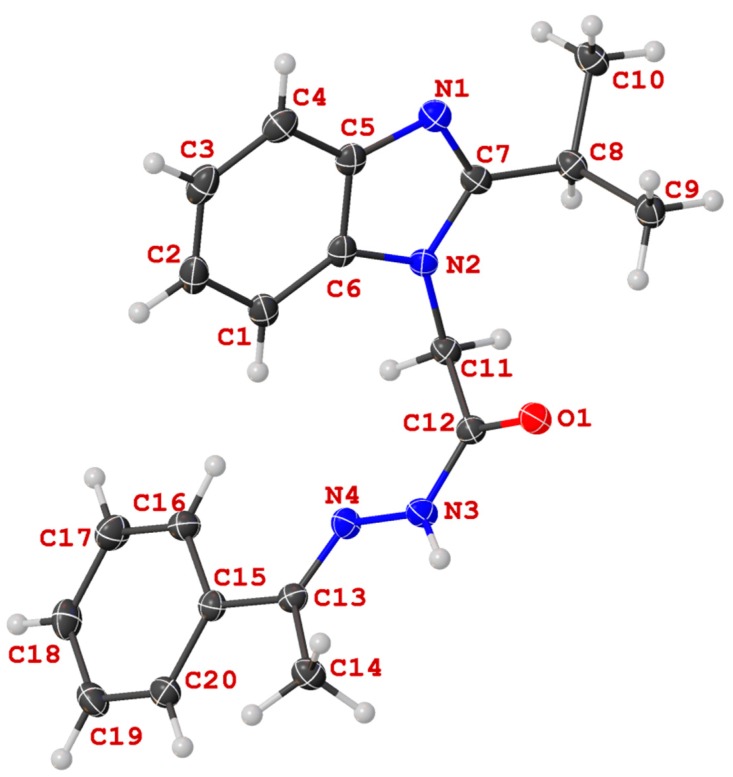
View of the structure of (*N*′-(-1-phenylethylidene)-2-(2-(propan-2-yl)-1*H*-benzimidazol-1-yl)acetohydrazide (**14a**) in the crystal.

The structures of all Schiff bases **13a**–**h** and **14a**–**d** were unambiguously elucidated by NMR spectroscopy, as well as by X-ray crystal structure analysis of two representative derivatives: **13c** and **14a**. Analysis of the ^1^HNMR and ^13^CNMR spectra of the obtained Schiff bases showed the duplication of signals which was rationalized to the presence of either *E/Z* geometrical isomers around the C=N or *cis/trans* conformers on the CO-NH (Figure 5) [48]. However, it was reported that the *N*-acylhydrazones that results from the reaction between hydrazides and aromatic aldehydes favor the sterically less-hindered geometric *E* isomers **A, B** [49,50,51]. This result was also confirmed by our X-ray study (Figure 6 and Figure 7). Therefore, the duplication of signals was rationalized to the presence of a mixture of *E,cis* **A** and *E,trans* **B** conformers in different ratios in the NMR solvent (Figure 5) (for further details, see experimental part and SI).

Unequivocal evidence for the structures of **13c** and **14a** was provided by X-ray crystal structure analysis. The molecular structures of **13c** and **14a** are depicted in Figure 6 and Figure 7.

**Scheme 3:**
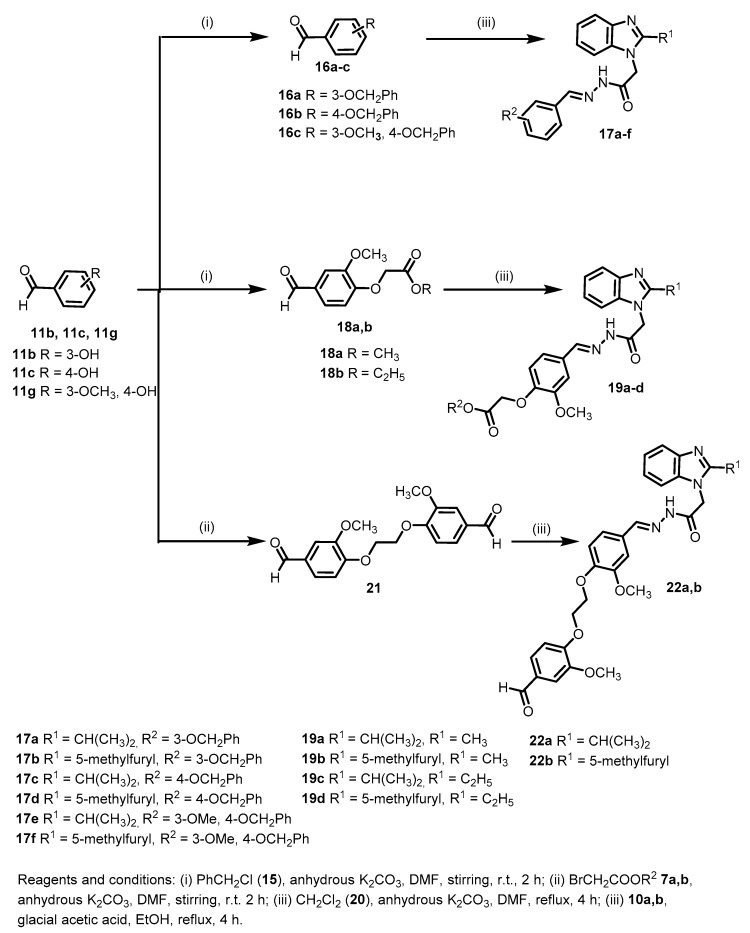
Synthesis of target compounds **17**, **19** and **22**.

For the synthesis of the target compounds **17a**–**f**, 3-hydroxybenzaldehyde (**11b**), 4-hydroxybenzaldehyde (**11c**) or vanillin (**11g**) was reacted with benzyl chloride (**15**) to afford the corresponding benzyloxybenzaldehydes **16a**–**c**. Subsequently, the acetohydrazides **10a**,**b** were reacted with the intermediates **16a**–**c** under acid catalyzed conditions to yield the corresponding Schiff bases **17a**–**f** (Scheme 3). In a similar way, vanillin (**11g**) was reacted with methyl bromoacetate (**7a**), ethyl bromoacetate (**7b**) or dichloroethane (**20**) under basic conditions to afford the corresponding intermediates **18a**,**b** and **21**, respectively. Reaction of the acetohydrazides **10a**,**b** with the intermediates **18a**,**b** or **21** in ethanol in the presence of catalytic amounts of acetic acid afforded the target compounds **19a**–**d** and **22a**,**b**, respectively (Scheme 3). The resulting target compounds **17a**–**f, 19a**–**d** and **22a**,**b** were found to exist in a mixture of two conformers in the solution state (^1^H NMR and ^13^C NMR).

**Scheme 4:**
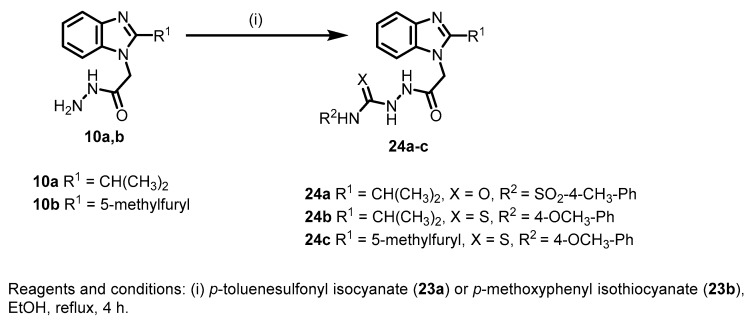
Synthesis of the target compounds **24a**–**c**.

In Scheme 4, 2-substitued-1*H*-benzo[*d*]imidazol-1-yl-acetohydrazides **10a**,**b** were reacted with *p*-toluenesulfonyl isocyanate (**23a**) or *p*-methoxyphenyl isothiocyanate (**23b**) in ethanol to afford the corresponding of the target compounds **24a**–**c**.

### 2.2. Biological Studies

The cytotoxic activities of the synthesized compounds against the human hepatocellular carcinoma (HepG2) cell line were screened [52]. Concurrently, some of the synthesized 1,2-disubstituted benzimidazoles were selected by the NCI (Bethesda, Rockville, MD, USA) to be evaluated in vitro for their effects on the growth of a panel of 60 cell lines at 10 μM. Subsequently, the effects of the target compounds on the VEGFR-2 inhibitory activity in the HepG2 cell line was evaluated using a VEGFR-2 ELSIA kit according to the manufacturer´s protocol. The most potent compound, **17a**, was further evaluated for its effect on the cell cycle and its apoptotic effect on the HepG2 cell line. 

#### 2.2.1. In Vitro Anti-Proliferative Activity

The newly synthesized 1,2-disubstituted benzimidazole derivatives were screened for their in vitro cytotoxic activity against the HepG2 cell line using the Sulfo-Rhodamine-B (SRB) assay, and the results were compared with sorafenib (**I**) as a reference standard [52]. The IC_50_ values are presented in Table 1. The observed IC_50_ values showed that some of the newly synthesized compounds **17a**, **24c** and **17b** are with a high potent antiproliferative activity with IC_50_ of 1.98, 8.73 and 10.04 μM, respectively, in comparison to sorafenib (**I**) (IC_50_ = 10.99 μM) and to **IX** (IC_50_ = 22.58 μM) [17]. Compounds **6**, **13d**, **13f**, **13g** and **17d** exhibited a potent cytotoxic activity with an IC_50_ range of 11.93–15.55 μM. In addition, compounds **14a**, **14b**, **17c** and **17e** showed a moderate in vitro antiproliferative activity against the HepG2 cell line with an IC_50_ range of 20.18–26.16 μM.

Worth mentioning here is that in series **13a**–**h**, compounds **13d**, **13f** and **13g** with a methoxy-substituted phenyl moiety exhibited a potent cytotoxic activity with IC_50_ of 11.93, 16.67 and 13.90 μM, in comparison to the analogues having chlorine, hydroxy or dimethylamino-substituted phenyl groups **13a**–**c**, **13e** (IC_50_ = 86.97–158.14 μM) or 5-methylfuryl moiety **13h** (IC_50_ = 59.85 μM). In the case of series **14a**–**d**, the introduction of a phenyl or 2-methylphenyl group in **14a** and **14b** resulted in a more promising activity than the 4-bromophenyl derivatives **14c** and **14d** (IC_50_ = 25.88 and 26.16 μM, respectively vs. 112.35 and 62.00 μM, respectively).

In series **17a**–**f**, incorporation of a 3-(benzyloxy)phenyl group in **17a** and **17b** resulted in a potent cytotoxic activity (IC_50_ = 1.98 and 10.04 μM, respectively). Whereas, shifting to the 4-(benzyloxy)phenyl group in **17c** and **17d** slightly decreased the potency, giving IC_50_ of 20.18 and 15.55 μM, respectively. A further decrease in activity was observed upon the introduction of the 3-methoxy-4-(benzyloxy)phenyl group in compounds **17e** and **17f** (IC_50_ = 24.11 and 42.49 μM, respectively). The 2-isopropylbenzimidazole derivatives **17a** (IC_50_ = 1.98 μM) and **17e** (IC_50_ = 24.11 μM) showed higher potency over the 2-furylbenzimidazole congeners **17b** (IC_50_ = 10.04 μM) and **17f** (IC_50_ = 42.49 μM), while **17d** (IC_50_ = 15.55 μM) with a 2-furylbenzimidazole moiety displayed higher potency in comparison to the corresponding isopropyl derivative **17c** (IC_50_ = 20.18 μM).

In series **19a**–**d**, introduction of phenoxy acetates resulted in a very weak cytotoxic activity (IC_50_ = 71.73–133.39 μM). In series **22**, both **22a** and **22b** were totally inactive (IC_50_ > 100 μM). The 2-furylbenzimidazole derivative **24c** showed more promising activity than the 2-isopropylbenzimidazole one **24b** (IC_50_ = 8.73 vs. 15.92 μM, respectively). 

#### 2.2.2. In Vitro One Dose (10 μM) Anticancer Assay on NCI 60 Cell Line Panel 

Some of the synthesized compounds were selected by the NCI, Bethesda, MD, USA for evaluation of their anticancer activity. An in vitro one dose (10 μM) anticancer assay was conducted on a full NCI 60-cell line panel derived from 9 different cancer types. Table 2 presents the percent of growth inhibition of some cell lines. The presented results revealed that the tested 1,2-disubstituted benzimidazole-based compounds have a different selectivity pattern against the various NCI cell lines panel. K-562 and MOLT-4 from leukemia, NCI-H522 from non-small cell lung cancer, HCT-116 from colon cancer, SK-MEL-5 and UACC-62 from melanoma, UO-31 from renal cancer, PC-3 from prostate cancer and HS 578T and T-47D from breast cancer are the most sensitive cell lines to the tested compounds. No growth inhibition was observed from the selected compounds against COLO205, HCC-2998, MALME-3M, SK-MEL-28, DU-145 and BT-549 cell lines. Compounds **17a** and **17b** showed a broad spectrum of antiproliferative activity against most of the cell lines. At 10 µM, **17a** displayed 41%, 32%, 57%, 50% and 64% growth inhibition against CCRF-CEM, HL-60(TB), K-562, MOLT-4 and SR cell lines from leukemia, respectively. Against colon cancer, **17a** showed 66% growth inhibition on HCT-116. It also displayed 50% and 41% growth inhibition on the melanoma cell lines SK-MEL-5 and UACC-62, respectively. Against UO-31 from renal cancer and PC-3 from prostate cancer, it inhibited the growth of 44% and 58% of the cancer cells, respectively. In addition, **17a** showed GI% ranging from 20% to 47% against breast cancer cell lines MDA-MB-231/ATTC, HS 578T, BT-549, T-47D and MDA-MB-468.

#### 2.2.3. In Vitro Growth Inhibitory Activity of 17a and 17b on Normal Human Skin Fibroblast (HSF)

The most potent compounds **17a** and **17b** were further evaluated for their growth inhibitory activity on human skin fibroblasts as an example of a normal cell line, and the results were presented in Table 3. It was found that compounds **17a** and **17b** displayed IC_50_ = of 19.90 and 4.80 µM, respectively. From the obtained results, it is obvious that compound **17a** (IC_50_ = 19.90 µM) showed about ten-fold higher selectivity to the HepG2 cell line (IC_50_ = 1.98 µM) over the normal HSF cell line, while compound **17b** was found to be toxic to normal cells (IC_50_ = 4.80 µM).

#### 2.2.4. In Vitro Cellular VEGFR-2 Inhibition Assessment

The inhibitory activity of the synthesized compounds on VEGFR-2 in the HepG2 cell line at their previously determined IC_50_ was evaluated using an enzyme-linked immunosorbent assay (ELISA) assay kit (Table 4). Some of the newly synthesized compounds exert moderate to strong VEGFR-2 inhibitory activity relative to the negative control with a range of 56% to 82% inhibition in comparison to sorafenib (**I**), which showed 92% inhibition. Compounds **17a** and **6** showed 82% and 80% inhibition, respectively, whereas, compounds **13d**, **13f** and **17b** showed 78% inhibition for VEGFR-2 in the HepG2 cell line.

#### 2.2.5. Biochemical Kinase Assay 

The most potent compounds **13d, 13f**, **17a** and **17b** were further evaluated biochemically for their inhibitory activity on VEGFR-2 using a VEGFR-2 (KDR) Kinase Assay Kit, and the results were presented in Table 5. From the obtained results, it was apparent that the tested compounds displayed potent inhibitory activity against VEGFR-2, with IC_50_ ranging from 0.09 to 0.40 µM in comparison to sorafenib (**I**, IC_50_ = 0.10 µM). Based on the results obtained from the cell-based assay and biochemical assay, compound **17a** was further selected to be evaluated for its inhibitory activity on FGFR-1 and PDGFR-β, and the results were presented in Table 5. The obtained results demonstrated that compound **17a** showed potent inhibitory activity against both FGFR-1 and PDGFR-β, with IC_50_ = 0.11 and 0.05 µM, respectively. These results demonstrated that compound **17a** acts not only as a VEGFR-2 inhibitor, but also, it has triple angiokinase properties against VEGFR-2, FGFR-1 and PDGFR-β, which emphasizes that it will be a promising candidate that can be further optimized for the discovery of targeted anticancer agents.

#### 2.2.6. Analysis of Cell Cycle Distribution

**Figure 8:**
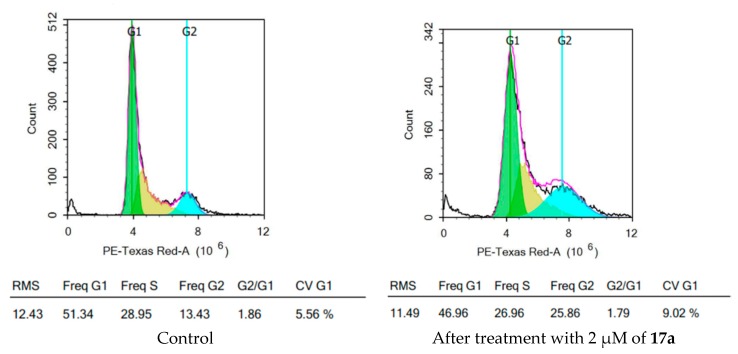
HepG2 cell cycle analysis before and after treatment with 2 µM of **17a**.

Based on the promising antiproliferative activity and antiangiogenic activity of series **17**, compound **17a** was further assayed for its effect on the cell cycle distribution by flow cytometric analyses of propidium iodide-stained nuclei at 2 µM. Cell cycle parameters were compared for HepG2 cells with DMSO as control and after treatment with **17a** and incubation for 48 h, and the results were depicted in Figure 8. From the obtained results, it is obvious that there is a decrease in the percent of cell distribution in the G1 phase, from 51.34% in control to 46.96% in treated cells, and an increase in the percent of cells accumulated in the G2 phase, from 13.43% in the control to 25.86% in the treated cells. This result indicated that compound **17a** arrests the cell cycle at the G2/M phase.

Moreover, in order to examine the effect of compound **17a** on cell apoptosis (programed cell death), the morphological markers of apoptosis of the HepG2 cell line were examined before and after treatment with **17a**. This assay is based on the fact that cells performing apoptosis translocate their phosphatidylserine (PS) phospholipid to the cell surface, which, in turn, can be easily detected by staining with a fluorescent conjugate of annexin V, followed by flow cytometry analysis. Concurrently, the HepG2 cells were stained with propidium iodide (PI), which enters only cells with damaged plasma membranes. This stain allows the discrimination between early apoptotic cells (positive for PS, but negative for PI) from late apoptotic and necrotic cells (positive for both PS and PI).

**Figure 9:**
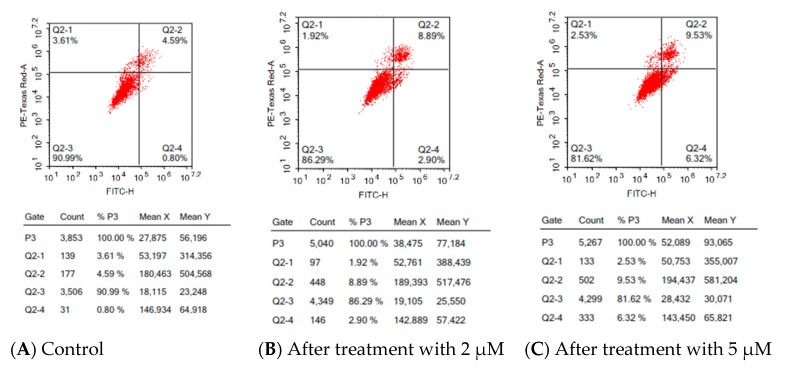
Effects of compound **17a** on HepG2 cell apoptosis at (**B**) 2 µM and (**C**) 5 µM in comparison to control untreated cells (**A**). The four quadrants are identified as: necrosis phase (Q1), late apoptosis phase (Q2), normal intact cells (Q3) and apoptosis phase (Q4).

Figure 9, shows HepG2 cells receiving no treatment possess apoptotic cell populations less than 5.0%. The treatment of HepG2 with **17a** at a concentration of 2 μM and 5 μM resulted in a dose-dependent increase in the early apoptotic phase, from 0.80% to 2.90% and 6.32%, respectively. Additionally, dose-dependent increases in the late apoptotic phase, from 4.59% in control to 8.89% and 9.53% after treatment with 2 and 5 µM, respectively, were observed. 

### 2.3. Molecular Docking Study and Structure Activity Relationship (SAR)

In order to rationalize the promising VEGFR-2 inhibitory activity of the newly synthesized 1,2-disubstituted benzimidazoles, in silico molecular docking of the target candidates in the VEGFR-2 active site was performed employing Molecular Operating Environment (MOE, 2010.10) software. The crystal structure of VEGFR-2 co-crystallized with sorafenib (**I**) as a type II inhibitor (PDB ID: 4ASD) in the inactive “DFG-out” conformation was downloaded from the protein databank [26].

Prior to the molecular docking of the newly synthesized compounds, self-docking of sorafenib (**I**), the co-crystallized ligand, in the VEGFR-2 active site was initially performed in order to confirm the validity of the applied protocol for further molecular docking studies. The validation step showed the ability of the docked pose to regenerate the experimental binding pattern of the co-crystallized sorafenib (**I**) with an energy score (S) = −15.19 kcal/mol and a low RMSD of 0.470Å between the docked pose and the co-crystalized sorafenib (**I**). Besides, the docked pose showed all the noncovalent interactions experimentally performed by the co-crystallized ligand, sorafenib (**I**), with the key amino acids (hot spots) in the active site (Glu885, Cys919 and Asp1046) (for further details, see SI). 

The validated molecular docking protocol was subsequently applied for further simulation studies. The target candidates were docked in the VEGFR-2 active site implementing the same parameters of the validated setup. The synthesized compounds efficiently occupied the binding site of VEGFR-2 with docking scores ranging from −11.27 to −16.23 kcal /mol (Table 6).

**Figure 10:**
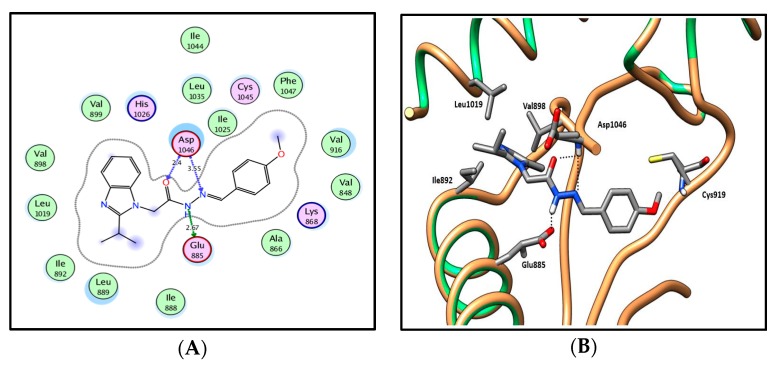
2D diagram (**A**) and 3D representation (**B**) of compound **13d** showing its interaction with the VEGFR-2 active site.

In silico molecular docking results of series **13a**–**h** and **14a**–**d**, which exhibit 2-isopropyl moiety at the two position, displayed the previously reported general predicted binding pattern of the 1-substituted-2-furylbenzimidazole, e.g., **IX** [17]. This binding pattern involves the accommodation of the 2-substituted benzimidazole moiety in the allosteric hydrophobic back pocket and its stabilization through hydrophobic interactions with the hydrophobic side chains of Ile888, Leu889, Ile892, Val898, Val899, Leu1019 and Ile1044 (Figure 10; for further details, see SI). The hydrazide-hydrazone moiety in series **13a**–**h** and **14a**–**d** is involved in hydrogen-bonding interactions with the side chain carboxylate of Glu885 of the αC helix and/or with Asp1046 in the conserved DFG motif. This binding pattern directs the (un)substituted phenyl moiety towards the hydrophobic gate area, resulting in hydrophobic interactions with the hydrophobic side chains of Val848, Lys868, Leu889, Val916 and Phe1047, which enhances the interactions in a type III inhibitor’s binding manner. 

In series **13a**–**e**, where various monosubstituted phenyl moieties are introduced, the 4-methoxyphenyl derivative **13d** was the most promising, with a VEGFR-2 inhibitory activity of IC_50_ = 0.09 µM, 78% inhibition of cellular VEGFR-2 and a docking energy score of −11.75 kcal/mol. On the contrary, introducing 2-chlorophenyl **13a**, 3-hydroxyphenyl **13b**, 4-hydroxyphenyl **13c** or 4-dimethylaminophenyl **13e** groups showed very weak cellular VEGFR-2 inhibitory activity (4–10%) and docking scores between −11.64 to −12.78 kcal/mol. This weak in vitro activity could be attributed to their poor cellular permeation.

Introducing a disubstituted phenyl moiety including 3-methoxy-4-hydroxyphenyl **13g** or 2,5-dimethoxyphenyl **13f** groups results in an apparent increase in the VEGFR-2 inhibitory activity, as well as in the predicted binding energy scores. The 3-methoxy-4-hydroxyphenyl derivative **13g** results in 64% VEGFR-2 inhibition and a docking score of −13.20 kcal/mol, while compound **13f** with a 2,5-dimethoxyphenyl group showed IC_50_ = 0.40 µM against VEGFR-2, 78% inhibition of cellular VEGFR-2 and a docking score of −13.23 kcal /mol. 

The replacement of the phenyl group of **13a**–**g** with a 5-methylfuryl moiety in **13h** resulted in a weak inhibition of VEGFR-2 of 6%, as well as low docking energy of −11.27 kcal/mol.

In series **14a**–**d**, it was found that the nature of the substituent on the phenyl moiety has a great influence on the activity. Introducing an unsubstituted phenyl group in **14a** gave moderate potency against VEGFR-2 (% inhibition = 51%), as well as a docking score of −11.87 kcal/mol. Further introduction of a methyl group at the two position **14b** enhances both the in vitro VEGFR-2 inhibitory activity and the in silico predicted binding energy scores, as evidenced by a VEGFR-2 inhibitory activity of 61% and a docking score of −12.33 kcal/mol. Although the introduction of bromo-substituted phenyl groups in **14c** and **14d** enhanced the binding affinity in comparison to the unsubstituted phenyl group (energy score = −12.23 and −14.08 versus −11.87 kcal/mol, respectively), an apparent reduction in the VEGFR-2 inhibitory activity was observed, which could be due to a poor cellular permeation.

Comparing the reported results of the recently designed and synthesized 2-furylbenzimidazole derivative **IX** (docking score = −13.44 kcal/mol) [17] with the current results demonstrated that compounds **13d,f,g** in series **13** and compounds **14a,b** in series **14** showed comparable docking scores (S = −11.75 to −13.23 kcal/mol) and slightly less potent VEGFR-2 inhibitory activity (% inhibition in the range of 51% to 78%). Meanwhile, the benzimidazole derivatives **13a**–**c,e** and **14c,d** displayed low potency against VEGFR-2 (% inhibition = 4%–10%). This decreased activity could be rationalized to their poor cellular permeation. 

**Figure 11:**
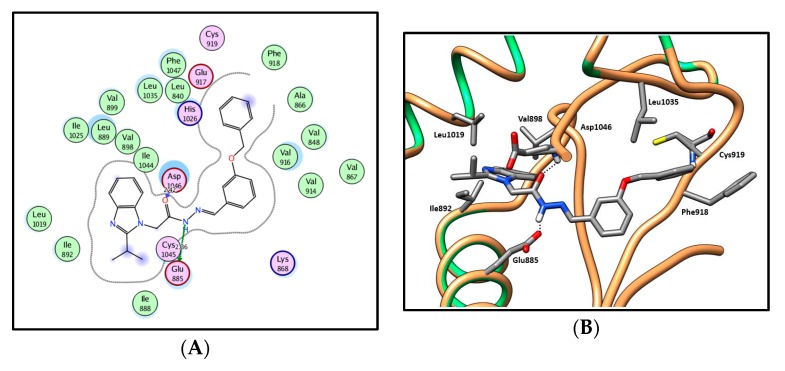
2D diagram (**A**) and 3D representation (**B**) of compound **17a** showing its interaction with the VEGFR-2 active site.

**Figure 12:**
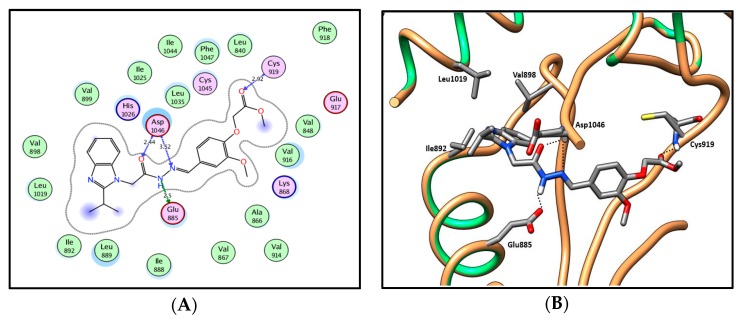
2D diagram (**A**) and 3D representation (**B**) of compound **19a** showing its interaction with the VEGFR-2 active site.

In an attempt to achieve our second goal, which aims to shift type III-like inhibitors in series **13a**–**h** and **14a**–**d** into type II-like inhibitors, further structural elongation was carried out on the phenyl moiety by introduction of benzyloxy groups, acetate groups or ethoxybenzaldehydes to afford series **17a**–**f**, **19a**–**d** and **22a,b,** respectively. Docking of these series into the VEGFR-2 active site reproduced the previously stated general binding pattern of the substituted benzimidazoles, vide supra. Moreover, the additional groups extend over the hinge region (front pocket) of the binding site and are involved in a hydrophobic interaction with the hydrophobic side chains of the amino acids Leu840, Phe918, Cys919, Leu1035 and Phe1047, as well as in a hydrogen-bonding interaction with the key amino acid Cys919, resulting in a higher affinity (Figure 11 and Figure 12; for further details, see SI). This was evidenced by the better docking scores, which ranged between −13.41 and −16.23 kcal/mol for series **17a**–**f**, **19a**–**d** and **22a,b** versus docking scores of −11.27 to −14.08 kcal/mol for the shorter series **13a**–**h** and **14a**–**d.**

In series **17a**–**f**, the presence of the benzyloxyphenyl extension had a great influence, as predicted, on the VEGFR-2 inhibitory activity. Compound **17a** was found to have IC_50_ = 0.11 µM. Additionally, moderate to potent inhibitory activity against cellular VEGFR-2 (56% to 82%) was observed. Only compound **17f** showed weak VEGFR-2 inhibitory activity (14%).

Despite the promising docking energy scores and predicted binding pattern of series **19a**–**d** (Figure 12; for further details, see SI), they demonstrated very weak VEGFR-2 inhibitory activity. Likewise, a strong binding interaction was displayed by series **22a,b** when the substituent at position one extended with a long chain, as indicated in their docking scores (Table 2). Surprisingly, this series gave weak cellular VEGFR-2 inhibitory activity. This unfavorable cellular activity could be due to their poor cellular permeation.

**Figure 13:**
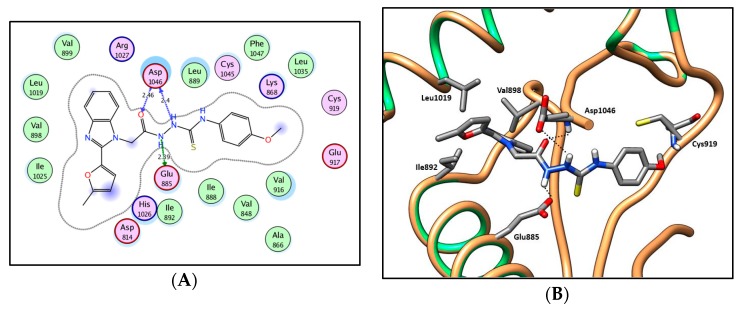
2D diagram (**A**) and 3D representation (**B**) of compound **24c** showing its interaction with the VEGFR-2 active site.

In series **24a**–**c** where the amide moiety of series **13a**–**h** and **14a**–**d** was replaced by uriedo or thiouriedo moieties, similar binding patterns to **13a**–**h** and **14a**–**d** were observed with the uriedo or the thiouriedo moieties, which are involved in hydrogen-bonding interactions with the key amino acids Glu885 and Asp1046 in the gate area between the ATP binding site and the allosteric hydrophobic back pocket (Figure 13; for further details, see SI). Compound **24a** with a uriedo moiety showed a very weak VEGFR-2 inhibitory activity of 10%. Replacement of the uriedo moiety with its thiouriedo congener resulted in an increase in the potency. Compounds **24b** and **24c** demonstrated 60% and 63% VEGFR-2 inhibitory activity and docking scores of −13.41 and −14.50 kcal /mol, respectively. Compound **24c,** which has 5-methylfuryl moiety at the two-position of the benzimidazole, showed slightly higher potency, as well as docking score.

In summary, the newly synthesized compounds adopted a common binding pattern in which the 2-substituted benzimidazole moiety is accommodated in the allosteric hydrophobic back pocket, achieving hydrophobic interactions with the hydrophobic side chains of Ile888, Leu889, Ile892, Val898, Val899, Leu1019 and Ile1044. The hydrazide-hydrazone moiety in series **13, 14, 17, 19** and **22** is involved in hydrogen-bonding interactions with the side chain carboxylate of Glu885 of the αC helix and/or with Asp1046 in the conserved DFG motif. Whereas, in series **24**, the uriedo or thioureido moiety accomplishes these interactions with Asp1046 and Glu885. This binding pattern directs the (un)substituted phenyl moiety of the newly synthesized compounds towards the hydrophobic gate area, resulting in hydrophobic interactions with the hydrophobic side chains of Val848, Lys868, Leu889, Val916 and Phe1047. Series **17**, **19** and **22,** with further extension on the distal phenyl moiety, showed additional binding interactions, where their additional extensions extend over the hinge region (front pocket) and are involved in a hydrophobic interaction with the hydrophobic side chains of the amino acids Leu840, Phe918, Cys919, Leu1035 and Phe1047, as well as in a hydrogen-bonding interaction with the key amino acid Cys919, resulting in a higher affinity. Although some compounds in the different series showed promising predicted binding patterns, as well as binding scores, their weak in vitro activity indicated their possible poor cellular permeation. From the obtained in vitro and in silico results, series **17** with the benzyloxyphenyl extension displayed the most promising cytotoxic and VEGFR-2 inhibitory activity, as well as docking scores, and so they are encouraging and to be further optimized as promising targeted anticancer agents.

## 3. Materials and Methods 

### 3.1. Chemistry

#### 3.1.1. General Remarks

Chemicals required for synthesis and biological experiments were purchased from commercial suppliers. Analytical thin layer chromatography (TLC) was carried out on precoated silica gel 60 F_245_aluminium plates (Merck, Darmstadt, Germany) with visualization under UV light. Melting points were determined with open capillary tubes on a Stuart SMP30 (Cole-Parmer Ltd., Staffordshire, United Kingdom) melting point apparatus and are uncorrected. Elemental analysis and spectral data of the compounds were performed in the Micro Analytical Labs, National Research Centre and Micro Analytical Laboratory Centre, Faculty of Science, Cairo University, Cairo, Egypt. IR spectra (4000–400 cm^−1^) were recorded using KBr pellets in a Jasco FT/IR 300E Fourier transform infrared spectrophotometer. ^1^H NMR and ^13^C NMR spectra were recorded at 400 (100) MHz on a Bruker instrument (Zurich, Switzerland) using DMSO-*d*_6_ as a solvent. Splitting patterns are abbreviated as s (singlet), d (doublet), t (triplet), q (quartet), m (multiplet), br. (broad) and ov. (overlapped).

#### 3.1.2. Synthesis and Analytical Data of Starting and Target Benzimidazoles 

*2-Isopropyl-1H-benzo[d]imidazole* (**4a**)

A saturated solution of Na_2_S_2_O_5_ (2.85 g, 15 mmol in 2 mL water) was added to a solution of isobutyraldehyde (**1a**) (1.08 g, 15 mmol) in methanol (15 mL), and the mixture was stirred at r.t. for 15 min. The mixture was left in the fridge overnight, and the precipitated bisulfite adducts **2a** was filtered and dried. Subsequently, a mixture of the formed adduct **2a** (1.76 g, 10 mmol) and 1,2-phenylenediamine (**3**) (1.08 g, 10 mmol) was refluxed in DMF (15 mL) for 4 h. The reaction mixture was cooled to room temperature and poured onto ice/water (50 mL) to give the crude product 4a (CAS No. 5851-43-4), which was collected by filtration and further purified by recrystallization from methanol to give 4a as a buff powder (1.40 g, 88%); mp: 233–235 °C; ^1^H-NMR (DMSO-*d*_6_, 400 MHz): *δ*_H_ 1.33 (d, ^3^*J* = 7.2 Hz, 6H), 3.12 (sep, ^3^*J* = 7.2 Hz, 1H), 7.08–7.10 (m, 2H), 7.44–7.45 (m, 2H), 12.10 ppm (s, 1H); Anal. Calcd. for C_10_H_12_N_2_: C, 74.97; H, 7.55; N, 17.48. Found: C, 74.59; H, 7.31; N, 17.25.

*2-(5-Methylfuran-2-yl)-1H-benzo[d]imidazole (**4b**) was synthesized according to the previously reported procedure* [17].

*4-(2-(2-Isopropyl-1H-benzo[d]imidazol-1-yl)acetyl)benzonitrile* (**6**)

A solution of **4a** (0.16 g, 1.5 mmol) and anhydrous K_2_CO_3_ (0.21 g, 1.5 mmol) was stirred in dry acetone (20 mL) at room temperature for 30 min. 2-Bromo-4′-cyanoacetophenone (**5**) (0.34 g, 1.5 mmol) was added, and the reaction mixture was refluxed for 8 h. The reaction mixture was then poured onto ice/water (100 mL) with continuous stirring, and the precipitated product was collected by filtration and recrystallized from ethanol to give analytically pure derivative **6** as a grey powder (0.37 g, 81%); mp 209–211 °C; IR (KBr): *ṽ* 3095, 2927, 2231, 1706, 1624, 1542, 1506, 1467 cm^−1^; ^1^H-NMR (DMSO-*d*_6_, 400 MHz) *δ*_H_ 1.31 (d, ^3^*J* = 6.8 Hz, 6H), 3.34 (sep, ^3^*J* = 6.8 Hz, 1H), 6.24 (s, 2H), 7.27–7.34 (m, 2H), 7.64 (d, ^3^*J* = 7.6 Hz, 1H), 7.69 (d, ^3^*J* = 7.6 Hz, 1H), 8.13 (d, ^3^*J* = 8.0 Hz, 2H), 8.29 ppm (d, ^3^*J* = 8.0 Hz, 2H); ^13^C-NMR (DMSO-*d*_6_, 100 MHz) *δ*_C_ 21.17, 25.34, 50.86, 111.48, 116.05, 116.52, 118.09, 123.37, 123.47, 129.23, 132.85, 134.06, 137.28, 160.08, 192.57 ppm; Anal. Calcd. for C_19_H_17_N_3_O: C, 75.23; H, 5.65; N, 13.85. Found: C, 75.55; H, 5.29; N, 13.53.

General procedure I for the synthesis of **8a,c**

A solution of **4a** and anhydrous K_2_CO_3_ was stirred for 30 min in dry acetone (20 mL). Methyl bromoacetate (**7a**) or ethyl bromoacetate (**7b**) was added drop-wise, and the mixture was stirred under reflux for 8 h. The reaction mixture was then poured onto ice / water (100 mL) with continuous stirring. The precipitated product was collected by filtration and recrystallized from ethanol to give analytically pure derivatives **8a,c**.

*Methyl 2-(2-isopropyl-1H-benzo[d]imidazol-1-yl)acetate* (**8a**)

According to the general procedure I, **4a** (2.40 g, 15 mmol), anhydrous K_2_CO_3_ (2.07 g, 15 mmol) and methyl bromoacetate (**7a**) (2.30 g, 15 mmol) were reacted in dry acetone (20 mL) to give **8a** as a grey powder (2.20 g, 63%); mp 93–95 °C; IR (KBr): *ṽ* 3042, 2975, 1744, 1613, 1512, 1459 cm^−1^; ^1^H-NMR (DMSO-*d*_6_, 400 MHz) *δ*_H_ 1.27 (d, ^3^*J* = 6.8 Hz, 6H), 3.19 (sep, ^3^*J* = 6.8 Hz, 1H), 3.70 (s, 3H), 5.21 (s, 2H), 7.15–7.18 (m, 2H), 7.42–7.44 (m, 1H), 7.55–7.57 (m, 1H); ^13^C-NMR (DMSO-*d*_6_, 100 MHz) *δ*_C_ 21.55, 25.47, 44.08, 52.40, 109.89, 118.50, 121.52, 121.77, 135.35, 142.10, 160.00, 168.96 ppm; Anal. Calcd. for C_13_H_16_N_2_O_2_: C, 67.22; H, 6.94; N, 12.06. Found: C, 67.45; H, 6.65; N, 11.72.

*Methyl 2-(2-(5-methylfuran-2-yl)-1H-benzo[d]imidazol-1-yl)acetate* (**8b**) was synthesized according to the previously reported procedure [17].

*Ethyl 2-(2-isopropyl-1H-benzo[d]imidazol-1-yl)acetate* (**8c**)

According to the general procedure I, **4a** (2.40 g, 15 mmol), anhydrous K_2_CO_3_ (2.07 g, 15 mmol) and ethyl bromoacetate (**7b**) (2.51 g, 15 mmol) were reacted in dry acetone (20 mL) to give **8c** as a white powder (2.30 g, 62%); mp 103–105 °C; IR (KBr): *ṽ* 3068, 2983, 1738, 1618, 1510, 1461 cm^−1^; ^1^H-NMR (DMSO-*d*_6_, 400 MHz): *δ*_H_ 1.20 (t, ^3^*J* = 7.2 Hz, 3H), 1.28 (d, ^3^*J* = 6.8 Hz, 6H), 3.18 (sep, ^3^*J* = 6.8 Hz, 1H), 4.16 (q, ^3^*J* = 7.2 Hz, 2H), 5.19 (s, 2H), 7.15–7.18 (m, 2H), 7.41–7.44 (m, 1H), 7.55–7.57 (m, 1H); ^13^C-NMR (DMSO-*d*_6_, 100 MHz): *δ*_C_ 14.00, 21.53, 25.53, 44.25, 61.33, 109.86, 118.50, 121.50, 121.77, 135.38, 142.11, 160.01, 168.44 ppm; Anal. Calcd. for C_14_H_18_N_2_O_2_: C, 68.27; H, 7.37; N, 11.37. Found: C, 68.61; H, 6.99; N, 11.05.

*2-(2-Isopropyl-1H-benzo[d]imidazol-1-yl)acetic acid* (**9a**)

A solution of **8a** (0.47 g, 2 mmol) and K_2_CO_3_ (0.28 g, 2 mmol) in methanol:water 10:1 mixture (10 mL) was refluxed for 4 h. Solvent was evaporated under reduced pressure, and the precipitated product was collected, washed and recrystallized from ethanol to give **9a** as gray needle crystals (0.40 g, 91%); mp 238–240 °C; IR (KBr) *ṽ* 3417, 2976, 2939, 1608, 1513, 1468 cm^−1^; ^1^H-NMR (DMSO-*d*_6_, 400 MHz): *δ*_H_ 1.30 (d, ^3^*J* = 6.8 Hz, 6H), 3.23–3.27 (m, 1H), 4.77 (s, 2H), 7.32–7.34 (m, 2H), 7.46–7.48 (m, 1H), 7.60–7.62 ppm (m, 1H); ^13^C-NMR (DMSO-*d*_6_, 100 MHz): *δ*_C_ 21.57, 26.73, 45.31, 110.06, 118.28, 121.34, 121.66, 135.58, 141.87, 160.16, 170.13, 172.35 ppm; Anal. Calcd. for C_12_H_14_N_2_O_2_: C, 66.04; H, 6.47; N, 12.84. Found: C, 66.32; H, 6.21; N, 12.52.

*2-(2-(5-Methylfuran-2-yl)-1H-benzo[d]imidazol-1-yl)acetic acid* (**9b**) was synthesized according to the previously reported procedure [17].

*2-(2-Isopropyl-1H-benzo[d]imidazol-1-yl)acetohydrazide* (**10a**)

Hydrazine hydrate (0.60 g, 12 mmol) was added drop-wise to a solution of **8a** (0.70 g, 3 mmol) in ethanol (15 ml). The reaction mixture was stirred at room temperature for 1 h and then poured onto ice/water (100 mL). The precipitated product was collected by filtration, washed with water and dried to afford **10a** as a white powder (0.50 g, 71%); mp: 243–245 °C; IR (KBr) *ṽ* 3433, 3292, 3163, 3073, 2965, 1646, 1552, 1508 cm^−1^; ^1^H-NMR (DMSO-*d*_6_, 400 MHz) *δ*_H_ 1.30 (d, ^3^*J* = 6.8 Hz, 6H), 3.23 (sep, ^3^*J* = 6.8 Hz, 1H), 4.34 (br., 2H), 4.82 (s, 2H), 7.13–7.16 (m, 2H), 7.38–7.40 (m, 1H), 7.53–7.55 (m, 1H), 9.53 ppm (s, 1H); ^13^C-NMR (DMSO-*d*_6_, 100 MHz) *δ*_C_ 21.67, 25.60, 44.17, 109.92, 118.42, 121.33, 121.55, 135.47, 142.09, 160.27, 166.18 ppm; Anal. Calcd. for C_12_H_16_N_4_O: C, 62.05; H, 6.94; N, 24.12. Found: C, 62.35; H, 6.67; N, 23.83.

*2-(2-(5-Methylfuran-2-yl)-1H-benzo[d]imidazol-1-yl)acetohydrazide* (**10b**) was synthesized according to the previously reported procedure [17].

General procedure II for the synthesis of Schiff bases **13a–h** and **14a–d**

A mixture of **10a** (1 mmol), aldehyde **11a**–**g** or ketone **12a**–**d** (1 mmol) and glacial acetic acid (1 mL) in ethanol (20 mL) was refluxed for 4 h. The reaction mixture was then poured onto ice/water (50 mL) and neutralized with dilute ammonia, and the precipitated product was filtered, dried and further purified by recrystallization from appropriate solvent to give the corresponding analytically pure compound.

*(E)-N′-(2-Chlorobenzylidene)-2-(2-isopropyl-1H-benzo[d]imidazol-1-yl)acetohydrazide* (**13a**) 

According to the general procedure II, **10a** (0.23 g, 1 mmol) was reacted with 2-chlorobenzaldehyde (**11a**) (0.14 g, 1 mmol) in ethanol in the presence of acetic acid (1 mL). Work-up followed by crystallization from methanol/dichloromethane (1:1) gave **13a** as a white powder (0.25 g, 71%); mp 211–213 °C; IR (KBr) *ṽ* 3428, 3063, 2962, 1679, 1634, 1634 cm^−1^; ^1^H-NMR (DMSO-*d*_6_, 400 MHz) of major conformer *δ*_H_ 1.29 (d, ^3^*J* = 6.8 Hz, 6H), 3.19 (sep, ^3^*J* = 6.8 Hz, 1H), 5.51 (s, 2H), 7.13–7.15 (m, 2H), 7.42–7.47 (m, 3H), 7.52–7.57 (m, 2H), 8.15 (dd, ^3^*J* = 7.6 Hz, ^4^*J* = 1.6 Hz, 1H), 8.47 (s, 1H), 11.95 ppm (s, 1H); ^1^H-NMR (DMSO-*d*_6_, 400 MHz) of minor conformer *δ*_H_ 1.32 (d, ^3^*J* = 7.2 Hz, 6H), 3.22 (sep, ^3^*J* = 6.8 Hz, 1H), 5.05 (s, 2H), 7.13–7.15 (ov. m, 2H), 7.42–7.47 (ov. m, 3H), 7.52–7.57 (ov. m, 2H), 7.94 (dd, ^3^*J* = 7.6 Hz, ^4^*J* = 1.2 Hz, 1H), 8.65 (s, 1H), 12.16 (s, 1H); ^13^C-NMR (DMSO-*d*_6_, 100 MHz) of major conformer *δ*_C_ 21.62, 25.58, 43.92, 109.91, 118.33, 121.14, 121.44, 127.28, 127.55, 129.87, 131.25, 131.48, 133.03, 135.78, 140.27, 142.17, 160.37 and 168.64 ppm; ^13^C-NMR (DMSO-*d*_6_, 100 MHz) of minor conformer *δ*_C_ 21.62, 25.58, 44.63, 109.81, 118.45, 121.33, 121.60, 126.90, 127.66, 129.92, 131.13, 131.69, 133.21, 135.54, 142.12, 143.49, 160.21 and 163.69 ppm; Anal. Calcd. for C_19_H_19_ClN_4_O: C, 64.31; H, 5.40; N, 15.79. Found: C, 64.63; H, 5.15; N, 15.53.

*(E)-N′-(3-Hydroxybenzylidene)-2-(2-isopropyl-1H-benzo[d]imidazol-1-yl)acetohydrazide* (**13b**)

According to the general procedure II, **10a** (0.23 g, 1 mmol) was reacted with 3-hydroxybenzaldehyde (0.12 g, 1 mmol) (**11b**) in the presence of acetic acid in ethanol. Work-up followed by crystallization from ethanol gave **13b** as white crystals (0.23 g, 69%); mp 249–251 °C; ^1^H-NMR (DMSO-*d*_6_, 400 MHz) of major conformer *δ*_H_ 1.30 (d, ^3^*J* = 6.8 Hz, 6H), 3.18 (sep, ^3^*J* = 6.8 Hz, 1H), 5.47 (s, 2H), 6.85 (dd, ^3^*J* = 7.2 Hz, ^4^*J* = 1.6 Hz, 1H), 7.13–7.20 (m, 4H), 7.25 (d, ^3^*J* = 8.0 Hz, 1H), 7.40-7.42 (m, 1H), 7.55–7.58 (m, 1H), 8.00 (s, 1H), 9.66 (s, 1H), 11.72 ppm (s, 1H); ^1^H-NMR (DMSO-*d*_6_, 400 MHz) of minor conformer *δ*_H_ 1.32 (d, ^3^*J* = 6.8 Hz, 6H), 3.23 (ov. sep, ^3^*J* = 6.8 Hz, 1H), 5.03 (s, 2H), 6.82 (ov. dd, ^3^*J* = 7.2 Hz, ^4^*J* = 2.0 Hz, 1H), 7.09 (d, ^3^*J* = 7.6 Hz, 1H), 7.13–7.20 (ov. m, 3H), 7.27 (d, ^3^*J* = 7.6 Hz, 1H), 7.40–7.42 (ov. m, 1H), 7.55–7.58 (ov. m, 1H), 8.17 (s, 1H), 9.66 (s, 1H), 11.72 ppm (s, 1H); ^13^C-NMR (DMSO-*d*_6_, 125 MHz) of major conformer *δ*_C_ 21.68, 25.78, 43.90, 110.01, 113.17, 117.54, 118.42, 118.65, 121.40, 121.72, 130.01, 135.33, 135.84, 142.14, 144.77, 157.78, 160.56 and 168.44 ppm; ^13^C-NMR (DMSO-*d*_6_, 125 MHz) of minor conformer *δ*_C_ 21.72, 25.76, 44.70, 109.96, 112.86, 117.80, 118.53, 119.06, 121.57, 121.82, 130.02, 135.33, 135.61, 142.11, 147.93, 157.78, 160.45 and 163.60 ppm; Anal. Calcd. for C_19_H_20_N_4_O_2_: C, 67.84; H, 5.99; N, 16.66. Found: C, 67.61; H, 6.32; N, 16.31. 

*(E)-N′-(4-Hydroxybenzylidene)-2-(2-isopropyl-1H-benzo[d]imidazol-1-yl)acetohydrazide* (**13c**)

According to the general procedure II, **10a** (0.23 g, 1 mmol) was reacted with 4-hydroxybenzaldehyde (**11c**) (0.12 g, 1 mmol) in the presence of acetic acid in ethanol. Work-up followed by crystallization from DMSO gave **13c** as colorless crystals (0.24 g, 72%); mp: 296–298 °C; IR (KBr): *ṽ* 3437, 3211, 3059, 2977, 1682, 1607, 1577, 1510 cm^−1^; ^1^H-NMR (DMSO-*d*_6_, 400 MHz) of major conformer: *δ*_H_ 1.29 (d, ^3^*J* = 6.8 Hz, 6H), 3.17 (sep, ^3^*J* = 6.8 Hz, 1H), 5.44 (s, 2H), 6.83 (d, ^3^*J* = 8.8 Hz, 2H), 7.12–7.14 (m, 2H), 7.39–7.42 (m, 1H), 7.55–7.57 (m, 1H), 7.61 (d, ^3^*J* = 8.4 Hz, 2H), 7.97 (s, 1H), 9.94 (s, 1H), 11.57 ppm (s, 1H); ^1^H-NMR (DMSO-*d*_6_, 400 MHz) of minor conformer: *δ*_H_ 1.31 (d, ^3^*J* = 6.8 Hz, 6H), 3.23 (sep, ^3^*J* = 6.8 Hz, 1H), 5.01 (s, 2H), 6.82 (d, ^3^*J* = 8.8 Hz, 2H), 7.14–7.16 (ov. m, 2H), 7.38–7.42 (ov. m, 1H), 7.55–7.57 (ov. m, 1H), 7.53 (d, ^3^*J* = 8.4 Hz, 2H), 8.15 (s, 1H), 9.94 (ov. s, 1H), 12.15 ppm (s, 1H); ^13^C-NMR (DMSO-*d*_6_, 100 MHz) of major conformer: *δ*_C_ 21.65, 25.72, 44.88, 109.99, 115.76, 118.37, 121.27, 121.60, 125.06, 128.93, 135.85, 142.14, 144.74, 159.46, 160.47, 168.10 ppm; ^13^C-NMR (DMSO-*d*_6_, 100 MHz) of minor conformer: *δ*_C_ 21.69, 25.69, 44.64, 109.91, 115.76, 118.49, 121.45, 121.71, 124.96, 129.07, 135.58, 142.10, 148.05, 159.64, 160.37, 163.16 ppm; Anal. Calcd. for C_19_H_20_N_4_O_2_: C, 67.84; H, 5.99; N, 16.66. Found: C, 67.53; H, 5.66; N, 16.91.

*(E)-2-(2-Isopropyl-1H-benzo[d]imidazol-1-yl)-N′-(4-methoxybenzylidene)acetohydrazide* (**13d**)

According to the general procedure II, **10a** (0.23 g, 1 mmol) was reacted with 4-methoxybenzaldehyde (**11d**) (0.14 g, 1 mmol) in the presence of acetic acid in ethanol. Work-up followed by crystallization from DMSO gave **13d** as colorless crystals (0.32 g, 91%); mp: 190–192 °C; IR (KBr): *ṽ* 3441, 2964, 2928, 1672, 1609, 1570, 1456 cm^−1^; ^1^H-NMR (DMSO-*d*_6_, 400 MHz) of major conformer *δ*_H_ 1.29 (d, ^3^*J* = 6.8 Hz, 6H), 3.18 (d, ^3^*J* = 6.8 Hz, 1H), 3.80 (s, 3H), 5.47 (s, 2H), 7.02 (d, ^3^*J* = 8.8 Hz, 2H), 7.12–7.14 (m, 2H), 7.39–7.43 (m, 1H), 7.55–7.57 (ov., 1H), 7.73 (d, ^3^*J* = 8.8 Hz, 2H), 8.02 (s, 1H), 11.65 ppm (s, 1H); ^1^H-NMR (DMSO-*d*_6_, 400 MHz) of minor conformer *δ*_H_ 1.31 (d, ^3^*J* = 6.8 Hz, 6H), 3.23 (d, ^3^*J* = 6.8 Hz, 1H), 3.79 (s, 3H), 5.01 (s, 2H), 7.04 (d, ^3^*J* = 8.8 Hz, 2H), 7.16–7.17 (m, 2H), 7.39–7.43 (ov. m, 1H), 7.55–7.57 (ov. m, 1H), 7.65 (d, ^3^*J* = 8.8 Hz, 2H), 8.21 (s, 1H), 11.78 ppm (s, 1H); Anal. Calcd. for C_20_H_22_N_4_O_2_: C, 68.55; H, 6.33; N, 15.99. Found: C, 68.21; H, 6.00; N, 16.27.

*(E)-N′-(4-(Dimethylamino)benzylidene)-2-(2-isopropyl-1H-benzo[d]imidazol-1-yl)acetohydrazide* (**13e**)

According to the general procedure II, **10a** (0.23 g, 1 mmol) was reacted with 4-dimethylaminobenzaldehyde (0.15 g, 1 mmol) (**11f**) in the presence of acetic acid in ethanol. Work-up followed by crystallization from dioxan/*n*-hexan (1:1) gave **13e** as orange crystals (0.24 g, 67%); mp: 225–227 °C; IR (KBr): *ṽ* 3441, 3056, 2964, 1672, 1607, 1456 cm^−1^; ^1^H-NMR (DMSO-*d*_6_, 400 MHz) of major conformer *δ*_H_ 1.29 (d, ^3^*J* = 6.8 Hz, 6H), 2.97 (s, 6H), 3.18 (sep, ^3^*J* = 6.8 Hz, 1H), 5.43 (s, 2H), 6.75 (d, ^3^*J* = 8.8 Hz, 2H), 7.12–7.14 (m, 2H), 7.38–7.41 (m, 1H), 7.50 (d, ^3^*J* = 8.8 Hz, 1H), 7.58 (d, ^3^*J* = 8.8 Hz, 2H), 7.94 (s, 1H), 11.49 (s, 1H); ^1^H-NMR (DMSO-*d*_6_, 400 MHz) of minor conformer *δ*_H_ 1.31 (d, ^3^*J* = 6.8 Hz, 6H), 2.98 (s, 6H), 3.23 (sep, ^3^*J* = 6.8 Hz, 1H), 4.98 (s, 2H), 6.76 (d, ^3^*J* = 8.8 Hz, 2H), 7.14–7.17 (m, 2H), 7.42-7.43 (m, 1H), 7.52 (d, ^3^*J* = 8.8 Hz, 1H), 7.55–7.56 (m, 2H), 8.11 (s, 1H), 11.60 (s, 1H); ^13^C-NMR (DMSO-*d*_6_, 100 MHz) of major conformer *δ*_C_ 21.69, 25.82, 43.92, 110.07, 111.90, 118.43, 121.36, 121.40, 121.74, 128.57, 128.74, 135.88, 142.13, 145.48, 151.67, 160.57, 162.95, 167.89; ^13^C-NMR (DMSO-*d*_6_, 100 MHz) of minor conformer *δ*_C_ 21.74, 25.77, 44.70, 109.99, 111.84, 118.54, 121.18, 121.58, 121.83, 128.57, 129.70, 135.61, 142.10, 148.68, 151.80, 152.16, 160.04, 160.50 ppm; Anal. Calcd. for C_21_H_25_N_5_O: C, 69.40; H, 6.93; N, 19.27. Found: C, 69.71; H, 6.65; N, 19.55.

*(E)-N′-(2,4-Dimethoxybenzylidene)-2-(2-isopropyl-1H-benzo[d]imidazol-1-yl)acetohydrazide* (**13f**)

According to the general procedure II, **10a** (0.23 g, 1 mmol) was reacted with 2,5-dimethoxybenzaldehyde (**11f**) (0.17 g, 1 mmol) in the presence of acetic acid in ethanol. Work-up followed by crystallization from dioxan/*n*-hexan (1:1) gave **13f** as orange needle crystals (0.32 g, 85%); mp 202–204 °C; IR (KBr) *ṽ* 3439, 2929, 2966, 1677, 1637, 1458 cm^−1^; ^1^H-NMR (DMSO-*d*_6_, 400 MHz) of major conformer *δ*_H_ 1.29 (d, ^3^*J* = 6.8 Hz, 6H), 3.19 (sep, ^3^*J* = 6.8 Hz, 1H), 3.75 (s, 3H), 3.81 (s, 3H), 5.49 (s, 2H), 7.03 (d, ^3^*J* = 2.8 Hz, 1H), 7.05 (s, 1H), 7.12–7.15 (m, 2H), 7.41–7.43 (m, 1H), 7.51 (d, ^3^*J* = 2.8 Hz, 1H), 7.55–7.57 (m, 1H), 8.38 (s, 1H), 11.74 (s, 1H); ^1^H-NMR (DMSO-*d*_6_, 400 MHz) of minor conformer 1.32 (d, ^3^*J* = 6.8 Hz, 6H), 3.24 (sep, ^3^*J* = 6.8 Hz, 1H), 3.70 (s, 3H), 3.81 (ov. s, 3H), 5.01 (s, 2H), 7.00 (d, ^3^*J* = 2.8 Hz, 1H), 7.07 (s, 1H), 7.16–7.19 (m, 2H), 7.27 (d, ^3^*J* = 2.8 Hz, 1H), 7.41–7.43 (ov. m, 1H), 7.55–7.57 (ov. m, 1H), 8.58 (s, 1H), 11.93 (s, 1H); ^13^C-NMR (DMSO-*d*_6_, 100 MHz) of major conformer *δ*_C_ 21.68, 25.73, 44.00, 55.66, 56.43, 110.07, 110.32, 113.37, 117.29, 118.40, 121.36, 121.66, 122.70, 135.85, 139.96, 142.13, 152.34, 153.39, 160.56, 168.51 ppm; ^13^C-NMR (DMSO-*d*_6_, 100 MHz) of minor conformer *δ*_C_ 21.71, 25.73, 44.73, 55.56, 56.39, 109.26, 109.93, 113.58, 118.14, 118.54, 121.56, 121.82, 122.45, 135.58, 139.96, 143.21, 152.46, 153.35, 160.43, 163.50 ppm; Anal. Calcd. for C_21_H_24_N_4_O_3_: C, 66.30; H, 6.36; N, 14.73. Found: C, 66.65; H, 6.03; N, 14.51. 

*(E)-N′-(4-Hydroxy-3-methoxybenzylidene)-2-(2-isopropyl-1H-benzo[d]imidazol-1-yl)acetohydrazide* (**13g**)

According to the general procedure II, **10a** (0.23 g, 1 mmol) was reacted with vanillin (**11g**) (0.15 g, 1 mmol) in the presence of acetic acid in ethanol. Work-up followed by crystallization from dioxan/*n*-hexan (1:1) gave **13g** as white crystals (0.29 g, 81%); mp: 165–167 °C; IR (KBr): *ṽ* 3446, 3186, 3034, 2970, 1697, 1594, 1512, 1459 cm^−1^; ^1^H-NMR (DMSO-*d*_6_, 400 MHz) of major conformer *δ*_H_ 1.30 (d, ^3^*J* = 6.8 Hz, 6H), 3.19 (sep, ^3^*J* = 6.8 Hz, 1H), 3.83 (s, 3H), 5.47 (s, 2H), 6.84 (d, ^3^*J* = 8.0 Hz, 1H), 7.12–7.16 (m, 3H), 7.38 (d, ^3^*J* = 1.2 Hz, 1H), 7.41–7.43 (m, 1H), 7.55–7.57 (m, 1H), 7.96 (s, 1H), 9.53 (s, 1H), 11.61 ppm (s, 1H); ^1^H-NMR (DMSO-*d*_6_, 400 MHz) of minor conformer *δ*_H_ 1.31 (d, ^3^*J* = 8.0 Hz, 6H), 3.23 (sep, ^3^*J* = 6.8 Hz, 1H), 3.78 (s, 3H), 5.00 (s, 2H), 6.82 (d, ^3^*J* = 8.0 Hz, 1H), 7.09 (dd, ^3^*J* = 8.4 Hz, ^3^*J* = 1.2 Hz, 1H), 7.12–7.16 (ov. m, 2H), 7.26 (d, ^3^*J* = 1.2 Hz, 1H), 7.41–7.43 (ov. m, 1H), 7.55–7.57 (ov. m, 1H), 8.14 (s, 1H), 9.55 (s, 1H), 11.72 ppm (s, 1H); ^13^C-NMR (DMSO-*d*_6_, 100 MHz) of major conformer *δ*_C_ 21.74, 25.87, 44.05, 55.88, 109.87, 110.16, 115.72, 118.49, 121.54, 121.86, 121.88, 125.63, 135.89, 142.13, 145.15, 148.22, 149.11, 160.70, 168.30 ppm; ^13^C-NMR (DMSO-*d*_6_, 100 MHz) of minor conformer *δ*_C_ 21.78, 25.85, 44.73, 55.76, 109.34, 110.05, 115.62, 118.60, 121.72, 121.97, 122.54, 125.51, 135.64, 142.10, 148.25, 148.50, 149.34, 160.60, 163.41 ppm; Anal. Calcd. for C_20_H_22_N_4_O_3_: C, 65.56; H, 6.05; N, 15.29. Found: C, 65.13; H, 5.79; N, 15.53.

*(E)-2-(2-Isopropyl-1H-benzo[d]imidazol-1-yl)-N′-((5-methylfuran-2-yl)methylene)acetohydrazide* (**13h**)

According to the general procedure II, **10a** (0.23 g, 1 mmol) was reacted with 5-methylfurfural (**11h**) (0.11 g, 1 mmol) in the presence of acetic acid in ethanol. Work-up followed by crystallization from ethanol gave **13h** as white crystals (0.24 g, 75%); mp: 131–133 °C; IR (KBr): *ṽ* 3450, 3100, 2919, 1689, 1618, 1592, 1512, 1452 cm^−1^; ^1^H-NMR (DMSO-*d*_6_, 400 MHz) of major conformer *δ*_H_ 1.28 (d, ^3^*J* = 6.8 Hz, 6H), 2.34 (s, 3H), 3.18 (sep, ^3^*J* = 6.8 Hz, 1H), 5.37 (s, 2H), 6.27 (d, ^3^*J* = 2.4 Hz, 1H), 6.84 (d, ^3^*J* = 3.2 Hz, 1H), 7.12–7.14 (m, 2H), 7.40–7.42 (m, 1H), 7.55–7.58 (m, 1H), 7.88 (s, 1H), 11.65 ppm (s, 1H); ^1^H-NMR (DMSO-*d*_6_, 400 MHz) of minor conformer *δ*_H_ 1.30 (d, ^3^*J* = 6.8 Hz, 6H), 2.31 (s, 3H), 3.23 (sep, ^3^*J* = 6.8 Hz, 1H), 5.01 (s, 2H), 6.24 (d, ^3^*J* = 2.8 Hz, 1H), 6.81 (d, ^3^*J* = 3.2 Hz, 1H), 7.16–7.19 (m, 2H), 7.40–7.42 (ov. m, 1H), 7.55–7.58 (ov. m, 1H), 8.05 (s, 1H), 11.65 ppm (s, 1H); ^13^C-NMR (DMSO-*d*_6_, 100 MHz) of major conformer *δ*_C_ 13.61, 21.67, 25.70, 43.89, 108.74, 109.98, 115.86, 118.38, 121.28, 121.61, 134.73, 135.81, 142.18, 147.55, 154.75, 160.49, 168.05 ppm; ^13^C-NMR (DMSO-*d*_6_, 100 MHz) of minor conformer *δ*_C_ 13.51, 21.67, 25.70, 44.65, 108.69, 109.90, 116.03, 118.51, 121.46, 121.72, 135.60, 137.46, 142.13, 147.59, 154.92, 160.34, 163.37 ppm; Anal. Calcd. for C_18_H_20_N_4_O_2_: C, 66.65; H, 6.21; N, 17.27. Found: C, 66.29; H, 6.57; N, 17.59.

*(E)-2-(2-Isopropyl-1H-benzo[d]imidazol-1-yl)-N′-(1-phenylethylidene)acetohydrazide* (**14a**)

According to the general procedure II, **10a** (0.23 g, 1 mmol) was reacted with acetophenone (0.12 g, 1 mmol) (**12a**) in the presence of acetic acid in ethanol. Work-up followed by crystallization from ethanol gave **14a** as buff needle crystals (0.28 g, 85%); mp: 176–178 °C; ^1^H-NMR (DMSO-*d*_6_, 400 MHz) of major conformer *δ*_H_ 1.30 (d, ^3^*J* = 6.8 Hz, 6H), 2.32 (s, 3H), 3.18 (sep, ^3^*J* = 6.8 Hz, 1H), 5.52 (s, 2H), 7.13–7.16 (m, 2H), 7.40–7.46 (m, 4H), 7.56–7.58 (m, 1H), 7.90–7.92 (m, 2H), 11.04 (s, 1H); ^1^H-NMR (DMSO-*d*_6_, 400 MHz) of minor conformer *δ*_H_ 1.33 (d, ^3^*J* = 6.8 Hz, 6H), 2.37 (s, 3H), 3.24 (sep, ^3^*J* = 6.8 Hz, 1H), 5.18 (s, 2H), 7.13–7.16 (ov. m, 2H), 7.40–7.46 (m, 4H), 7.56–7.58 (m, 1H), 7.76–7.80 (m, 2H), 10.90 (s, 1H); ^13^C-NMR (DMSO-*d*_6_, 100 MHz) of major conformer: *δ*_C_ 13.90, 21.72, 25.80, 44.52, 110.06, 118.44, 121.40, 121.72, 126.52, 128.57, 129.47, 135.89, 138.10, 142.17, 149.23, 160.64, 169.41 ppm; ^13^C-NMR (DMSO-*d*_6_, 100 MHz) of minor conformer: *δ*_C_ 14.48, 21.72, 25.80, 44.59, 110.01, 118.54, 121.56, 121.82, 126.52, 128.52, 129.62, 135.66, 138.04, 142.13, 153.09, 160.53, 164.21 ppm; Anal. Calcd. for C_20_H_22_N_4_O: C, 71.83; H, 6.63; N, 16.75. Found: C, 71.63; H, 6.91; N, 16.49.

*(E)-2-(2-Isopropyl-1H-benzo[d]imidazol-1-yl)-N′-(1-(o-tolyl)ethylidene)acetohydrazide* (**14b**)

According to the general procedure II, **10a** (0.23 g, 1 mmol) was reacted with 2-methylacetophenone (0.13 g, 1 mmol) (**12b**) in the presence of acetic acid in ethanol. Work-up followed by crystallization from DMSO gave **14b** as colorless crystals (0.27 g, 78%); mp: 238–240 °C; IR (KBr) *ṽ* 3438, 3073, 2966, 1630, 1525, 1448 cm^−1^; ^1^H-NMR (DMSO-*d*_6_, 400 MHz) of major conformer *δ*_H_ 1.27 (d, ^3^*J* = 6.8 Hz, 6H), 2.26 (s, 3H), 2.43 (s, 3H), 3.12 (sep, ^3^*J* = 6.8 Hz, 1H), 5.34 (s, 2H), 7.12–7.14 (m, 2H), 7.24–7.28 (m, 3H), 7.36–7.38 (m, 1H), 7.41–7.43 (m, 1H), 7.54–7.57 (m, 1H), 10.94 ppm (s, 1H); ^1^H-NMR (DMSO-*d*_6_, 400 MHz) of minor conformer *δ*_H_ 1.31 (d, ^3^*J* = 6.8 Hz, 6H), 2.30 (s, 3H), 2.41 (s, 3H), 3.23 (sep, ^3^*J* = 6.8 Hz, 1H), 5.16 (s, 2H), 7.12–7.14 (ov. m, 2H), 7.24–7.28 (ov. m, 3H), 7.36–7.38 (ov. m, 1H), 7.41–7.43 (ov. m, 1H), 7.54–7.57 (ov. m, 1H), 10.83 ppm (s, 1H); ^13^C-NMR (DMSO-*d*_6_, 100 MHz) of major conformer *δ*_C_ 18.00, 20.57, 21.60, 25.74, 44.33, 109.92, 118.39, 121.26, 121.59, 125.86, 128.36, 128.41, 130.85, 135.34, 135.83, 139.43, 142.14, 151.94, 160.40, 169.16 ppm; ^13^C-NMR (DMSO-*d*_6_, 100 MHz) of minor conformer *δ*_C_ 18.44, 20.10, 21.70, 25.04, 44.47, 109.92, 118.48, 121.43, 121.69, 125.72, 127.99, 128.41, 130.64, 135.13, 135.63, 139.57, 142.14, 156.07, 159.68, 163.99 ppm; Anal. Calcd. for C_21_H_24_N_4_O: C, 72.39; H, 6.94; N, 16.08. Found: C, 72.00; H, 6.71; N, 16.31. 

*(E)-N′-(1-(4-Bromophenyl)ethylidene)-2-(2-isopropyl-1H-benzo[d]imidazol-1-yl)acetohydrazide* (**14c**)

According to the general procedure II, **10a** (0.23 g, 1 mmol) was reacted with 4-bromoacetophenone (**12c**) (0.20 g, 1 mmol) in the presence of acetic acid in ethanol. Work-up followed by crystallization from methanol gave **14c** as white crystals (0.32 g, 78%); mp: 215–217 °C; IR (KBr): *ṽ* 3435, 2926, 2866, 1664, 1630, 1454 cm^−1^; ^1^H-NMR (DMSO-*d*_6_, 400 MHz) of major conformer: *δ*_H_ 1.28 (d, ^3^*J* = 6.8 Hz, 6H), 2.29 (s, 3H), 3.17 (d, ^3^*J* = 6.8 Hz, 1H), 5.51 (s, 2H), 7.12–7.16 (m, 2H), 7.41–7.43 (m, 1H), 7.55–7.57 (m, 1H), 7.62 (d, ^3^*J* = 8.4 Hz, 2H), 7.86 (d, ^3^*J* = 8.4 Hz, 2H), 11.08 ppm (s, 1H); ^1^H-NMR (DMSO-*d*_6_, 400 MHz) of minor conformer: *δ*_H_ 1.31 (d, ^3^*J* = 6.8 Hz, 6H), 2.35 (s, 3H), 3.22 (sep, ^3^*J* = 6.8 Hz, 1H), 5.17 (s, 2H), 7.12–7.16 (ov. m, 2H), 7.41–7.43 (ov. m, 1H), 7.55–7.57 (ov. m, 1H), 7.62 (ov. d, ^3^*J* = 8.4 Hz, 2H), 7.73 (d, ^3^*J* = 8.8 Hz, 2H), 10.93 ppm (s, 1H); ^13^C-NMR (DMSO-*d*_6_, 100 MHz) of major conformer *δ*_C_ 13.63, 21.67, 25.68, 44.44, 109.94, 118.37, 121.22, 121.53, 122.79, 128.49, 131.35, 135.83, 137.23, 142.18, 147.90, 160.48, 169.39 ppm; ^13^C-NMR (DMSO-*d*_6_, 100 MHz) of minor conformer *δ*_C_ 14.17, 21.67, 25.68, 44.51, 109.88, 118.47, 121.38, 121.64, 122.97, 128.60, 131.78, 135.61, 137.16, 142.12, 151.59, 160.34, 164.18 ppm; Anal. Calcd. for C_20_H_21_BrN_4_O: C, 58.12; H, 5.12; N, 13.56. Found: C, 58.43; H, 5.38; N, 13.35.

*(E)-N′-(1-(4-Bromo-3-nitrophenyl)ethylidene)-2-(2-isopropyl-1H-benzo[d]imidazol-1-yl)acetohydrazide* (**14d**)

According to the general procedure II, **10a** (0.23 g, 1 mmol) was reacted with 4´-bromo-3´-nitroacetophenone (0.24 g, 1 mmol) (**12d**) in the presence of acetic acid in ethanol. Work-up followed by crystallization from methanol gave **14d** as white crystals (0.32 g, 71%); mp: 231–233 °C; IR (KBr): *ṽ* 3430, 2968, 2929, 1685, 1626, 1531, 1454 cm^−1^; ^1^H-NMR (DMSO-*d*_6_, 400 MHz) of major conformer *δ*_H_ 1.29 (d, ^3^*J* = 6.8 Hz, 6H), 2.33 (s, 3H), 3.17 (sep, ^3^*J* = 6.8 Hz, 1H), 5.56 (s, 2H), 7.13–7.16 (m, 2H), 7.41–7.43 (m, 1H), 7.56–7.58 (m, 1H), 7.95 (d, ^3^*J* = 8.4 Hz, 1H), 8.09 (dd, ^3^*J* = 8.4 Hz, ^4^*J* = 1.8 Hz, 1H), 8.47 (d, ^3^*J* = 2.0 Hz, 1H), 11.24 ppm (s, 1H); ^1^H-NMR (DMSO-*d*_6_, 400 MHz) of minor conformer *δ*_H_ 1.32 (d, ^3^*J* = 6.8 Hz, 6H), 2.40 (s, 3H), 3.17 (ov. sep, ^3^*J* = 6.8 Hz, 1H), 5.20 (s, 2H), 7.13–7.16 (ov. m, 2H), 7.41–7.43 (ov. m, 1H), 7.56–7.58 (ov. m, 1H), 7.95 (ov. d, ^3^*J* = 8.4 Hz, 1H), 8.09 (dd, ^3^*J* = 8.4 Hz, ^4^*J* = 1.8 Hz, 1H), 8.32 (s, 1H), 11.10 ppm (s, 1H); ^13^C-NMR (DMSO-*d*_6_, 100 MHz) of major conformer δ_C_ 13.53, 21.69, 25.68, 44.57, 110.03, 113.20, 118.38, 121.33, 121.60, 122.57, 131.00, 134.54, 135.80, 138.96, 142.12, 146.05, 150.32, 160.54, 169.69 ppm; ^13^C-NMR (DMSO-*d*_6_, 100 MHz) of minor conformer *δ*_C_ 14.13, 21.58, 25.54, 44.27, 109.93, 113.20, 118.51, 121.48, 121.85, 122.92, 124.77, 132.64, 134.86, 135.36, 137.03, 142.05, 149.67, 160.06, 164.53 ppm; Anal. Calcd. for C_20_H_20_BrN_5_O_3_: C, 52.41; H, 4.40; N, 15.28. Found: C, 52.110; H, 4.76; N, 15.00.

*(E)-N′-(3-(Benzyloxy)benzylidene)-2-(2-isopropyl-1H-benzo[d]imidazol-1-yl)acetohydrazide* (**17a**)

According to the general procedure II, **10a** (0.23 g, 1 mmol) was reacted with **16a** (0.21 g, 1 mmol) in the presence of acetic acid in ethanol. Work-up followed by crystallization from ethanol gave **17a** as white crystals (0.32 g, 75%); mp 188–190 °C; ^1^H-NMR (DMSO-*d*_6_, 400 MHz) of major conformer *δ*_H_ 1.29 (d, ^3^*J* = 6.8 Hz, 6H), 3.18 (sep, ^3^*J* = 6.8 Hz, 1H), 5.16 (s, 2H), 5.48 (s, 2H), 7.10 (d, ^3^*J* = 7.6 Hz, 1H), 7.13–7.15 (m, 2H), 7.30–7.38 (m, 5H), 7.40–7.42 (m, 1H), 7.46–7.47 (m, 3H), 7.55–7.57 (m, 1H), 8.04 (s, 1H), 11.79 ppm (s, 1H); ^1^H-NMR (DMSO-*d*_6_, 400 MHz) of minor conformer *δ*_H_ 1.31 (d, ^3^*J* = 6.8 Hz, 6H), 3.23 (sep, ^3^*J* = 6.8 Hz, 1H), 5.03 (s, 2H), 5.13 (s, 2H), 7.10 (ov. d, ^3^*J* = 7.6 Hz, 1H), 7.15–7.17 (m, 2H), 7.30–7.38 (ov. m, 4H), 7.40–7.42 (ov. m, 2H), 7.46–7.47 (ov. m, 3H), 7.55–7.57 (ov. m, 1H), 8.23 (s, 1H, CH), 11.92 ppm (s, 1H); Anal. Calcd. for C_26_H_26_N_4_O_2_: C, 73.22; H, 6.14; N, 13.14. Found: C, 73.54; H, 6.43; N, 13.37.

*(E)-N′-(3-(Benzyloxy)benzylidene)-2-(2-(5-methylfuran-2-yl)-1H-benzo[d]imidazol-1-yl)acetohydrazide* (**17b**)

According to the general procedure II, **10b** (0.27 g, 1 mmol) was reacted with **16a** (0.21 g, 1 mmol) in the presence of acetic acid in ethanol. Work-up followed by crystallization from ethanol gave **17b** as white crystals (0.32 g, 69%); mp 180–182 °C; ^1^H-NMR (DMSO-*d*_6_, 400 MHz) of major conformer *δ*_H_ 2.22 (s, 3H), 5.15 (s, 2H), 5.74 (s, 2H), 6.32 (d, ^3^*J* = 2.8 Hz, 1H), 7.02 (d, ^3^*J* = 3.6 Hz, 1H), 7.09 (dd, ^3^*J* = 8.0 Hz, ^4^*J* = 2.4 Hz, 1H), 7.22–7.29 (m, 3H), 7.32–7.35 (m, 3H), 7.37–7.39 (m, 1H), 7.44–7.46 (ov. m, 3H), 7.62–7.64 (m, 2H), 8.07 (s, 1H), 11.80 ppm (s, 1H); ^1^H-NMR (DMSO-*d*_6_, 400 MHz) of minor conformer *δ*_H_ 2.34 (s, 3H), 5.13 (s, 2H), 5.29 (s, 2H), 6.34 (d, ^3^*J* = 3.2 Hz, 1H), 7.05 (d, ^3^*J* = 3.6 Hz, 1H), 7.09 (ov. dd, ^3^*J* = 8.0 Hz, ^4^*J* = 2.4 Hz, 1H), 7.22–7.29 (ov. m, 3H), 7.32–7.35 (ov. m, 3H), 7.37–7.39 (ov. m, 2H), 7.44–7.46 (ov. m, 2H), 7.62–7.64 (ov. m, 2H), 8.24 (s, 1H), 11.97 ppm (s, 1H); Anal. Calcd. for C_28_H_24_N_4_O_3_: C, 72.40; H, 5.21; N, 12.06. Found: C, 72.72; H, 5.44; N, 12.34.

*(E)-N′-(4-(Benzyloxy)benzylidene)-2-(2-isopropyl-1H-benzo[d]imidazol-1-yl)acetohydrazide* (**17c**)

According to the general procedure II, **10a** (0.23 g, 1 mmol) was reacted with **16b** (0.21 g, 1 mmol) in the presence of acetic acid in ethanol. Work-up followed by crystallization from ethanol gave **17c** as white crystals (0.32 g, 75%); mp 209–211 °C; ^1^H-NMR (DMSO-*d*_6_, 400 MHz) of major conformer *δ*_H_ 1.29 (d, ^3^*J* = 6.8 Hz, 6H), 3.18 (sep, ^3^*J* = 6.8 Hz, 1H), 5.16 (s, 2H), 5.47 (s, 2H), 7.10 (d, ^3^*J* = 8.4 Hz, 2H), 7.13–7.15 (m, 2H), 7.33–7.35 (m, 1H), 7.38–7.41 (m, 3H), 7.42–7.47 (m, 2H), 7.55–7.58 (m, 1H), 7.73 (d, ^3^*J* = 8.4 Hz, 2H), 8.02 (s, 1H), 11.66 ppm (s, 1H); ^1^H-NMR (DMSO-*d*_6_, 400 MHz) of minor conformer *δ*_H_ 1.32 (d, ^3^*J* = 7.2 Hz, 6H), 3.23 (sep, ^3^*J* = 6.8 Hz, 1H), 5.02 (s, 2H), 5.14 (s, 2H), 7.08 (d, ^3^*J* = 8.4 Hz, 2H), 7.15–7.17 (ov. m, 2H), 7.33–7.35 (ov. m, 1H), 7.38–7.41 (ov, m, 2H), 7.42–7.47 (m, 3H), 7.55–7.58 (ov. m, 1H), 7.65 (d, ^3^*J* = 8.8 Hz, 2H), 8.21 (s, 1H), 11.79 ppm (s, 1H); ^13^C-NMR (DMSO-*d*_6_, 100 MHz) of major conformer *δ*_C_ 21.60, 25.66, 43.90, 69.40, 110.00, 115.19, 118.28, 121.29, 121.60, 126.80, 127.80, 128.50, 128.74, 135.77, 136.77, 141.95, 144.20, 159.92, 160.40, 168.18 ppm; ^13^C-NMR (DMSO-*d*_6_, 100 MHz) of minor conformer *δ*_C_ 21.65, 25.66, 44.64, 69.40, 109.89, 114.45, 118.43, 121.44, 121.69, 126.72, 127.98, 128.50, 128.86, 135.53, 136.74, 141.99, 147.54, 160.09, 160.30, 163.24 ppm; Anal. Calcd. for C_26_H_26_N_4_O_2_: C, 73.22; H, 6.14; N, 13.14. Found: C, 73.58; H, 6.47; N, 12.97.

*(E)-N′-(4-(Benzyloxy)benzylidene)-2-(2-(5-methylfuran-2-yl)-1H-benzo[d]imidazol-1-yl)acetohydrazide* (**17d**)

According to the general procedure II, **10b** (0.27 g, 1 mmol) was reacted with **16b** (0.21 g, 1 mmol) in the presence of acetic acid in ethanol. Work-up followed by crystallization from ethanol afforded **17d** as white crystals (0.35 g, 76%); mp 201–203 °C; ^1^H-NMR (DMSO-*d*_6_, 400 MHz) of major conformer *δ*_H_ 2.24 (s, 3H), 5.16 (s, 2H), 5.73 (s, 2H), 6.31 (d, ^3^*J* = 2.8 Hz, 1H), 7.01 (d, ^3^*J* = 3.6 Hz, 1H), 7.09 (d, ^3^*J* = 8.8 Hz, 2H), 7.21–7.24 (m, 2H), 7.31–7.35 (m, 1H), 7.39 (t like, ^3^*J* = 7.6 Hz, 2H), 7.45–7.47 (m, 2H), 7.59–7.66 (m, 2H), 7.72 (d, ^3^*J* = 8.4 Hz, 2H), 8.05 (s, 1H), 11.67 ppm (s, 1H); ^1^H-NMR (DMSO-*d*_6_, 400 MHz) of minor conformer *δ*_H_ 2.34 (s, 3H), 5.14 (s, 2H), 5.26 (s, 2H), 6.34 (d, ^3^*J* = 2.8 Hz, 1H), 7.05 (d, ^3^*J* = 3.6 Hz, 1H), 7.07–7.10 (m, 2H), 7.21–7.24 (ov. m, 2H), 7.31–7.35 (ov. m, 1H), 7.39 (ov. t like, ^3^*J* = 7.6 Hz, 2H), 7.45–7.47 (ov. m, 2H), 7.59–7.66 (ov. m, 4H), 8.21 (s, 1H), 11.83 ppm (s, 1H); Anal. Calcd. for C_28_H_24_N_4_O_3_: C, 72.40; H, 5.21; N, 12.06. Found: C, 72.62; H, 5.54; N, 12.34.

*(E)-N′-(4-(Benzyloxy)-3-methoxybenzylidene)-2-(2-isopropyl-1H-benzo[d]imidazol-1-yl)acetohydrazide* (**17e**) 

According to the general procedure II, **10a** (0.23 g, 1 mmol) was reacted with **16c** (0.24 g, 1 mmol) in the presence of acetic acid in ethanol. Work-up followed by crystallization from ethanol gave **17e** as white crystals (0.32 g, 70%); mp: 180–182 °C; ^1^H-NMR (DMSO-*d*_6_, 400 MHz) of major conformer *δ*_H_ 1.30 (d, ^3^*J* = 6.8 Hz, 6H), 3.16–3.25 (m, 1H), 3.83 (s, 3H), 5.14 (s, 2H), 5.49 (s, 2H), 7.10–7.21 (m, 3H), 7.25 (d, ^3^*J* = 8.0 Hz, 1H), 7.32–7.46 (m, 7H), 7.55–7.58 (m, 1H), 8.00 (s, 1H), 11.69 ppm (s, 1H); ^1^H-NMR (DMSO-*d*_6_, 400 MHz) of minor conformer *δ*_H_ 1.32 (d, ^3^*J* = 7.2 Hz, 6H), 3.16–3.25 (ov. m, 1H), 3.78 (s, 3H), 5.02 (s, 2H), 5.12 (s, 2H), 7.10–7.21 (ov. m, 4H), 7.32–7.46 (ov. m, 7H), 7.55–7.58 (m, 1H), 8.19 (s, 1H), 11.79 ppm (s, 1H); ^13^C-NMR (DMSO-*d*_6_, 100 MHz) of major conformer *δ*_C_ 21.64, 25.70, 43.89, 55.70, 69.94, 109.28, 109.99, 113.25, 118.38, 121.26, 121.35, 121.56, 127.10, 127.93, 128.50, 135.83, 136.82, 142.15, 144.42, 149.43, 149.70, 160.45, 168.29 ppm; ^13^C-NMR (DMSO-*d*_6_, 100 MHz) of minor conformer *δ*_C_ 21.67, 25.70, 44.65, 55.56, 69.94, 108.76, 109.87, 113.10, 118.50, 121.44, 121.69, 121.91, 126.99, 128.01, 128.50, 135.58, 136.78, 142.15, 144.42, 147.87, 149.91, 160.35, 163.33 ppm; Anal. Calcd. for C_27_H_28_N_4_O_3_: C, 71.03; H, 6.18; N, 12.27. Found: C, 71.36; H, 6.42; N, 11.98.

*(E)-N′-(4-(Benzyloxy)-3-methoxybenzylidene)-2-(2-(5-methylfuran-2-yl)-1H-benzo[d]imidazol-1-yl)acetohydrazide* (**17f**)

According to the general procedure II, **10b** (0.27 g, 1 mmol) was reacted with **16c** (0.24 g, 1 mmol) in the presence of acetic acid in ethanol. Work-up followed by crystallization from ethanol gave **17e** as white crystals (0.37 g, 75%); mp 139–141 °C; ^1^H-NMR (DMSO-*d*_6_, 400 MHz) of major conformer *δ*_H_ 2.25 (s, 3H), 3.82 (s, 3H), 5.14 (s, 2H), 5.75 (s, 2H), 6.31 (d, ^3^*J* = 2.4 Hz, 1H), 7.01 (d, ^3^*J* = 3.2 Hz, 1H), 7.11 (d, ^3^*J* = 8.4 Hz, 1H), 7.16–7.26 (m, 3H), 7.32–7.46 (m, 6H), 7.62–7.64 (m, 2H), 8.03 (s, 1H), 11.70 ppm (s, 1H); ^1^H-NMR (DMSO-*d*_6_, 400 MHz) of minor conformer *δ*_H_ 2.35 (s, 3H), 3.78 (s, 3H), 5.16 (s, 2H), 5.27 (s, 2H), 7.06 (d, ^3^*J* = 3.2 Hz, 1H), 7.11 (ov. d, ^3^*J* = 8.4 Hz, 1H), 7.16–7.26 (ov. m, 3H), 7.32–7.46 (ov. m, 7H), 7.62–7.64 (ov. m, 2H), 8.19 (s, 1H), 11.84 ppm (s, 1H); Anal. Calcd. for C_29_H_26_N_4_O_4_: C, 70.43; H, 5.30; N, 11.33. Found: C, 70.72; H, 5.04; N, 11.54.

*Methyl-(E)-2-(4-((2-(2-(2-isopropyl-1H-benzo[d]imidazol-1-yl)acetyl)hydrazono)methyl)-2-methoxyphenoxy)acetate* (**19a**)

According to the general procedure II, **10a** (0.23 g, 1 mmol) was reacted with **18a** (0.22 g, 1 mmol) in the presence of acetic acid in ethanol. Work-up followed by crystallization from ethanol gave **19a** as colorless crystals (0.28 g, 65%); mp 175–177 °C; ^1^H-NMR (DMSO-*d*_6_, 400 MHz) of major conformer *δ*_H_ 1.30 (d, ^3^*J* = 6.8 Hz, 6H), 3.17–3.28 (m, 1H), 3.70 (s, 3H), 3.84 (s, 3H), 4.85 (s, 2H), 5.49 (s, 2H), 6.94 (d, ^3^*J* = 8.4 Hz, 1H), 7.13–7.15 (m, 2H), 7.23 (d, ^3^*J* = 7.6 Hz, 1H), 7.41–7.43 (m, 1H), 7.45 (s, 1H), 7.55–7.57 (m, 1H), 8.01 (s, 1H), 11.68 ppm (s, 1H); ^1^H-NMR (DMSO-*d*_6_, 400 MHz) of minor conformer *δ*_H_ 1.31 (d, ^3^*J* = 8.4 Hz, 6H), 3.17–3.28 (ov. m, 1H), 3.69 (s, 3H), 3.80 (s, 3H), 4.84 (s, 2H), 5.02 (s, 2H), 6.94 (ov. d, ^3^*J* = 8.4 Hz, 1H), 7.16–7.19 (m, 2H), 7.23 (d, ^3^*J* = 8.4 Hz, 1H), 7.32 (s, 1H), 7.41–7.43 (ov. m, 1H), 7.55–7.57 (ov. m, 1H), 8.19 (s, 1H), 11.82 ppm (s, 1H); ^13^C-NMR (DMSO-*d*_6_, 100 MHz) of major conformer *δ*_C_ 21.64, 25.69, 43.87, 51.90, 55.73, 65.10, 109.53, 109.99, 113.11, 118.38, 121.12, 121.25, 121.55, 127.72, 135.83, 142.15, 144.22, 148.85, 149.19, 160.44, 168.34, 169.08 ppm; ^13^C-NMR (DMSO-*d*_6_, 100 MHz) of minor conformer *δ*_C_ 21.67, 25.69, 44.63, 51.90, 55.61, 65.06, 109.09, 109.86, 113.03, 118.49, 121.42, 121.60, 121.67, 127.62, 135.58, 142.12, 144.22, 147.69, 149.03, 160.34, 163.36, 169.05 ppm; Anal. Calcd. for C_23_H_26_N_4_O_5_: C, 63.00; H, 5.98; N, 12.78. Found: C, 63.32; H, 5.64; N, 12.54.

*Methyl-(E)-2-(2-methoxy-4-((2-(2-(2-(5-methylfuran-2-yl)-1H-benzo[d]imidazol-1-yl)acetyl)hydrazono)methyl)phenoxy)acetate* (**19b**)

According to the general procedure II, **10b** (0.27 g, 1 mmol) was reacted with **18a** (0.22 g, 1 mmol) in the presence of acetic acid in ethanol. Work-up followed by crystallization from ethanol gave **19b** as colorless crystals (0.48 g, 71%); mp 150–152 °C; ^1^H-NMR (DMSO-*d*_6_, 400 MHz) of major conformer *δ*_H_ 2.25 (s, 3H), 3.70 (s, 3H), 3.83 (s, 3H), 4.84 (s, 2H), 5.75 (s, 2H), 6.32 (d, ^3^*J* = 2.8 Hz, 1H), 6.94 (d, ^3^*J* = 8.4 Hz, 1H), 7.01 (d, ^3^*J* = 3.2 Hz, 1H), 7.22–7.25 (m, 3H), 7.43 (s, 1H), 7.62–7.64 (m, 2H), 8.03 (s, 1H), 11.72 ppm (s, 1H); ^1^H-NMR (DMSO-*d*_6_, 400 MHz) of minor conformer *δ*_H_ 2.34 (s, 3H), 3.70 (ov. s, 3H), 3.80 (s, 3H), 4.84 (ov. s, 2H), 5.27 (s, 2H), 6.34 (d, ^3^*J* = 2.4 Hz, 1H), 6.93 (d, ^3^*J* = 8.0 Hz, 1H), 7.05 (d, ^3^*J* = 2.8 Hz, 1H), 7.18 (d, ^3^*J* = 8.4 Hz, 1H), 7.22–7.25 (ov. m, 2H), 7.32 (s, 1H), 7.59–7.64 (ov. m, 2H), 8.20 (s, 1H), 11.85 ppm (s, 1H); ^13^C-NMR (DMSO-*d*_6_, 100 MHz) of major conformer *δ*_C_ 13.32, 45.76, 51.94, 55.77, 65.12, 108.48, 109.55, 110.44, 113.20, 113.25, 118.71, 120.99, 122.27, 122.53, 127.79, 136.54, 142.49, 143.66, 144.12, 144.33, 148.86, 149.24, 153.79, 168.66, 169.13 ppm; ^13^C-NMR (DMSO-*d*_6_, 100 MHz) of minor conformer *δ*_C_ 13.46, 48.68, 51.94, 55.67, 65.14, 108.52, 109.14, 110.34, 113.08, 113.51, 118.80, 121.56, 122.41, 127.72, 136.37, 142.46, 143.40, 144.12, 144.29, 147.25, 149.01, 149.22, 154.05, 163.71, 169.10 ppm; Anal. Calcd. for C_25_H_24_N_4_O_6_: C, 63.02; H, 5.08; N, 11.76. Found: C, 63.22; H, 5.32; N, 12.01.

*Ethyl-(E)-2-(4-((2-(2-(2-isopropyl-1H-benzo[d]imidazol-1-yl)acetyl)hydrazono)methyl)-2-methoxyphenoxy)acetate* (**19c**) 

According to the general procedure II, **10a** (0.23 g, 1 mmol) was reacted with **18b** (0.24 g, 1 mmol) in the presence of acetic acid in ethanol. Work-up followed by crystallization from ethanol gave **19c** as white crystals (0.36 g, 80%); mp: 93–95 °C; ^1^H-NMR (DMSO-*d*_6_, 400 MHz) of major conformer *δ*_H_ 1.21 (t, ^3^*J* = 7.2 Hz, 3H), 1.30 (d, ^3^*J* = 7.2 Hz, 6H), 3.19–3.25 (m, 1H), 3.84 (s, 3H), 4.17 (q, ^3^*J* = 7.2 Hz, 2H), 4.83 (s, 2H), 5.49 (s, 2H), 6.93 (d, ^3^*J* = 8.4 Hz, 1H), 7.14 (dd, ^3^*J* = 6.0 Hz, ^4^*J* = 2.8 Hz, 2H), 7.23 (dd, ^3^*J* = 8.4 Hz, ^4^*J* = 2.8 Hz, 1H), 7.42 (dd, ^3^*J* = 6.0 Hz, ^4^*J* = 3.2 Hz, 1H), 7.45 (d, ^4^*J* = 1.2 Hz, 1H), 7.56 (dd, ^3^*J* = 6.0 Hz, ^4^*J* = 3.2 Hz, 1H), 8.00 ppm (s, 1H); ^1^H-NMR (DMSO-*d*_6_, 400 MHz) of minor conformer *δ*_H_ 1.20 (ov. t, ^3^*J* = 6.8 Hz, 3H), 1.31 (d, ^3^*J* = 7.2 Hz, 6H), 3.19–3.25 (ov. m, 1H), 3.80 (s, 3H), 4.17 (ov. q, ^3^*J* = 7.2 Hz, 2H), 4.81 (s, 2H), 5.03 (s, 2H), 6.92 (d, ^3^*J* = 8.4 Hz, 1H), 7.17 (dd, ^3^*J* = 8.0 Hz, ^4^*J* = 2.8 Hz, 2H), 7.32–7.34 (m, 1H), 7.42 (ov. dd, ^3^*J* = 6.0 Hz, ^4^*J* = 3.2 Hz, 1H), 7.51 (d, ^4^*J* = 1.2 Hz, 1H), 7.56 (ov. dd, ^3^*J* = 6.0 Hz, ^4^*J* = 3.2 Hz, 1H), 8.20 ppm (s, 1H); ^13^C-NMR (DMSO-*d*_6_, 100 MHz) of major conformer *δ*_C_ 14.10, 21.65, 25.70, 43.89, 55.76, 60.78, 65.22, 109.60, 110.00, 113.17, 118.38, 121.12, 121.27, 121.58, 127.71, 135.83, 142.15, 144.26, 148.90, 149.22, 160.46, 168.35, 168.60 ppm; ^13^C-NMR (DMSO-*d*_6_, 100 MHz) of minor conformer *δ*_C_ 14.10, 21.68, 25.70, 44.63, 55.64, 60.81, 65.18, 109.13, 109.89, 113.09, 118.49, 121.44, 121.69, 127.64, 135.58, 142.12, 147.71, 149.07, 149.16, 149.84, 160.37, 163.40, 168.51, 168.57 ppm; Anal. Calcd. for C_24_H_28_N_4_O_5_: C, 63.70; H, 6.24; N, 12.38. Found: C, 63.42; H, 5.89; N, 12.14.

*Ethyl-(E)-2-(2-methoxy-4-((2-(2-(2-(5-methylfuran-2-yl)-1H-benzo[d]imidazol-1-yl)acetyl)hydrazono)methyl)phenoxy)acetate* (**19d**)

According to the general procedure II, **10b** (0.27 g, 1 mmol) was reacted with **18b** (0.24 g, 1 mmol) in the presence of acetic acid in ethanol. Work-up followed by crystallization from ethanol gave **19d** as colorless crystals (0.43 g, 88%); mp 136–138 °C; ^1^H-NMR (DMSO-*d*_6_, 400 MHz) of major conformer *δ*_H_ 1.20 (t, ^3^*J* = 6.8 Hz, 3H), 2.25 (s, 3H), 3.83 (s, 3H), 4.16 (q, ^3^*J* = 7.2 Hz, 2H), 4.82 (s, 2H), 5.76 (s, 2H), 6.31 (d, ^3^*J* = 3.2 Hz, 1H), 6.93 (d, ^3^*J* = 8.4 Hz, 1H), 7.01 (d, ^3^*J* = 3.2 Hz, 1H), 7.21–7.25 (m, 3H), 7.43 (d, ^4^*J* = 1.2 Hz, 1H), 7.62–7.64 (m, 2H), 8.03 (s, 1H), 11.88 ppm (br., 1H); ^1^H-NMR (DMSO-*d*_6_, 400 MHz) of minor conformer *δ*_H_ 1.20 (ov. t, ^3^*J* = 6.8 Hz, 3H), 2.34 (s, 3H), 3.79 (s, 3H), 4.16 (ov. q, ^3^*J* = 7.2 Hz, 2H), 4.81 (s, 2H), 5.27 (s, 2H), 6.34 (d, ^3^*J* = 2.8 Hz, 1H), 6.92 (ov. d, ^3^*J* = 8.4 Hz, 1H), 7.05 (d, ^3^*J* = 3.2 Hz, 1H), 7.21–7.25 (ov. m, 3H), 7.32 (d, ^4^*J* = 1.2 Hz, 1H), 7.62–7.64 (ov. m, 2H), 8.19 (s, 1H), 11.88 ppm (br., 1H); ^13^C-NMR (DMSO-*d*_6_, 100 MHz) of major conformer *δ*_C_ 13.32, 14.11, 45.77, 55.79, 60.81, 65.81, 108.49, 109.61, 110.45, 113.26, 118.72, 120.97, 122.27, 122.53, 127.78, 136.54, 142.50, 143.67, 144.14, 144.34, 148.90, 149.26, 153.79, 168.64, 168.66 ppm; ^13^C-NMR (DMSO-*d*_6_, 100 MHz) of minor conformer *δ*_C_ 13.46, 14.11, 46.36, 55.66, 60.81, 65.23, 108.52, 109.16, 110.35, 113.51, 118.80, 121.55, 122.41, 122.63, 127.74, 136.40, 142.47, 143.41, 143.74, 144.31, 147.26, 149.04, 154.05, 163.73, 168.61 ppm; Anal. Calcd. for C_26_H_26_N_4_O_6_: C, 63.66; H, 5.34; N, 11.42. Found: C, 63.42; H, 5.59; N, 11.64.

*(E)-N′-(4-(2-(4-Formyl-2-methoxyphenoxy)ethoxy)-3-methoxybenzylidene)-2-(2-isopropyl-1H-benzo[d]imidazol-1-yl)acetohydrazide* (**22a**)

According to the general procedure II, **10a** (0.23 g, 1 mmol) was reacted with **21** (0.33 g, 1 mmol) in the presence of acetic acid in ethanol. Work-up followed by crystallization from DMSO gave **22a** as a white powder (0.26 g, 48%); mp 218–220 °C; ^1^H-NMR (DMSO-*d*_6_, 400 MHz) of major conformer *δ*_H_ 1.29 (d, ^3^*J* = 6.8 Hz, 6H), 3.20 (sep, ^3^*J* = 6.8 Hz, 1H), 3.82 (s, 3H), 3.83 (s, 3H), 4.38 (s, 2H), 4.47 (s, 2H), 5.49 (s, 2H), 7.12–7.16 (m, 3H), 7.26–7.28 (m, 2H), 7.41–7.45 (m, 3H), 7.55–7.57 (m, 2H), 8.01 (s, 1H), 9.85 (s, 1H), 11.69 ppm (s, 1H); ^1^H-NMR (DMSO-*d*_6_, 400 MHz) of minor conformer *δ*_H_ 1.31 (d, ^3^*J* = 6.8 Hz, 6H), 3.20 (ov. sep, ^3^*J* = 6.8 Hz, 1H), 3.77 (s, 3H), 3.78 (s, 3H), 4.37 (s, 2H), 4.47 (s, 2H), 5.01 (s, 2H), 7.12–7.16 (m, 3H), 7.26–7.28 (ov. m, 2H), 7.41–7.45 (ov. m, 3H), 7.55–7.57 (ov. m, 2H), 8.20 (s, 1H), 9.85 (ov. s, 1H), 11.80 ppm (s, 1H); ^13^C-NMR (DMSO-*d*_6_, 100 MHz) of major conformer *δ*_C_ 21.68, 25.74, 43.95, 55.62, 55.67, 67.30, 109.24, 109.94, 112.52, 112.87, 118.41, 121.35, 121.53, 121.56, 121.65, 126.04, 127.21, 130.04, 135.84, 142.13, 144.49, 149.20, 149.27, 149.77, 153.23, 160.53, 168.35, 191.62 ppm; ^13^C-NMR (DMSO-*d*_6_, 100 MHz) of minor conformer *δ*_C_ 21.71, 25.74, 44.66, 55.55, 55.62, 67.19, 108.86, 110.06, 112.49, 112.81, 118.53, 121.76, 121.78, 122.03, 126.04, 127.12, 130.00, 135.59, 142.11, 147.91, 149.20, 149.27, 149.95, 153.30, 160.53, 163.41, 191.62 ppm; Anal. Calcd. for C_30_H_32_N_4_O_6_: C, 66.16; H, 5.92; N, 10.29. Found: C, 66.50; H, 6.22; N, 9.93.

*(E)-N′-(4-(2-(4-Formyl-2-methoxyphenoxy)ethoxy)-3-methoxybenzylidene)-2-(2-(5-methylfuran-2-yl)-1H-benzo[d]imidazol-1-yl)acetohydrazide* (**22b**)

According to the general procedure II, **10b** (0.27 g, 1 mmol) was reacted with **21** (0.33 g, 1 mmol) in the presence of acetic acid in ethanol. Work-up followed by crystallization from DMSO gave **22b** as white crystals (0.48 g, 82%); mp 203–205 °C; ^1^H-NMR (DMSO-*d*_6_, 400 MHz) of major conformer *δ*_H_ 2.25 (s, 3H), 3.81 (s, 3H), 3.82 (s, 3H), 4.37 (s, 2H), 4.47 (s, 2H), 5.76 (s, 2H), 6.32 (d, ^3^*J* = 2.8 Hz, 1H), 7.02 (d, ^3^*J* = 3.2 Hz, 1H), 7.12 (d, ^3^*J* = 8.0 Hz, 1H), 7.22–7.31 (m, 4H), 7.41–7.43 (m, 2H), 7.56 (dd, ^3^*J* = 8.0 Hz, ^4^*J* = 1.6 Hz 1H), 7.62–7.65 (m, 2H), 8.04 (s, 1H), 9.85 (s, 1H), 11.71 ppm (s, 1H); ^1^H-NMR (DMSO-*d*_6_, 400 MHz) of minor conformer *δ*_H_ 2.35 (s, 3H), 3.80 (ov. s, 3H), 3.83 (s, 3H), 4.37 (s, 2H), 4.47 (s, 2H), 5.27 (s, 2H), 6.34 (d, ^3^*J* = 2.8 Hz, 1H), 7.06 (d, ^3^*J* = 3.2 Hz, 1H), 7.12 (ov. d, ^3^*J* = 8.0 Hz, 1H), 7.22–7.31 (ov. m, 4H), 7.41–7.43 (ov. m, 2H), 7.56 (ov. dd, ^3^*J* = 8.0 Hz, ^4^*J* = 1.6 Hz, 1H), 7.62–7.65 (ov. m, 2H), 8.20 (s, 1H), 9.85 (ov. s, 1H), 11.85 ppm (s, 1H); Anal. Calcd. for C_32_H_30_N_4_O_7_: C, 65.97; H, 5.19; N, 9.62. Found: C, 65.63; H, 5.45; N, 9.42.

General procedure III for the synthesis of **24a–c**

A solution of **10a** or **10b** (1 mmol), *p*-toluenesulfonyl isocyanate (**23a**) or *p*-methoxyphenyl isothiocyanate (**23b**) (1 mmol) in ethanol (10 mL) was refluxed for 4 h. The reaction mixture was filtered, dried and further purified by recrystallization from methanol to give the corresponding analytically pure compound.

*2-(2-(2-Isopropyl-1H-benzo[d]imidazol-1-yl)acetyl)-N-tosylhydrazine-1-carboxamide* (**24a**)

According to the general procedure III, **10a** (0.23 g, 1 mmol) was reacted with *p*-toluenesulfonyl isocyanate (**23a**) (0.20 g, 1 mmol) in ethanol to give **24a** as white powder (0.28 g, 66%); mp 197–199 °C; ^1^H-NMR (DMSO-*d*_6_, 400 MHz) *δ*_H_ 1.29 (br., 6H), 2.36 (s, 3H), 3.13-3.25 (m, 1H), 4.86 (d, ^3^*J* = 25.6 Hz, 2H), 7.14–7.15 (m, 2H), 7.25–7.26 (m, 1H), 7.35–7.38 (m, 2H), 7.54–7.55 (m, 1H), 7.70 (d, ^3^*J* = 7.0 Hz, 1H), 7.77 (d, ^3^*J* = 7.0 Hz, 1H), 8.60 (br., 1H), 9.54 (br., 1H), 10.25 ppm (br., 1H); Anal. Calcd. for C_20_H_23_N_5_O_4_S: C, 55.93; H, 5.40; N, 16.31. Found: C, 55.65; H, 5.14; N, 16.62.

*2-(2-(2-Isopropyl-1H-benzo[d]imidazol-1-yl)acetyl)-N-(4-methoxyphenyl)hydrazine-1-carbothioamide* (**24b**).

According to the general procedure III, **10a** (0.23 g, 1 mmol) was reacted with *p*-methoxyphenyl isothiocyanate (**23b**) (0.23 g, 1 mmol) in ethanol to give **24b** as a white powder (0.30 g, 76%); mp 232–234°C; ^1^H-NMR (DMSO-*d*_6_, 400 MHz) *δ*_H_ 1.31 (d, ^3^*J* = 6.8 Hz, 6H), 3.23 (sep, ^3^*J* = 6.8 Hz, 1H), 3.75 (s, 3H), 5.00 (s, 2H), 6.92 (d, ^3^*J* = 8.8 Hz, 2H), 7.13–7.19 (m, 2H), 7.26 (d, ^3^*J* = 8.8 Hz, 2H), 7.42 (d, ^3^*J* = 6.8 Hz, 1H), 7.56 (dd, ^3^*J* = 8.4 Hz, ^4^*J* = 1.6 Hz 1H), 9.62 (s, 2H), 10.45 ppm (s, 1H); Anal. Calcd. for C_20_H_23_N_5_O_2_S: C, 60.43; H, 5.83; N, 17.62. Found: C, 60.73; H, 5.53; N, 17.42.

*N*-(4-Methoxyphenyl)-2-(2-(2-(5-methylfuran-2-yl)-1*H*-benzo[*d*]imidazol-1-yl)acetyl)hydrazine-1-carbothioamide (**24c**).

According to the general procedure III, **10b** (0.27 g, 1 mmol) was reacted with *p*-methoxyphenyl isothiocyante (**23b**) (0.23 g, 1 mmol) in ethanol to give **24c** as a white powder (0.35g, 81%); mp: 189–191 °C; ^1^H-NMR (DMSO-*d*_6_, 400 MHz) *δ*_H_ 2.41 (s, 3H), 3.75 (s, 3H), 5.27 (s, 2H), 6.34 (br., 1H), 6.92 (d, ^3^*J* = 8.4 Hz, 2H), 7.06 (br., 1H), 7.23–7.27 (m, 4H), 7.53 (d, ^3^*J* = 7.6 Hz, 1H), 7.63 (d, ^3^*J* = 7.0 Hz, 1H), 9.59 (br., 1H), 9.73 (br., 1H), 10.40 ppm (br., 1H); ^13^C-NMR (DMSO-*d*_6_, 400 MHz) *δ*_C_ 13.70, 45.97, 55.54, 108.64, 110.56, 113.79, 114.10, 118.97, 122.79, 122.92, 131.94, 136.33, 142.49, 143.15, 144.58, 154.86, 157.32 ppm; Anal. Calcd. for C_22_H_21_N_5_O_3_S: C, 60.68; H, 4.86; N, 16.08. Found: C, 60.34; H, 4.98; N, 16.43.

*X-ray Crystallography*

Crystals were grown following the protocol developed by Hope by dissolving the compound in DMSO and left to slowly crystalize [53,54]. Single crystal X-ray diffraction data for all compounds were collected on a Bruker APEX 2 DUO CCD diffractometer by using graphite-monochromated MoK*_α_* (*λ* = 0.71073 Å) radiation. Crystals were mounted on a MiTeGen MicroMount and collected at 100(2) K by using an Oxford Cryosystems Cobra low-temperature device. Data were collected by using omega and phi scans and were corrected for Lorentz and polarization effects by using the APEX software suite [55,56,57]. Using Olex2, the structure was solved with the XT structure solution program, using the intrinsic phasing solution method and refined against │F2│ with XL using least-squares minimization [58,59]. Hydrogen atoms were generally placed in geometrically calculated positions and refined using a riding model unless otherwise stated. All images were rendered using Olex2 [58]. Details of data refinements can be found in Tables S2 and S10 in the Supporting Information. Refinement details for **14a**: The hydrogen attached to N3 (H3) was allowed to freely refine. The distance of this bond was fixed using the DFIX restraint at 0.88 (0.01) Å. Refinement details for **13c**: The hydrogen attached to N3 (H3) was allowed to freely refine. The distance of this bond was fixed using the DFIX restraint at 0.88 (0.01) Å. The structure contained two solvent DMSO molecules, one of which was disordered over two positions. The disordered DMSO (Part one: S2s; Part two: S3s) were modelled over two positions in a 75:25% occupancy without using any restraint or constraints.

CCDC 1973400 and 1973401 contain the supplementary crystallographic data for this paper. These data can be obtained free of charge from the Cambridge Crystallographic Data Centre via www.ccdc.cam.ac.uk/data_request/cif.

### 3.2. Biological Evaluation:

#### 3.2.1. In Vitro Anti-Proliferative Activity

The anticancer activity of the synthesized compounds was measured in vitro using the Sulfo-Rhodamine-B (SRB) assay according to Skehan et al. [52]. Briefly, HepG2 or HSF cells (obtained from Nawah Scientific) were inoculated in 96-well microtiter plates (10^4^ cells/ well) for 24 h before treatment with the tested compounds to allow attachment of cells to the walls of the plates. Test compounds were dissolved in DMSO immediately before use and diluted to the appropriate volume just before addition to the cell culture. Different concentrations of tested compounds and sorafenib were added to the cells. Three wells were prepared for each individual dose. Monolayer cells were incubated with the compounds for 48 h at 37 °C and in an atmosphere of 5% CO_2_. After 48 h, cells were fixed, washed and stained for 30 min with 0.4% (*w*/*v*) SRB dissolved in 1% acetic acid. Unbound dye was removed by four washes with 1% acetic acid and attached stain was recovered with Tris-EDTA buffer. Color intensity was measured in an ELISA reader. The relation between surviving fraction and drug concentration was plotted to get the survival curve. The concentration required for 50% inhibition of cell viability (IC_50_) was calculated, and the results were compared to the effect of the reference drug sorafenib.

#### 3.2.2. In Vitro Cell-Based VEGFR-2 Inhibitory Activity

The target 1,2-disubstituted benzo[*d*]imidazoles were screened for their VEGFR-2 inhibitory activity in the HepG2 cell line. The cells in the culture medium were treated with 20 µL of 1/10 of the determined IC_50_ values of the compounds or the standard reference drug, sorafenib, dissolved in DMSO, then incubated for 48 h at 37 °C in a humidified 5% CO_2_ atmosphere. The cells were harvested, and homogenates were prepared in saline using a tight pestle homogenizer until complete cell disruption. The kit uses a double-antibody sandwich enzyme-linked immunosorbent assay (ELISA) to assay the concentration of human VEGFR-2 in the samples. Antibody specific for VEGFR-2 was precoated onto 96-well plates. Standard and test samples were added to the wells; then, a horseradish peroxidase (HRP)-conjugated antibody specific VEGFR-2 was added and incubated. Unbound conjugates were washed away. The TMB substrate was used to visualize the HRP enzymatic reaction. TMB was catalyzed by HRP to produce a blue color product that changed into yellow after adding the stop solution. The density of yellow was proportional to the human VEGFR-2 amount. The optical density was determined spectrophotometrically at a wavelength of 450 nm. The amount of VEGFR-2 in the samples was calculated (ng/mL) as duplicate determinations, and the data were compared with sorafenib (**I**) as the standard VEGFR-2 inhibitor.

#### 3.2.3. Biochemical Kinase Inhibition Assay

This assay was carried out using the VEGFR-2 kinase enzyme system or FGFR-1 kinase enzyme system and Kinase-Glo Plus luminescence kinase (Promega) kits according to the manufacturers´ protocols. This assay evaluated the activity of kinase by measuring the amount of ATP remaining in solution after a kinase reaction. The luminescent signal from the assay was related to the quantity of ATP available, which was inversely proportional to the amount of kinase activity. A stock solution of the synthesized compounds and sorafenib (**I**) in DMSO, e.g., 10% was initially prepared. Subsequently, serial dilutions were carried out, and 5 µL of the dilution was added to a 50 µL reaction. The enzymatic reactions were performed at 30 °C for 45 minutes. The 50 µL reaction mixture contained 40 mM Tris, pH 7.4, 10 mM MgCl_2_, 0.1 mg/mL BSA, 1 mM DTT, 0.2 mg/ml Poly (Glu, Tyr) substrate, 10 μM ATP and protein. After the enzymatic reaction, 50 µL of Kinase-Glo Plus Luminescence kinase assay solution was added to each reaction, and the plates were incubated for 15 minutes at room temperature. The luminescence signal was measured using a Tecan–spark multimode microplate reader. The difference between luminescence intensities in the absence of Kinase (Lu_t_) and in the presence of Kinase (Lu_c_) was defined as 100% activity (Lu_t_ − Lu_c_). Using a luminescence signal (Lu) in the presence of the compound, % activity was calculated as: % activity = {(Lu_t_ − Lu)/(Lu_t_ − Lu_c_)}×100%, where Lu = the luminescence intensity in the presence of the compound. The luminescence data were analyzed using Graphpad Prism, and IC_50_ values were calculated as the average value from two independent experiments [18].

#### 3.2.4. Analysis of Cell Cycle Distribution 

After treatment with test compounds for 48 h, cells (10^5^ cells) were collected by trypsinization and washed twice with ice-cold PBS (pH 7.4). Cells were resuspended in two milliliters of 60% ice-cold ethanol and incubated at 4 °C for 1 h for fixation. Fixed cells were washed twice again with PBS (pH 7.4) and resuspended in 1 mL of PBS containing 50 µg/mL RNAase A and 10 µg/mL propidium iodide (PI). After 20 min of incubation in dark at 37 °C, cells were analyzed for DNA contents using flow cytometry analysis using an FL2 (λex/em 535/617 nm) signal detector (ACEA Novocyte™ flowcytometer, ACEA Biosciences Inc., San Diego, CA, USA). For each sample, 12,000 events were acquired. The cell cycle distribution was calculated using ACEA NovoExpress™ software (ACEA Biosciences Inc., San Diego, CA, USA) [60]. 

#### 3.2.5. Apoptosis Assay

Apoptosis and necrosis cell populations were determined using an Annexin V-FITC apoptosis detection kit (Abcam Inc., Cambridge Science Park, Cambridge, UK) coupled with 2 fluorescent channels flowcytometry. After treatment with test compounds for 48 h, cells (10^5^ cells) were collected by trypsinization and washed twice with ice-cold PBS (pH 7.4). Then, cells were incubated in the dark with 0.5 ml of Annexin V-FITC/PI solution for 30 min in the dark at room temperature, according to the manufacturer’s protocol. After staining, cells were injected via ACEA Novocyte™ flowcytometer (ACEA Biosciences Inc., San Diego, CA, USA) and analyzed for FITC and PI fluorescent signals using FL1 and FL2 signal detectors, respectively (λex/em 488/530 nm for FITC and λex/em 535/617 nm for PI). For each sample, 12,000 events were acquired, and positive FITC and/or PI cells were quantified by quadrant analysis and calculated using ACEA NovoExpress™ software (ACEA Biosciences Inc., San Diego, CA, USA) [60].

### 3.3. Molecular Docking Study

The molecular docking simulation studies were carried out using Molecular Operating Environment (MOE, 2010.10) software. All minimizations were performed with MOE until an RMSD gradient of 0.10 kcal∙mol^−1^Å^−1^ with a MMFF94× forcefield and the partial charges were automatically calculated. The X-ray crystallographic structure of VEGFR-2 in its inactive “DFG-out” conformation co-crystallized with the aryl urea derivative sorafenib as the inhibitor (PDB ID: 4ASD) [26] and was downloaded from the protein databank [http://www.rcsb.org/]. Water molecules were first removed, then the VEGFR-2 protein structure was prepared for the molecular docking study using the *Protonate 3D* protocol in MOE with the default options. The co-crystalized ligand (sorafenib) was used to locate the active site for docking. The Triangle Matcher placement method and London dG scoring function were used for docking. The docking protocol was initially validated by self-docking of the co-crystallized ligand (sorafenib **I**) in the vicinity of the active site of the enzyme with an energy score of (S) = −15.19 kcal/mol and RMSD of 0.470Å (for further details, see SI). The validated setup was then used in predicting the ligand-target interactions of the newly synthesized compounds at the active site.

## 4. Conclusions

In the present study, a new series of 1,2-disubstituted benzo[*d*]imidazoles were rationally designed as VEGFR-2 inhibitors targeting hepatocellular carcinoma. Our first aim was to study the effect of replacing the 5-methylfuryl moiety at the two-position of the well-known antiangiogenic 2-furylbenzimidazoles, with an isopropyl moiety on the cytotoxic activity against the HepG2 cell line, as well as on VEGFR-2 inhibition. Compounds **13d** (IC_50_ = 11.93 μM), **13f** (IC_50_ = 16.67 μM) and **13g** (IC_50_ = 13.90 μM) displayed higher cytotoxic activity in comparison to the 2-furylbenzimidazole **IX** (IC_50_ = 22.58 μM). In addition, they displayed moderate to slightly lower potency against VEGFR-2 (% inhibition = 78%, 78% and 64%, respectively) in comparison to sorafenib (**I**) (% inhibition = 92%). Subsequently, further optimization of the type III-like VEGFR-2 benzimidazole structures **XI**, **13a**–**h** and **14a**–**d** through extension of the side chain at their one-position to obtain type II-like VEGFR-2 inhibitors was performed. Introduction of different benzyloxy groups was found to have a great influence on the activity. The benzimidazoles **17a** (IC_50_ = 1.98 μM) and **17b** (IC_50_ = 10.04 μM) demonstrated greater cytotoxic activity in comparison to both sorafenib (**I**) (IC_50_ = 10.99 μM) and to **XI**, **13a**–**h** and **14a**–**d**. At 10 µM, the benzimidazoles **17a** and **17b,** exhibiting the 3-benzyloxyphenyl group as an extension, displayed a broad spectrum of antiproliferative activity against various NCI cancer cell lines. In addition, **17a** and **17b** (% inhibition = 82% and 78%, respectively) displayed promising VEGFR-2 inhibitory activity in the HepG2 cell line in comparison to sorafenib (% inhibition = 92%). Besides, **17a** was found to have potent triple-angiokinase inhibitory activity against VEGFR-2, FGFR-1 and PDGFR-β. Analysis of the HepG2 cell cycle after treatment with **17a** revealed that the cell cycle was arrested at the G2/M phase and a dose-dependent apoptotic effect was induced. In silico molecular docking simulations of the target benzimidazoles in the VEGFR-2 binding site revealed their ability to be accommodated and stabilized in the active site by performing the essential hydrogen-bonding and hydrophobic interactions with the key amino acids in the active site. Hence, it can be concluded that series **17,** especially compounds **17a** and **17b,** are promising scaffolds that can be further optimized for the future discovery of antiangiogenic agents for cancer therapy.

## Figures and Tables

**Table 1:** Results of the in vitro cytotoxic activity of benzimidazoles against the HepG2 cell line.

Entry	Compound	IC_50_ (μM) ^a^ on HepG2 Cell Line
**1**	**4a**	> 200
**2**	**4b**	102.54
**3**	**6**	13.52
**4**	**8a**	> 200
**5**	**8b**	> 150
**6**	**8c**	> 200
**7**	**8d**	> 150
**8**	**9a**	32.53
**9**	**10a**	301.35
**10**	**13a**	158.14
**11**	**13b**	136.84
**12**	**13c**	140.11
**13**	**13d**	11.93
**14**	**13e**	86.97
**15**	**13f**	16.67
**16**	**13g**	13.90
**17**	**13h**	59.85
**18**	**14a**	25.88
**19**	**14b**	26.16
**20**	**14c**	112.35
**21**	**14d**	62.00
**22**	**17a**	1.98
**23**	**17b**	10.04
**24**	**17c**	20.18
**25**	**17d**	15.55
**26**	**17e**	24.11
**27**	**17f**	42.49
**28**	**19a**	109.47
**29**	**19b**	71.73
**30**	**19c**	133.39
**31**	**19d**	114.17
**32**	**22a**	122.93
**33**	**22b**	106.88
**34**	**24a**	80.68
**35**	**24b**	15.92
**36**	**24c**	8.73
**37**	**Sorafenib (I)**	10.99

^a^ IC_50_ are presented as mean of three independent experiments.

**Table 2:** In vitro growth inhibition % (GI%) of some selected 1,2-disubstituted benzimidazoles against a panel of tumor cell lines at 10 µM.

Subpanel		Growth Inhibition %					
	14a	17a	17b	17c	17d	17e	17f
**Leukemia**							
CCRF-CEM	16	41	28	- ^a^	-	-	19
HL-60(TB)	-	32	50	-	19	-	-
K-562	-	57	54	28	41	25	34
MOLT-4	-	50	58	25	40	22	50
PRMI-8226	-	nd	50	nd	nd	nd	nd
SR	nd ^b^	64	25	-	16	-	25
Non-small cell lung Cancer							
A549/ATTC	-	16	42	-	23	-	27
EKVX	-	21	43	-	21	-	25
HOP-62	-	-	15	-	17	-	-
HOP-92	18	nd	32	-	nd	-	nd
NCI-H226	22	29	25	-	-	-	16
NCI-H23	-	39	14	-	-	-	-
NCI-H322M	-	-	21	-	20	-	-
NCI-H460	-	-	27	-	-	-	-
NCI-H522	-	33	28	26	27	32	26
Colon Cancer							
HCT-116	-	66	32	18	-	-	28
HCT-15	-	-	35	-	25	-	26
HT29	-	-	29	-	-	-	-
KM12	-	-	23	-	-	-	15
SW-620	18	-	13	-	-	-	-
CNS Cancer							
SF-268	-	-	37	-	23	-	24
SNB-19	-	22	21	-	-	-	20
SNB-75	18	15	39	-	20	-	17
U251	-	-	36	-	-	-	-
Melanoma							
LOX IMVI	-	-	23	-	17	-	-
M14	-	16	24	-	-	-	-
MDA-MB-435	-	-	21	-	-	-	-
SK-MEL-5	-	50	33	-	15	17	23
UACC-62	13	41	29	26	-	22	22
Ovarian Cancer							
OVCAR-4	-	-	21	.	-	-	-
OVCAR-5	21	-	14	-	-	-	-
OVCAR-8	-	-	26	-	20	-	19
NCI/ADR-RES	-	-	29	-	-	-	-
Renal Cancer							
786-0	-	24	26	-	-	-	-
A498	49	17	16	-	17	22	21
CAK1-1	12	-	33	15	21	-	17
RXF 393	33	-	40	-	-	-	-
TK-10	-	-	10	-	-	-	-
UO-31	-	44	57	28	30	20	38
Prostate Cancer							
PC-3	-	58	46	17	29	20	37
Breast Cancer							
MCF7	-	-	48	-	19	-	24
MDA-MB-231/ATTC	-	25	25	-	18	-	21
HS 578T	24	20	25	16	32	-	30
BT-549	-	28	-	-	-	-	-
T-47D	-	32	49	24	31	27	31
MDA-MB-468	29	47	20	-	-	-	23

^a^ Growth inhibition % produced by the compound is below 10%; ^b^ not determined.

**Table 3:** Results of the in vitro growth inhibitory of benzimidazoles **17a** and **17b** against the HSF cell line.

Entry	Compound	IC_50_ (μM) ^a^ on HSF Cell Line
**1**	17a	19.90
**2**	17b	4.80

^a^ IC_50_ are presented as mean of three independent experiments.

**Table 4:** Results of the effects of the synthesized benzimidazoles on VEGFR-2 inhibitory activity in the HepG2 cell line.

Entry	Product	Amount of VEGFR-2 (ng/mL) ^a^	% Inhibition of VEGFR-2 in HepG2 Cell Line
**1**	**6**	37.11 ± 3.00	80
**2**	**13a**	175.00 ± 15.65	5.5
**3**	**13b**	177.11 ± 20.67	4
**4**	**13c**	170.85 ± 20.38	8
**5**	**13d**	39.90 ± 4.60	78
**6**	**13e**	165.71 ± 18.20	10
**7**	**13f**	41.10 ± 3.88	78
**8**	**13g**	66.00 ± 8.35	64
**9**	**13h**	173.80 ± 15.76	6
**10**	**14a**	90.00 ± 8.65	51
**11**	**14b**	72.60 ± 9.14	61
**12**	**14c**	180.50 ± 21.00	3
**13**	**14d**	169.12 ± 16.80	9
**14**	**17a**	34.00 ± 4.28	82
**15**	**17b**	40.12 ± 5.70	78
**16**	**17c**	66.13 ± 9.11	64
**17**	**17d**	81.70 ± 9.16	56
**18**	**17e**	61.86 ± 7.85	67
**19**	**17f**	158.60 ± 19.35	14
**20**	**19a**	173.16 ± 19.66	6.5
**21**	**19b**	168.80 ± 16.21	9
**22**	**19c**	177.10 ± 18.80	4
**23**	**19d**	205.71 ± 23.30	11
**24**	**22a**	183.00 ± 19.00	1
**25**	**22b**	160.85 ± 15.70	13
**26**	**24a**	166.00 ± 19.50	10
**27**	**24c**	74.18 ± 9.70	60
**28**	**24b**	68.81 ± 8.14	63
**29**	**Sorafenib**	14.60 ± 1.02	92
**30**	**DMSO**	185.20 ± 21.85	-

^a^ Data were expressed as mean ± standard error (S.E.) of two experiments.

**Table 5:** Biochemical kinase inhibitory activity of selected benzimidazoles on VEGFR-2, FGFR-1 and PDGFR-β.

Entry	Compound	IC_50_ (µM) ^a^		
		VEGFR-2	FGFR-1	PDGFR-β
1	**13d**	0.09 ± 0.002	nd ^b^	nd
2	**13f**	0.40 ± 0.01	nd	nd
3	**17a**	0.11 ± 0.003	0.11 ± 0.004	0.05 ± 0.002
4	**17b**	0.14 ± 0.004	nd	nd
5	**Sorafenib (I)**	0.10 ± 0.02	0.65 ± 0.10	0.06 ± 0.02

^a^ IC_50_ are presented as mean of two independent experiments; ^b^ not determined.

**Table 6:** Docking energy scores (S) in kcal/mol for the newly synthesized compounds and the reference compound (sorafenib **I**) in the VEGFR-2 active site.

Entry	Product	Energy Score (S) kcal/mol	Entry	Product	Energy Score (S) kcal/mol
1	**6**	−11.54	16	**17c**	−13.53
2	**13a**	−12.78	17	**17d**	−13.59
3	**13b**	−12.10	18	**17e**	−15.24
4	**13c**	−11.64	19	**17f**	−15.21
5	**13d**	−11.75	20	**19a**	−13.84
6	**13e**	−12.31	21	**19b**	−14.87
7	**13f**	−13.23	22	**19c**	−14.74
8	**13g**	−13.20	23	**19d**	−14.96
9	**13h**	−11.27	24	**22a**	−16.23
10	**14a**	−11.87	25	**22b**	−16.22
11	**14b**	−12.33	26	**24a**	−14.20
12	**14c**	−12.23	27	**24b**	−13.41
13	**14d**	−14.08	28	**24c**	−14.50
14	**17a**	−14.31	29	**Sorafenib (I)**	−15.19
15	**17b**	−15.06			

## Supplementary Materials

The following are available online: Figures S1–S51 NMR spectra of 1,2-disubstituted benzimidazoles.; Figures S52–S56, Table 1 Molecular structure and crystal data of **13c** and **14a**. Figure S57 Docking validation of sorafenic, the co-crystalized liged, in the VEGFR-2 active site. Figures S58–S85 2D diagrams of the newly synthesized 1,2-disubstituted benzimidazoles showing their interaction with the VEGFR-2 active site.
