# Supplementary material for: Targeting Receptor Tyrosine Kinase VEGFR-2 in Hepatocellular Cancer: Rational Design, Synthesis and Biological Evaluation of 1,2-Disubstituted Benzimidazoles
Source: Molecules. 2020 Feb 11;25(4):770. doi: 10.3390/molecules25040770 (PMC7071059; doi:10.3390/molecules25040770)
Supplement: Supplementary file 1 [file molecules-25-00770-s001.pdf]

Targeting Receptor Tyrosine Kinase VEGFR-2 in Hepatocellular Cancer: Rational Design, Synthesis and Biological Evaluation of 1,2-Disubstituted Benzimidazoles

Heba T. Abdel-Mohsen[1]*, Mona A. Abdullaziz[1], Ahmed M. El Kerdawy[2,3]*, Fatma A. F. Ragab[2], Keith J. Flanagan[3], Abeer E.E. Mahmoud[4], Mamdouh M. Ali[4], Hoda I. El Diwani[1], Mathias O. Senge[3]*

[1] Department of Chemistry of Natural and Microbial Products, Division of Pharmaceutical and Drug Industries Research, National Research Centre, Dokki, P.O. 12622, Cairo, Egypt.
[2] Department of Pharmaceutical Chemistry, Faculty of Pharmacy, Cairo University, Kasr El-Aini Street, P.O. Box 11562, Cairo, Egypt.
[3] Department of Pharmaceutical Chemistry, Faculty of Pharmacy, New Giza University, New Giza, km 22 Cairo–Alexandria Desert Road, Cairo, Egypt.
[4] Medicinal Chemistry, Trinity Translational Medicine Institute, Trinity Centre for Health Sciences, Trinity College Dublin, The University of Dublin, St. James's Hospital, Dublin 8, Ireland.
[5] Department of Biochemistry, Division of Genetic Engineering and Biotechnology, National Research Centre, Cairo, Egypt

***** Correspondence: Mathias.Senge@tcd.ie (M.S.), Tel.: 0035318968537; hebabdelmohsen@gmail.com; ht.abdel-mohsen@nrc.sci.eg (H.T. A.-M.); ahmed.elkerdawy@cu.edu.eg (A. M. E.-K).

Contents

		Page
1.	NMR Spectra of 1,2-disubstituted benzimidazoles (Fig. 1-51).	2
2.	Molecular structure and crystal data of **13c** and **14a** (Fig. 52-56 and Tables 1-16)	53
3.	Docking validation of sorafenib, the co-crystalized ligand, in the VEGFR-2 active site (Fig. 57)	87
4.	2D diagrams of the newly synthesized 1,2-disubstituted benzimidazole showing their interaction with the VEGFR-2 active site; distances in Å (Fig. 58-85)	89

1. NMR Spectra of 1,2-disubstituted benzimidazoles (Fig. 1-51)

2-Isopropyl-1*H*-benzo[*d*]imidazole (**4a**)

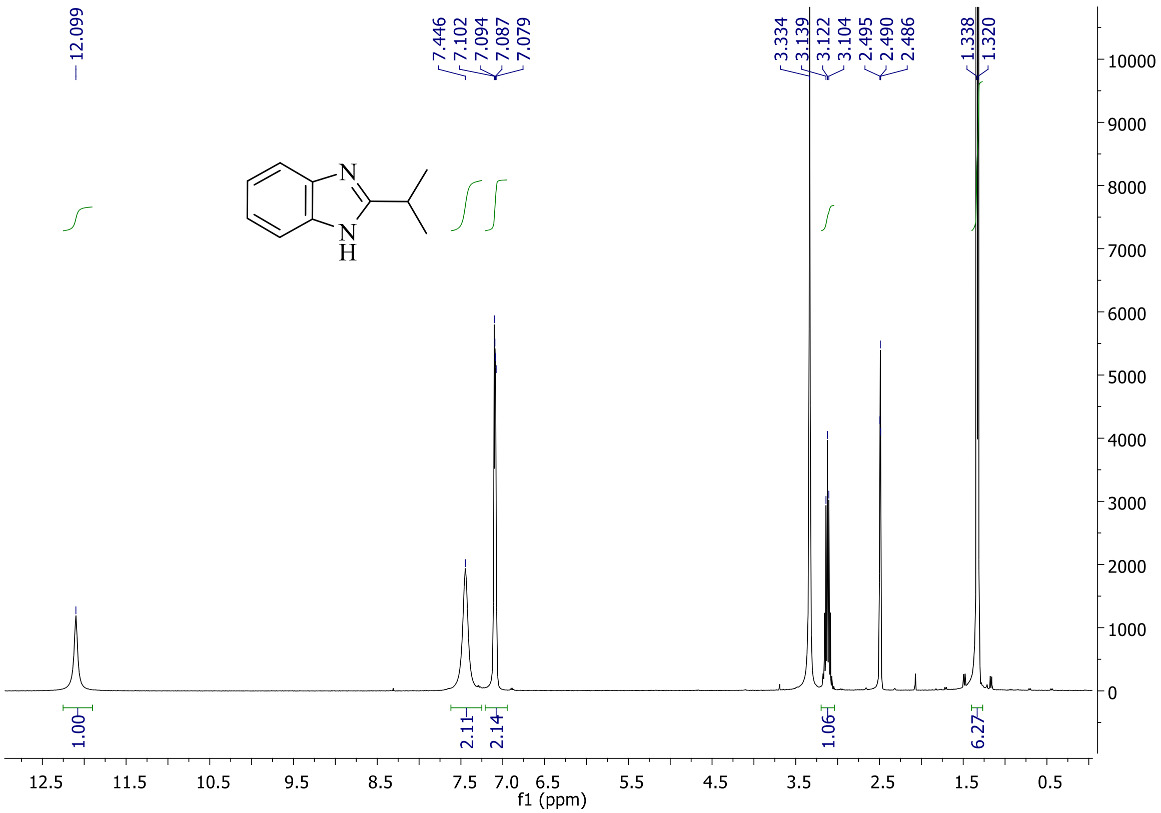

Fig. 1 ^{1}H (400 MHz) NMR spectrum of **4a** in DMSO-d_6

4-(2-(2-Isopropyl-1*H*-benzo[*d*]imidazol-1-yl)acetyl)benzonitrile (**6**)

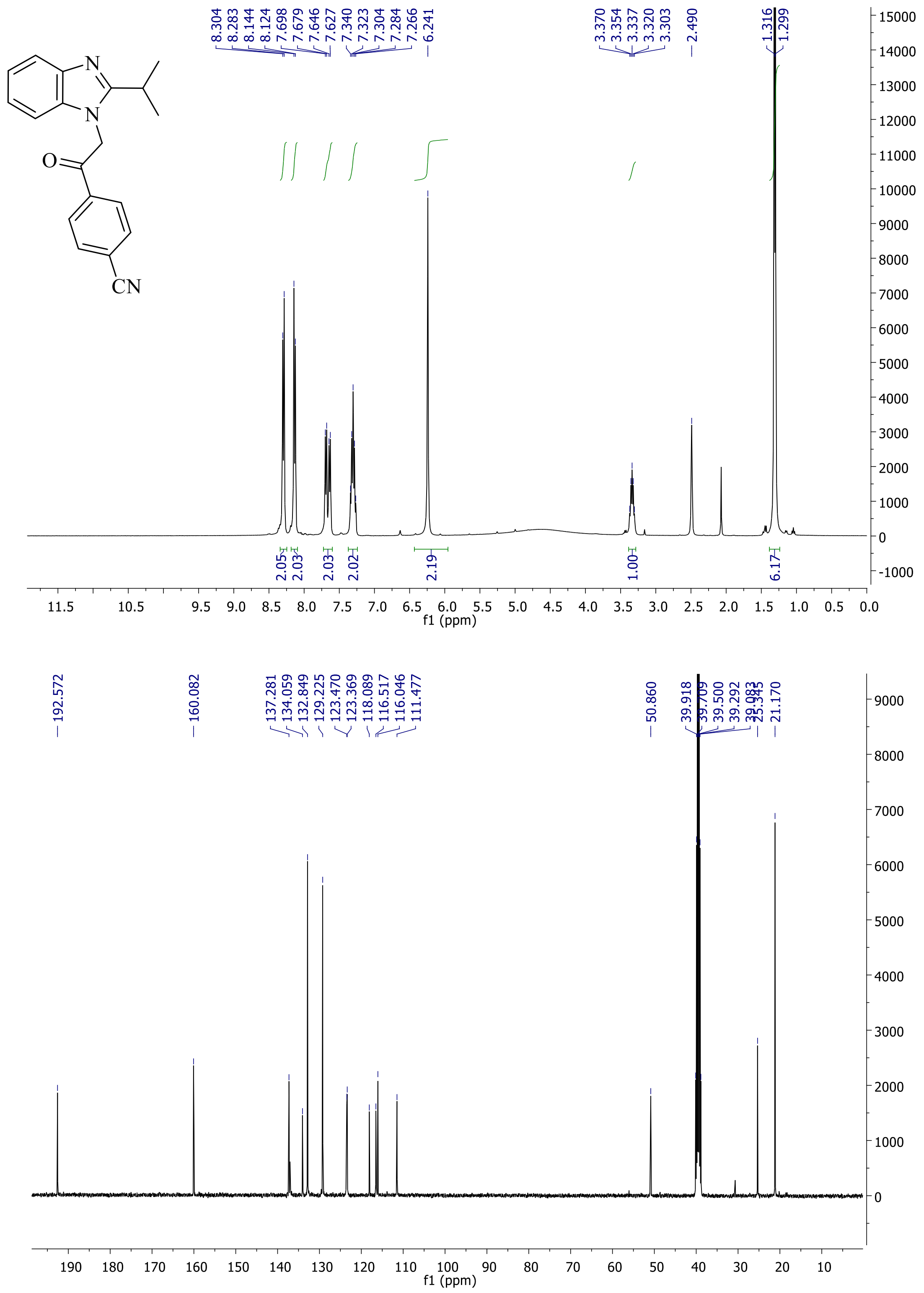

Fig. 2 ^{1}H (400 MHz) and ^{13}C (100 MHz) NMR spectra of **6** in DMSO-d_6

Methyl 2-(2-isopropyl-1*H*-benzo[*d*]imidazol-1-yl)acetate (**8a**)

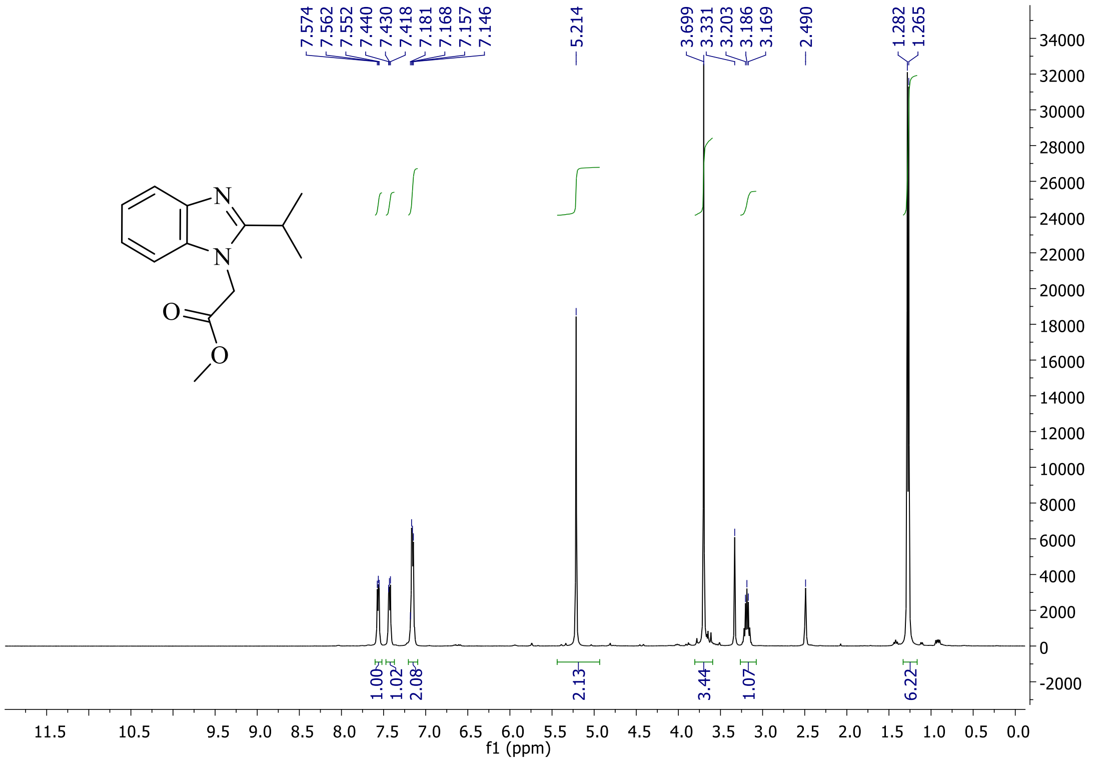

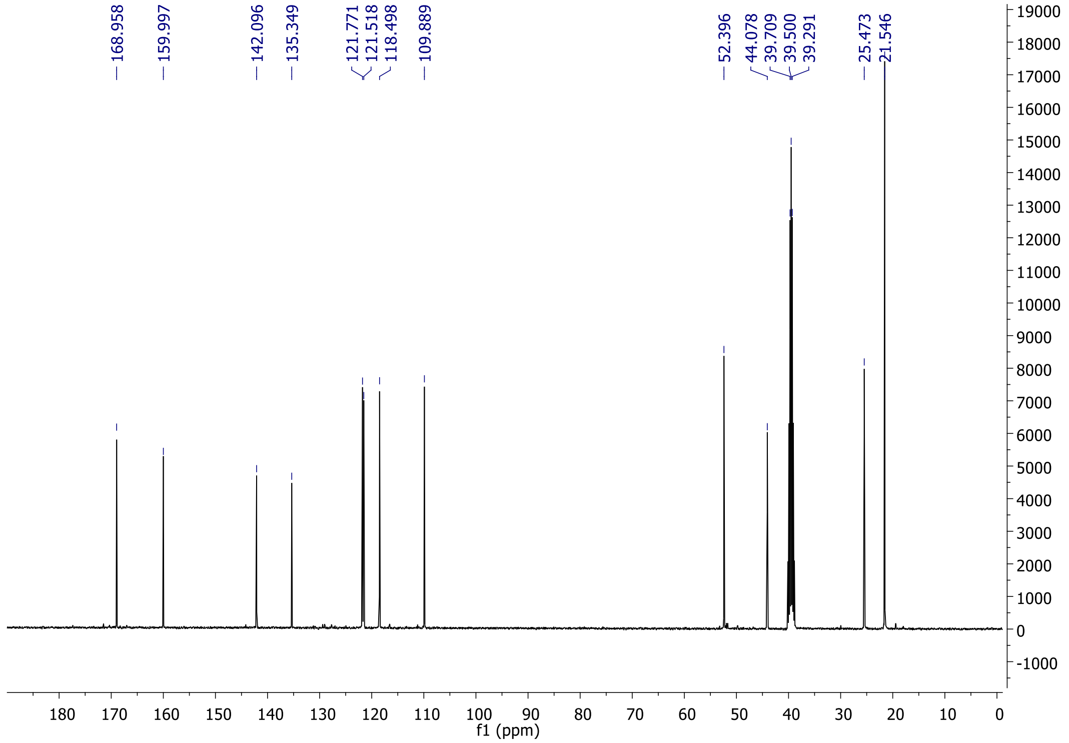

Fig. 3 ^{1}H (400 MHz) and ^{13}C (100 MHz) NMR spectra of **8a** in DMSO-d_6

Ethyl 2-(2-isopropyl-1*H*-benzo[*d*]imidazol-1-yl)acetate (**8c**)

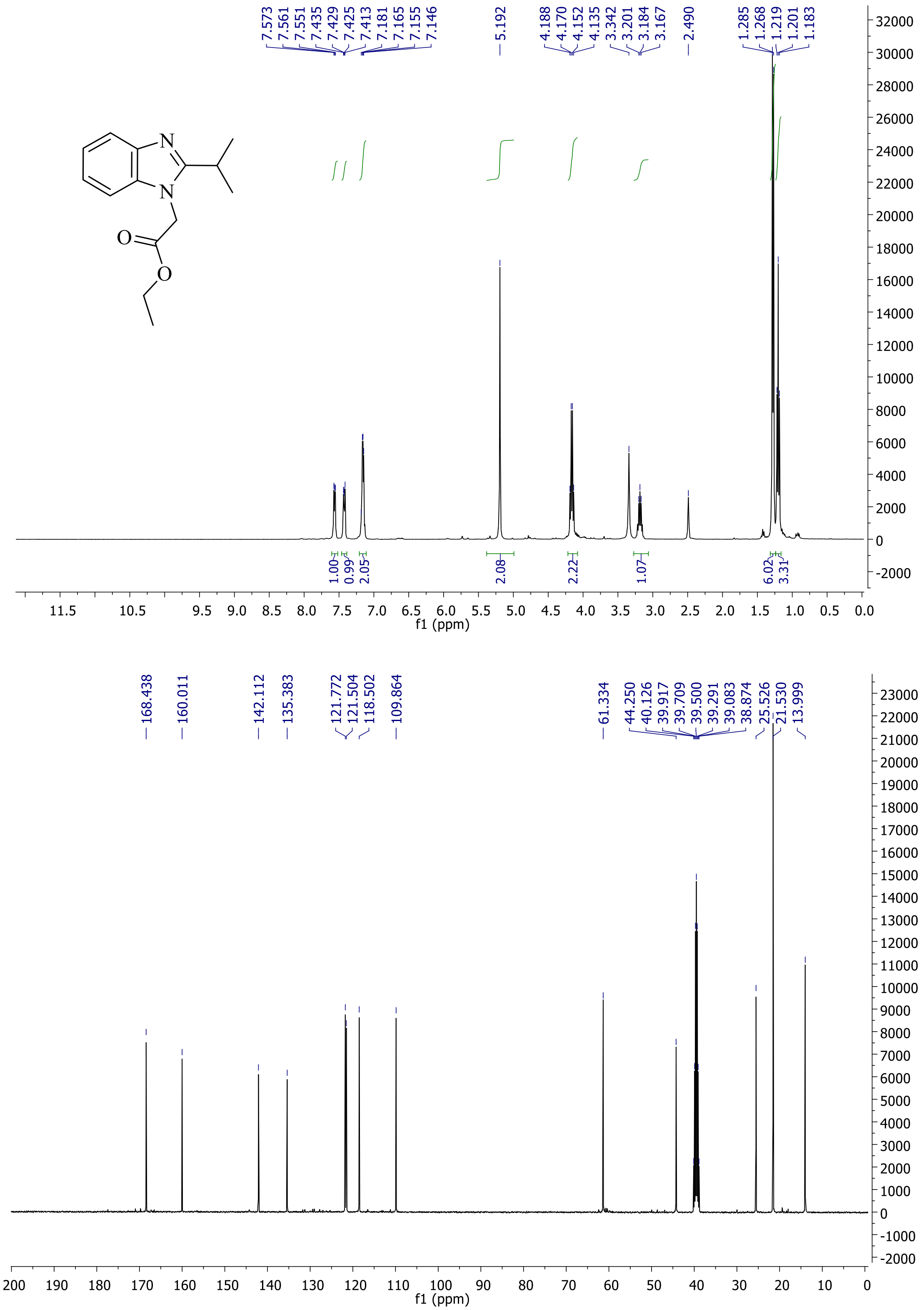

Fig. 4 ^{1}H (400 MHz) and ^{13}C (100 MHz) NMR spectra of **8c** in DMSO-d_6

2-(2-Isopropyl-1*H*-benzo[*d*]imidazol-1-yl)acetic acid (**9a**)

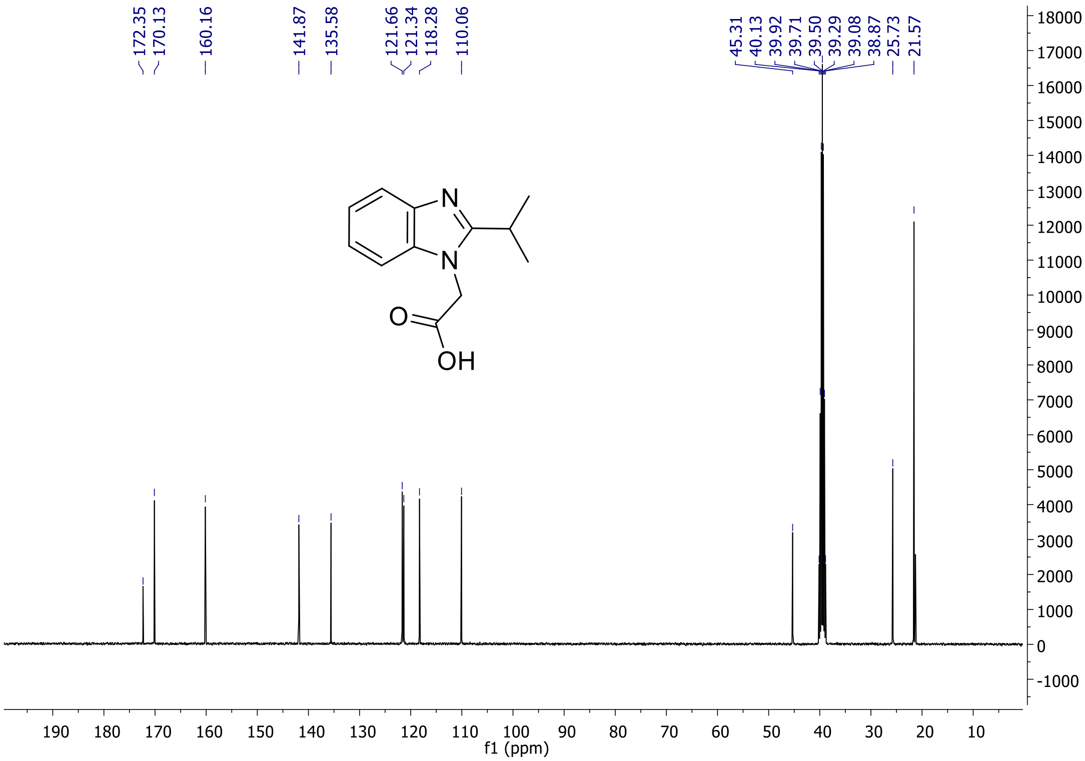

Fig. 5 ^{13}C (100 MHz) NMR spectrum of **9a** in DMSO-d_6

2-(2-Isopropyl-1*H*-benzo[*d*]imidazol-1-yl)acetohydrazide (**10a**)

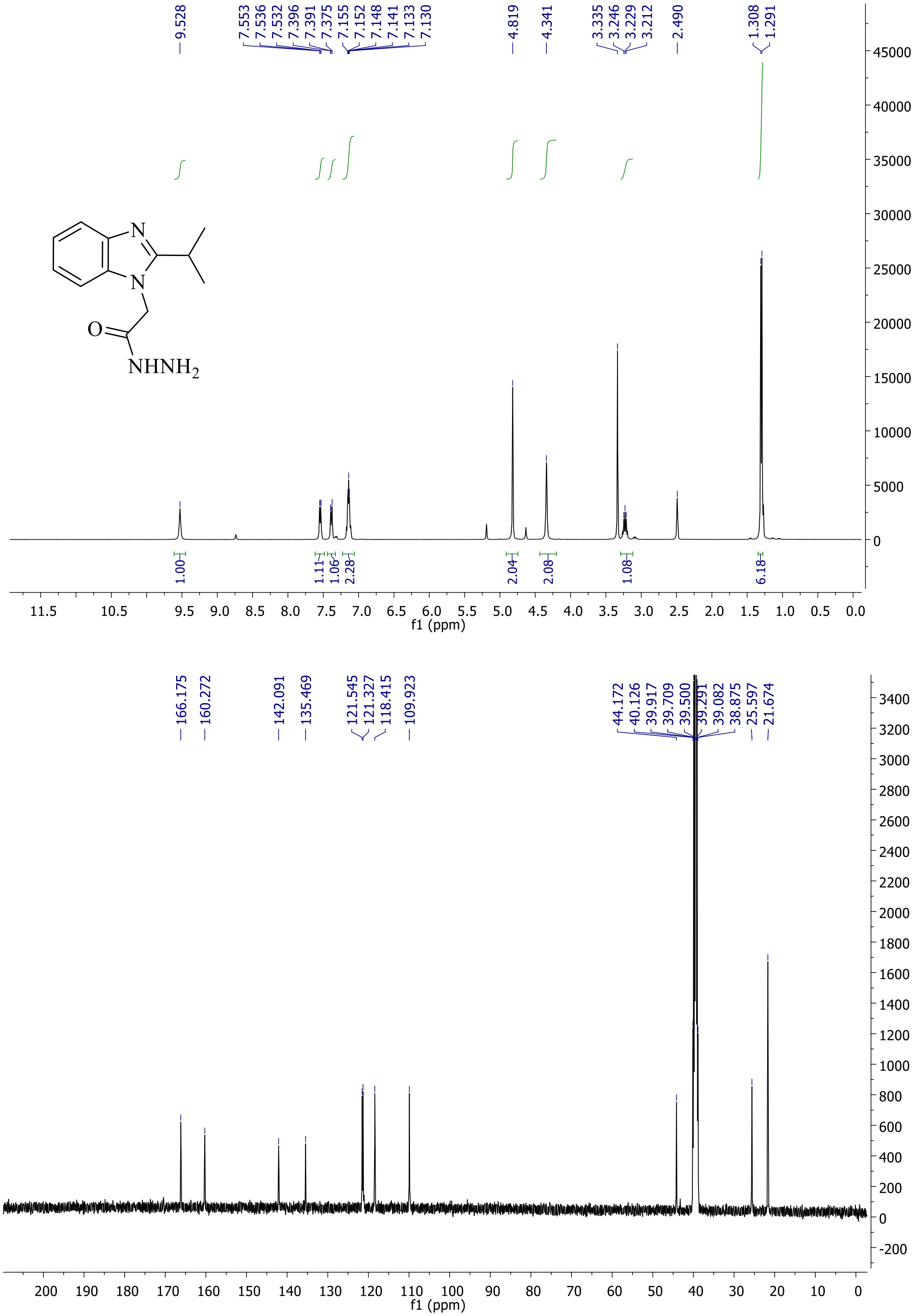

Fig. 6 ^{1}H (400 MHz) and ^{13}C (100 MHz) NMR spectra of **10a** in DMSO-d_6

(*E*)-*N*'-(2-Chlorobenzylidene)-2-(2-isopropyl-1*H*-benzo[*d*]imidazol-1-yl)acetohydrazide (**13a**)

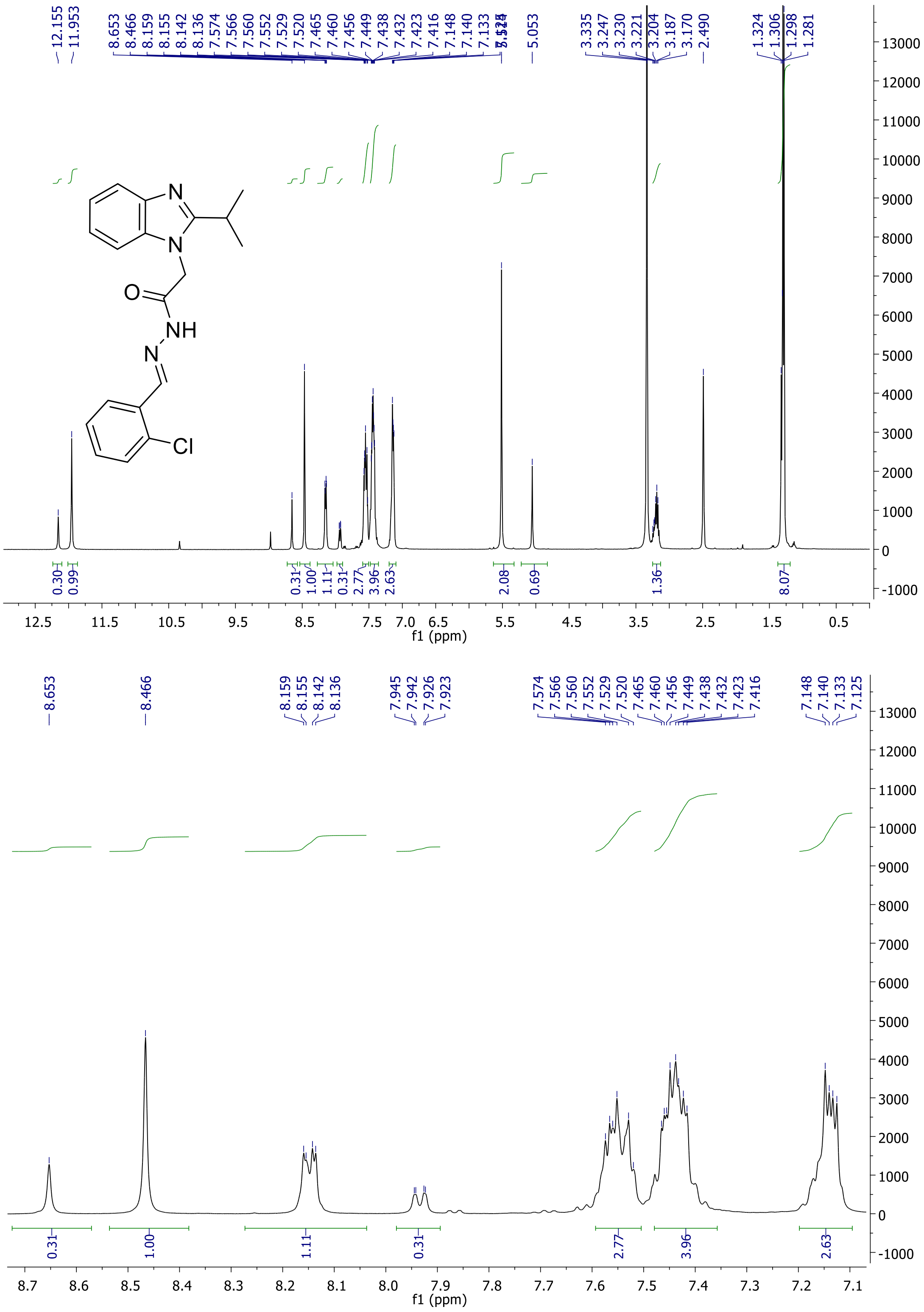

Fig. 7 ^{1}H (400 MHz) NMR spectrum of **13a** in DMSO-d_6

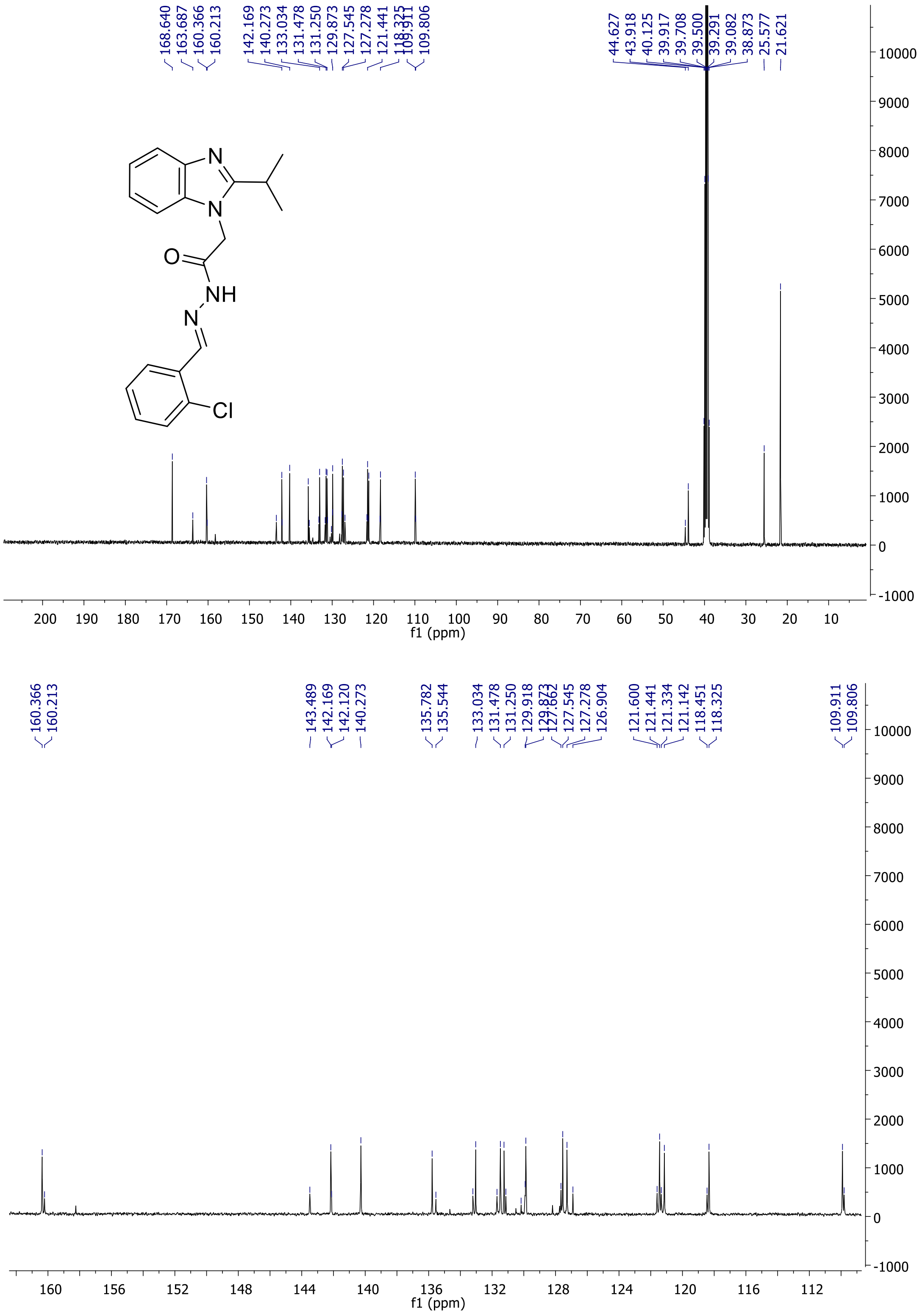

Fig. 8 ^{13}C (100 MHz) NMR spectrum of **13a** in DMSO-d_6

(*E*)-*N*'-(3-hydroxybenzylidene)-2-(2-isopropyl-1*H*-benzo[*d*]imidazol-1-yl)acetohydrazide (**13b**)

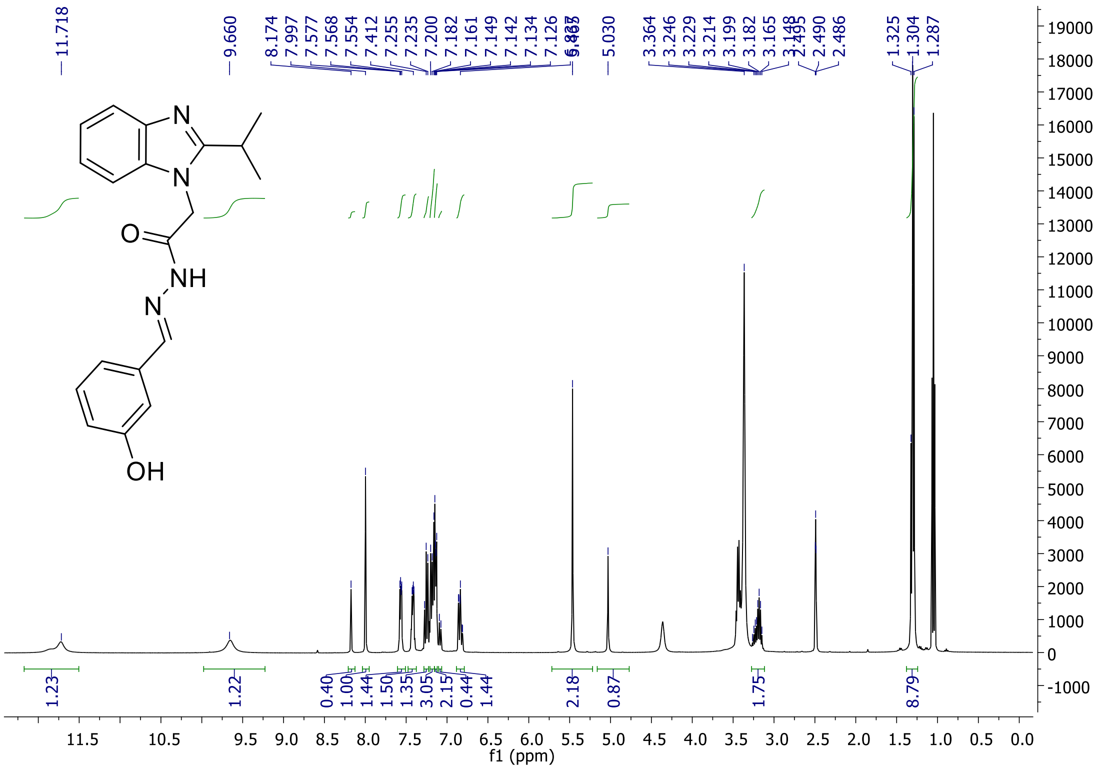

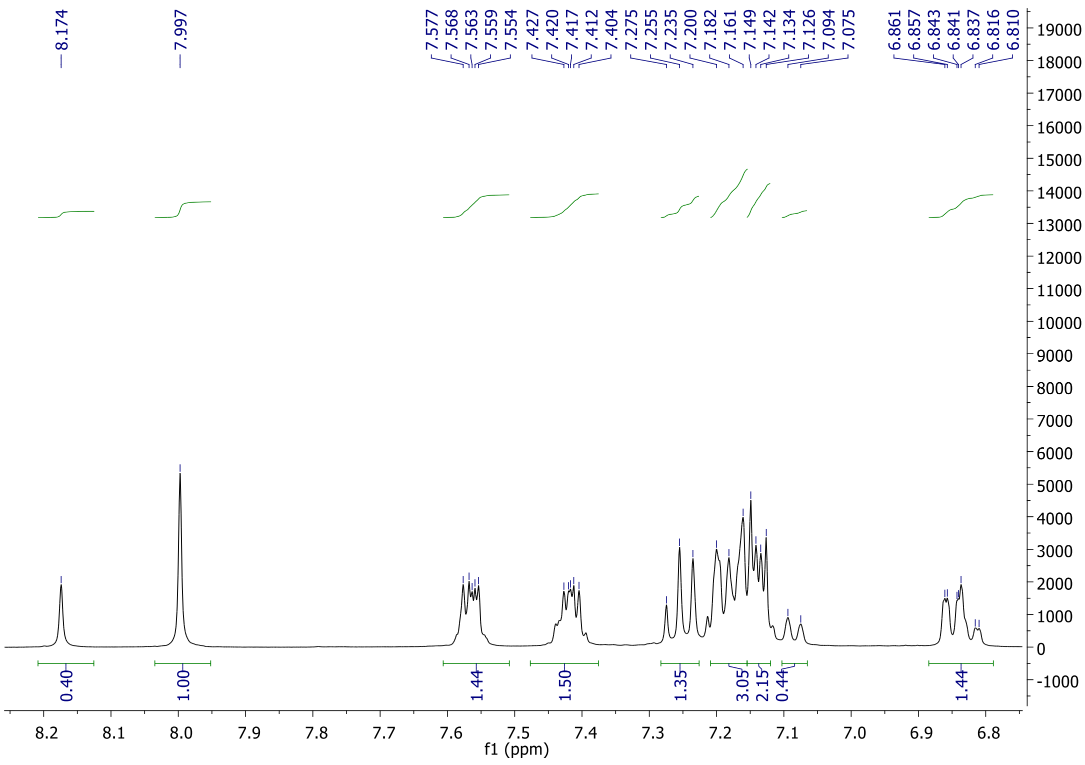

Fig. 9 ^{1}H (400 MHz) NMR spectrum of **13b** in DMSO-d_6

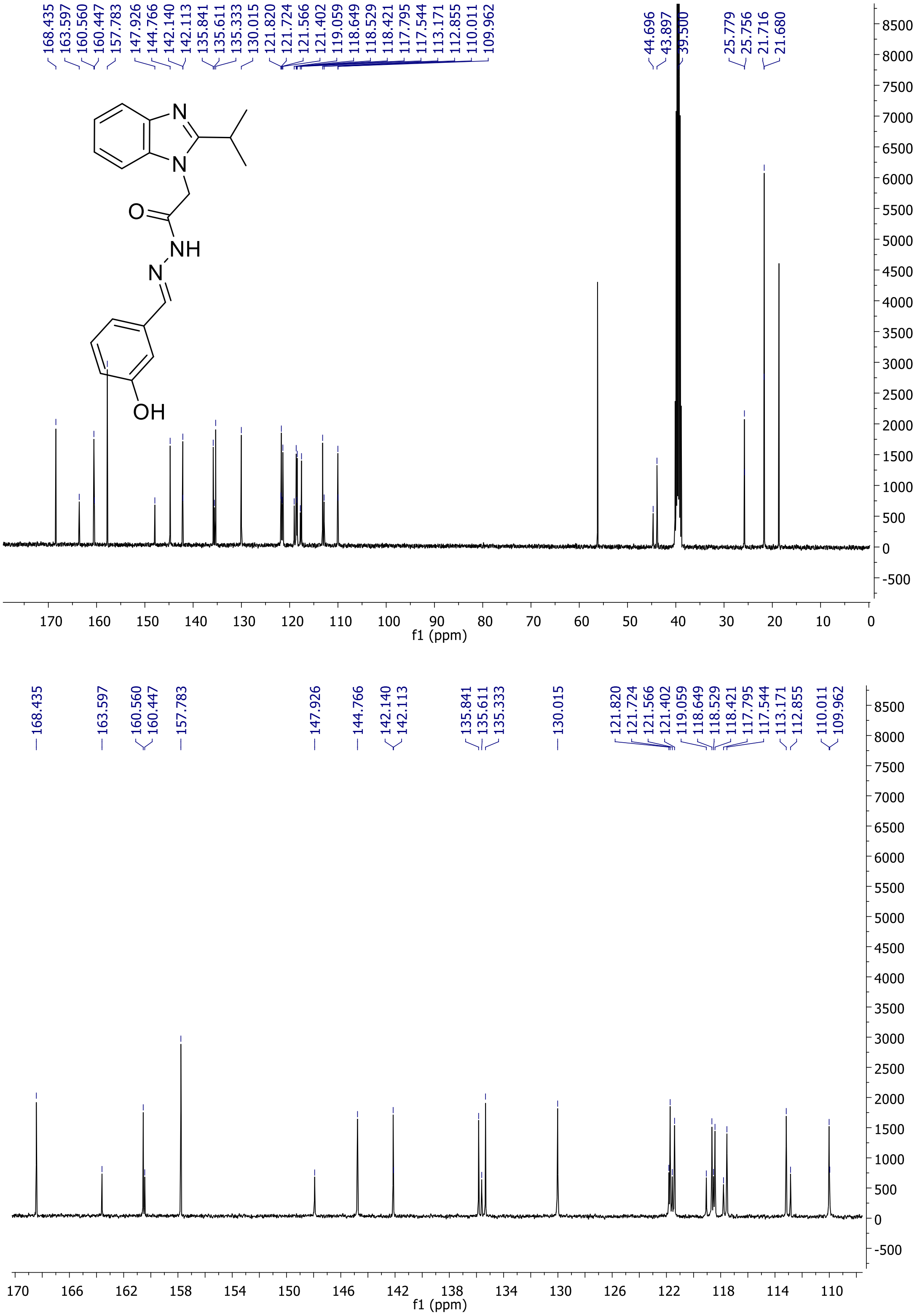

Fig. 10 ^{13}C (100 MHz) NMR spectrum of **13b** in DMSO-d_6

(*E*)-*N*'-(4-hydroxybenzylidene)-2-(2-isopropyl-1*H*-benzo[*d*]imidazol-1-yl)acetohydrazide (**13c**)

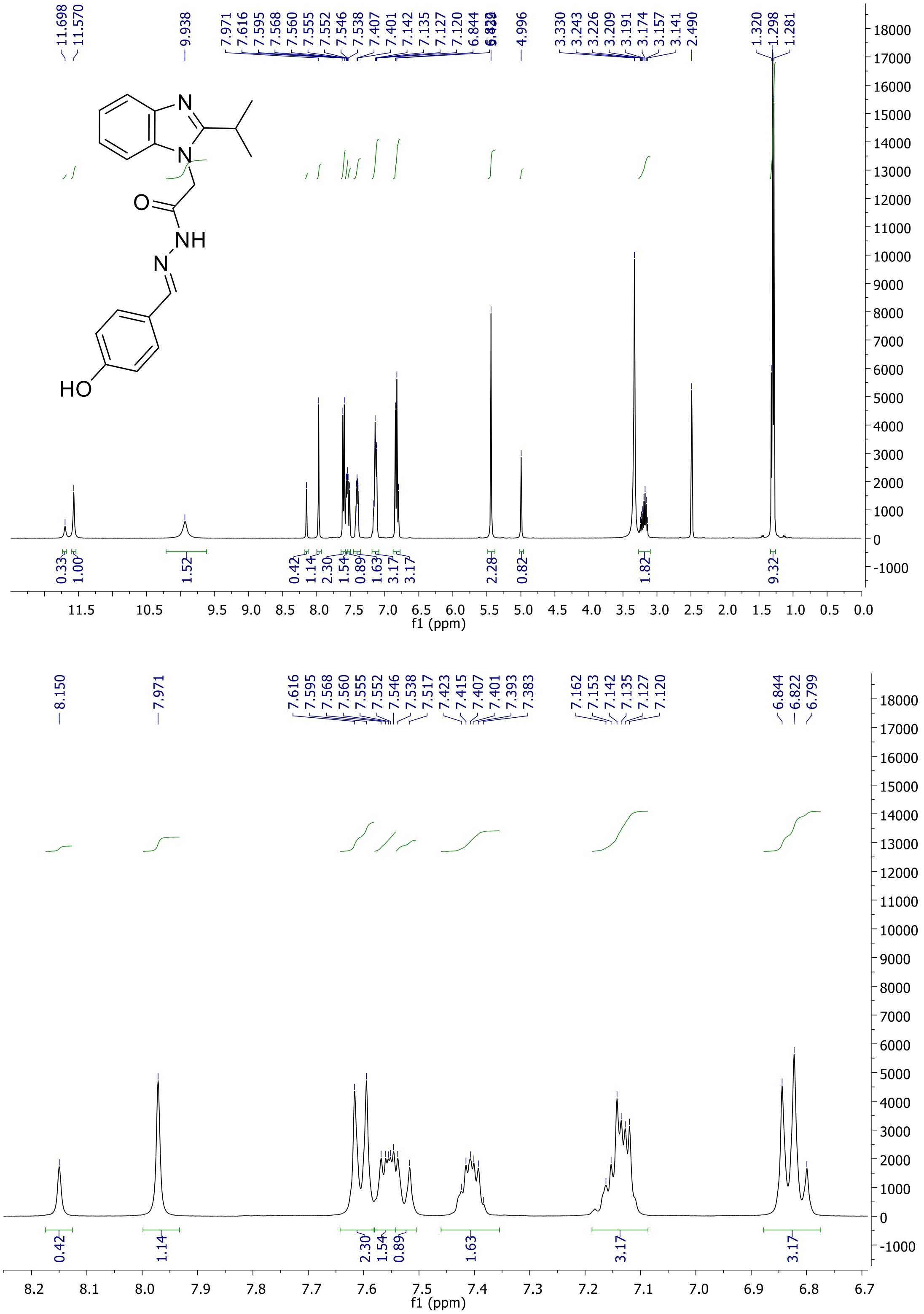

Fig. 11 ^{1}H (400 MHz) NMR spectrum of **13c** in DMSO-d_6

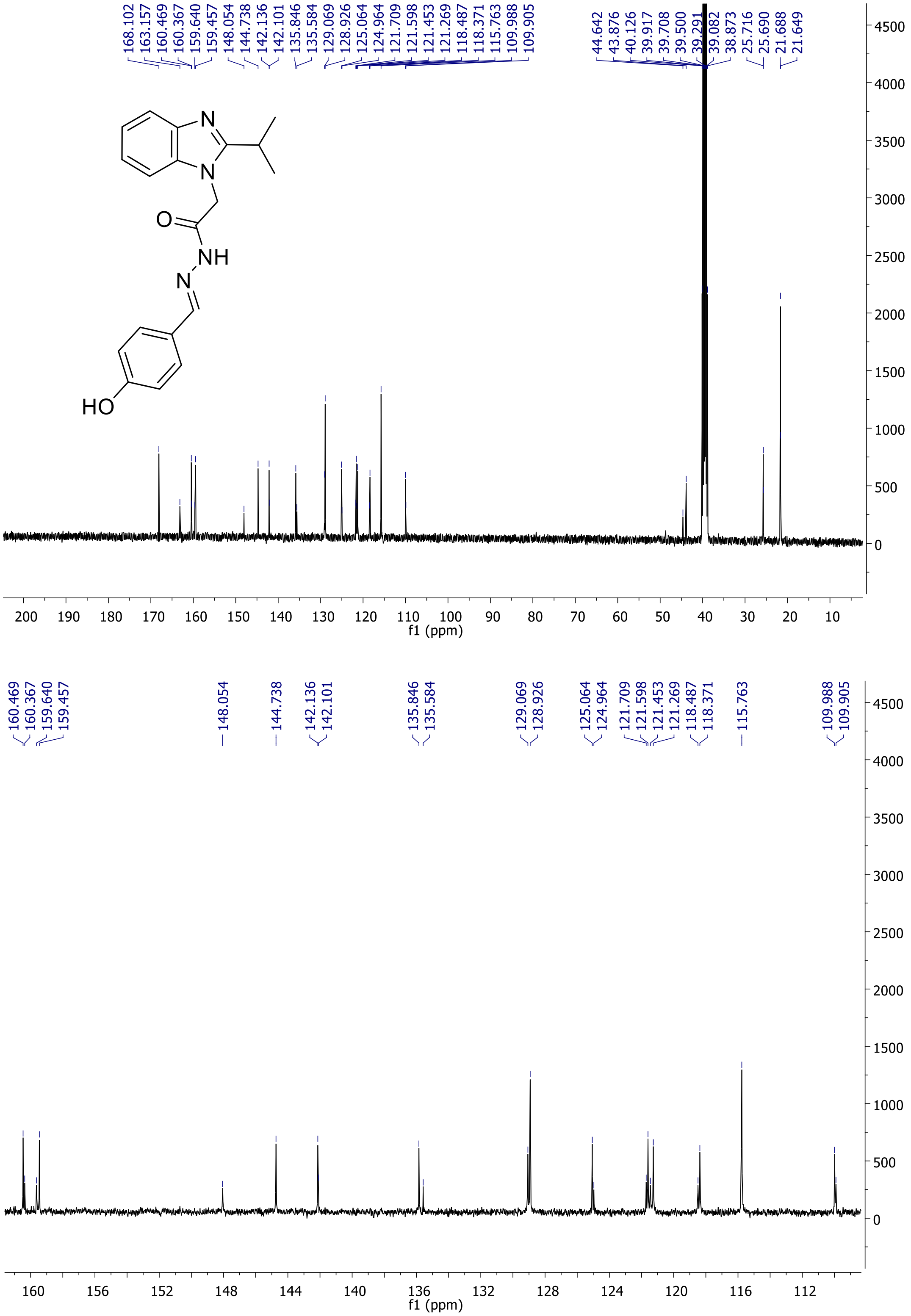

Fig. 12 ^{13}C (100 MHz) NMR spectrum of **13c** in DMSO-d_6

(*E*)-2-(2-Isopropyl-1*H*-benzo[*d*]imidazol-1-yl)-*N*'-(4-methoxybenzylidene)acetohydrazide (**13d**)

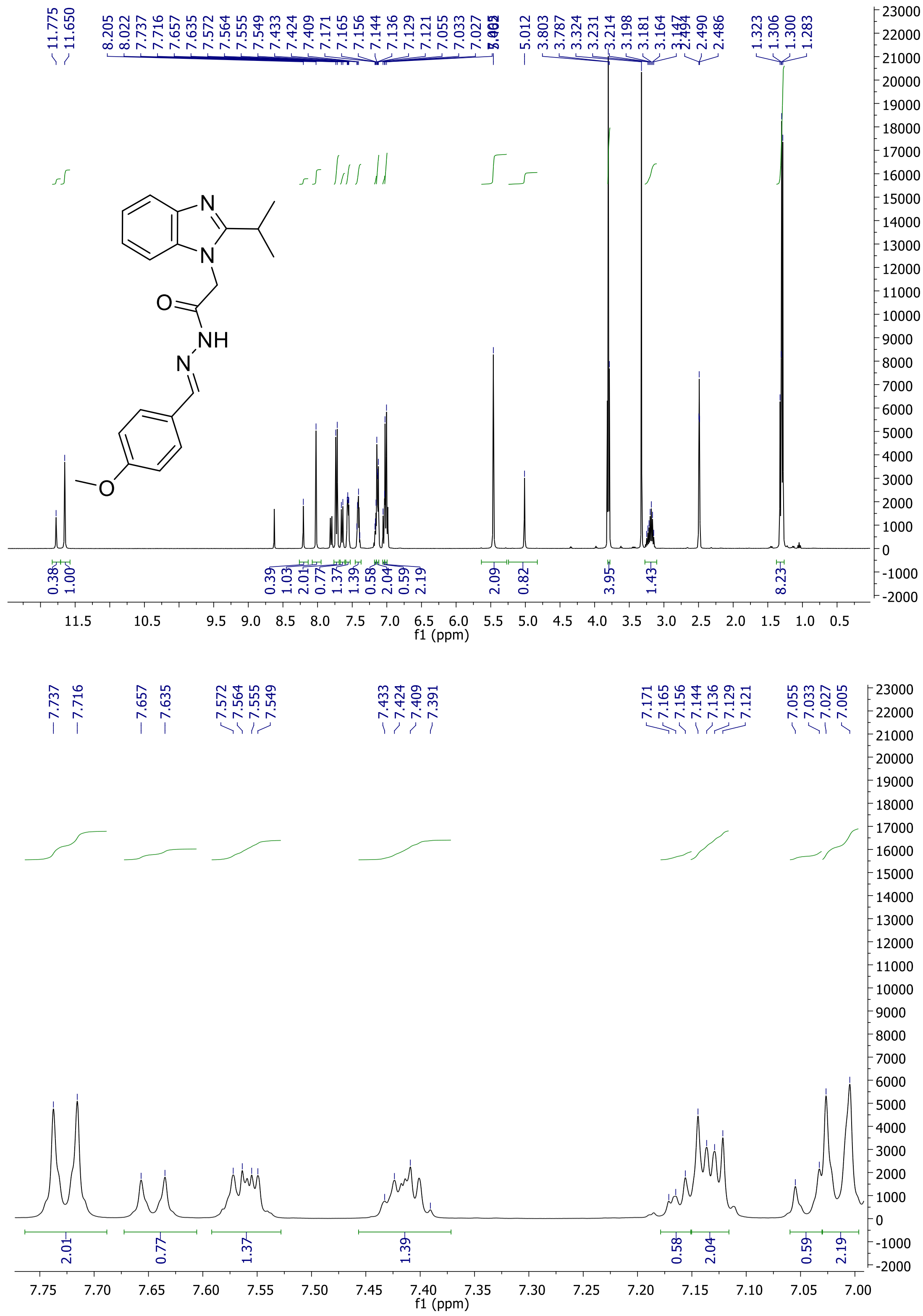

Fig. 13 ^{1}H (400 MHz) NMR spectrum of **13d** in DMSO-d_6

(*E*)-*N*'-(4-(dimethylamino)benzylidene)-2-(2-isopropyl-1*H*-benzo[*d*]imidazol-1-yl)acetohydrazide (**13e**)

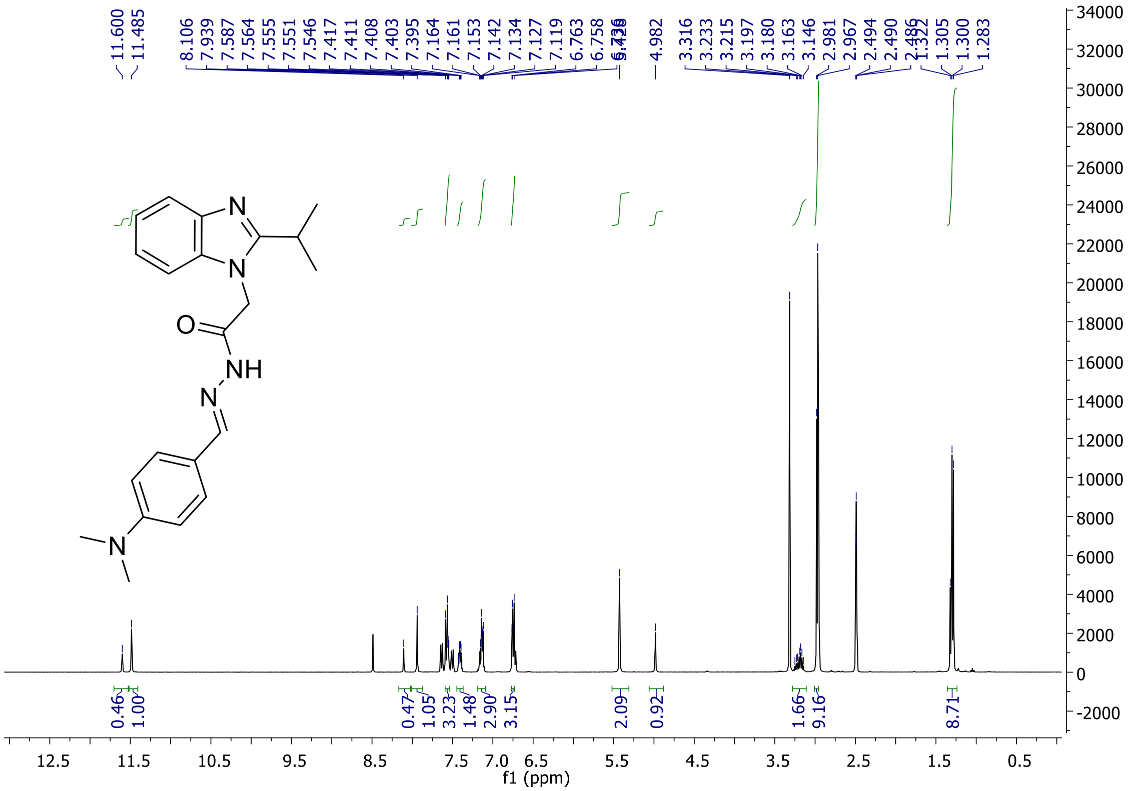

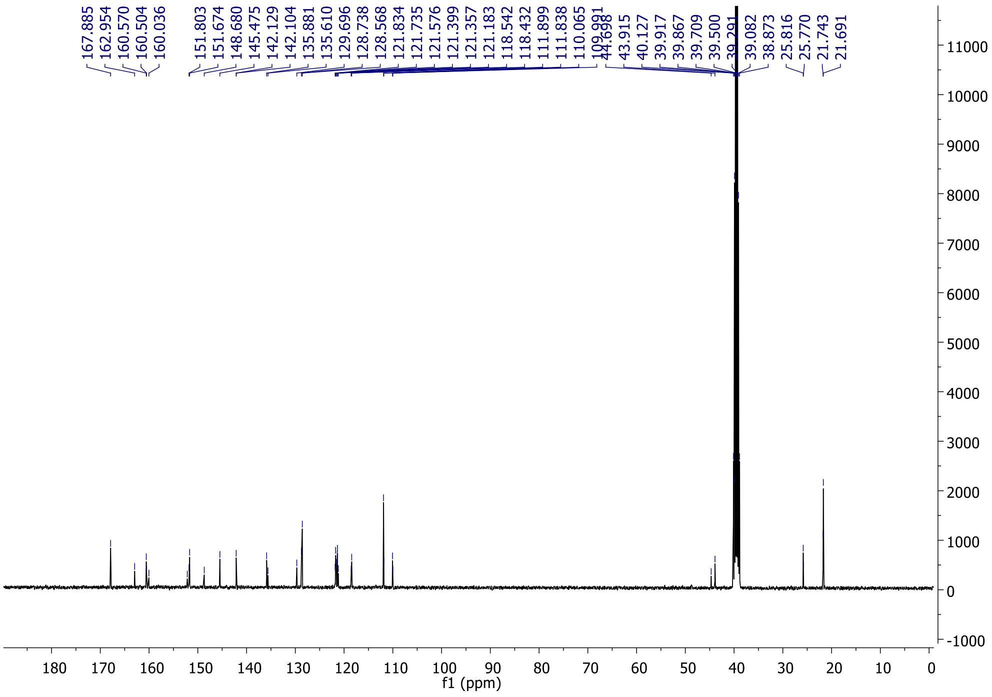

Fig. 14 ^{1}H (400 MHz) and ^{13}C (100 MHz) NMR spectra of **13e** in DMSO-d_6

(*E*)-*N*'-(2,4-dimethoxybenzylidene)-2-(2-isopropyl-1*H*-benzo[*d*]imidazol-1-yl)acetohydrazide (**13f**)

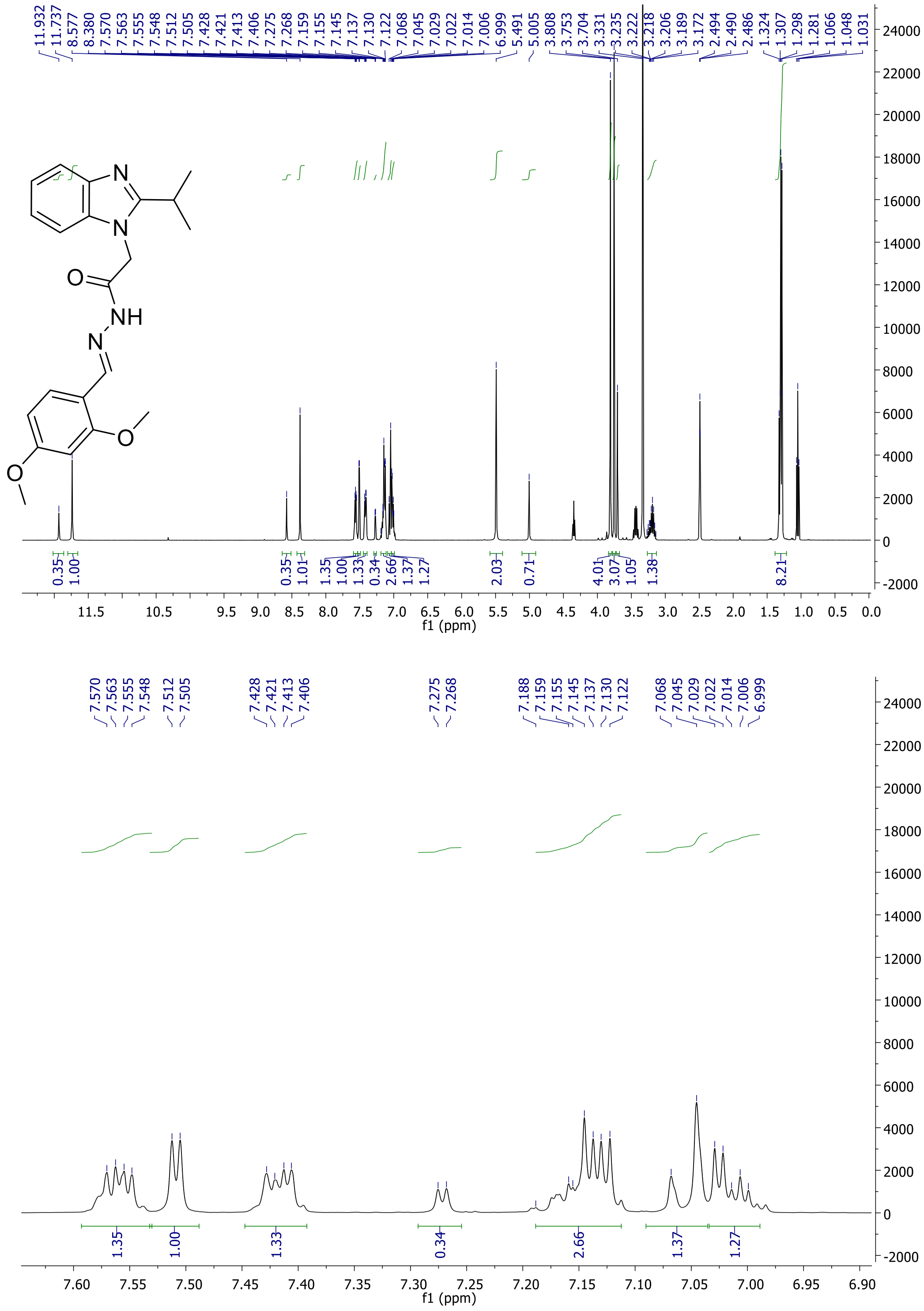

Fig. 15 ^{1}H (400 MHz) NMR spectrum of **13f** in DMSO-d_6

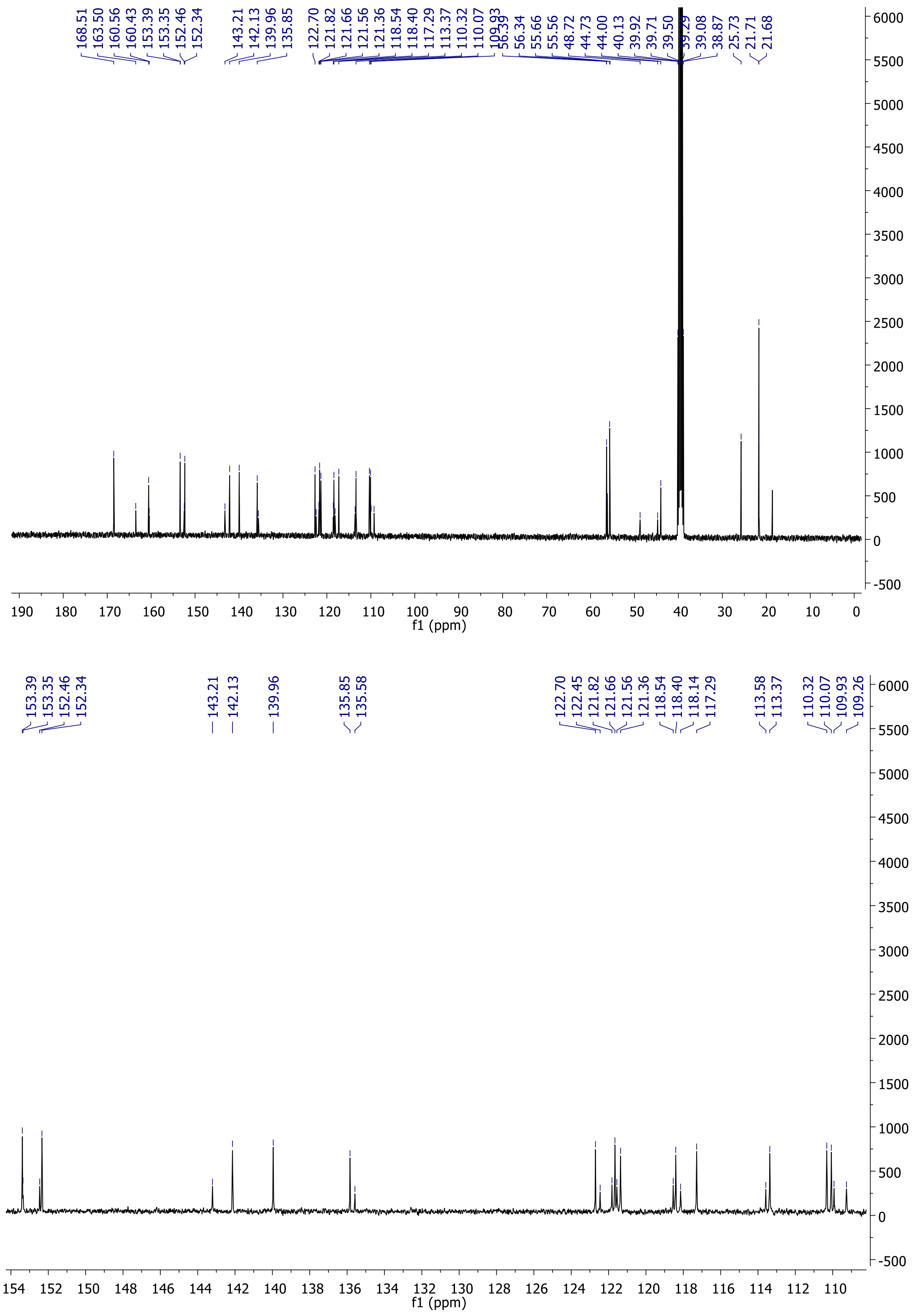

Fig. 16 ^{13}C (100 MHz) NMR spectrum of **13f** in DMSO-d_6

(*E*)-*N*'-(4-hydroxy-3-methoxybenzylidene)-2-(2-isopropyl-1*H*-benzo[*d*]imidazol-1-yl)acetohydrazide (**13g**)

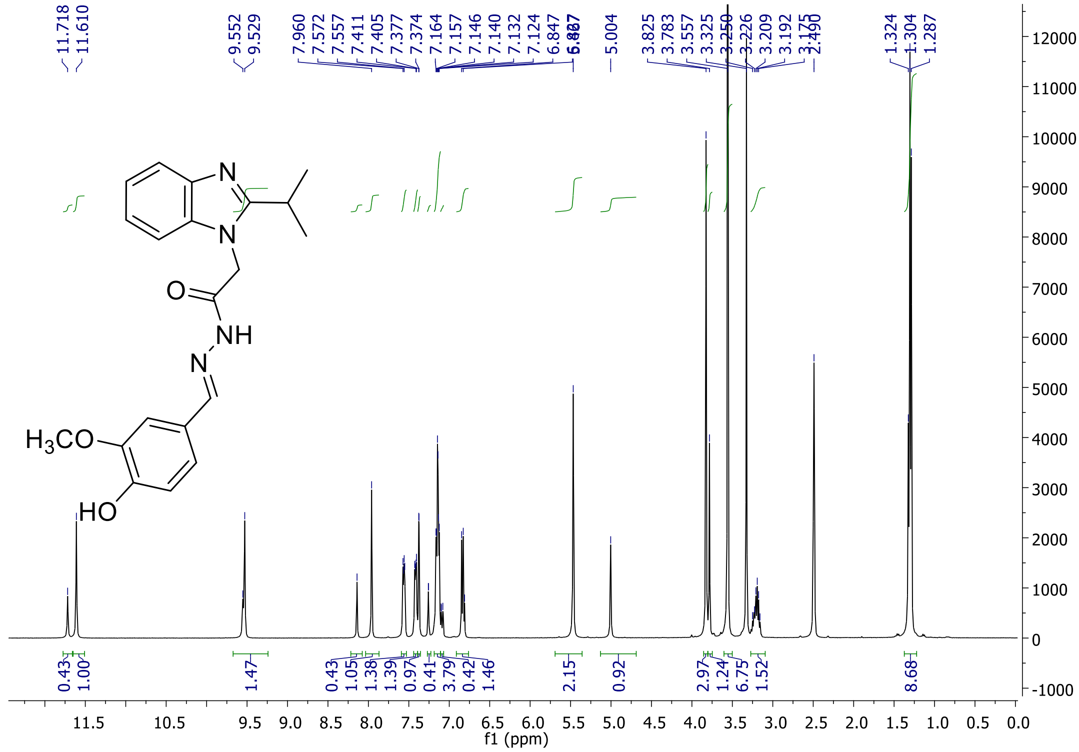

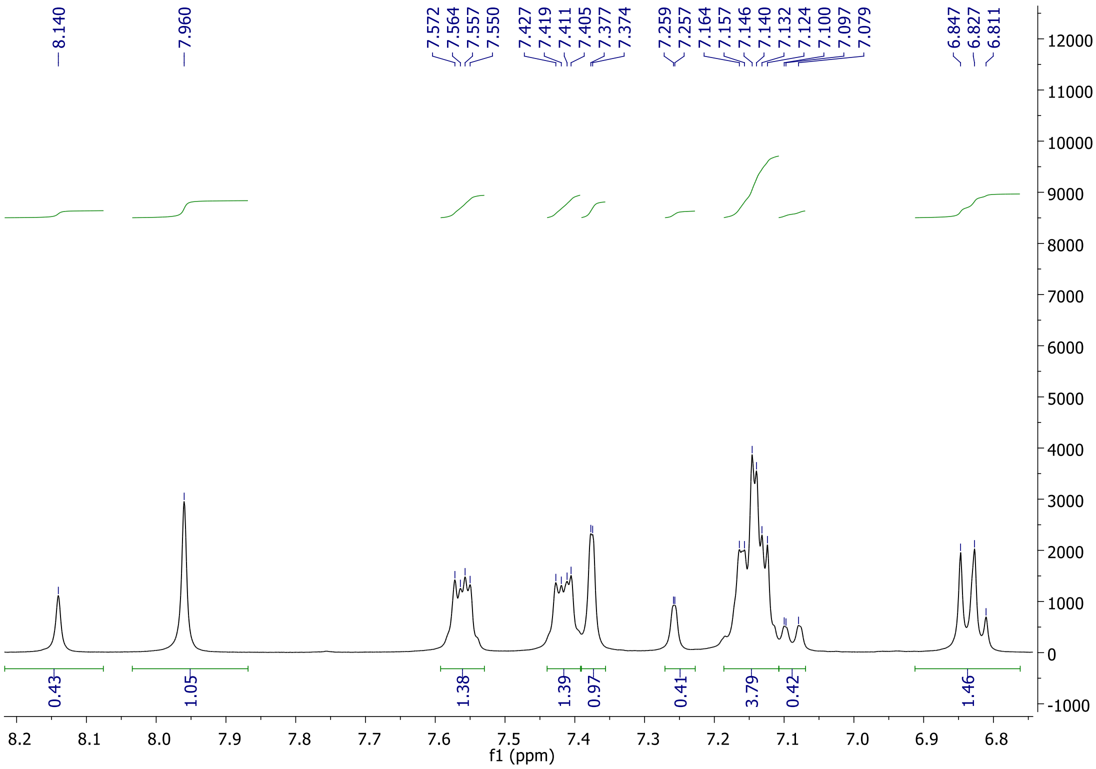

Fig. 17 ^{1}H (400 MHz) NMR spectrum of **13g** in DMSO-d_6

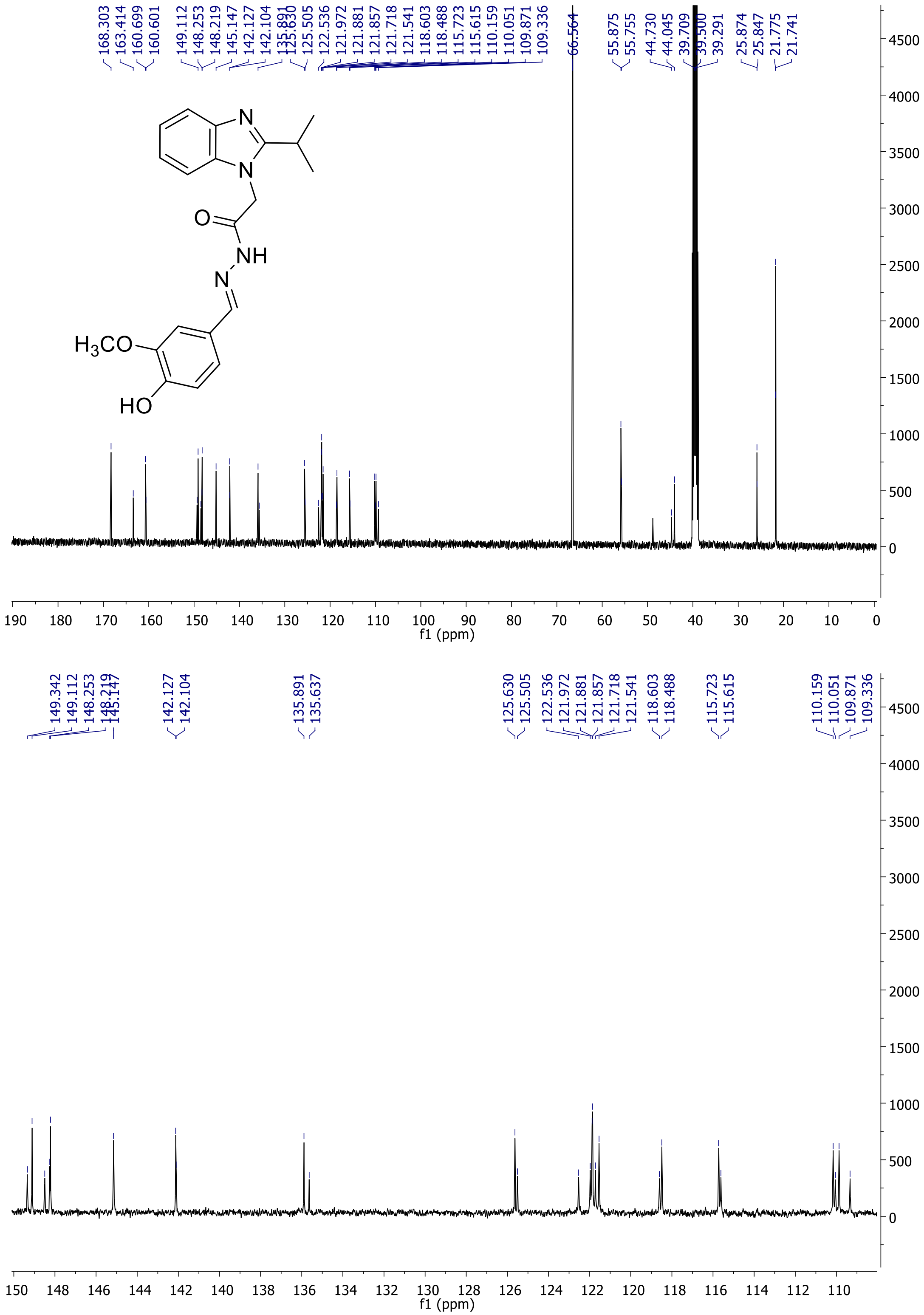

Fig. 18 ^{13}C (100 MHz) NMR spectra of **13g** in DMSO-d_6

(*E*)-2-(2-isopropyl-1*H*-benzo[*d*]imidazol-1-yl)-*N*'-((5-methylfuran-2-yl)methylene)acetohydrazide (**13h**)

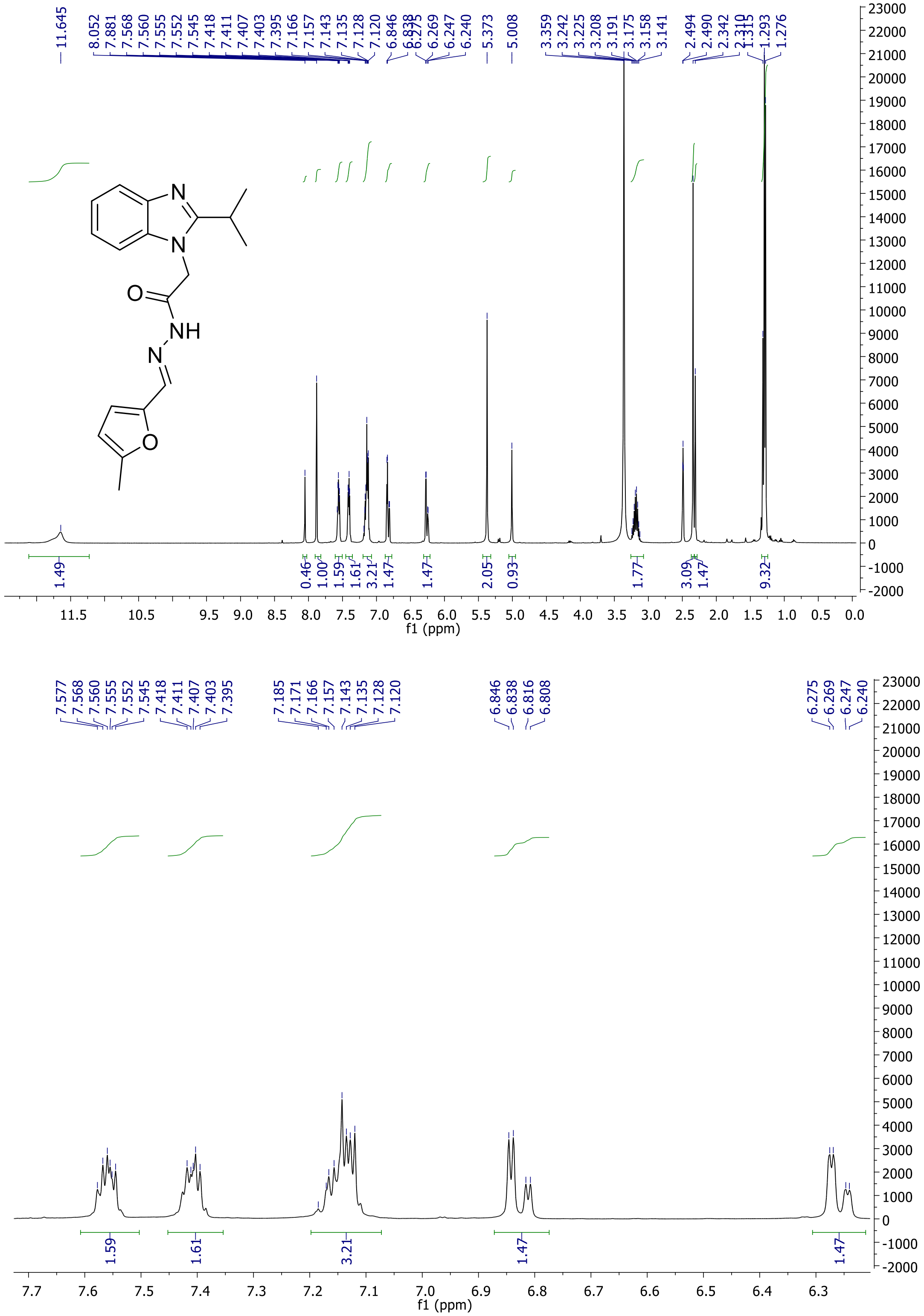

Fig. 19 ^{1}H (400 MHz) NMR spectrum of **13h** in DMSO-d_6

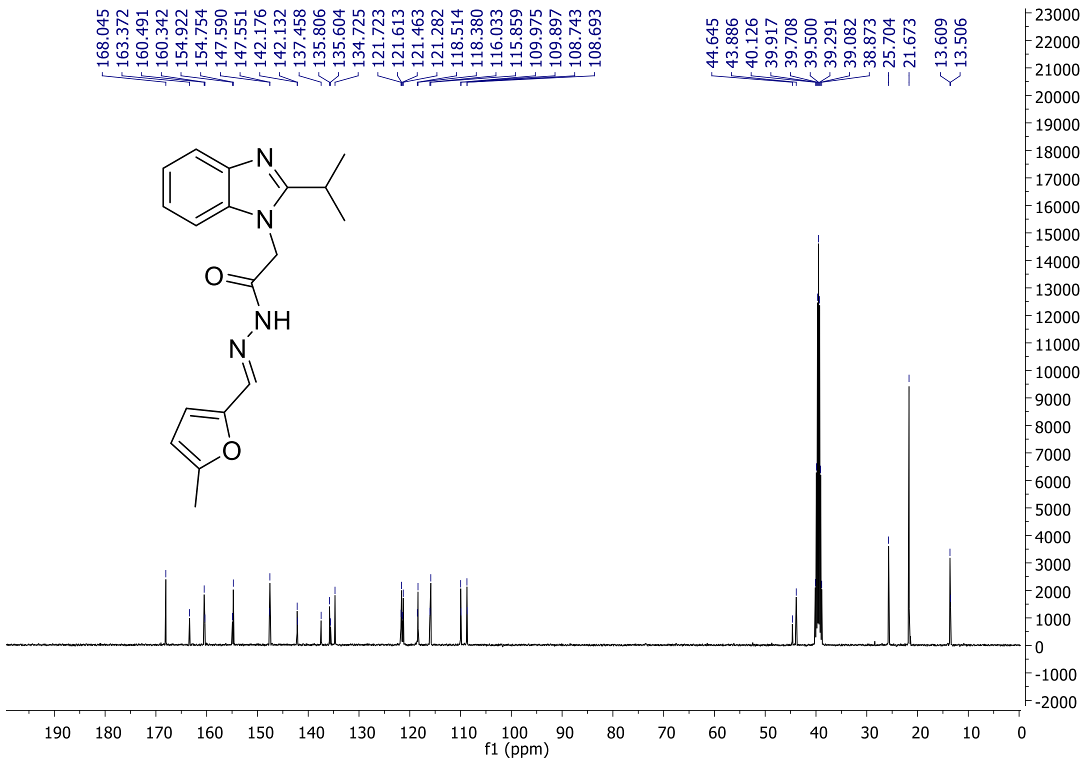

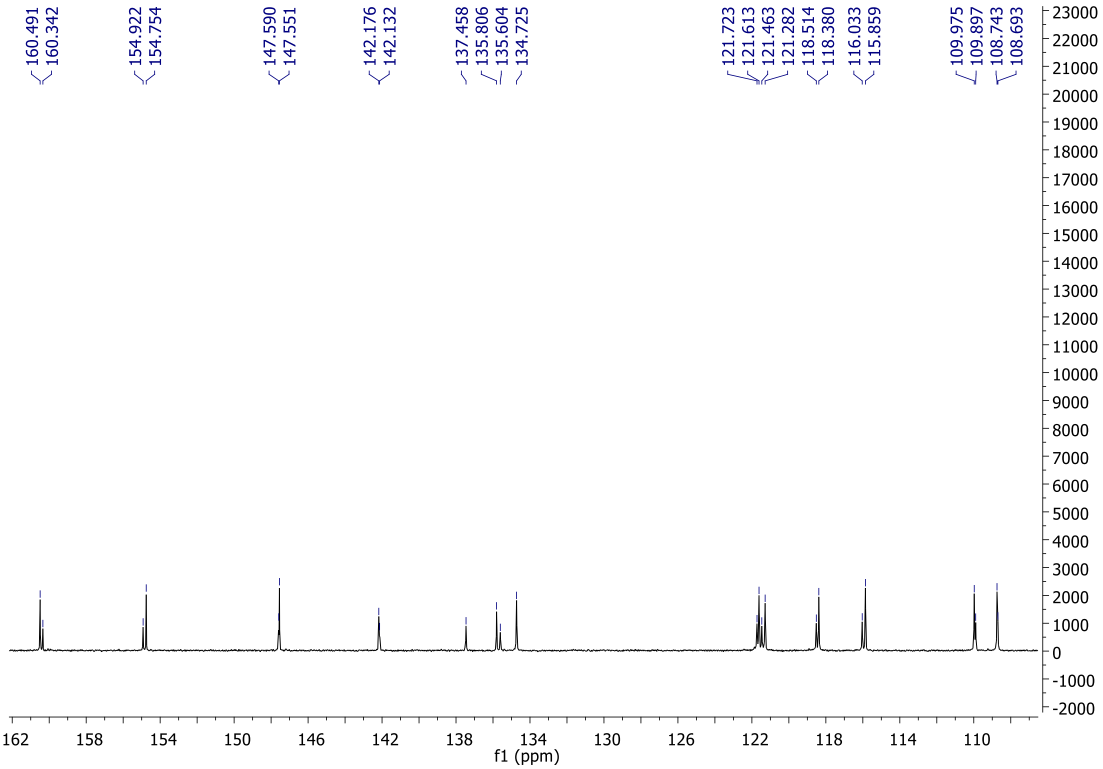

Fig. 20 ^{13}C (100 MHz) NMR spectrum of **13h** in DMSO-d_6

(*E*)-2-(2-isopropyl-1*H*-benzo[*d*]imidazol-1-yl)-*N*'-(1-phenylethylidene)acetohydrazide (**14a**)

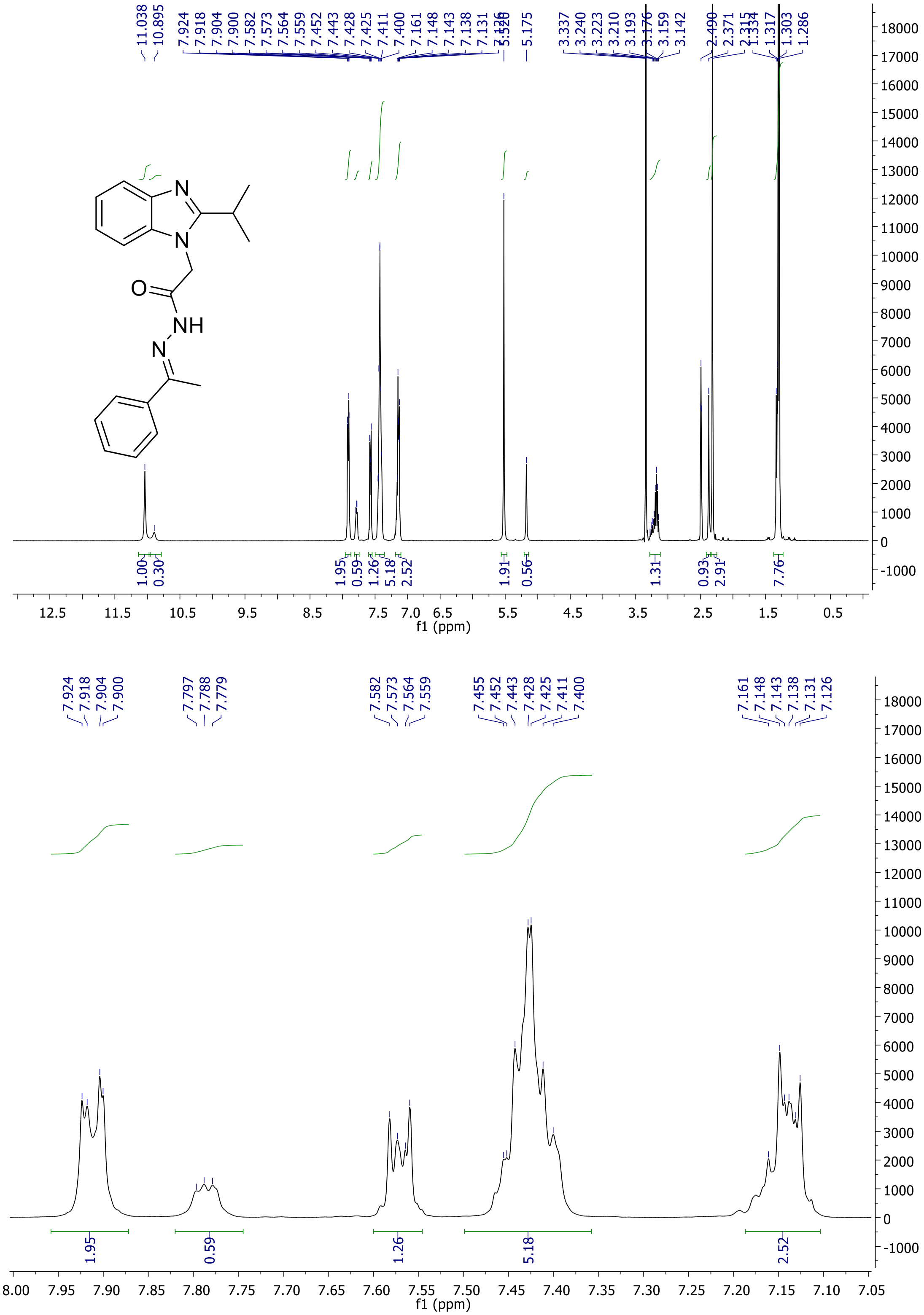

Fig. 21 ^{1}H (400 MHz) NMR spectrum of **14a** in DMSO-d_6

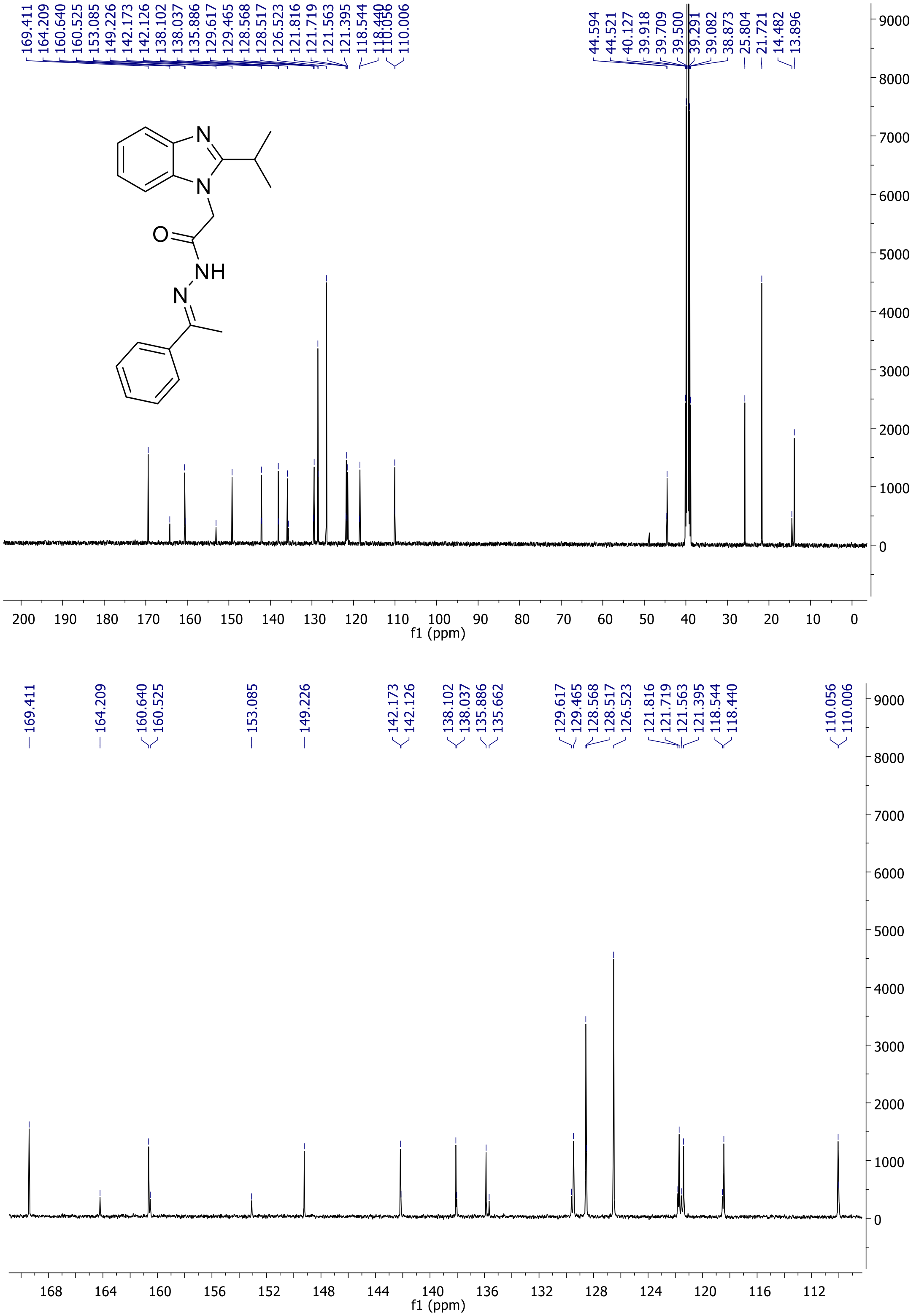

Fig. 22 ^{13}C (100 MHz) NMR spectrum of **14a** in DMSO-d_6

(*E*)-2-(2-isopropyl-1*H*-benzo[*d*]imidazol-1-yl)-*N*'-(1-(*o*-tolyl)ethylidene)acetohydrazide (**14b**)

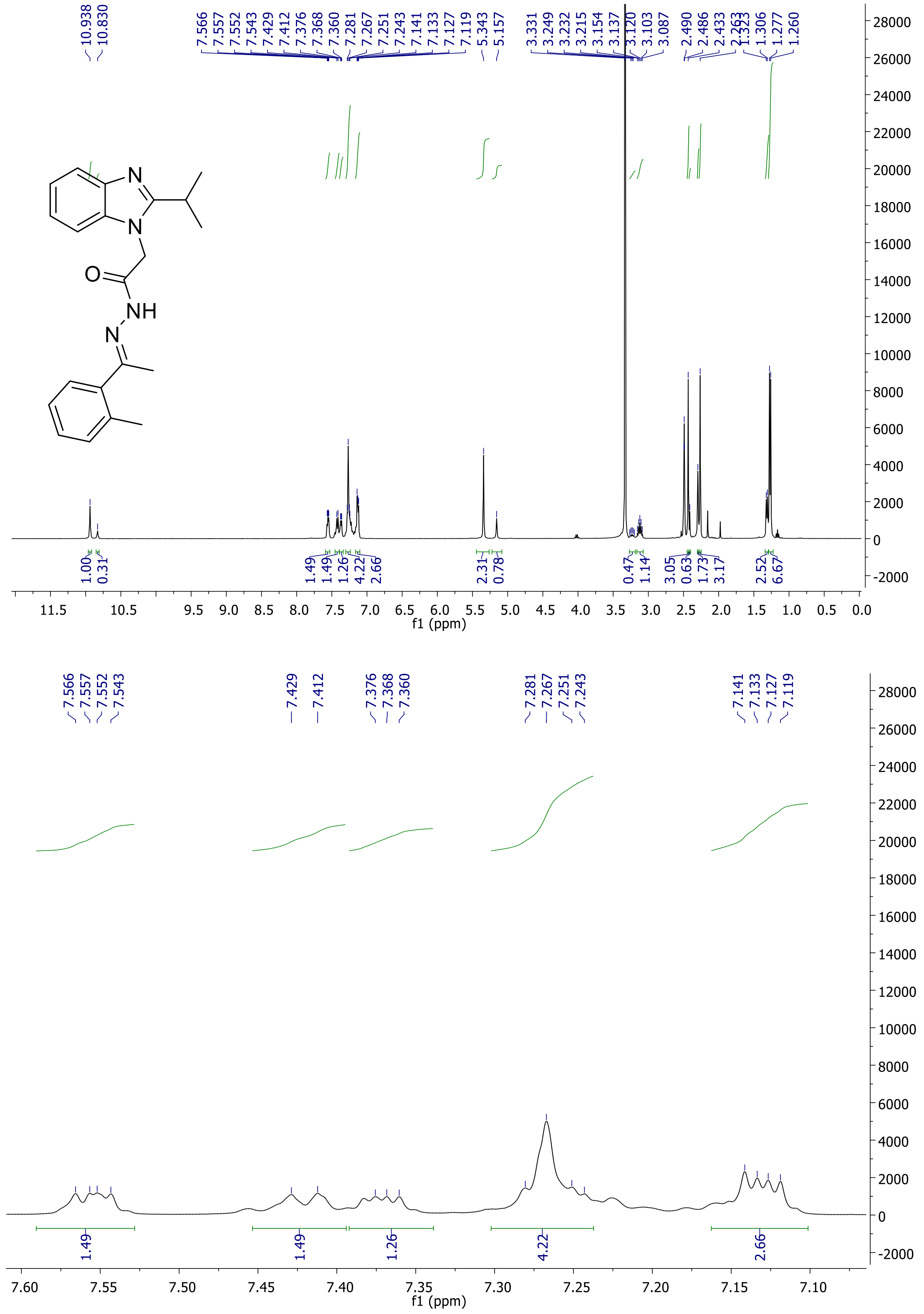

Fig. 23 ^{1}H (400 MHz) NMR spectrum of **14b** in DMSO-d_6

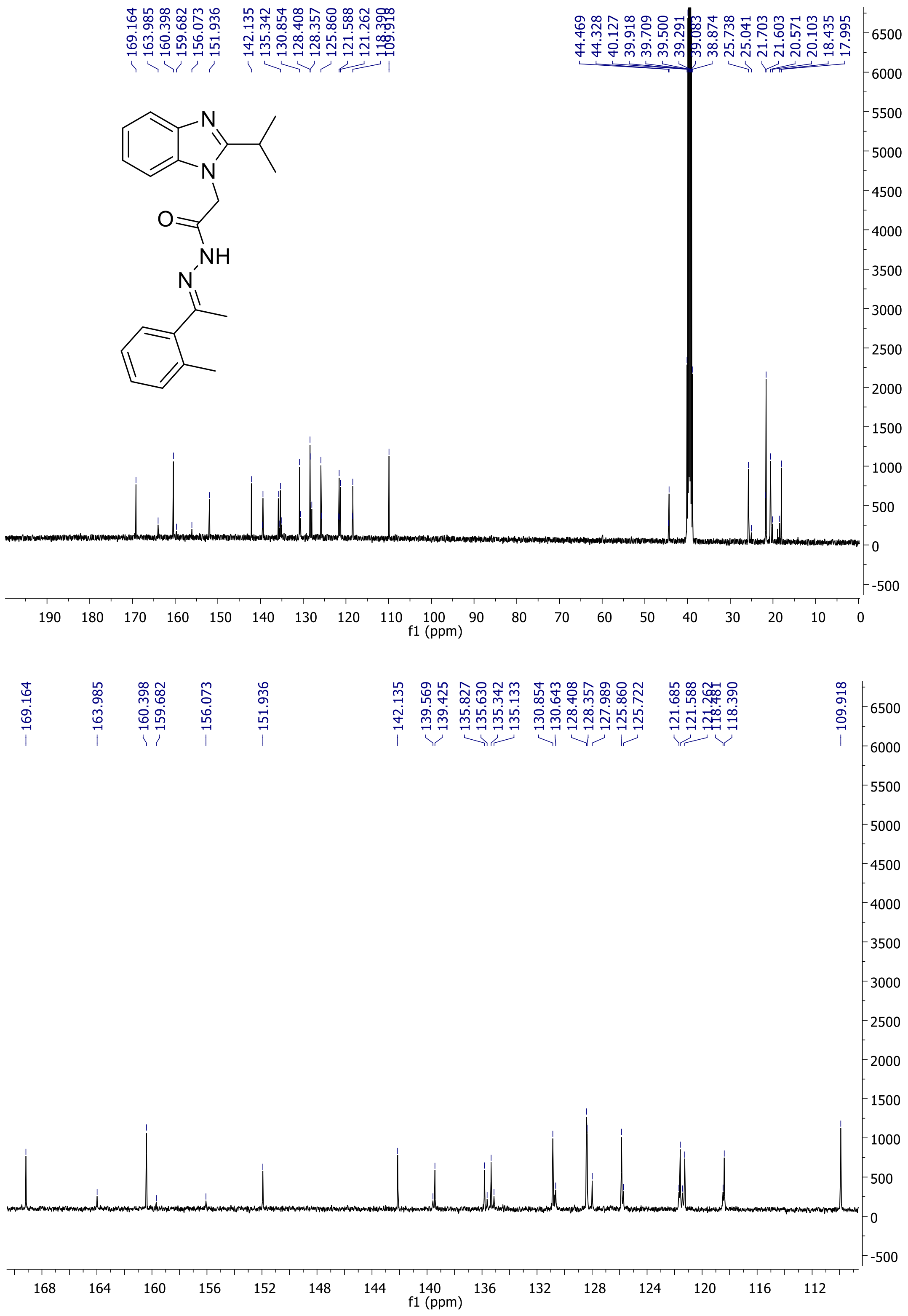

Fig. 24 ^{13}C (100 MHz) NMR spectrum of **14b** in DMSO-d_6

(*E*)-*N*'-(1-(4-bromophenyl)ethylidene)-2-(2-isopropyl-1*H*-benzo[*d*]imidazol-1-yl)acetohydrazide (**14c**)

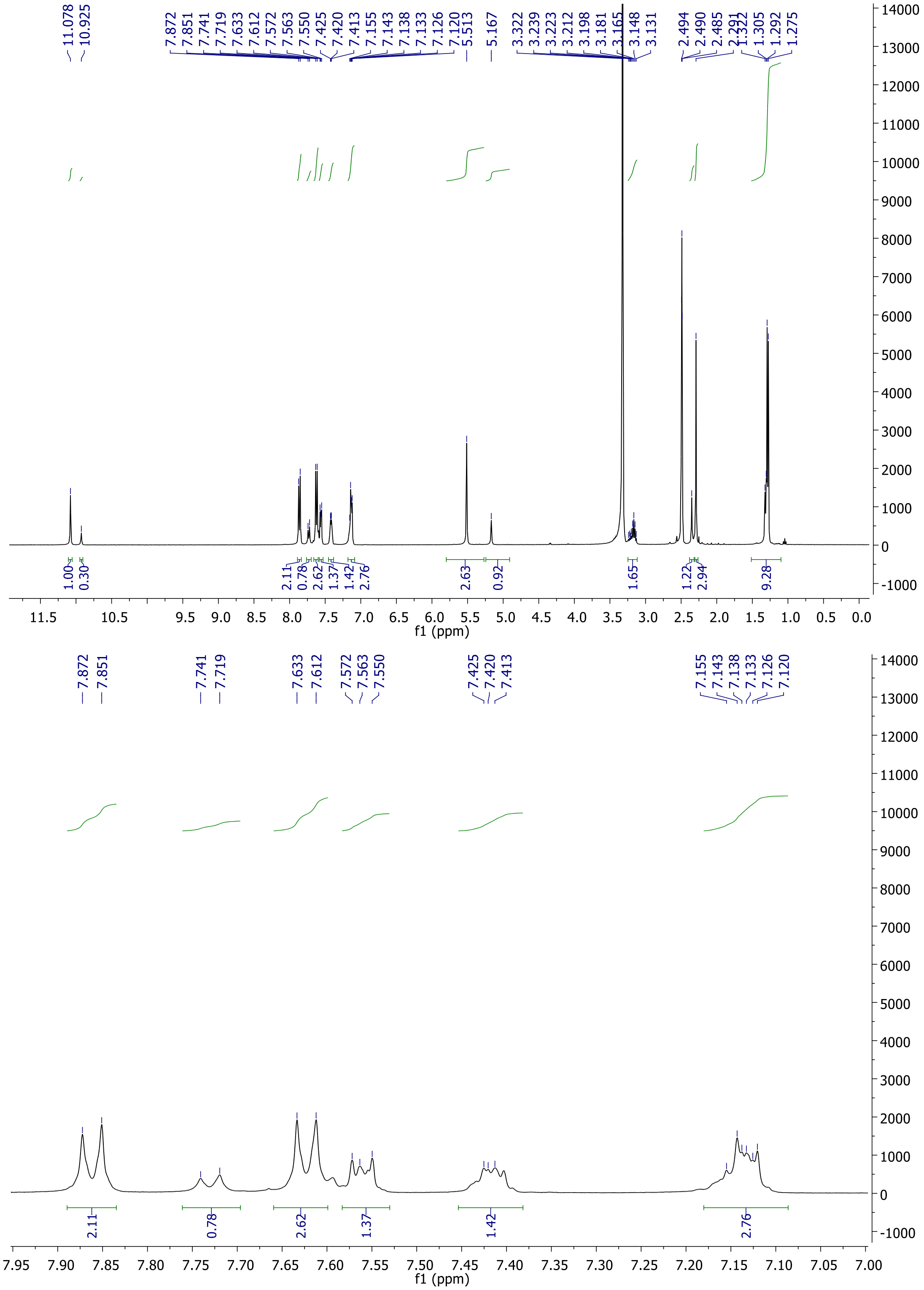

Fig. 25 ^{1}H (400 MHz) NMR spectrum of **14c** in DMSO-d_6

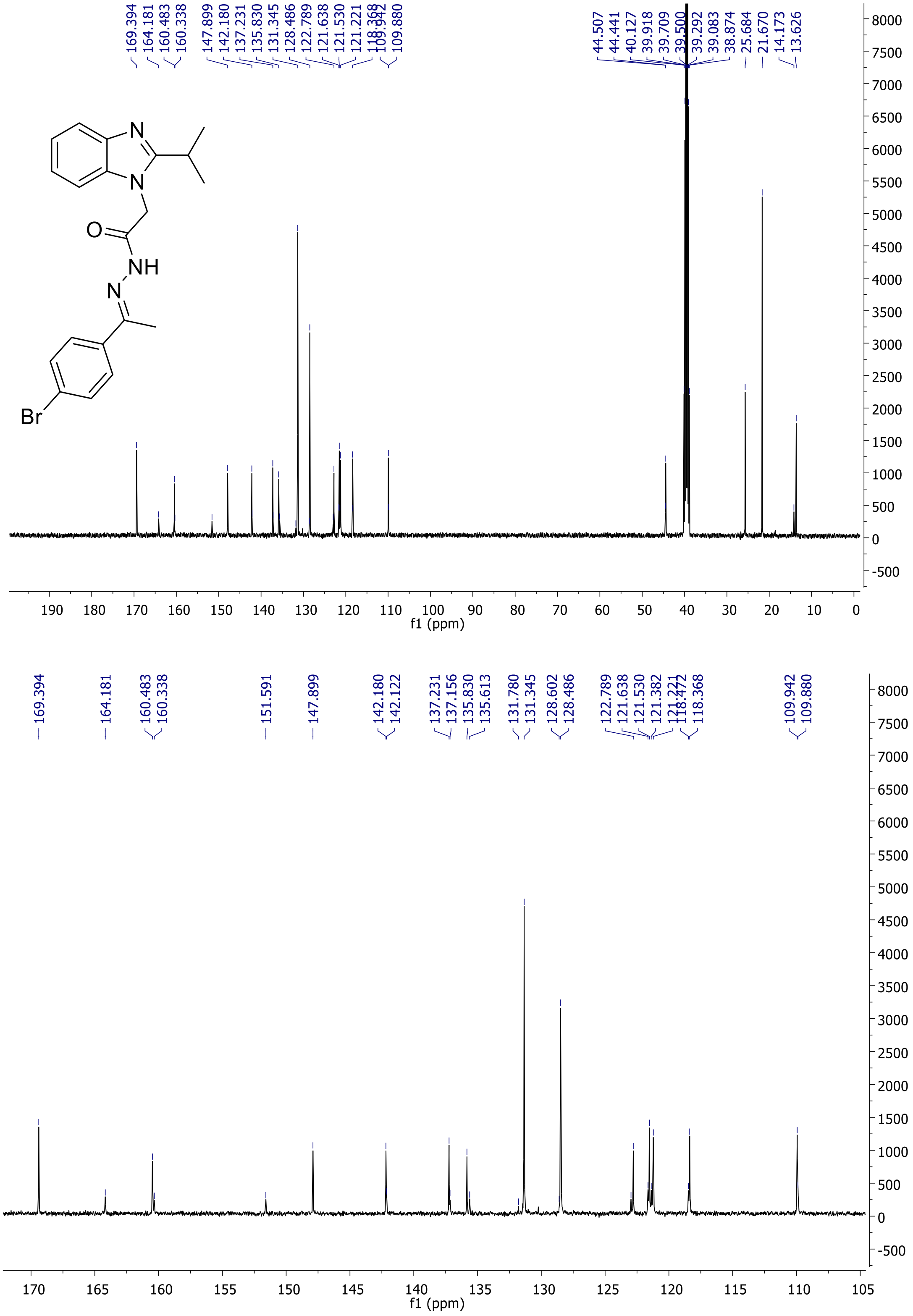

Fig. 26 ^{13}C (100 MHz) NMR spectrum of **14c** in DMSO-d_6

(*E*)-*N*'-(1-(4-bromo-3-nitrophenyl)ethylidene)-2-(2-isopropyl-1*H*-benzo[*d*]imidazol-1-yl)acetohydrazide (**14d**)

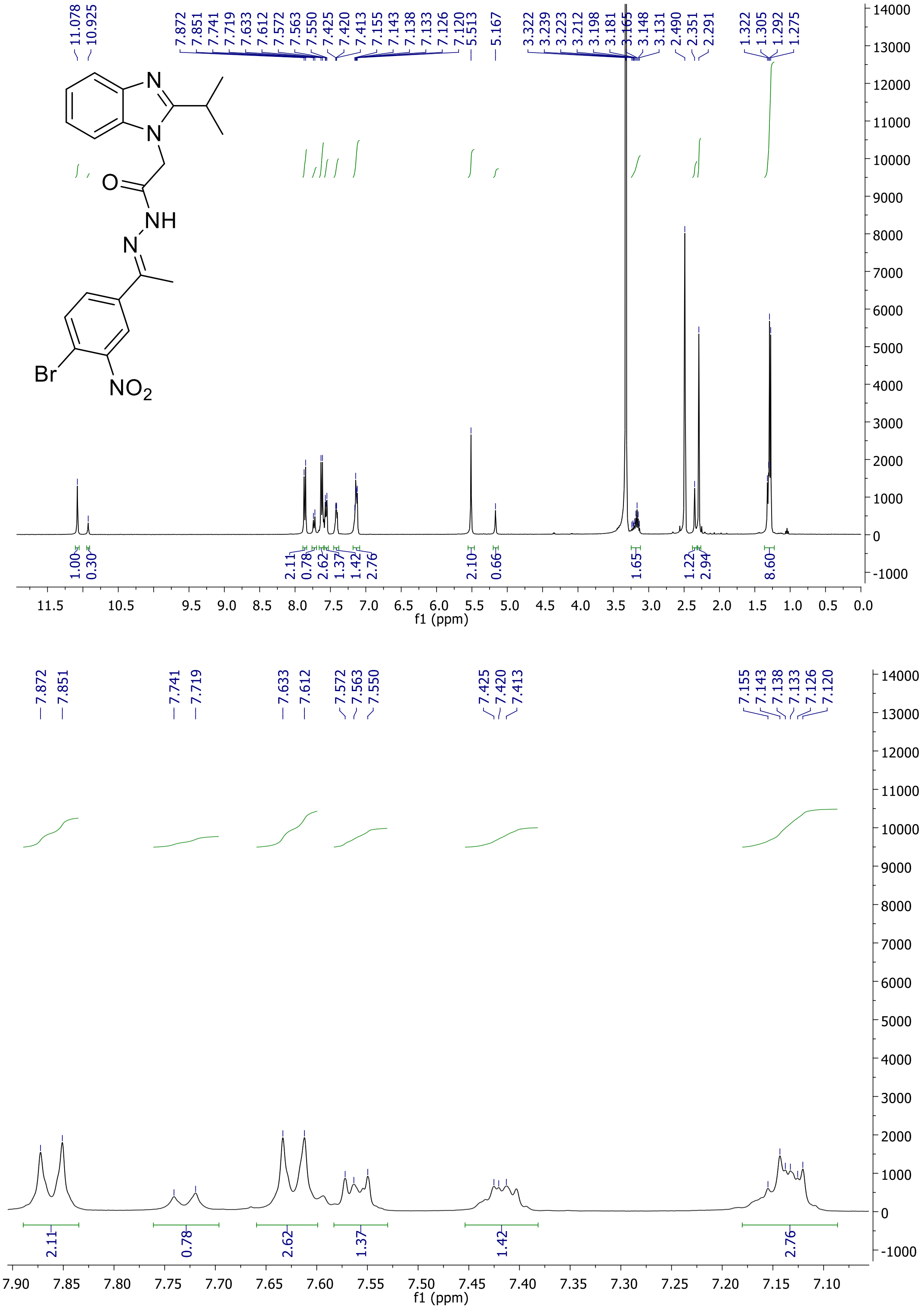

Fig. 27 ^{13}C (100 MHz) NMR spectrum of **14d** in DMSO-d_6

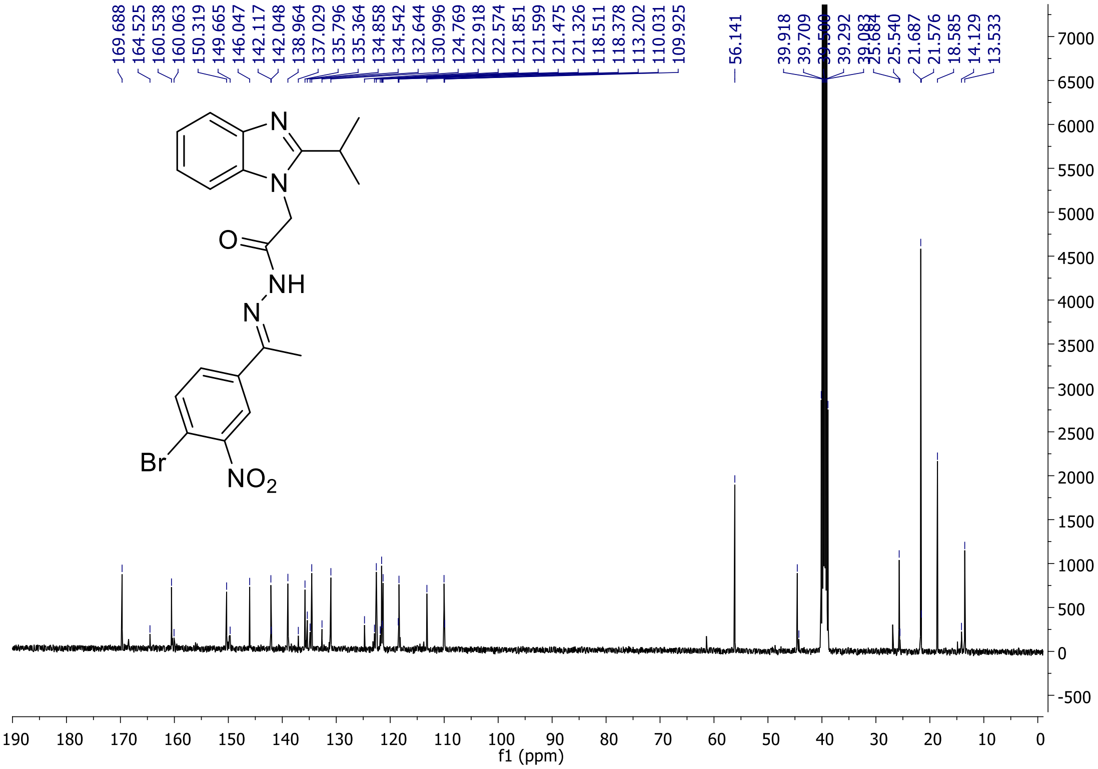

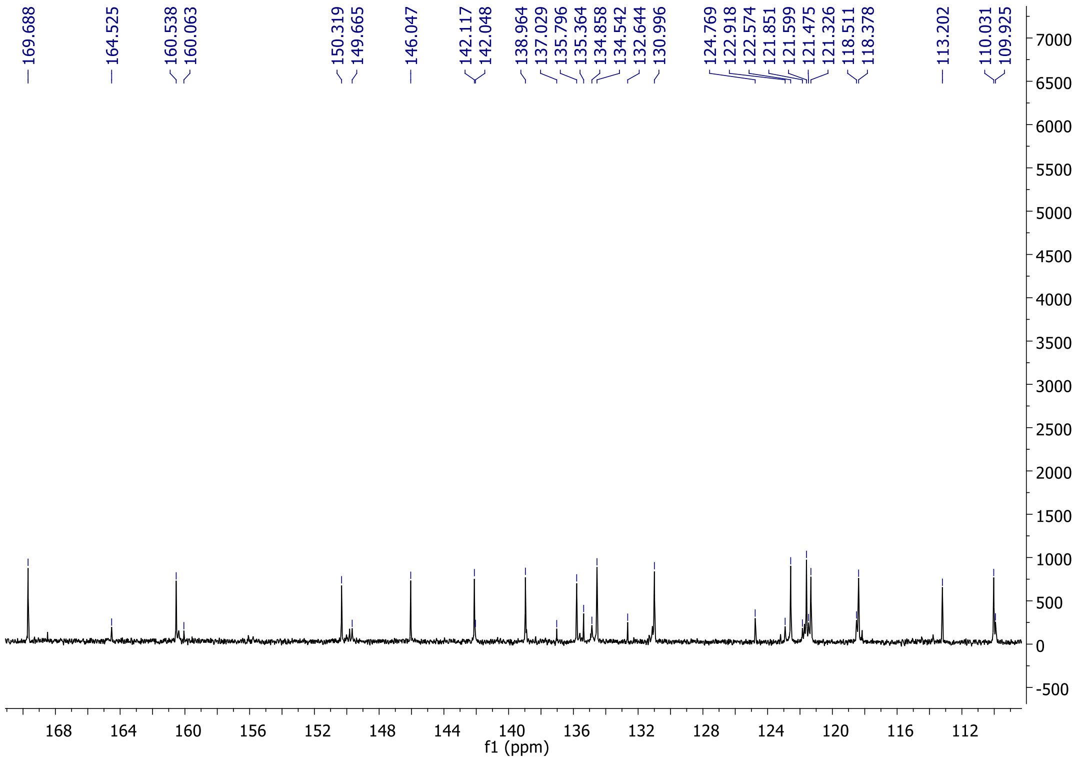

Fig. 28 ^{13}C (100 MHz) NMR spectrum of **14d** in DMSO-d_6

(*E*)-*N*'-(3-(Benzyloxy)benzylidene)-2-(2-isopropyl-1*H*-benzo[*d*]imidazol-1-yl)acetohydrazide (**17a**)

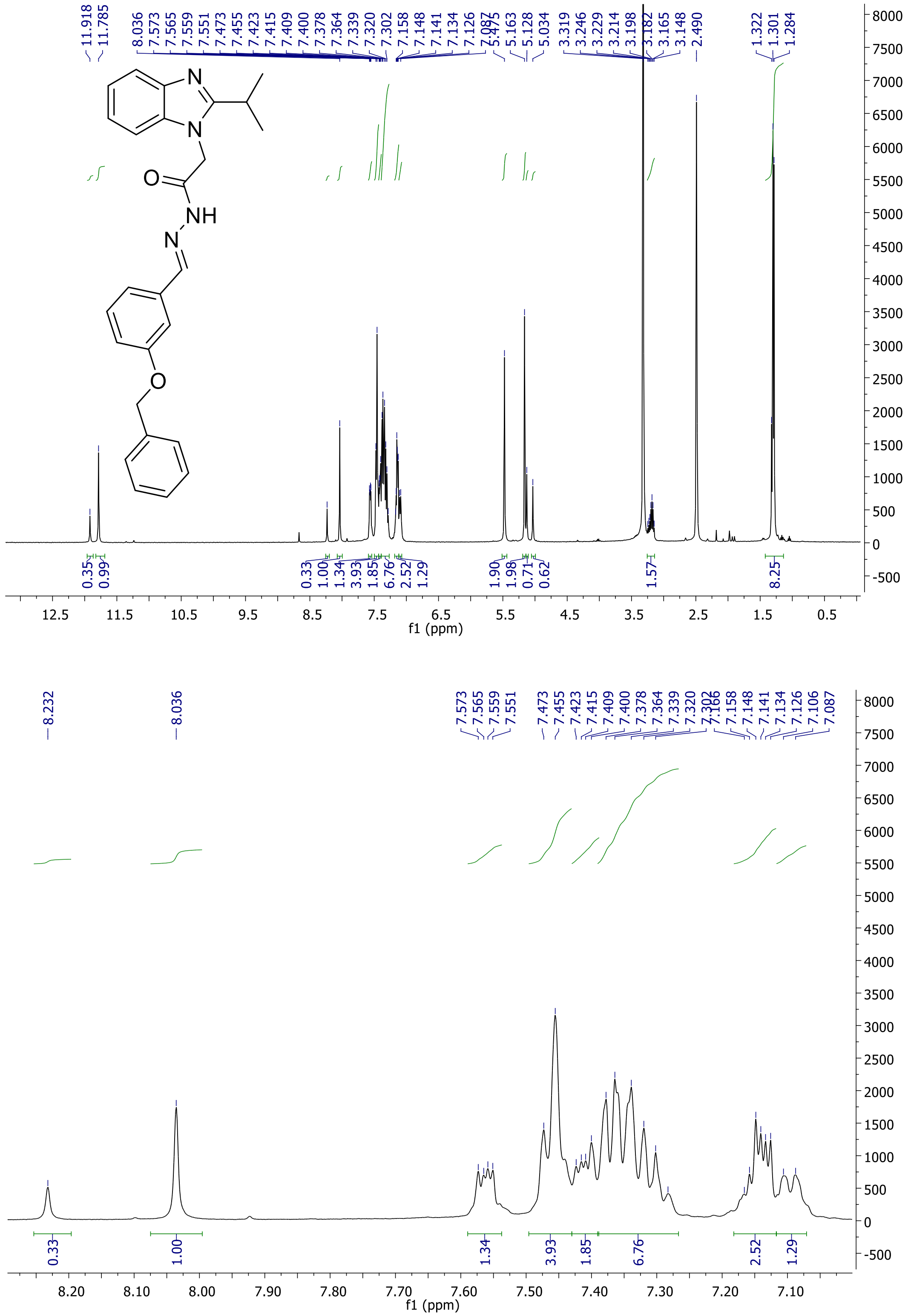

Fig. 29 ^{1}H (400 MHz) NMR spectrum of **17a** in DMSO-d_6

(*E*)-*N*'-(3-(benzyloxy)benzylidene)-2-(2-(5-methylfuran-2-yl)-1*H*-benzo[*d*]imidazol-1-yl)acetohydrazide (**17b**)

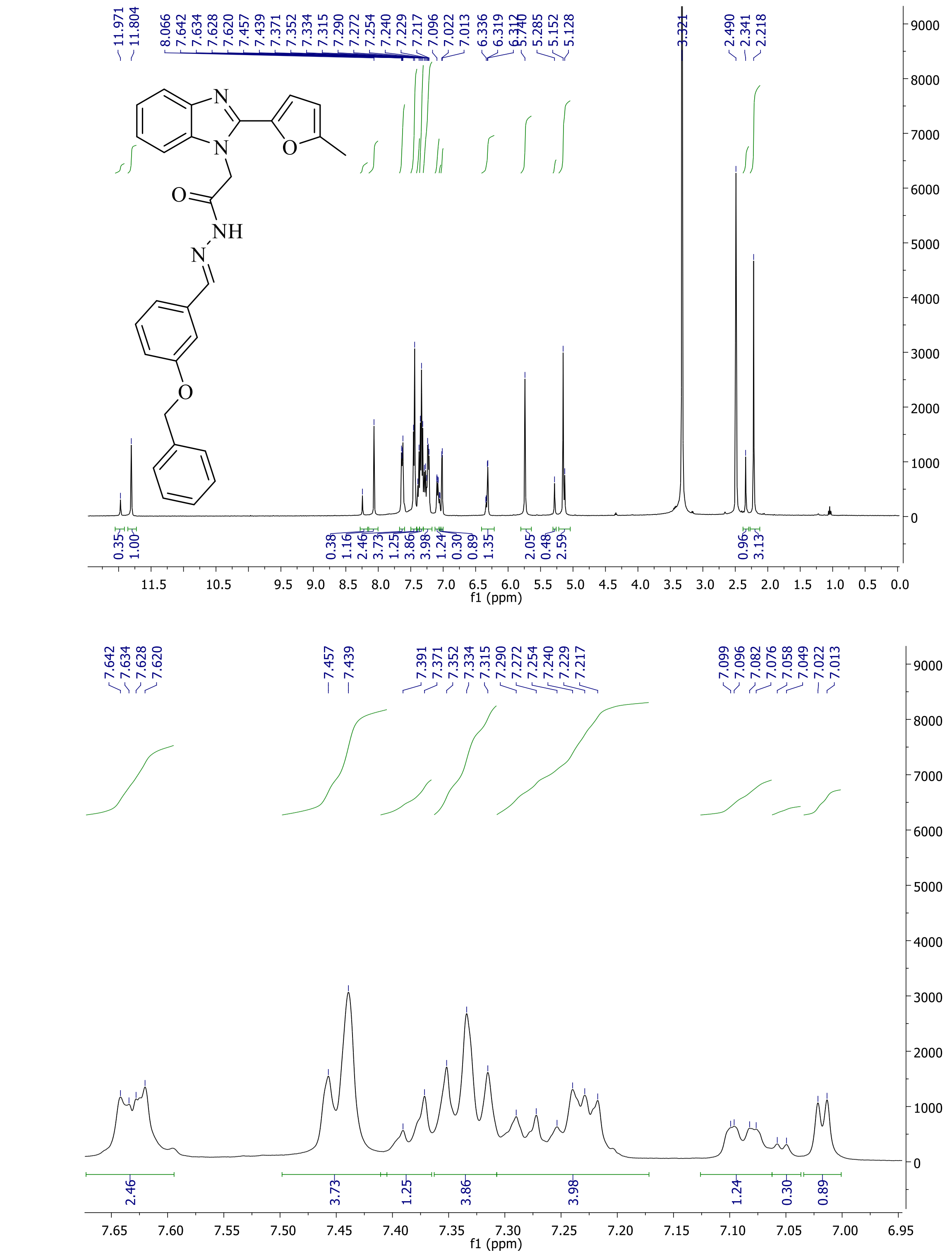

Fig. 30 ^{1}H (400 MHz) NMR spectrum of **17b** in DMSO-d_6

(*E*)-*N*'-(4-(benzyloxy)benzylidene)-2-(2-isopropyl-1*H*-benzo[*d*]imidazol-1-yl)acetohydrazide (**17c**)

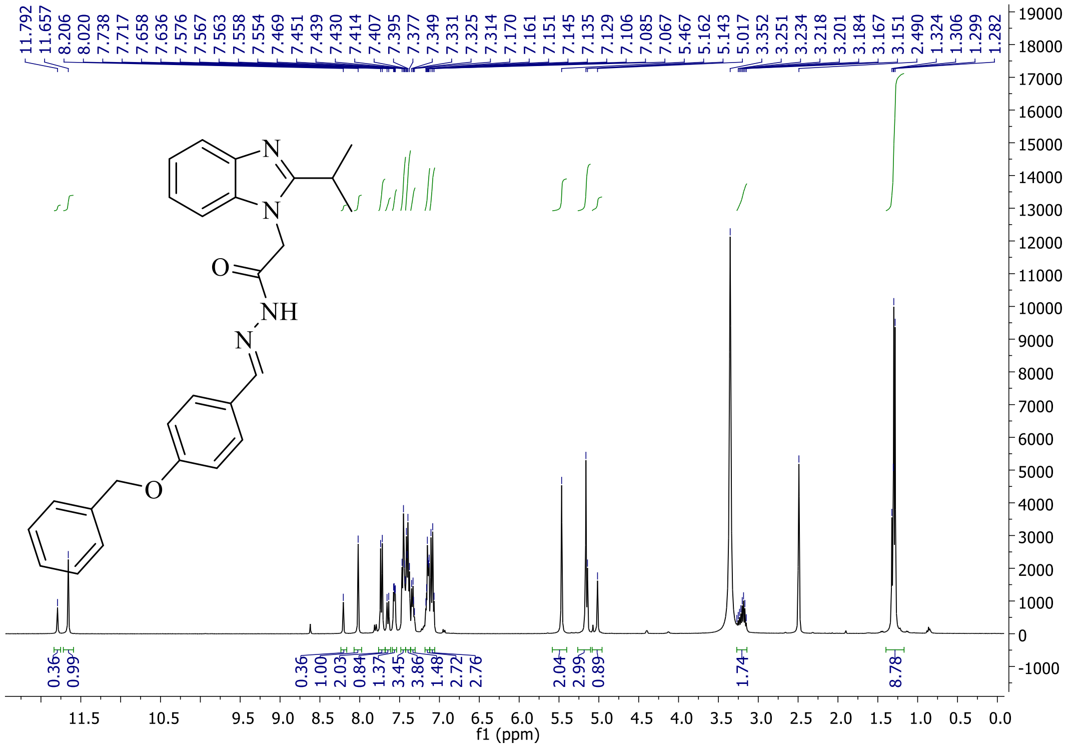

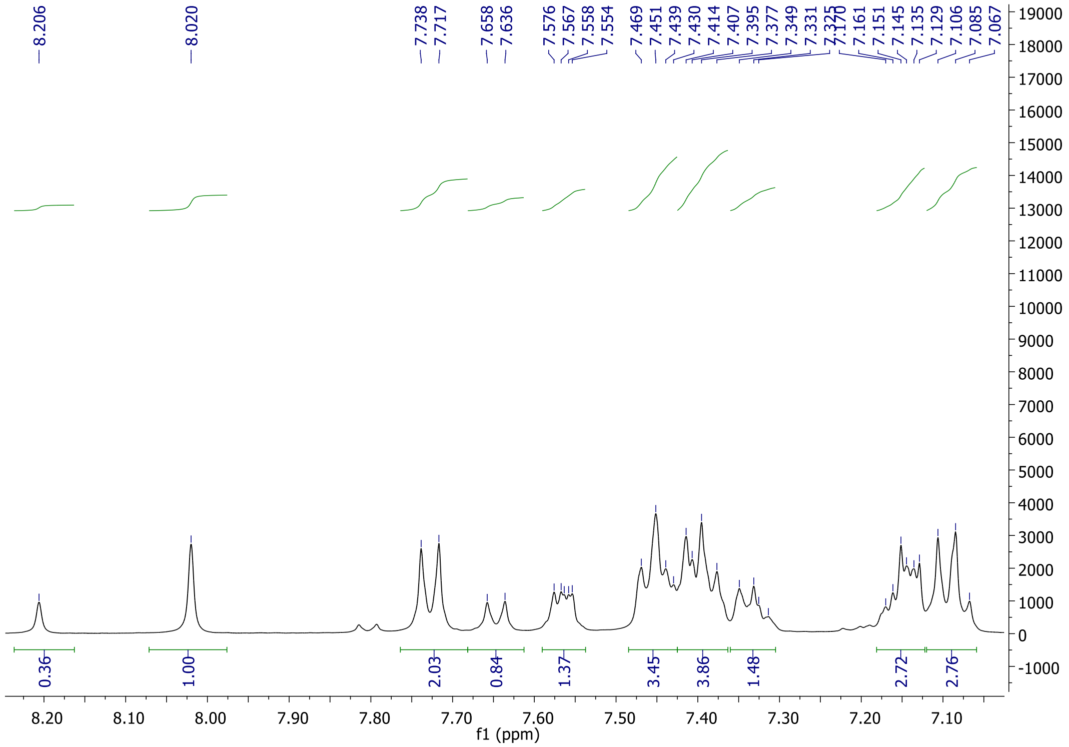

Fig. 31 ^{13}C (100 MHz) NMR spectrum of **17c** in DMSO-d_6

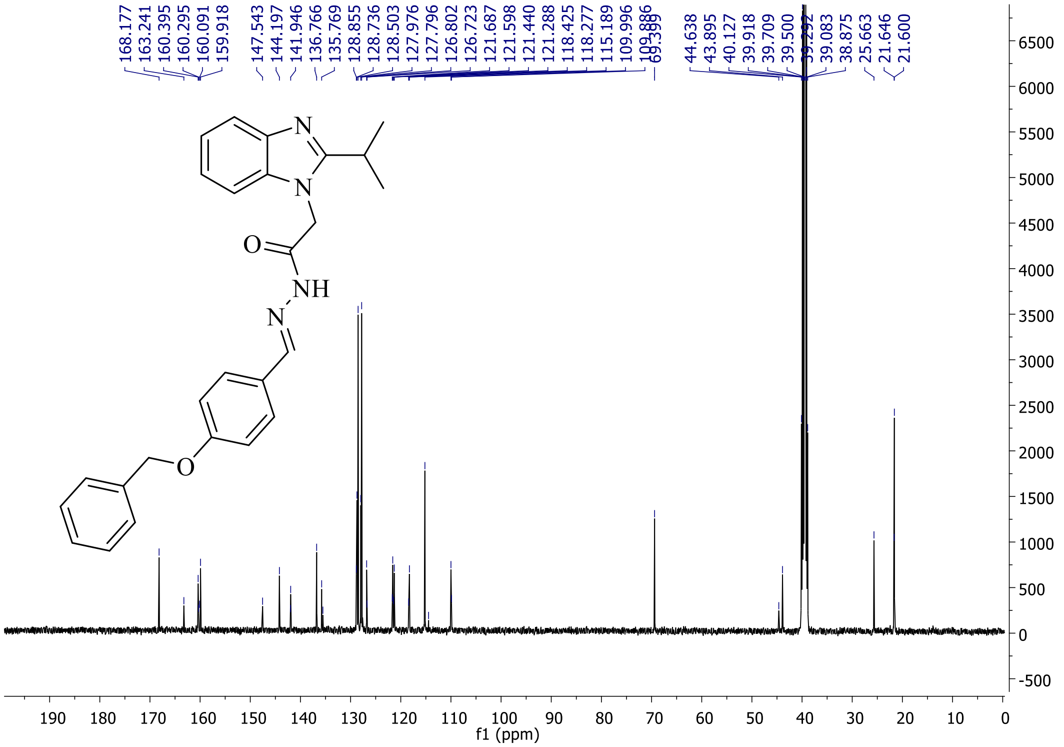

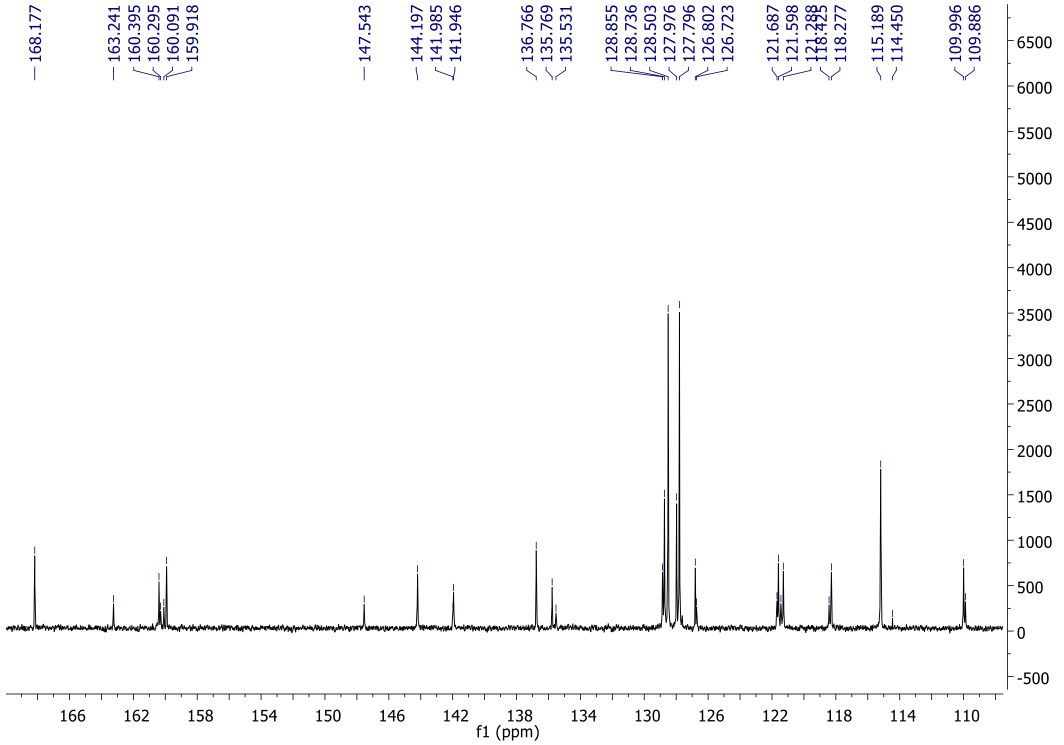

Fig. 32 ^{13}C (100 MHz) NMR spectrum of **17c** in DMSO-d_6

(*E*)-*N*'-(4-(benzyloxy)benzylidene)-2-(2-(5-methylfuran-2-yl)-1*H*-benzo[*d*]imidazol-1-yl)acetohydrazide (**17d**)

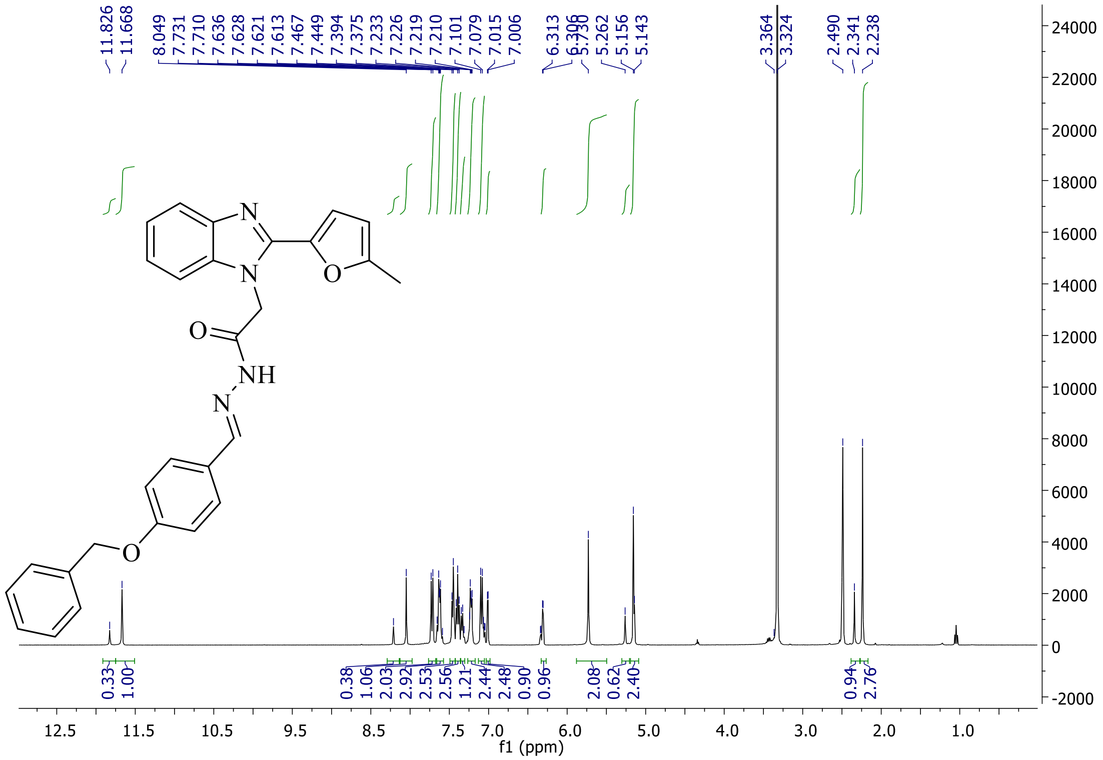

Fig. 33 ^{1}H (400 MHz) NMR spectrum of **17d** in DMSO-d_6

(*E*)-*N*'-(4-(benzyloxy)-3-methoxybenzylidene)-2-(2-isopropyl-1*H*-benzo[*d*]imidazol-1-yl)acetohydrazide (**17e**)

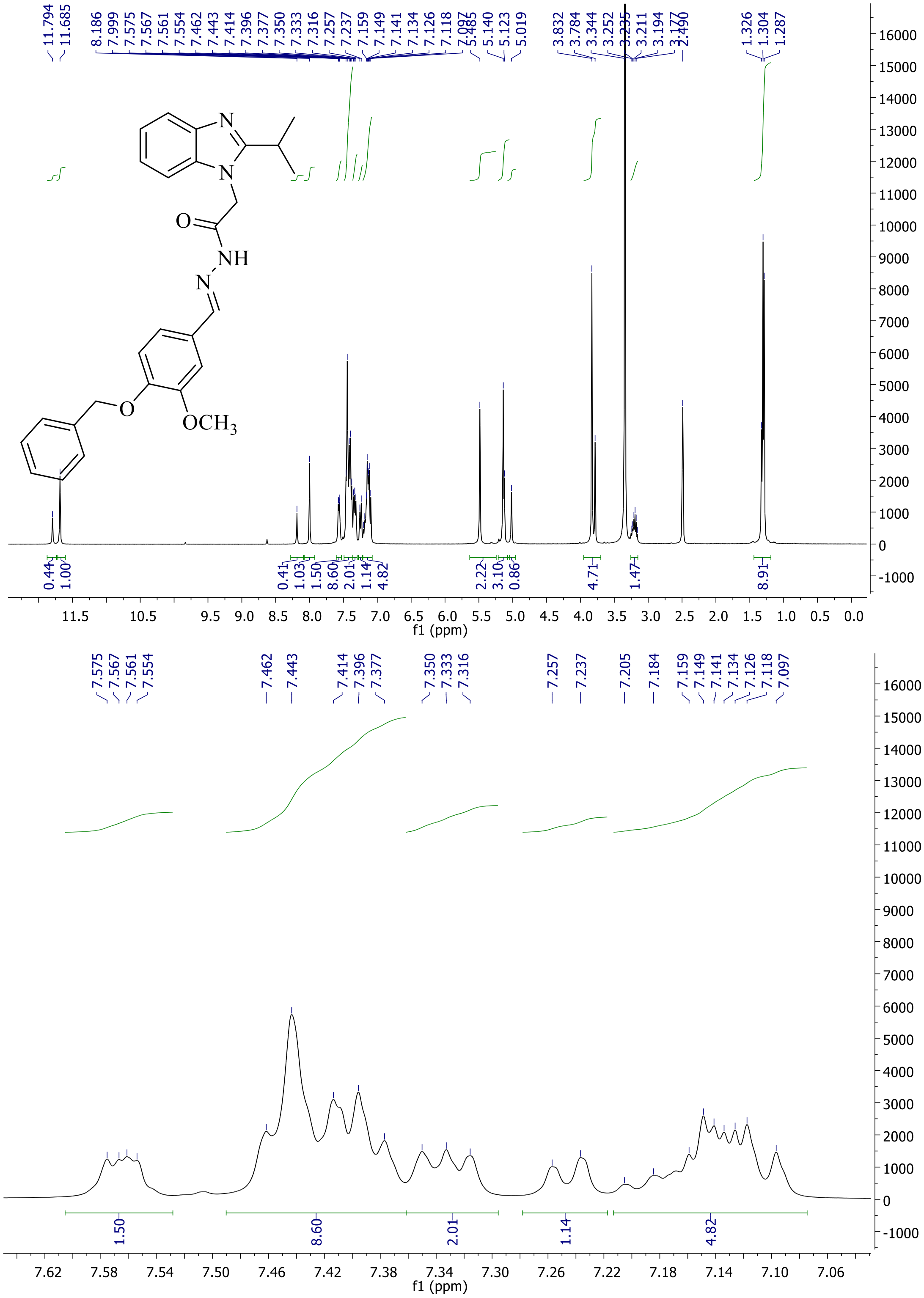

Fig. 34 ^{1}H (400 MHz) NMR spectrum of **17e** in DMSO-d_6

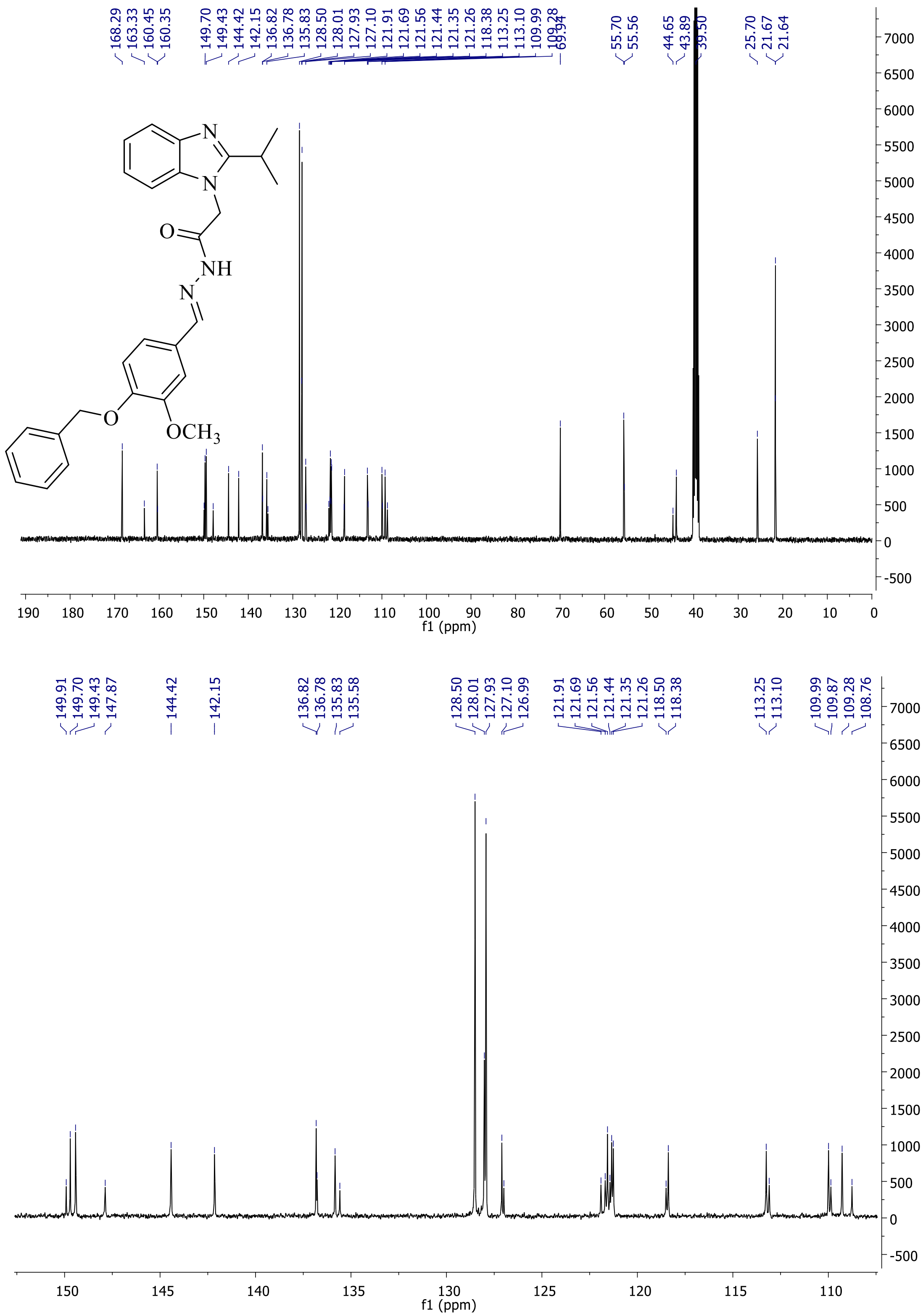

Fig. 35 ^{13}C (100 MHz) NMR spectrum of **17e** in DMSO-d_6

(E)-N'-(4-(benzyloxy)-3-methoxybenzylidene)-2-(2-(5-methylfuran-2-yl)-1H-benzo[d]imidazol-1-yl)acetohydrazide (**17f**)

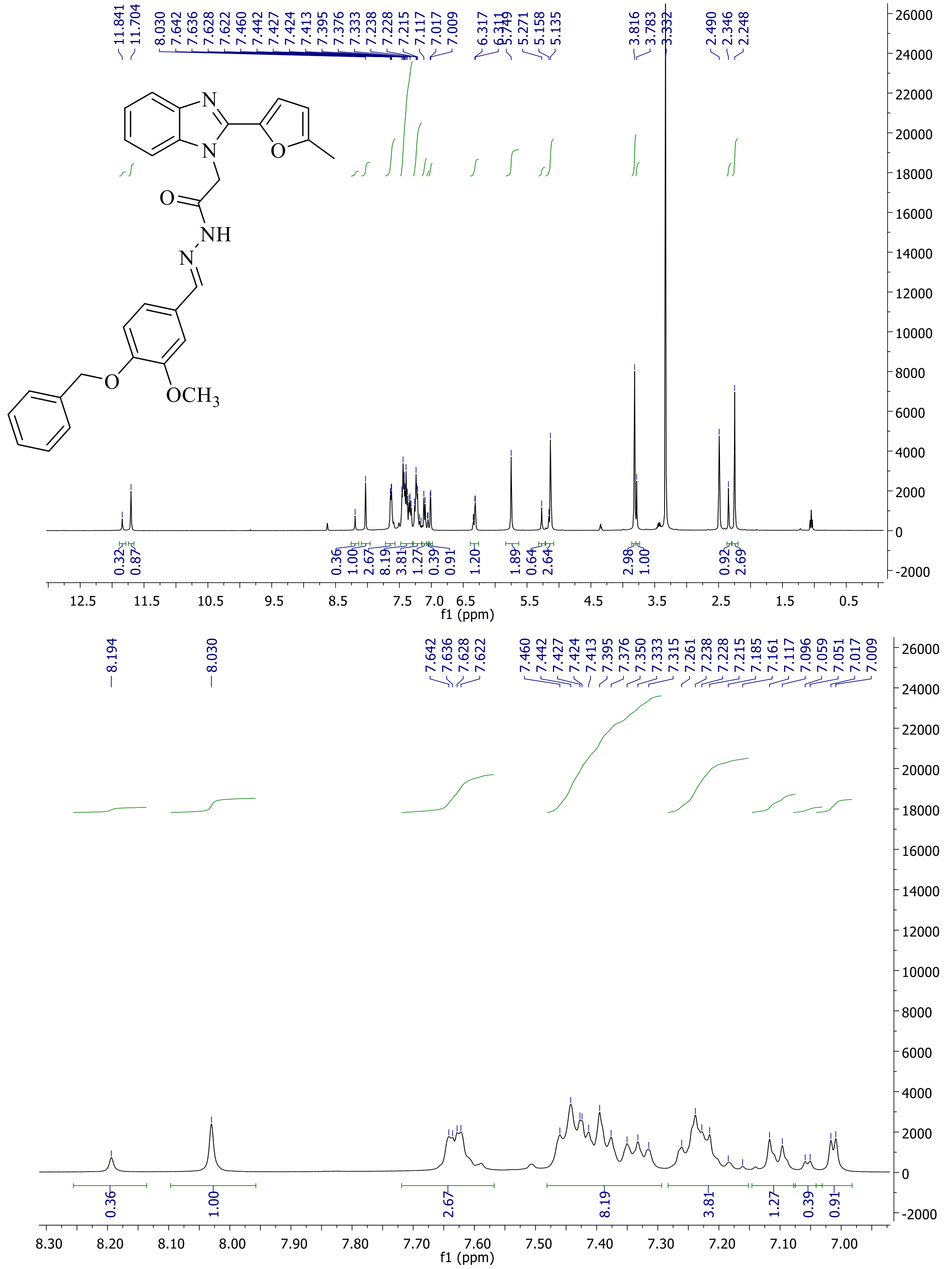

Fig. 36 ^{1}H (400 MHz) NMR spectrum of **17f** in DMSO-d_6

Methyl-(*E*)-2-(4-((2-(2-(2-isopropyl-1*H*-benzo[*d*]imidazol-1-yl)acetyl)hydrazono)methyl)-2-methoxyphenoxy)acetate (**19a**)

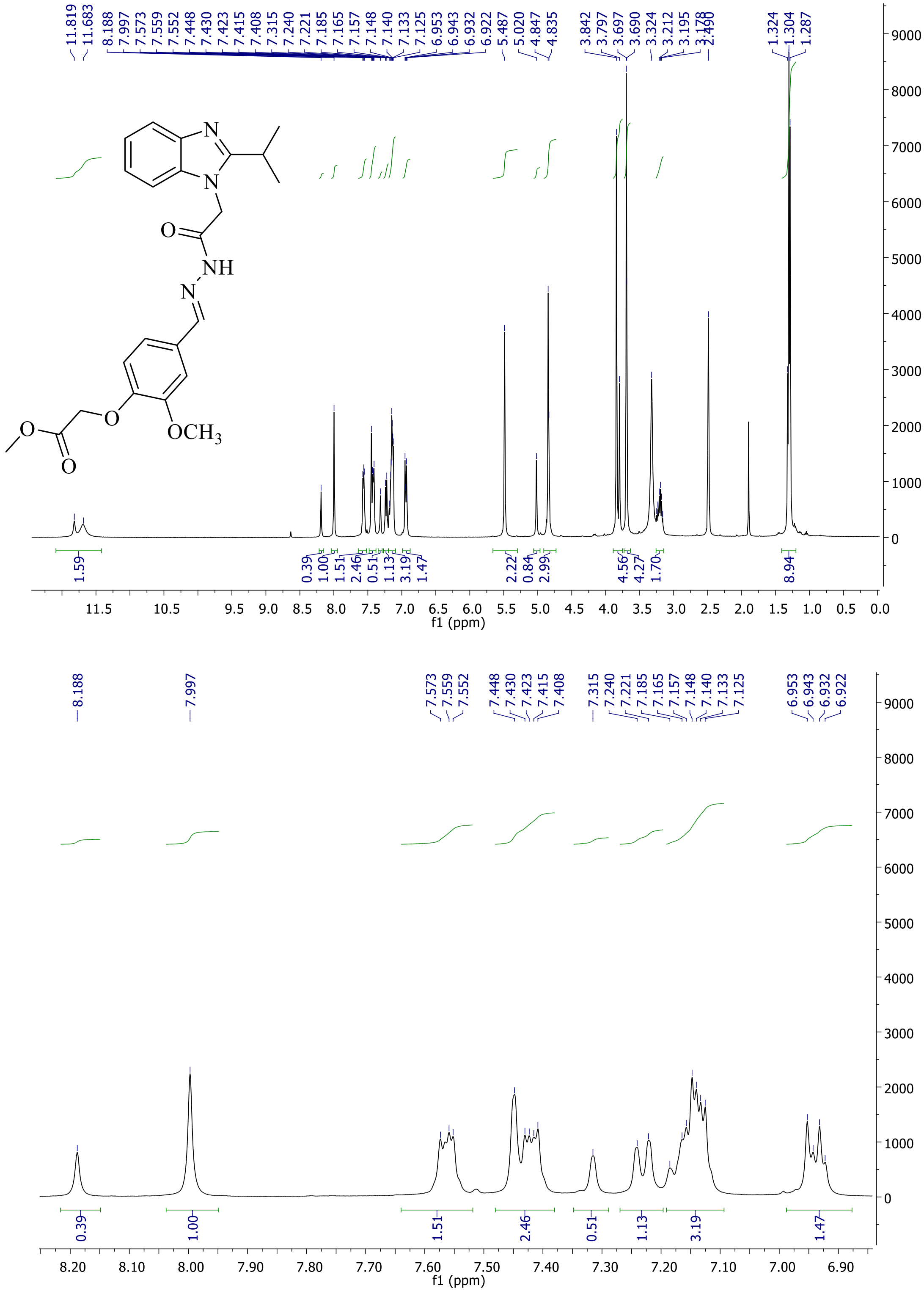

Fig. 37 ^{1}H (400 MHz) NMR spectrum of **19a** in DMSO-d_6

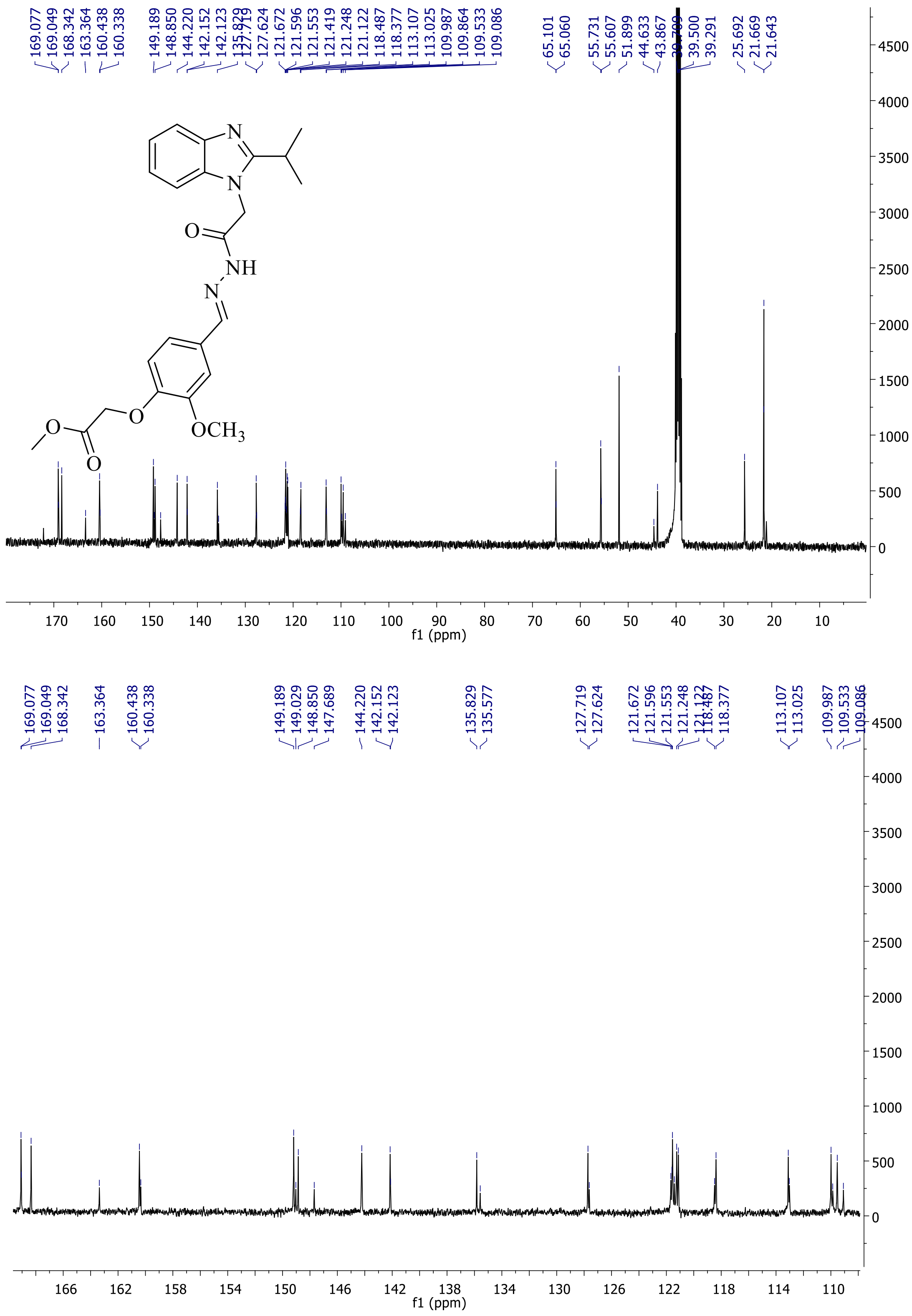

Fig. 38 ^{13}C (100 MHz) NMR spectrum of **19a** in DMSO-d_6

Methyl-(*E*)-2-(2-methoxy-4-((2-(2-(2-(5-methylfuran-2-yl)-1H-benzo[d]imidazol-1-yl)acetyl)hydrazono)methyl)phenoxy)acetate (**19b**)

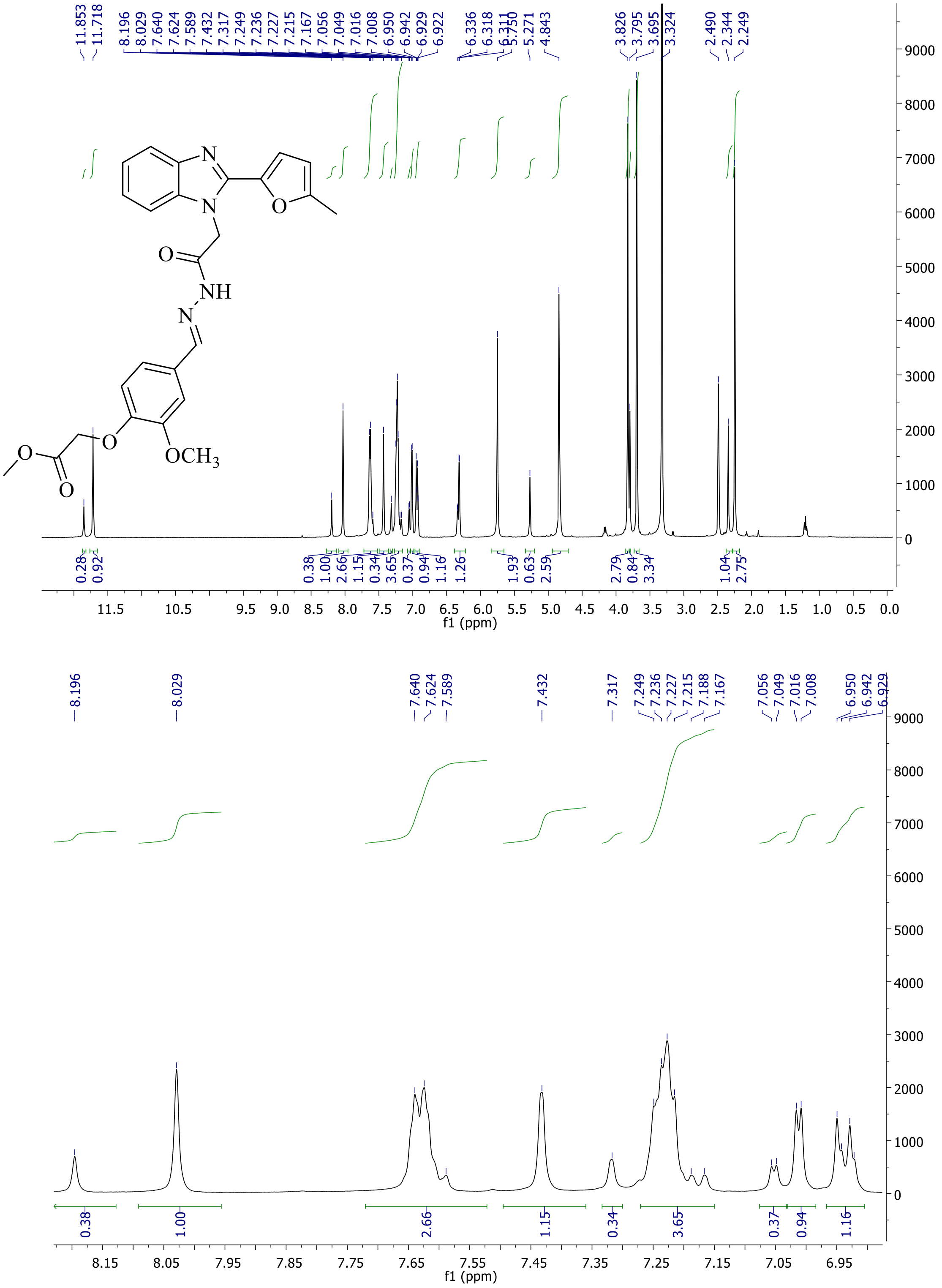

Fig. 39 ^{1}H (100 MHz) NMR spectrum of **19b** in DMSO-d_6

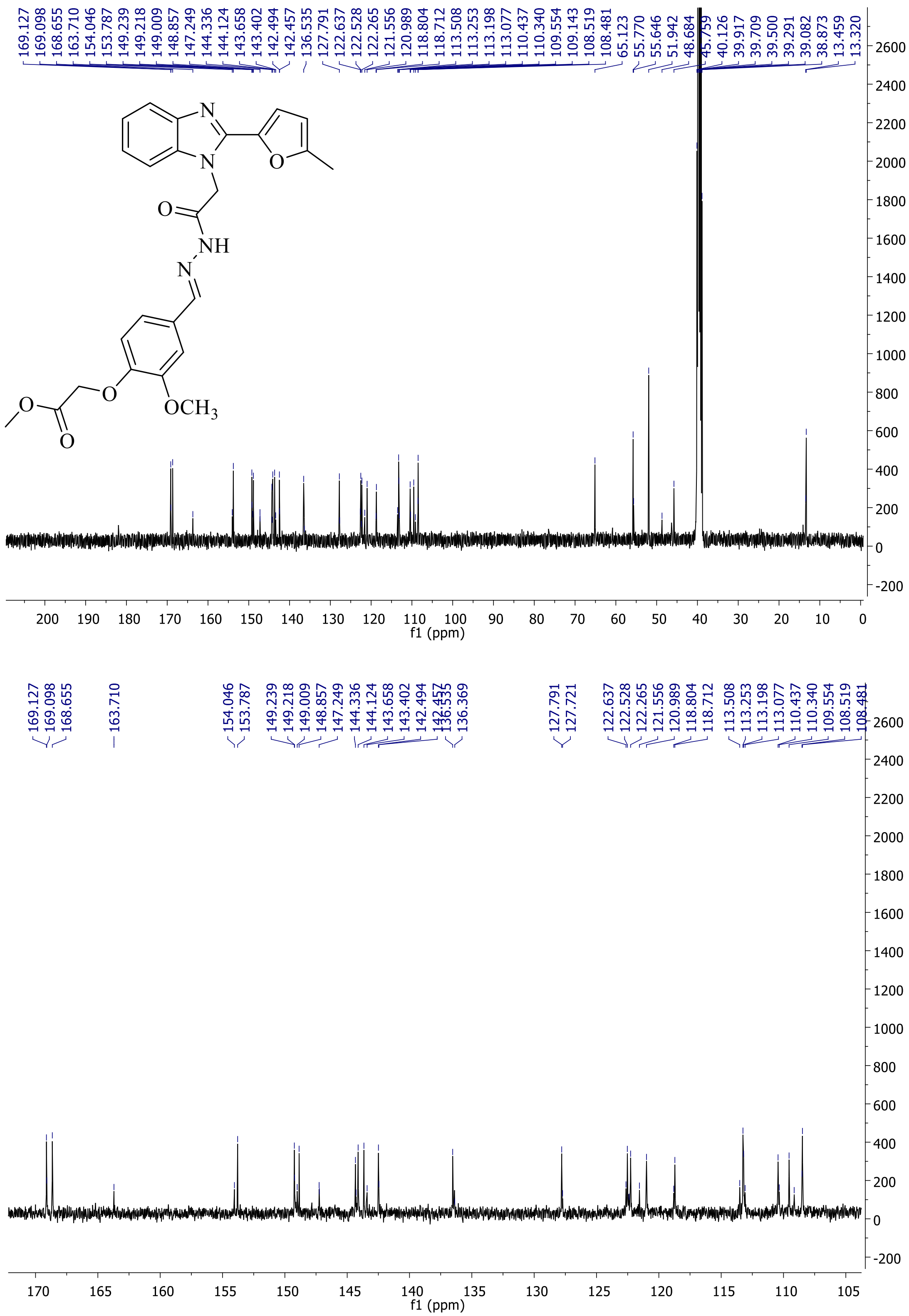

Fig. 40 ^{13}C (100 MHz) NMR spectrum of **19b** in DMSO-d_6

Ethyl-(*E*)-2-(4-((2-(2-(2-isopropyl-1*H*-benzo[*d*]imidazol-1-yl)acetyl)hydrazono)methyl)-2-methoxyphenoxy)acetate (**19c**)

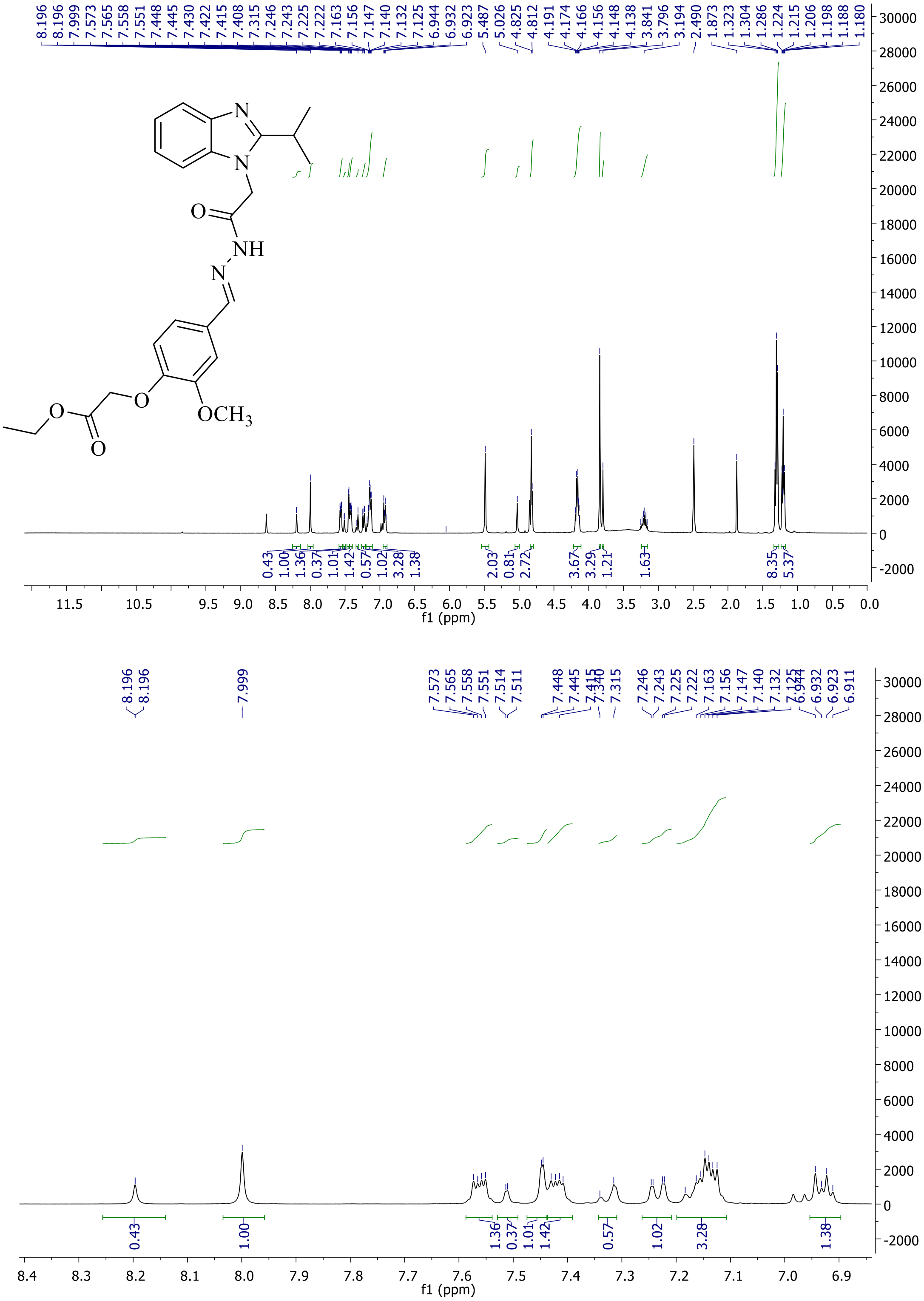

Fig. 41 ^{1}H (400 MHz) NMR spectrum of **19c** in DMSO-d_6

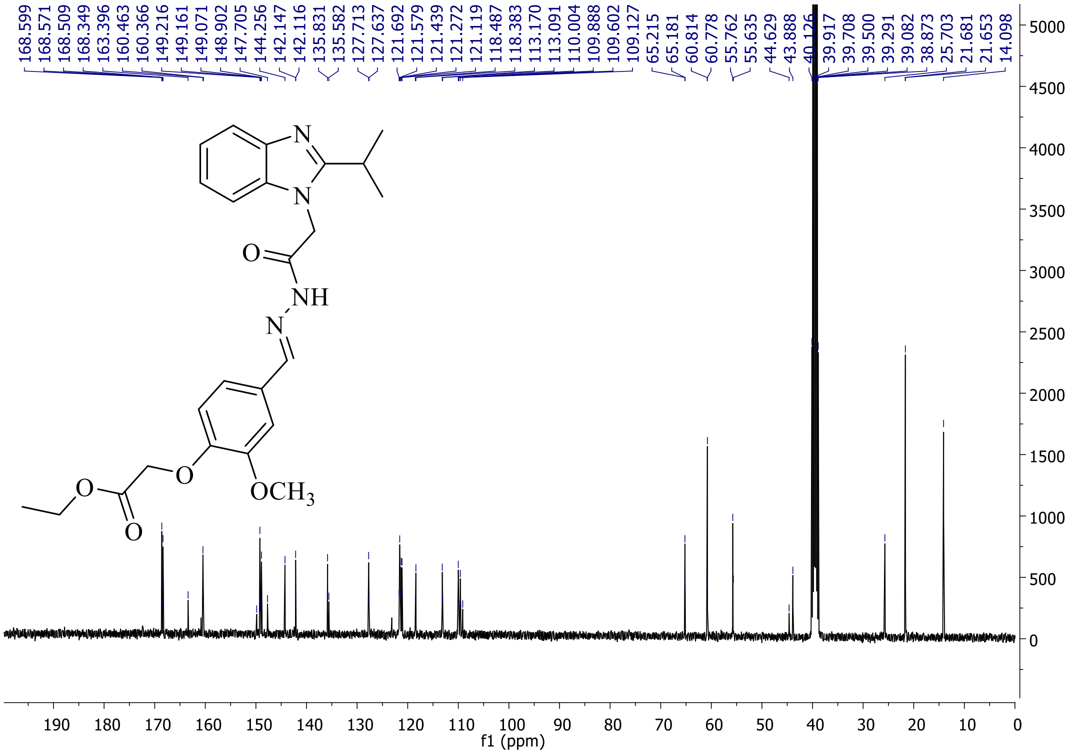

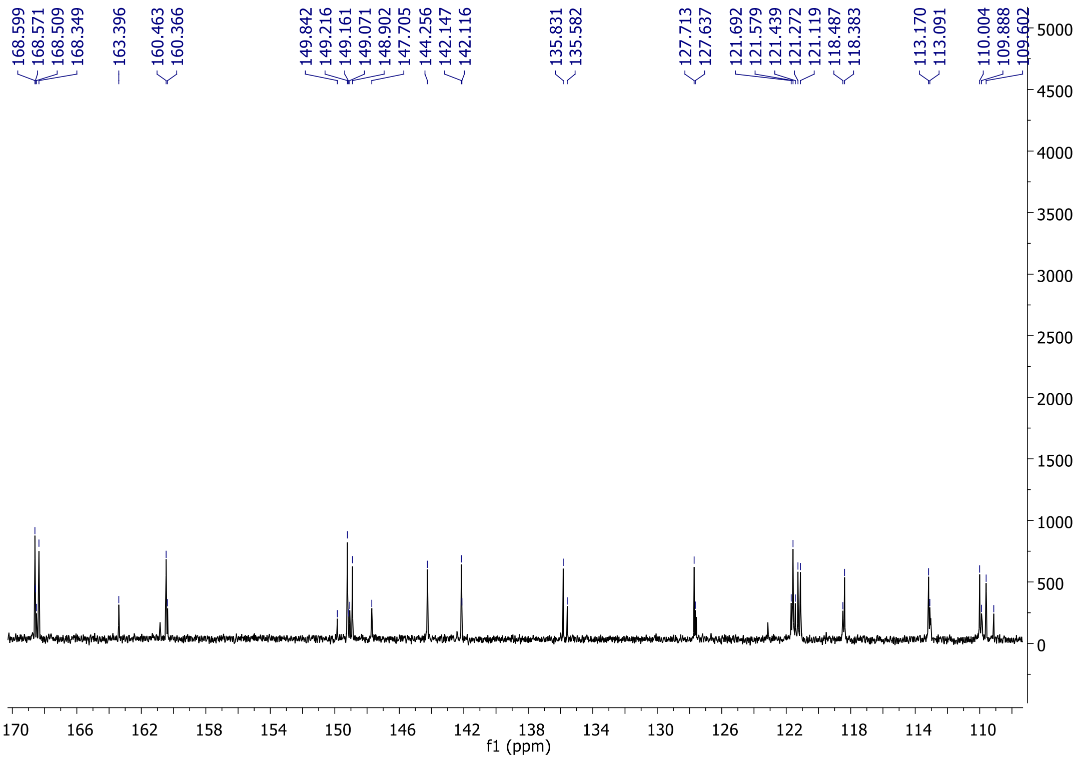

Fig. 42 ^{13}C (100 MHz) NMR spectrum of **19c** in DMSO-d_6

Ethyl-(*E*)-2-(4-((2-(2-(2-isopropyl-1*H*-benzo[*d*]imidazol-1-yl)acetyl)hydrazono)methyl)-2-methoxyphenoxy)acetate (**19d**)

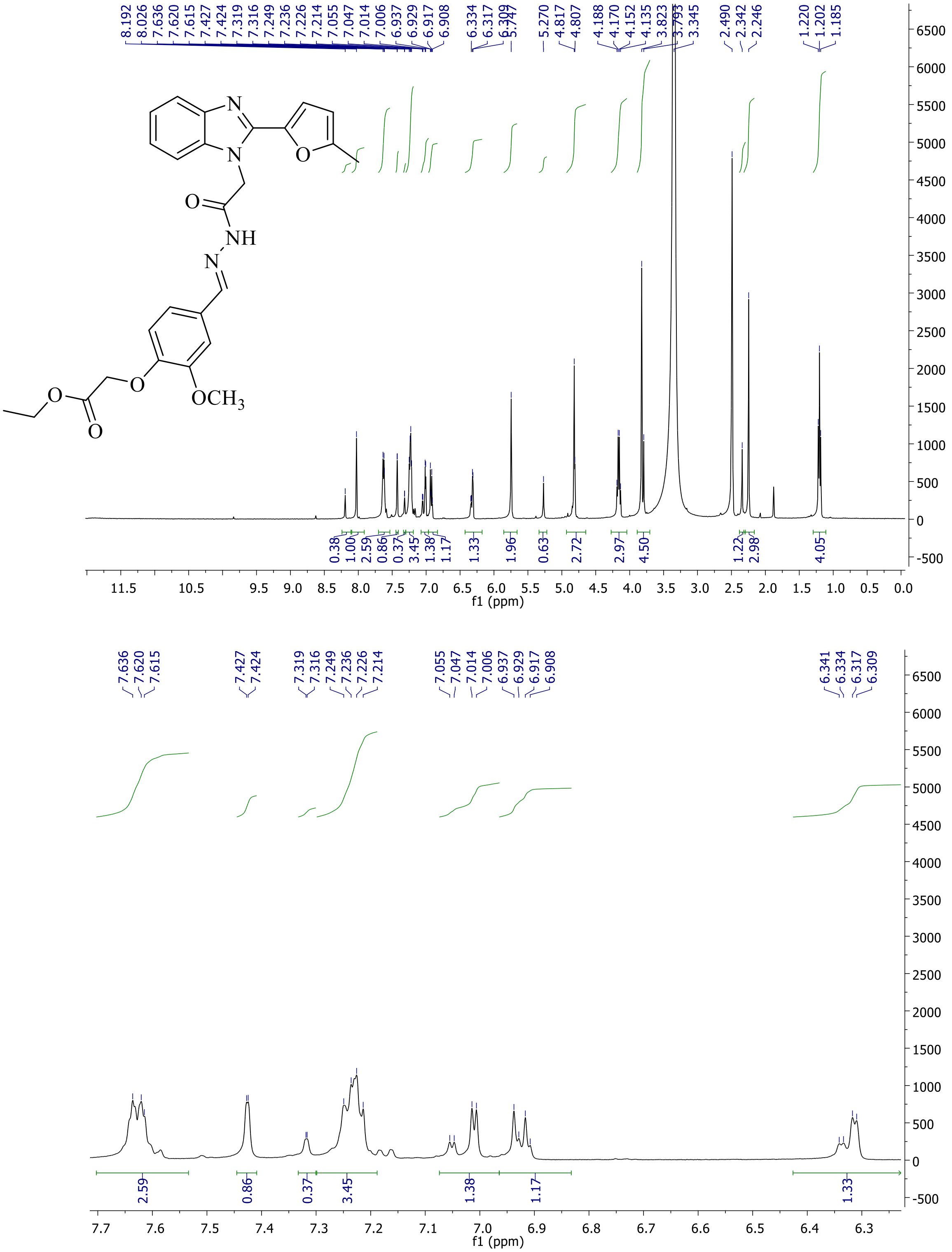

Fig. 43 ^{1}H (400 MHz) NMR spectrum of **19d** in DMSO-d_6

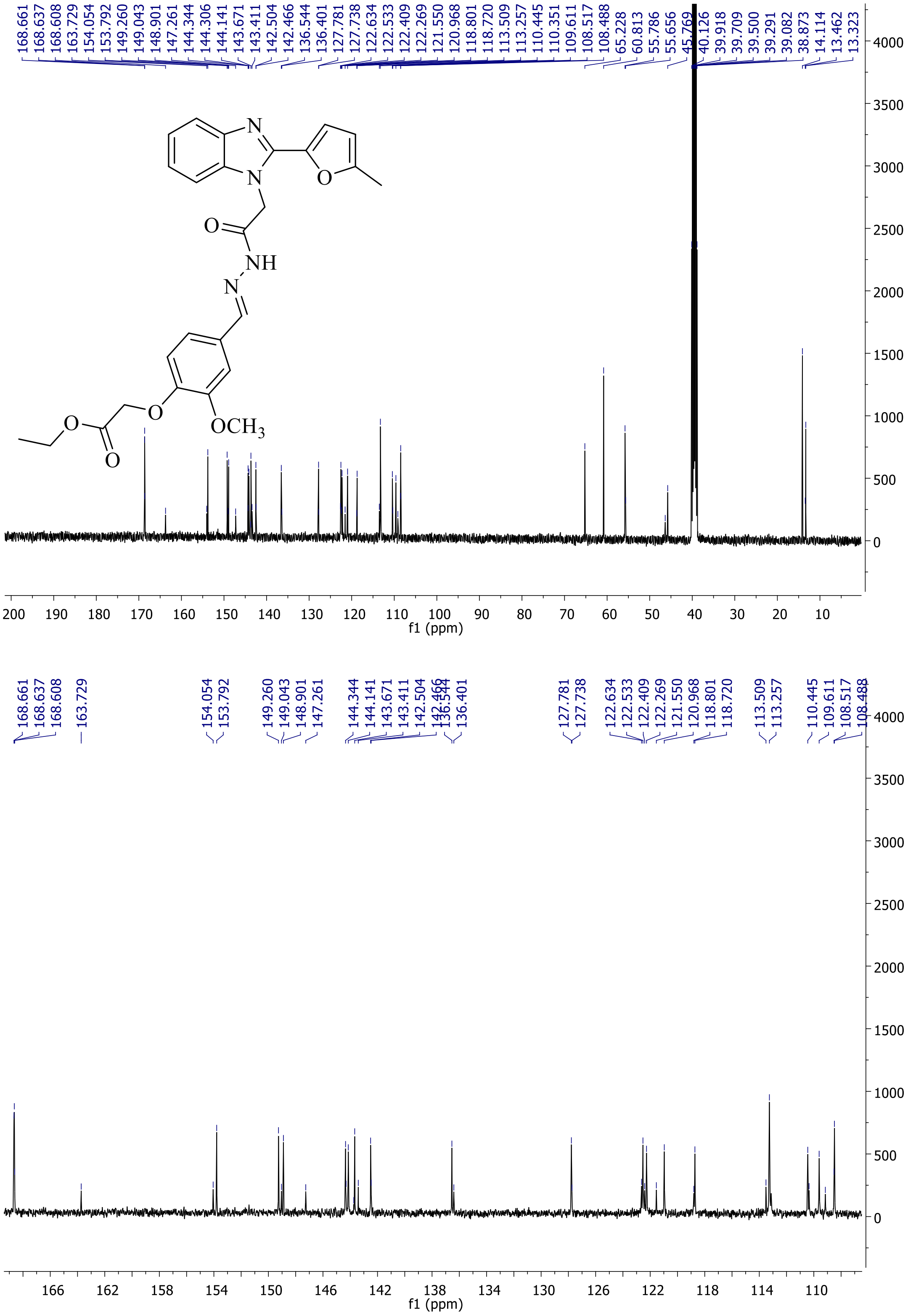

Fig. 44 ^{13}C (100 MHz) NMR spectrum of **19d** in DMSO-*d*

(*E*)-*N'*-(4-(2-(4-formyl-2-methoxyphenoxy)ethoxy)-3-methoxybenzylidene)-2-(2-isopropyl-1*H*-benzo[*d*]imidazol-1-yl)acetohydrazide (**22a**)

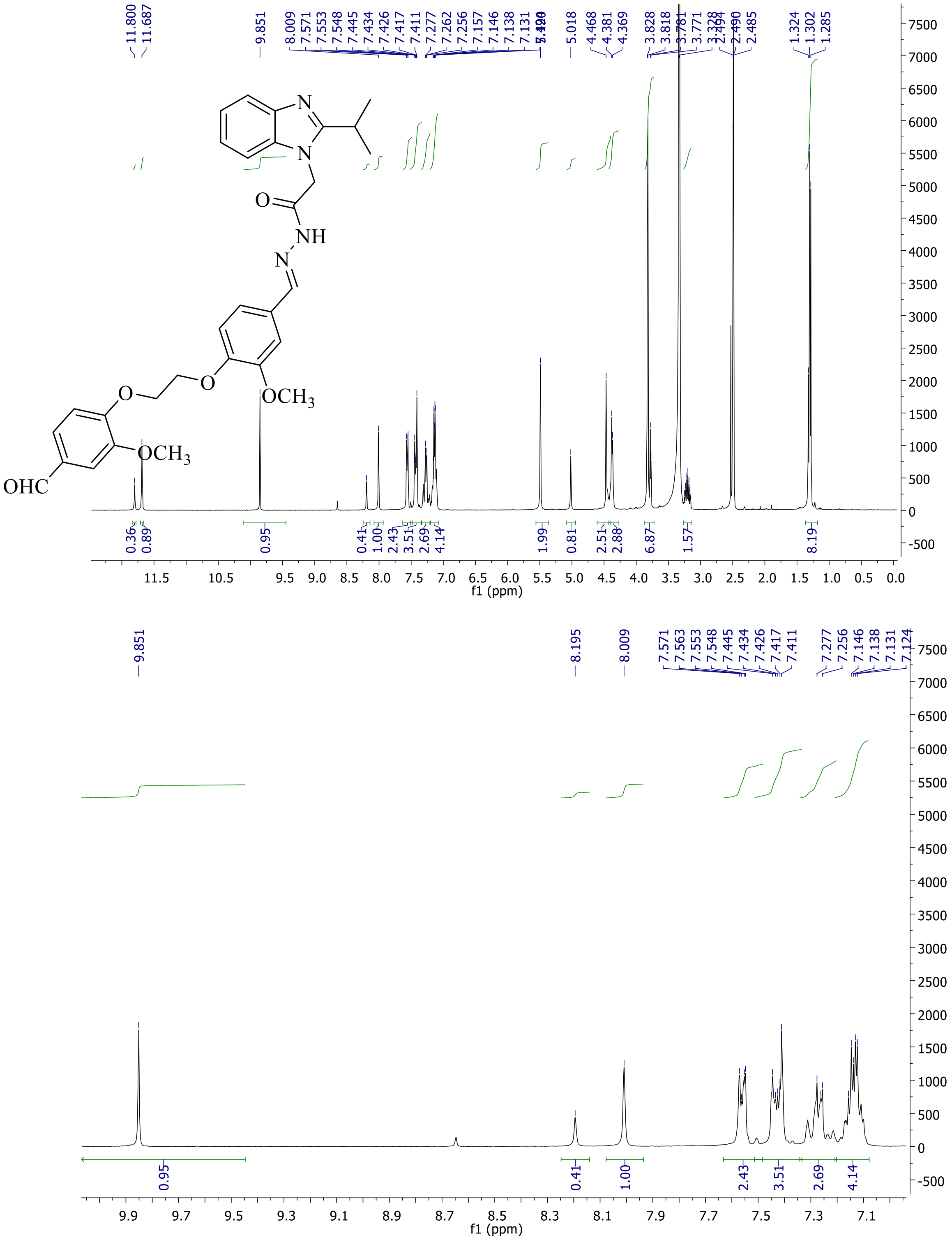

Fig. 45 ^{1}H (400 MHz) NMR spectrum of **22a** in DMSO-d_6

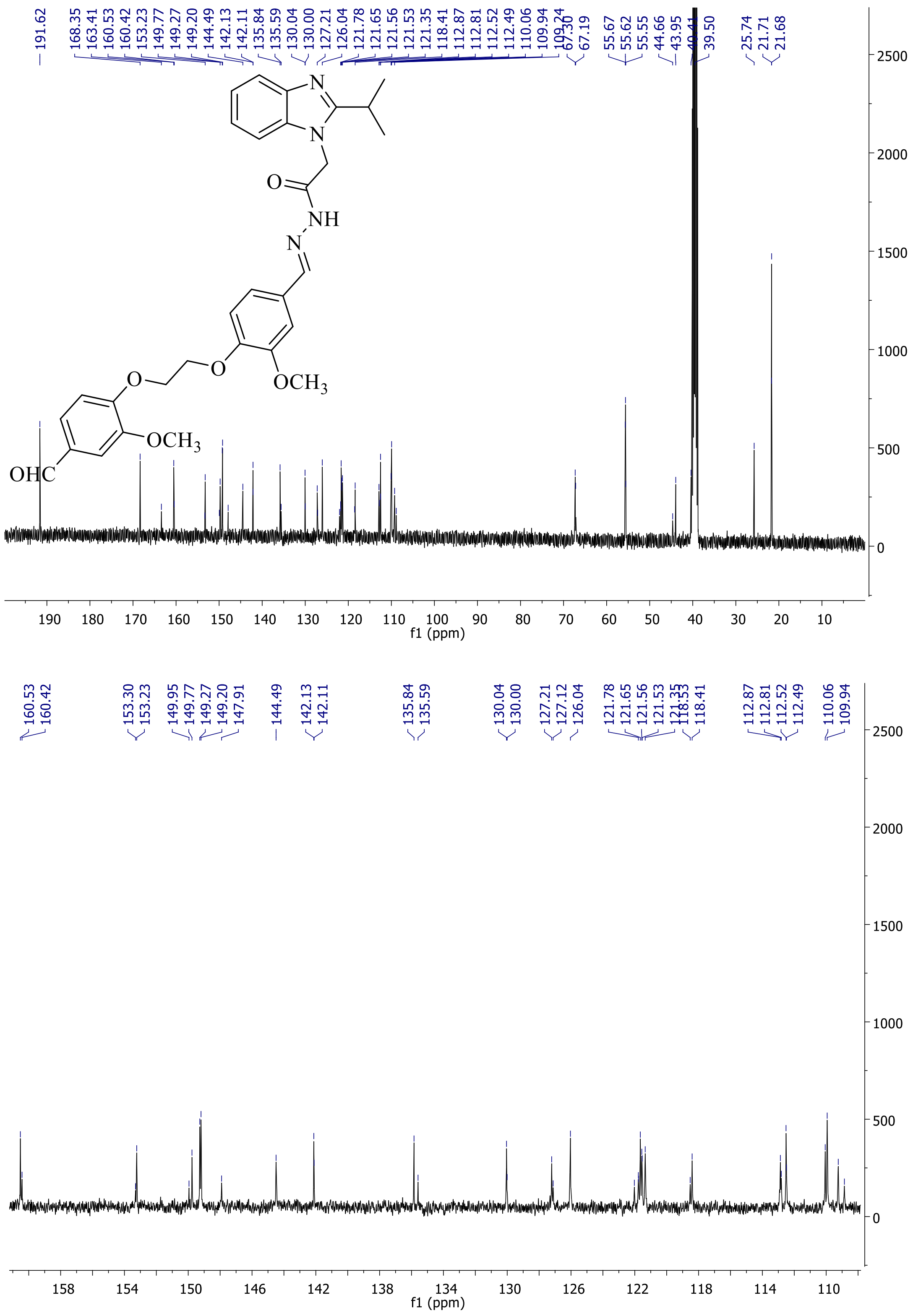

Fig. 46 ^{13}C (100 MHz) NMR spectrum of **22a** in DMSO-d_6

(*E*)-*N*'-(4-(2-(4-formyl-2-methoxyphenoxy)ethoxy)-3-methoxybenzylidene)-2-(2-(5-methylfuran-2-yl)-1*H*-benzo[*d*]imidazol-1-yl)acetohydrazide (**22b**)

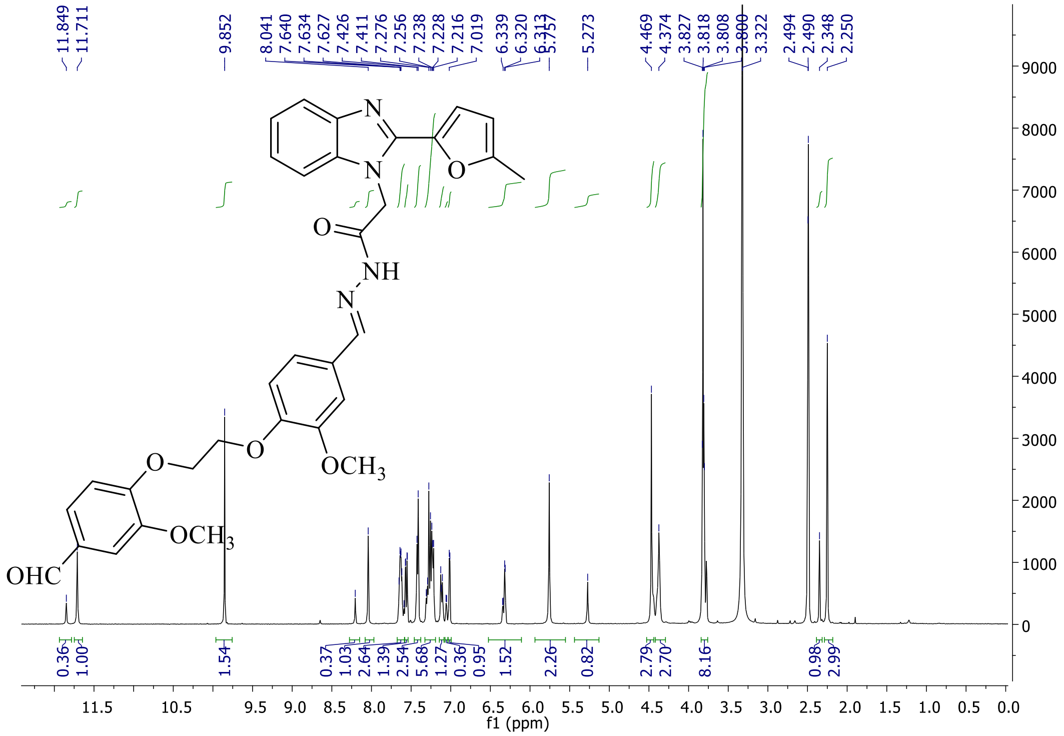

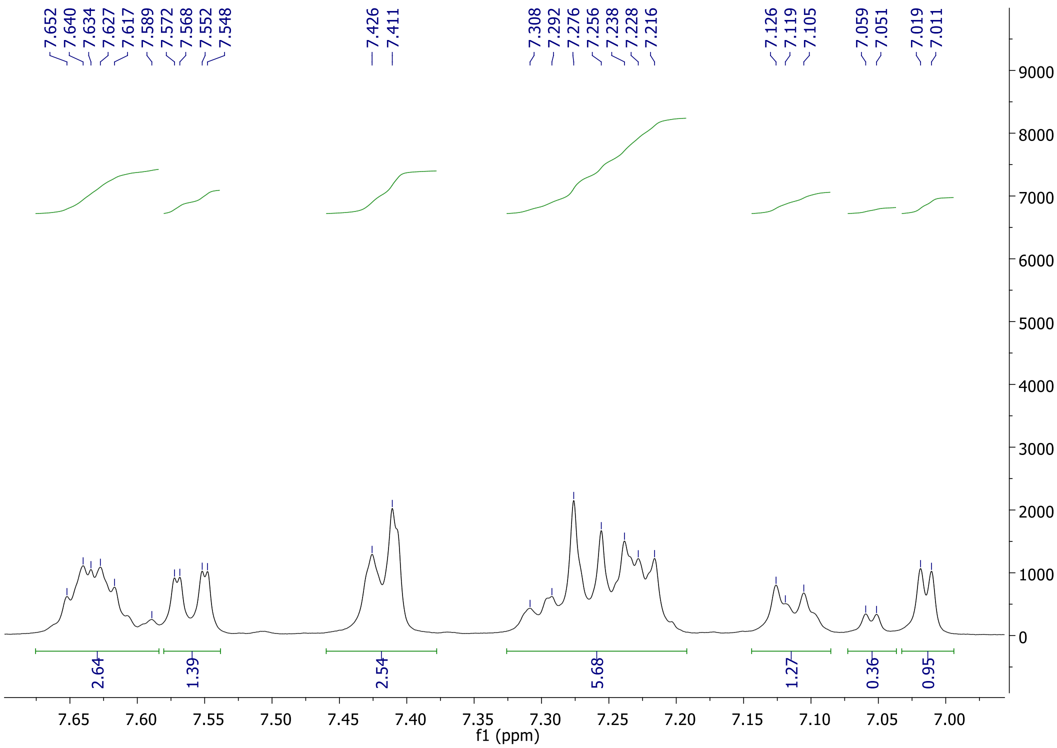

Fig. 47 ^{1}H (400 MHz) NMR spectrum of **22b** in DMSO-d_6

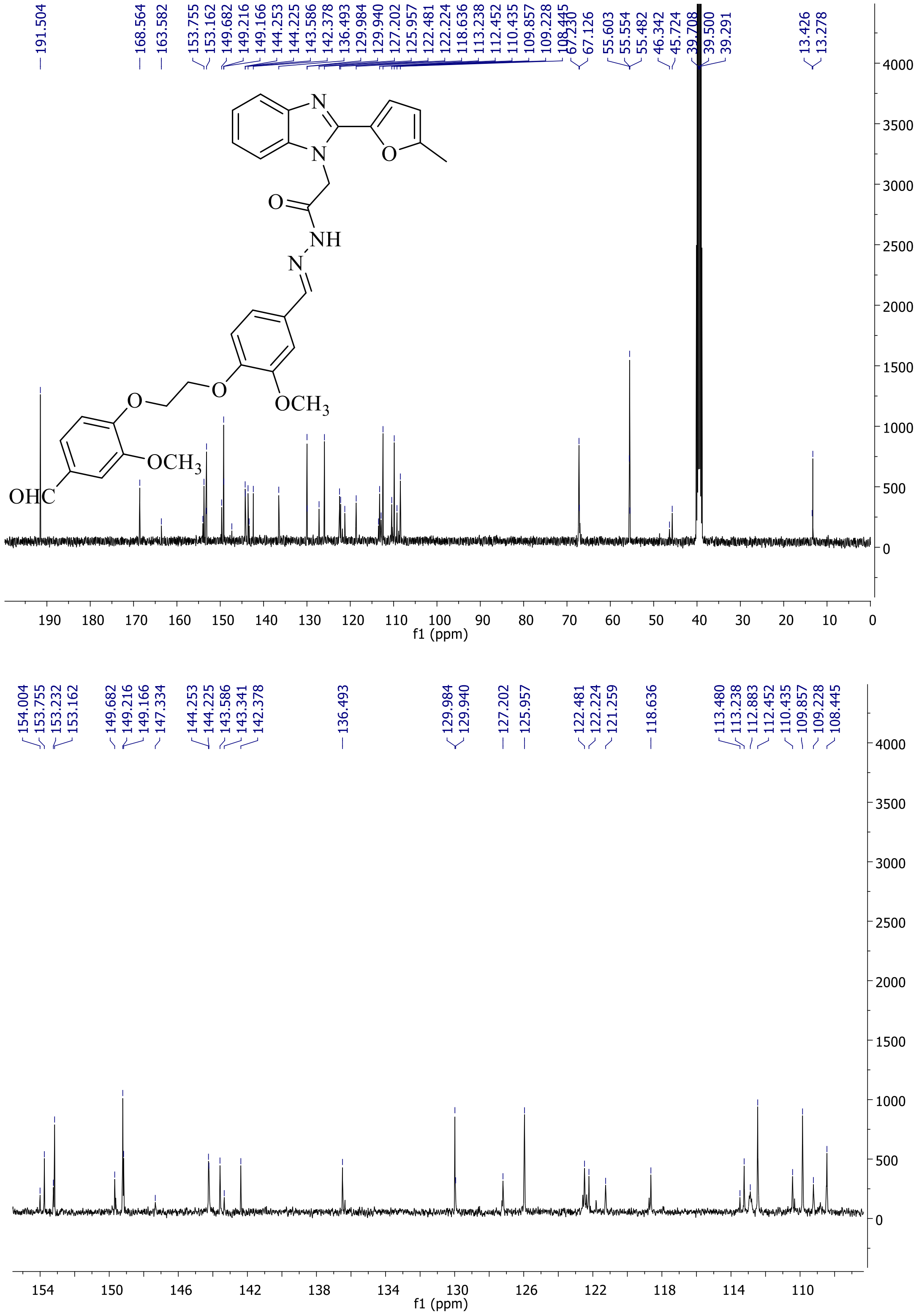

Fig. 48 ^{13}C (100 MHz) NMR spectrum of **22b** in DMSO-d_6

2-(2-(2-Isopropyl-1*H*-benzo[*d*]imidazol-1-yl)acetyl)-*N*-tosylhydrazine-1-carboxamide (**24a**)

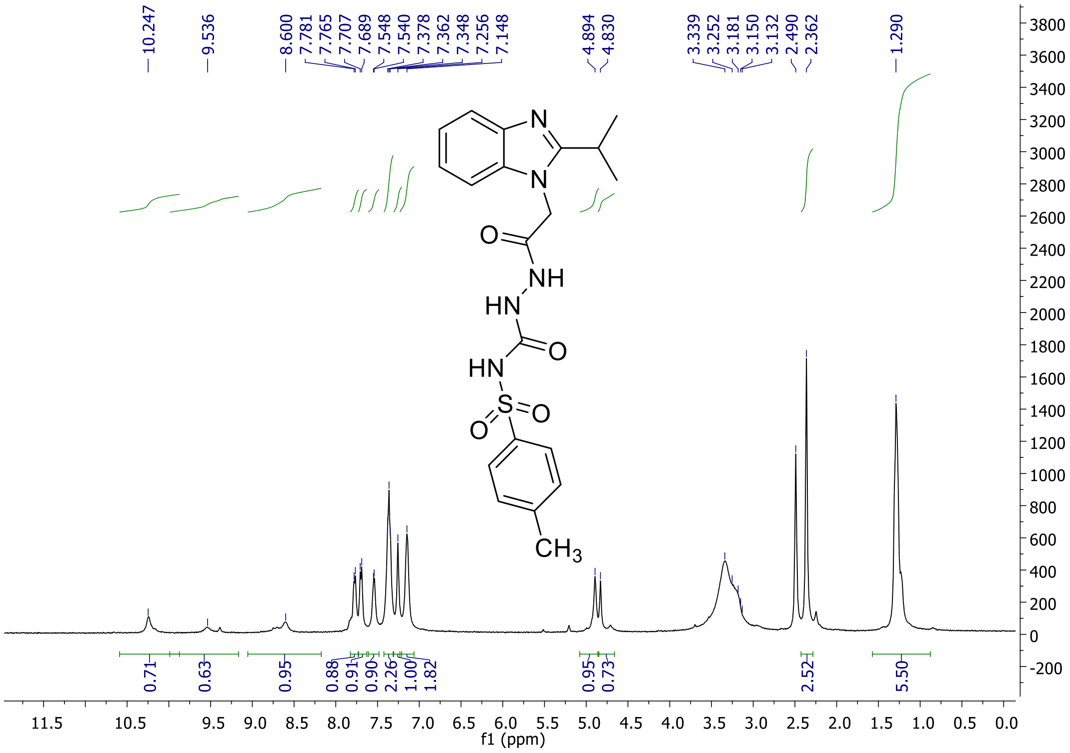

Fig. 49 ^{1}H (400 MHz) NMR spectrum of **24a** in DMSO-d_6

2-(2-(2-Isopropyl-1*H*-benzo[*d*]imidazol-1-yl)acetyl)-*N*-(4-methoxyphenyl)hydrazine-1-carbothioamide (**24b**).

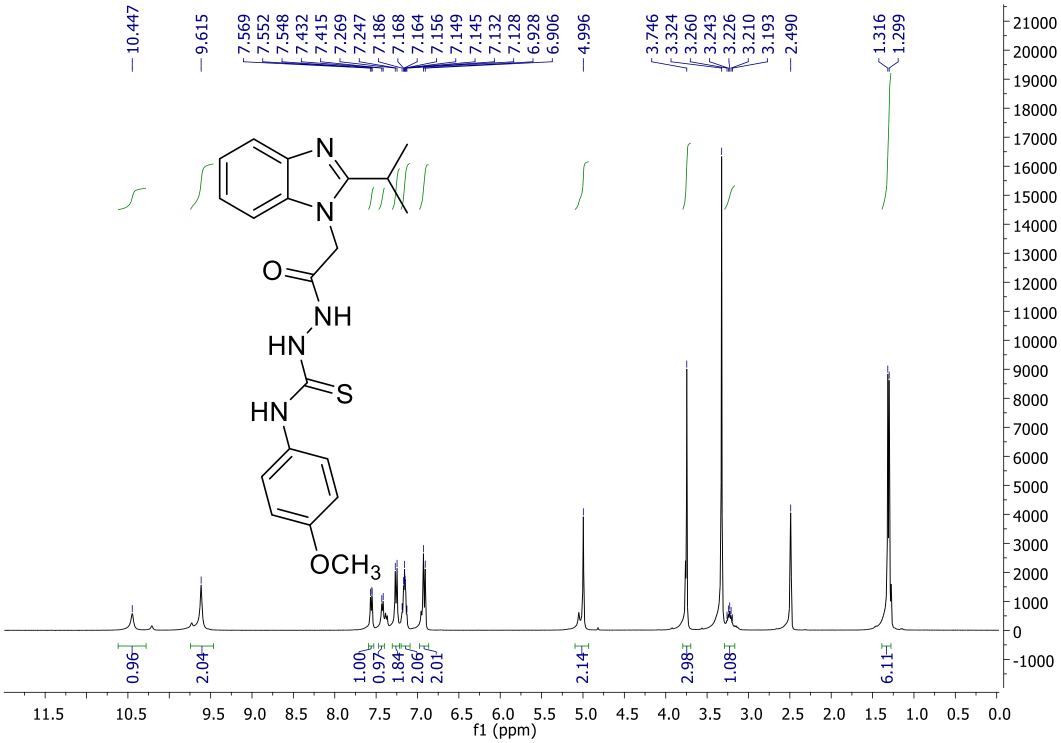

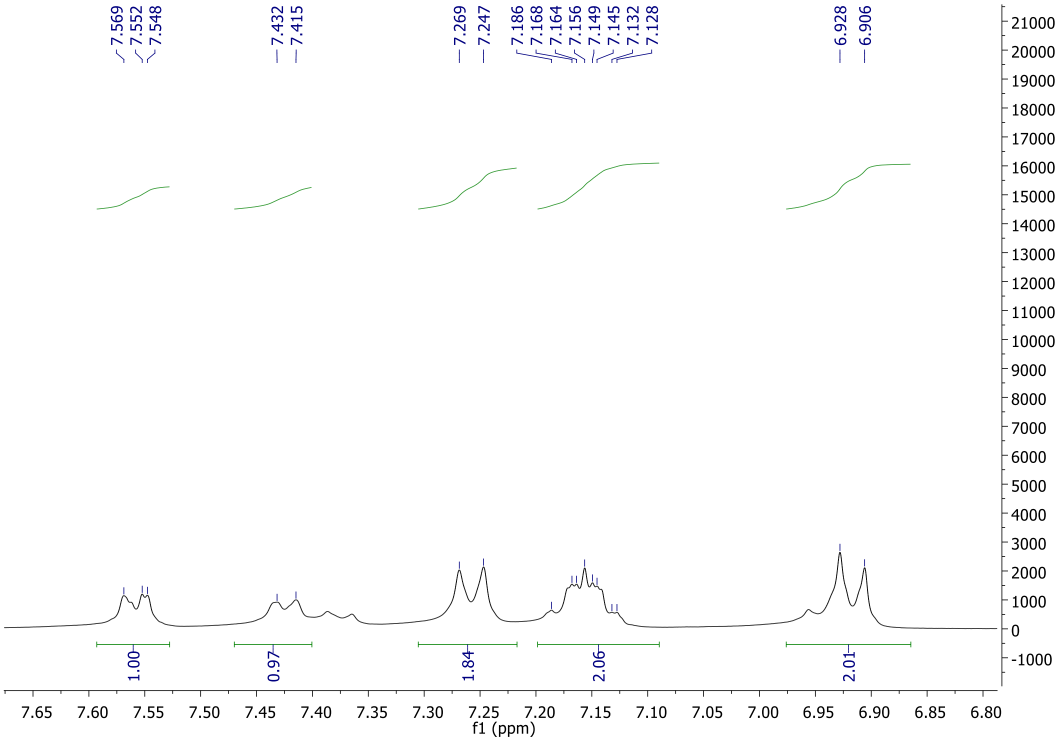

Fig. 50 ^{1}H (400 MHz) NMR spectrum of **24b** in DMSO-d_6

N-(4-Methoxyphenyl)-2-(2-(2-(5-methylfuran-2-yl)-1*H*-benzo[*d*]imidazol-1-yl)acetyl)hydrazine-1-carbothioamide (**24c**).

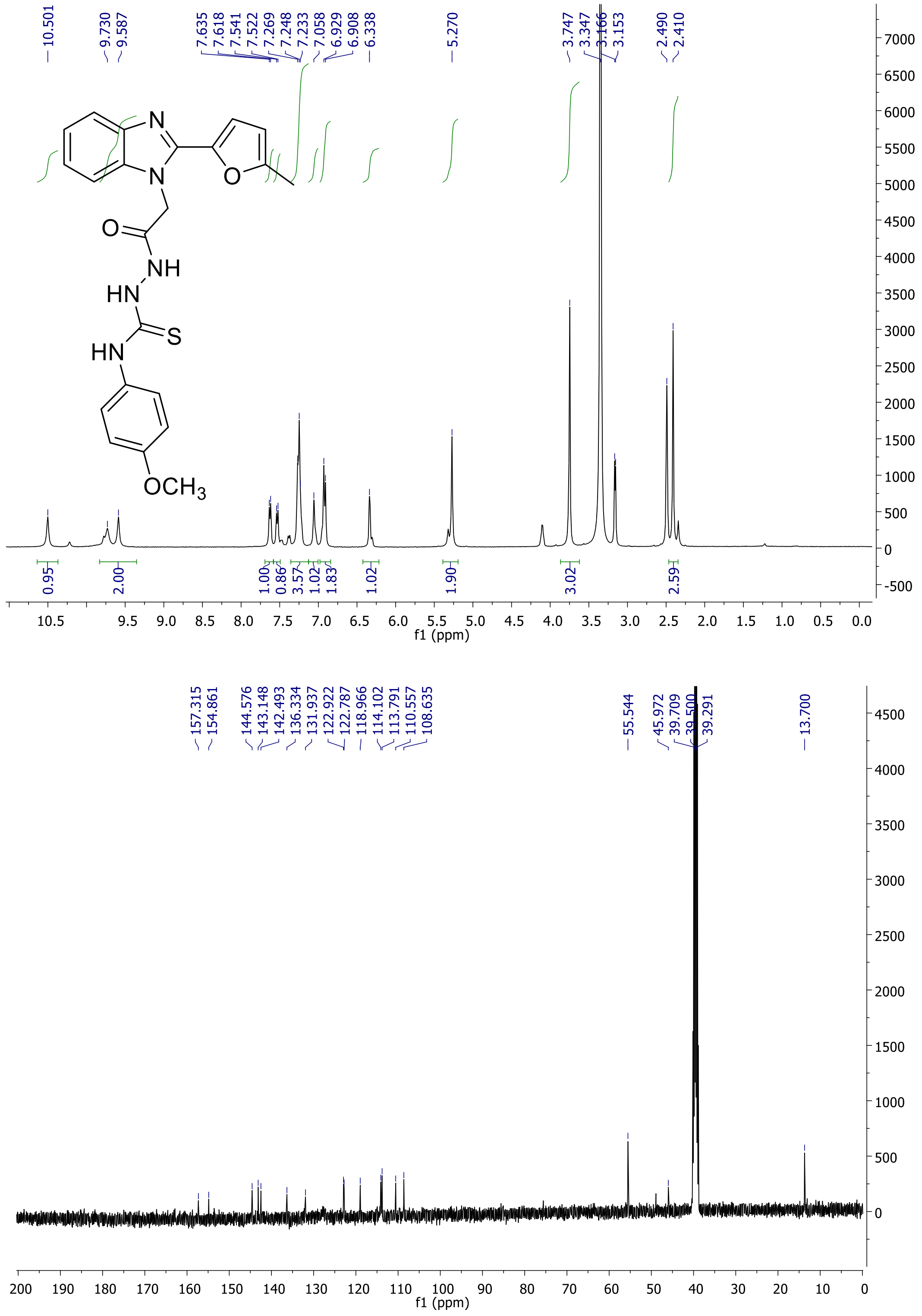

Fig. 51 ^{1}H (400 MHz) and ^{13}C (100 MHz) NMR spectra of **24c** in DMSO-d_6

2. Molecular structure and crystal data of 13c and 14a (Fig. 52-56 and Tables 1-16)

(*E*)-*N*'-(4-hydroxybenzylidene)-2-(2-isopropyl-1*H*-benzo[*d*]imidazol-1-yl)acetohydrazide (13c)

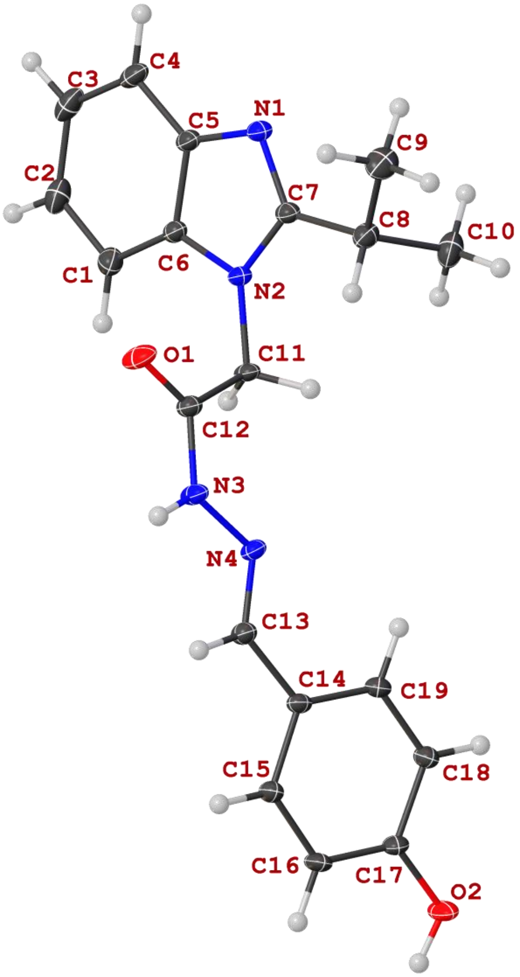

Fig.52: Molecular structure of **13c** (thermal displacement 50%). Solvent molecules excluded.

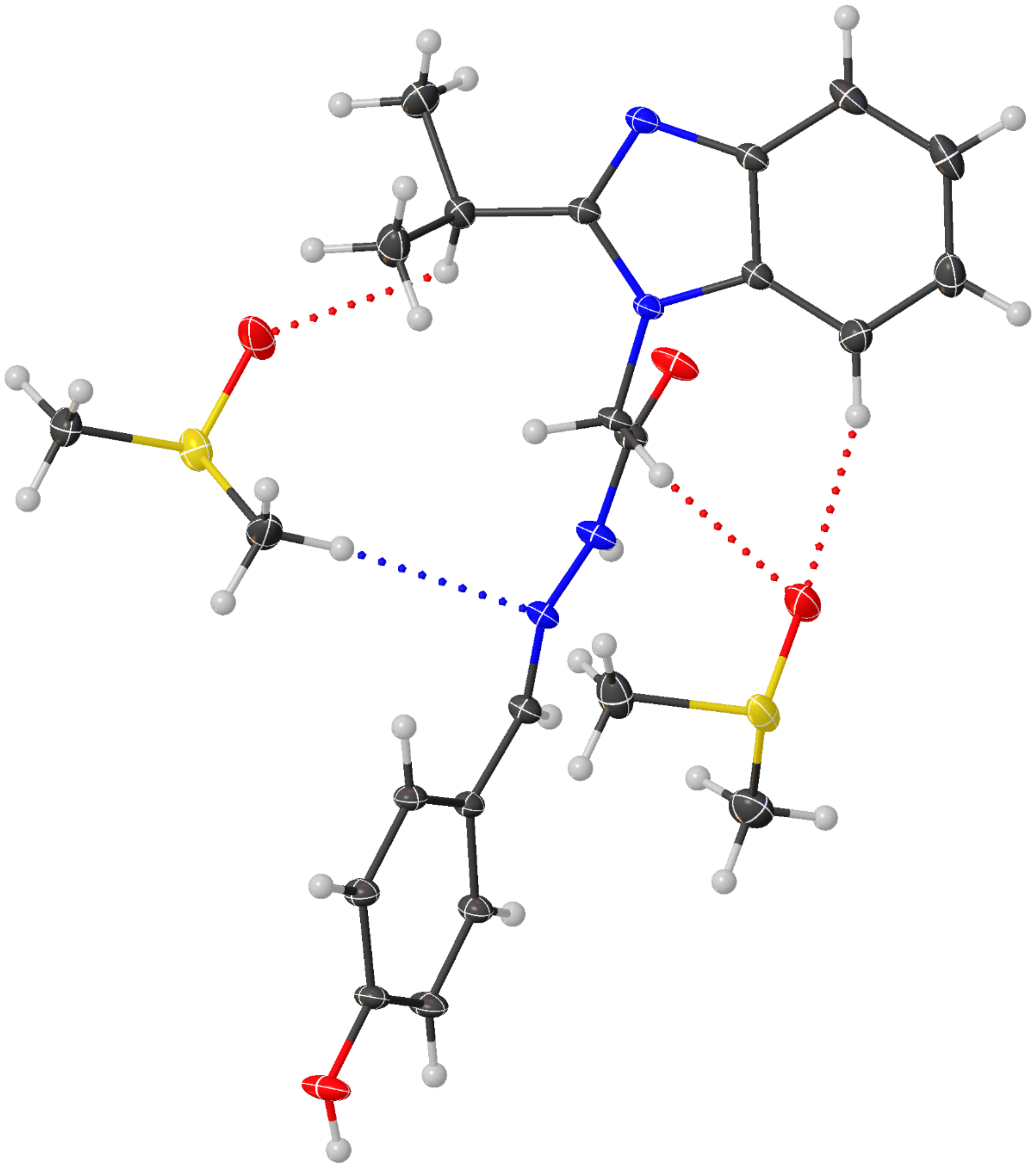

Fig.53: Molecular structure of **13c** (thermal displacement 50%). Labels have been omitted.

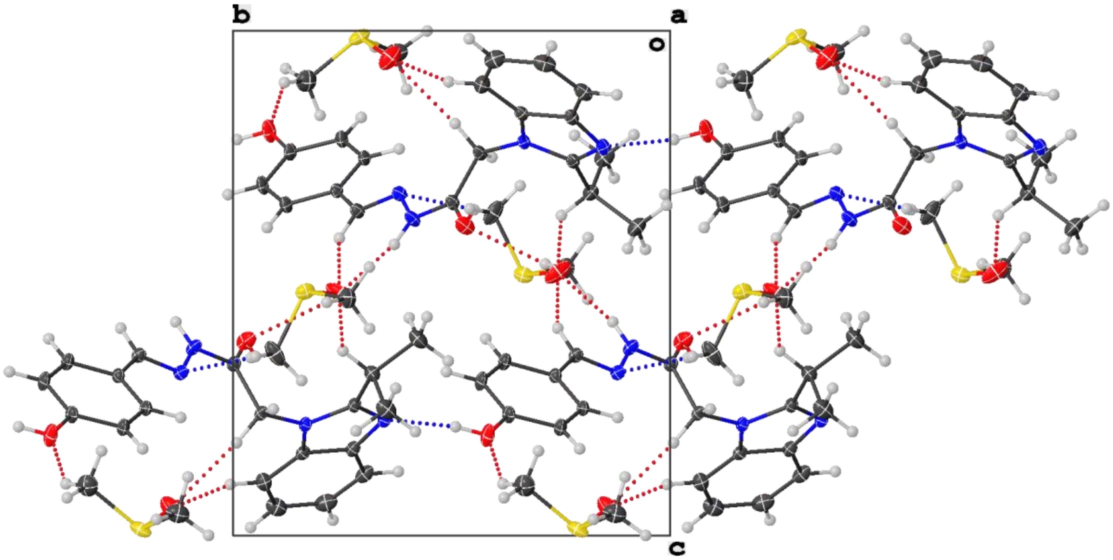

Fig 54: Moiety packing of **13c** with atom labels omitted.

Crystal Structure Report for 13c

A specimen of $C_{23}H_{32}N_4O_4S_2$, approximate dimensions 0.170 mm x 0.240 mm x 0.280 mm, was used for the X-ray crystallographic analysis. The X-ray intensity data were measured.

Table 1: Data collection details for 13c.

Axis	dx/mm	2θ/°	ω/°	φ/°	χ/°	Width/°	Frames	Time/s	Wavelength/Å	Voltage/kV	Current/mA	Temperature/K
Phi	41.320	8.00	0.00	0.00	54.76	1.00	180	5.00	0.71073	50	20.0	100
Omega	41.320	36.51	218.76	200.00	54.76	2.00	85	20.00	0.71073	50	20.0	100
Omega	41.320	36.51	218.76	80.00	54.76	2.00	85	20.00	0.71073	50	20.0	100
Omega	41.320	36.51	218.76	240.00	54.76	2.00	85	20.00	0.71073	50	20.0	100
Omega	41.320	-20.85	161.40	306.00	54.76	2.00	85	20.00	0.71073	50	20.0	100
Omega	41.320	36.51	218.76	40.00	54.76	2.00	85	20.00	0.71073	50	20.0	100
Omega	41.320	36.51	218.76	240.00	54.76	2.00	85	20.00	0.71073	50	20.0	100
Phi	41.320	36.51	218.51	204.00	54.76	2.00	96	20.00	0.71073	50	20.0	100
Phi	41.320	36.51	29.45	196.00	54.76	2.00	96	20.00	0.71073	50	20.0	100
Omega	41.320	-20.85	161.40	51.00	54.76	2.00	85	20.00	0.71073	50	20.0	100
Phi	41.320	-10.85	342.09	348.00	54.76	2.00	96	20.00	0.71073	50	20.0	100

A total of 1063 frames were collected. The total exposure time was 5.16 hours. The integration of the data using a monoclinic unit cell yielded a total of 68411 reflections to a maximum θ angle of 32.81° (0.66 Å resolution), of which 9427 were independent (average redundancy 7.257, completeness = 99.4%, R_{int} = 4.90%, R_{sig} = 3.37%) and 8408 (89.19%) were greater than $2\sigma(F^2)$. The final cell constants of a = 8.7106(9) Å, b = 11.2493(12) Å, c = 13.0942(11) Å, β = 95.743(3)°, volume = 1276.6(2) $Å^3$, are based upon the refinement of the XYZ-centroids of reflections above 20 σ(I). The calculated minimum and maximum transmission coefficients (based on crystal size) are 0.7150 and 0.7464.

The structure was solved and refined using the Bruker SHELXTL Software Package, using the space group P 1 21 1, with Z = 2 for the formula unit, $C_{23}H_{32}N_4O_4S_2$. The final anisotropic full-matrix least-squares refinement on F^2 with 348variables converged at R1 = 3.75%, for the observed data and wR2 = 8.87% for all data. The goodness-of-fit was 1.065. The largest peak in the final difference electron density synthesis was 0.550 $e^-/Å^3$ and the largest hole was -0.320 $e^-/Å^3$with an RMS deviation of 0.050 $e^-/Å^3$. On the basis of the final model, the calculated density was 1.282 g/cm^3 and F(000), 524 e^-.

Table 2. Crystal data and structure refinement for 13c

Identification code	tcd888	
Empirical formula	$C_{23}H_{32}N_4O_4S_2$	
Formula weight	492.64	
Temperature	99.9 K	
Wavelength	0.71073 Å	
Crystal system	Monoclinic	
Space group	P 1 21 1	
Unit cell dimensions	a = 8.7106(9) Å	α= 90°.
	b = 11.2493(12) Å	β= 95.743(3)°.
	c = 13.0942(11) Å	γ = 90°.
Volume	1276.6(2) $Å^3$	
Z	2	
Density (calculated)	1.282 Mg/m^3	
Absorption coefficient	0.244 mm^{-1}	
F(000)	524	
Crystal size	0.28 x 0.24 x 0.17 mm^3	
Theta range for data collection	2.950 to 32.811°.	
Index ranges	-13≤h≤13, -17≤k≤17, -19≤l≤19	
Reflections collected	68411	
Independent reflections	9427 [R_{int} = 0.0490]	
Completeness to theta = 25.242°	99.8 %	
Absorption correction	Semi-empirical from equivalents	
Max. and min. transmission	0.7464 and 0.7150	
Refinement method	Full-matrix least-squares on F^2	
Data / restraints / parameters	9427 / 3 / 348	
Goodness-of-fit on F^2	1.065	
Final R indices [I>2sigma(I)]	R1 = 0.0375, wR2 = 0.0850	
R indices (all data)	R1 = 0.0471, wR2 = 0.0887	
Absolute structure parameter	0.008(14)	
Extinction coefficient	n/a	
Largest diff. peak and hole	0.550 and -0.320 e.$Å^{-3}$	

Table 3. Atomic coordinates (x 10^4) and equivalent isotropic displacement parameters ($Å^2$x 10^3) for 13c. U(eq) is defined as one third of the trace of the orthogonalized U^{ij} tensor.

	x	y	z	U(eq)
S(1S)	6746(1)	3353(1)	4835(1)	21(1)
O(1S)	8151(2)	2570(2)	4788(1)	33(1)
C(1S)	5119(2)	2384(2)	4703(2)	23(1)
C(2S)	6468(2)	4043(2)	3607(2)	30(1)
O(1)	2884(2)	4698(1)	3861(1)	23(1)
O(2)	-6828(2)	9174(1)	1989(1)	25(1)
N(1)	2971(2)	1536(1)	2302(1)	16(1)
N(2)	1923(2)	3348(1)	2201(1)	13(1)
N(3)	813(2)	5897(1)	3724(1)	17(1)
N(4)	-617(2)	6196(1)	3227(1)	14(1)
C(1)	3782(2)	4300(2)	1064(2)	19(1)
C(2)	5073(2)	4012(2)	568(2)	24(1)
C(3)	5748(2)	2875(2)	649(2)	24(1)
C(4)	5148(2)	1986(2)	1218(1)	20(1)
C(5)	3837(2)	2254(2)	1716(1)	15(1)
C(6)	3193(2)	3400(2)	1639(1)	14(1)
C(7)	1856(2)	2220(2)	2583(1)	14(1)
C(8)	595(2)	1816(2)	3207(1)	17(1)
C(9)	1114(2)	763(2)	3889(2)	25(1)
C(10)	-861(2)	1499(2)	2500(2)	25(1)
C(11)	974(2)	4356(2)	2422(1)	14(1)
C(12)	1643(2)	4984(2)	3400(1)	15(1)
C(13)	-1189(2)	7156(2)	3570(1)	15(1)
C(14)	-2685(2)	7629(2)	3168(1)	14(1)
C(15)	-3042(2)	8789(2)	3443(1)	16(1)
C(16)	-4420(2)	9324(2)	3059(2)	18(1)
C(17)	-5481(2)	8692(2)	2401(1)	16(1)
C(18)	-5164(2)	7514(2)	2147(1)	16(1)
C(19)	-3777(2)	6996(2)	2522(1)	15(1)

S(2S)	9898(1)	7117(1)	126(1)	23(1)
O(2S)	11283(6)	6416(4)	541(3)	31(1)
C(3S)	9699(3)	8326(3)	993(2)	29(1)
C(4S)	8268(3)	6270(4)	438(2)	27(1)
S(3S)	-231(2)	2561(2)	8897(2)	25(1)
O(3S)	-1433(17)	1642(13)	9123(9)	27(2)
C(5S)	-381(11)	3740(8)	9790(7)	30(2)
C(6S)	1561(11)	1949(11)	9459(8)	31(2)

Table 4. Bond lengths [Å] and angles [°] for 13c.

S(1S)-O(1S)	1.5143(16)
S(1S)-C(1S)	1.7817(19)
S(1S)-C(2S)	1.780(2)
C(1S)-H(1SA)	0.9800
C(1S)-H(1SB)	0.9800
C(1S)-H(1SC)	0.9800
C(2S)-H(2SA)	0.9800
C(2S)-H(2SB)	0.9800
C(2S)-H(2SC)	0.9800
O(1)-C(12)	1.227(2)
O(2)-H(2)	0.865(13)
O(2)-C(17)	1.355(2)
N(1)-C(5)	1.388(2)
N(1)-C(7)	1.320(2)
N(2)-C(6)	1.389(2)
N(2)-C(7)	1.368(2)
N(2)-C(11)	1.449(2)
N(3)-H(3)	0.858(13)
N(3)-N(4)	1.388(2)
N(3)-C(12)	1.348(2)
N(4)-C(13)	1.288(2)
C(1)-H(1)	0.9500
C(1)-C(2)	1.391(3)
C(1)-C(6)	1.391(3)
C(2)-H(2A)	0.9500
C(2)-C(3)	1.408(3)
C(3)-H(3A)	0.9500
C(3)-C(4)	1.380(3)
C(4)-H(4)	0.9500
C(4)-C(5)	1.403(2)
C(5)-C(6)	1.405(2)
C(7)-C(8)	1.504(2)
C(8)-H(8)	1.0000
C(8)-C(9)	1.524(3)

C(8)-C(10)	1.535(3)
C(9)-H(9A)	0.9800
C(9)-H(9B)	0.9800
C(9)-H(9C)	0.9800
C(10)-H(10A)	0.9800
C(10)-H(10B)	0.9800
C(10)-H(10C)	0.9800
C(11)-H(11A)	0.9900
C(11)-H(11B)	0.9900
C(11)-C(12)	1.526(2)
C(13)-H(13)	0.9500
C(13)-C(14)	1.457(2)
C(14)-C(15)	1.397(2)
C(14)-C(19)	1.402(2)
C(15)-H(15)	0.9500
C(15)-C(16)	1.390(2)
C(16)-H(16)	0.9500
C(16)-C(17)	1.395(3)
C(17)-C(18)	1.400(2)
C(18)-H(18)	0.9500
C(18)-C(19)	1.387(2)
C(19)-H(19)	0.9500
S(2S)-O(2S)	1.497(5)
S(2S)-C(3S)	1.792(3)
S(2S)-C(4S)	1.791(3)
C(3S)-H(3SA)	0.9800
C(3S)-H(3SB)	0.9800
C(3S)-H(3SC)	0.9800
C(4S)-H(4SA)	0.9800
C(4S)-H(4SB)	0.9800
C(4S)-H(4SC)	0.9800
S(3S)-O(3S)	1.522(15)
S(3S)-C(5S)	1.781(9)
S(3S)-C(6S)	1.796(11)
C(5S)-H(5SA)	0.9800
C(5S)-H(5SB)	0.9800

C(5S)-H(5SC)	0.9800
C(6S)-H(6SA)	0.9800
C(6S)-H(6SB)	0.9800
C(6S)-H(6SC)	0.9800

O(1S)-S(1S)-C(1S)	106.14(10)
O(1S)-S(1S)-C(2S)	104.76(10)
C(2S)-S(1S)-C(1S)	98.31(10)
S(1S)-C(1S)-H(1SA)	109.5
S(1S)-C(1S)-H(1SB)	109.5
S(1S)-C(1S)-H(1SC)	109.5
H(1SA)-C(1S)-H(1SB)	109.5
H(1SA)-C(1S)-H(1SC)	109.5
H(1SB)-C(1S)-H(1SC)	109.5
S(1S)-C(2S)-H(2SA)	109.5
S(1S)-C(2S)-H(2SB)	109.5
S(1S)-C(2S)-H(2SC)	109.5
H(2SA)-C(2S)-H(2SB)	109.5
H(2SA)-C(2S)-H(2SC)	109.5
H(2SB)-C(2S)-H(2SC)	109.5
C(17)-O(2)-H(2)	113(2)
C(7)-N(1)-C(5)	105.74(14)
C(6)-N(2)-C(11)	124.97(15)
C(7)-N(2)-C(6)	107.35(14)
C(7)-N(2)-C(11)	127.35(14)
N(4)-N(3)-H(3)	120.6(17)
C(12)-N(3)-H(3)	117.9(17)
C(12)-N(3)-N(4)	121.42(15)
C(13)-N(4)-N(3)	113.56(15)
C(2)-C(1)-H(1)	121.8
C(6)-C(1)-H(1)	121.8
C(6)-C(1)-C(2)	116.30(17)
C(1)-C(2)-H(2A)	119.1
C(1)-C(2)-C(3)	121.80(18)
C(3)-C(2)-H(2A)	119.1
C(2)-C(3)-H(3A)	119.3

C(4)-C(3)-C(2)	121.39(17)
C(4)-C(3)-H(3A)	119.3
C(3)-C(4)-H(4)	121.1
C(3)-C(4)-C(5)	117.72(17)
C(5)-C(4)-H(4)	121.1
N(1)-C(5)-C(4)	130.14(17)
N(1)-C(5)-C(6)	109.75(14)
C(4)-C(5)-C(6)	120.11(16)
N(2)-C(6)-C(1)	132.41(16)
N(2)-C(6)-C(5)	104.89(15)
C(1)-C(6)-C(5)	122.66(15)
N(1)-C(7)-N(2)	112.27(14)
N(1)-C(7)-C(8)	125.00(16)
N(2)-C(7)-C(8)	122.67(14)
C(7)-C(8)-H(8)	108.2
C(7)-C(8)-C(9)	111.28(15)
C(7)-C(8)-C(10)	110.25(15)
C(9)-C(8)-H(8)	108.2
C(9)-C(8)-C(10)	110.63(16)
C(10)-C(8)-H(8)	108.2
C(8)-C(9)-H(9A)	109.5
C(8)-C(9)-H(9B)	109.5
C(8)-C(9)-H(9C)	109.5
H(9A)-C(9)-H(9B)	109.5
H(9A)-C(9)-H(9C)	109.5
H(9B)-C(9)-H(9C)	109.5
C(8)-C(10)-H(10A)	109.5
C(8)-C(10)-H(10B)	109.5
C(8)-C(10)-H(10C)	109.5
H(10A)-C(10)-H(10B)	109.5
H(10A)-C(10)-H(10C)	109.5
H(10B)-C(10)-H(10C)	109.5
N(2)-C(11)-H(11A)	109.5
N(2)-C(11)-H(11B)	109.5
N(2)-C(11)-C(12)	110.63(13)
H(11A)-C(11)-H(11B)	108.1

C(12)-C(11)-H(11A)	109.5
C(12)-C(11)-H(11B)	109.5
O(1)-C(12)-N(3)	121.21(17)
O(1)-C(12)-C(11)	122.35(15)
N(3)-C(12)-C(11)	116.43(15)
N(4)-C(13)-H(13)	118.5
N(4)-C(13)-C(14)	122.90(16)
C(14)-C(13)-H(13)	118.5
C(15)-C(14)-C(13)	117.55(16)
C(15)-C(14)-C(19)	118.36(15)
C(19)-C(14)-C(13)	124.09(15)
C(14)-C(15)-H(15)	119.4
C(16)-C(15)-C(14)	121.17(17)
C(16)-C(15)-H(15)	119.4
C(15)-C(16)-H(16)	120.1
C(15)-C(16)-C(17)	119.79(16)
C(17)-C(16)-H(16)	120.1
O(2)-C(17)-C(16)	122.39(16)
O(2)-C(17)-C(18)	117.89(17)
C(16)-C(17)-C(18)	119.71(16)
C(17)-C(18)-H(18)	120.1
C(19)-C(18)-C(17)	119.89(17)
C(19)-C(18)-H(18)	120.1
C(14)-C(19)-H(19)	119.5
C(18)-C(19)-C(14)	121.01(15)
C(18)-C(19)-H(19)	119.5
O(2S)-S(2S)-C(3S)	107.16(18)
O(2S)-S(2S)-C(4S)	105.4(2)
C(4S)-S(2S)-C(3S)	97.45(16)
S(2S)-C(3S)-H(3SA)	109.5
S(2S)-C(3S)-H(3SB)	109.5
S(2S)-C(3S)-H(3SC)	109.5
H(3SA)-C(3S)-H(3SB)	109.5
H(3SA)-C(3S)-H(3SC)	109.5
H(3SB)-C(3S)-H(3SC)	109.5
S(2S)-C(4S)-H(4SA)	109.5

S(2S)-C(4S)-H(4SB)	109.5
S(2S)-C(4S)-H(4SC)	109.5
H(4SA)-C(4S)-H(4SB)	109.5
H(4SA)-C(4S)-H(4SC)	109.5
H(4SB)-C(4S)-H(4SC)	109.5
O(3S)-S(3S)-C(5S)	106.3(7)
O(3S)-S(3S)-C(6S)	104.3(6)
C(5S)-S(3S)-C(6S)	97.7(5)
S(3S)-C(5S)-H(5SA)	109.5
S(3S)-C(5S)-H(5SB)	109.5
S(3S)-C(5S)-H(5SC)	109.5
H(5SA)-C(5S)-H(5SB)	109.5
H(5SA)-C(5S)-H(5SC)	109.5
H(5SB)-C(5S)-H(5SC)	109.5
S(3S)-C(6S)-H(6SA)	109.5
S(3S)-C(6S)-H(6SB)	109.5
S(3S)-C(6S)-H(6SC)	109.5
H(6SA)-C(6S)-H(6SB)	109.5
H(6SA)-C(6S)-H(6SC)	109.5
H(6SB)-C(6S)-H(6SC)	109.5

Symmetry transformations used to generate equivalent atoms:

Table 5. Anisotropic displacement parameters ($Å^2$x 10^3) for 13c. The anisotropic displacement factor exponent takes the form: $-2\pi^2$[h^2 $a^{*2}U^{11}$ + ... + 2 h k a* b* U^{12}]

	U^{11}	U^{22}	U^{33}	U^{23}	U^{13}	U^{12}
S(1S)	15(1)	27(1)	20(1)	-4(1)	1(1)	-3(1)
O(1S)	15(1)	50(1)	36(1)	25(1)	4(1)	10(1)
C(1S)	18(1)	20(1)	30(1)	1(1)	5(1)	-4(1)
C(2S)	16(1)	28(1)	43(1)	18(1)	-1(1)	2(1)
O(1)	17(1)	27(1)	24(1)	-6(1)	-6(1)	10(1)
O(2)	18(1)	14(1)	39(1)	-6(1)	-8(1)	7(1)
N(1)	13(1)	13(1)	20(1)	-2(1)	0(1)	3(1)
N(2)	12(1)	12(1)	16(1)	-1(1)	2(1)	3(1)
N(3)	13(1)	17(1)	18(1)	-6(1)	-3(1)	5(1)
N(4)	11(1)	15(1)	16(1)	-1(1)	1(1)	4(1)
C(1)	16(1)	18(1)	24(1)	2(1)	1(1)	-1(1)
C(2)	16(1)	31(1)	25(1)	4(1)	3(1)	-4(1)
C(3)	13(1)	37(1)	22(1)	-4(1)	4(1)	1(1)
C(4)	12(1)	24(1)	22(1)	-7(1)	0(1)	5(1)
C(5)	12(1)	15(1)	18(1)	-3(1)	0(1)	2(1)
C(6)	12(1)	14(1)	15(1)	-3(1)	0(1)	2(1)
C(7)	13(1)	13(1)	16(1)	-1(1)	-1(1)	2(1)
C(8)	15(1)	18(1)	19(1)	2(1)	3(1)	1(1)
C(9)	25(1)	25(1)	25(1)	10(1)	4(1)	2(1)
C(10)	17(1)	30(1)	28(1)	6(1)	0(1)	-4(1)
C(11)	12(1)	13(1)	16(1)	-3(1)	-1(1)	5(1)
C(12)	14(1)	14(1)	17(1)	-1(1)	1(1)	3(1)
C(13)	14(1)	15(1)	16(1)	-2(1)	1(1)	2(1)
C(14)	14(1)	11(1)	15(1)	-1(1)	3(1)	2(1)
C(15)	14(1)	13(1)	21(1)	-3(1)	0(1)	2(1)
C(16)	17(1)	11(1)	26(1)	-4(1)	0(1)	4(1)
C(17)	14(1)	12(1)	22(1)	0(1)	0(1)	4(1)
C(18)	16(1)	12(1)	21(1)	-2(1)	-1(1)	1(1)
C(19)	16(1)	11(1)	17(1)	-1(1)	2(1)	3(1)
S(2S)	17(1)	32(1)	19(1)	11(1)	5(1)	7(1)
O(2S)	16(1)	34(2)	41(2)	16(2)	5(2)	7(1)

C(3S)	23(1)	30(1)	34(1)	2(1)	-1(1)	5(1)
C(4S)	17(1)	40(2)	25(1)	3(1)	1(1)	-2(1)
S(3S)	21(1)	30(1)	23(1)	-4(1)	1(1)	-3(1)
O(3S)	15(3)	24(5)	40(7)	-6(4)	-1(4)	-3(3)
C(5S)	31(4)	27(4)	32(4)	-6(3)	-3(4)	-5(3)
C(6S)	23(4)	38(6)	32(5)	-11(4)	-1(3)	2(4)

Table 6. Hydrogen coordinates (x 10^4) and isotropic displacement parameters ($Å^2$x 10^3) for 13c.

	x	y	z	U(eq)
H(1SA)	5171	1878	4098	34
H(1SB)	4170	2856	4621	34
H(1SC)	5123	1886	5317	34
H(2SA)	7326	4588	3526	44
H(2SB)	5497	4489	3547	44
H(2SC)	6429	3433	3071	44
H(2)	-6930(30)	9912(14)	2160(20)	37
H(3)	1210(30)	6310(20)	4231(15)	20
H(1)	3329	5068	1012	23
H(2A)	5510	4599	163	29
H(3A)	6637	2715	306	29
H(4)	5606	1220	1270	23
H(8)	338	2488	3660	21
H(9A)	1306	77	3459	37
H(9B)	307	565	4331	37
H(9C)	2065	971	4316	37
H(10A)	-1183	2188	2072	38
H(10B)	-1691	1279	2917	38
H(10C)	-639	829	2060	38
H(11A)	-86	4080	2506	17
H(11B)	916	4920	1840	17
H(13)	-609	7573	4110	18
H(15)	-2331	9220	3900	19
H(16)	-4637	10118	3246	22
H(18)	-5899	7070	1718	20
H(19)	-3563	6200	2340	18
H(3SA)	9573	8012	1678	44
H(3SB)	8792	8800	750	44

H(3SC)	10623	8827	1026	44
H(4SA)	8160	5559	4	41
H(4SB)	7332	6755	320	41
H(4SC)	8418	6032	1161	41
H(5SA)	-372	3412	10483	46
H(5SB)	-1347	4174	9613	46
H(5SC)	494	4283	9764	46
H(6SA)	1506	1821	10195	47
H(6SB)	2403	2502	9361	47
H(6SC)	1753	1189	9129	47

Table 7. Torsion angles [°] for 13c.

O(2)-C(17)-C(18)-C(19)	177.80(17)
N(1)-C(5)-C(6)-N(2)	0.33(19)
N(1)-C(5)-C(6)-C(1)	-177.48(16)
N(1)-C(7)-C(8)-C(9)	26.8(2)
N(1)-C(7)-C(8)-C(10)	-96.4(2)
N(2)-C(7)-C(8)-C(9)	-156.29(17)
N(2)-C(7)-C(8)-C(10)	80.6(2)
N(2)-C(11)-C(12)-O(1)	5.3(2)
N(2)-C(11)-C(12)-N(3)	-176.12(15)
N(3)-N(4)-C(13)-C(14)	-179.39(15)
N(4)-N(3)-C(12)-O(1)	-177.58(17)
N(4)-N(3)-C(12)-C(11)	3.8(2)
N(4)-C(13)-C(14)-C(15)	-166.91(17)
N(4)-C(13)-C(14)-C(19)	12.8(3)
C(1)-C(2)-C(3)-C(4)	0.8(3)
C(2)-C(1)-C(6)-N(2)	-177.82(18)
C(2)-C(1)-C(6)-C(5)	-0.7(3)
C(2)-C(3)-C(4)-C(5)	-0.1(3)
C(3)-C(4)-C(5)-N(1)	177.67(18)
C(3)-C(4)-C(5)-C(6)	-0.9(3)
C(4)-C(5)-C(6)-N(2)	179.15(16)
C(4)-C(5)-C(6)-C(1)	1.3(3)
C(5)-N(1)-C(7)-N(2)	0.9(2)
C(5)-N(1)-C(7)-C(8)	178.12(16)
C(6)-N(2)-C(7)-N(1)	-0.71(19)
C(6)-N(2)-C(7)-C(8)	-178.01(15)
C(6)-N(2)-C(11)-C(12)	-87.38(19)
C(6)-C(1)-C(2)-C(3)	-0.4(3)
C(7)-N(1)-C(5)-C(4)	-179.42(18)
C(7)-N(1)-C(5)-C(6)	-0.75(19)
C(7)-N(2)-C(6)-C(1)	177.70(19)
C(7)-N(2)-C(6)-C(5)	0.21(18)
C(7)-N(2)-C(11)-C(12)	85.1(2)
C(11)-N(2)-C(6)-C(1)	-8.5(3)

C(11)-N(2)-C(6)-C(5)	173.96(15)
C(11)-N(2)-C(7)-N(1)	-174.27(15)
C(11)-N(2)-C(7)-C(8)	8.4(3)
C(12)-N(3)-N(4)-C(13)	-173.66(17)
C(13)-C(14)-C(15)-C(16)	177.45(17)
C(13)-C(14)-C(19)-C(18)	-178.38(16)
C(14)-C(15)-C(16)-C(17)	1.0(3)
C(15)-C(14)-C(19)-C(18)	1.4(2)
C(15)-C(16)-C(17)-O(2)	-178.72(18)
C(15)-C(16)-C(17)-C(18)	1.3(3)
C(16)-C(17)-C(18)-C(19)	-2.2(3)
C(17)-C(18)-C(19)-C(14)	0.9(3)
C(19)-C(14)-C(15)-C(16)	-2.3(3)

Symmetry transformations used to generate equivalent atoms:

Table 8. Hydrogen bonds for 13c [Å and °].

D-H...A	d(D-H)	d(H...A)	d(D...A)	<(DHA)
C(1S)-H(1SB)...O(1)	0.98	2.51	3.370(2)	145.9
C(2S)-H(2SA)...N(4)#1	0.98	2.60	3.579(3)	173.8
C(2S)-H(2SB)...O(1)	0.98	2.36	3.257(2)	151.1
O(2)-H(2)...N(1)#2	0.865(13)	1.841(14)	2.698(2)	170(3)
N(3)-H(3)...O(1S)#3	0.858(13)	1.952(14)	2.792(2)	166(3)
C(1)-H(1)...O(2S)#4	0.95	2.37	3.252(5)	153.6
C(1)-H(1)...O(3S)#5	0.95	2.42	3.330(15)	161.5
C(8)-H(8)...O(1S)#4	1.00	2.53	3.228(2)	126.9
C(11)-H(11B)...O(2S)#4	0.99	2.44	3.412(5)	168.8
C(11)-H(11B)...O(3S)#5	0.99	2.38	3.322(16)	158.9
C(13)-H(13)...O(1S)#3	0.95	2.46	3.272(2)	143.3
C(18)-H(18)...O(3S)#6	0.95	2.52	3.395(13)	153.8
C(3S)-H(3SC)...O(2)#7	0.98	2.47	3.313(3)	143.6
C(5S)-H(5SB)...O(2)#8	0.98	2.50	3.231(10)	131.0

Symmetry transformations used to generate equivalent atoms:
#1 x+1,y,z #2 x-1,y+1,z #3 -x+1,y+1/2,-z+1
#4 x-1,y,z #5 -x,y+1/2,-z+1 #6 -x-1,y+1/2,-z+1
#7 x+2,y,z #8 -x-1,y-1/2,-z+1

Crystal structure of (*N*'-[-1-phenylethylidene]-2-[2-(propan-2-yl)-1*H*-benzimidazol-1-yl]acetohydrazide (14)

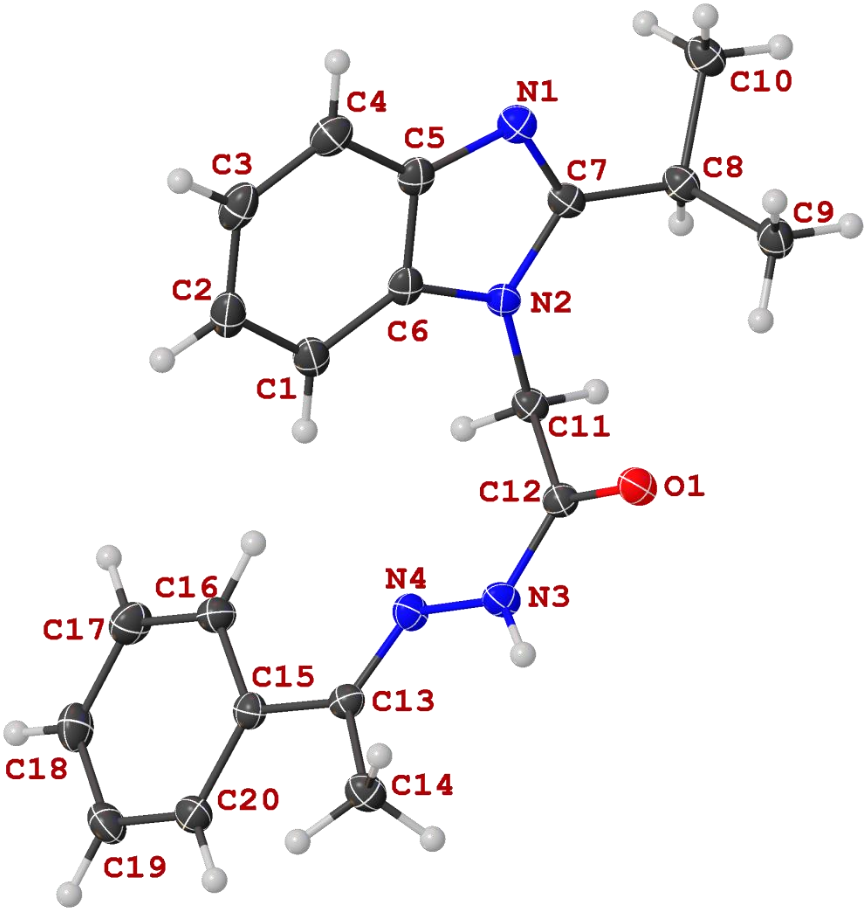

Fig.55: Molecular structure of **14a** (thermal displacement 50%).

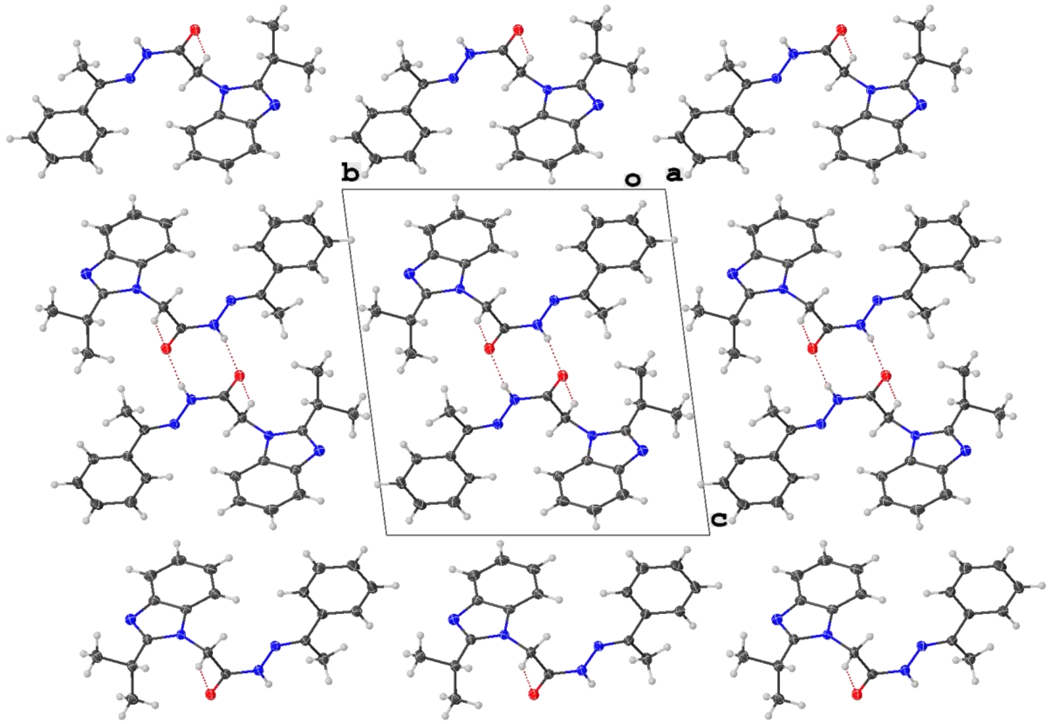

Fig 56: Moiety packing of **14a** shown with atom labels omitted.

Crystal Structure Report for **14a**

A specimen of $C_{20}H_{22}N_4O$, approximate dimensions 0.070 mm x 0.080 mm x 0.370 mm, was used for the X-ray crystallographic analysis. The X-ray intensity data were measured.

Table 9: Data collection details for 14a.

Axis	dx/mm	2θ/°	ω/°	φ/°	χ/°	Width/°	Frames	Time/s	Wavelength/Å	Voltage/kV	Current/mA	Temperature/K
Phi	41.320	8.00	0.00	0.00	54.76	1.00	180	10.00	0.71073	50	20.0	100
Omega	41.320	23.80	206.05	160.00	54.76	2.00	85	60.00	0.71073	50	20.0	100
Omega	41.320	23.80	206.05	40.00	54.76	2.00	85	60.00	0.71073	50	20.0	100
Omega	41.320	8.80	191.05	0.00	54.76	2.00	85	60.00	0.71073	50	20.0	100
Omega	41.320	23.80	206.05	80.00	54.76	2.00	85	60.00	0.71073	50	20.0	100
Omega	41.320	23.80	206.05	120.00	54.76	2.00	85	60.00	0.71073	50	20.0	100
Omega	41.320	23.80	206.05	0.00	54.76	2.00	85	60.00	0.71073	50	20.0	100

A total of 690 frames were collected. The total exposure time was 9.00 hours. The integration of the data using a triclinic unit cell yielded a total of 20218 reflections to a maximum θ angle of 26.55° (0.80 Å resolution), of which 3545 were independent (average redundancy 5.703, completeness = 99.5%, R_{int} = 5.54%, R_{sig} = 3.74%) and 2624 (74.02%) were greater than $2\sigma(F^2)$. The final cell constants of a = 4.6972(3) Å, b = 13.1750(8) Å, c = 14.1022(9) Å, α = 96.978(2)°, β = 92.318(2)°, γ = 97.067(2)°, volume = 858.32(9) $Å^3$, are based upon the refinement of the XYZ-centroids of reflections above 20 σ(I). The calculated minimum and maximum transmission coefficients (based on crystal size) are 0.6573 and 0.7454.

The structure was solved and refined using the Bruker SHELXTL Software Package, using the space group P -1, with Z = 2 for the formula unit, $C_{20}H_{22}N_4O$. The final anisotropic full-matrix least-squares refinement on F^2 with 233 variables converged at R1 = 3.99%, for the observed data and wR2 = 10.24% for all data. The goodness-of-fit was 1.037. The largest peak in the final difference electron density synthesis was 0.227 $e^-/Å^3$ and the largest hole was -0.256 $e^-/Å^3$ with an RMS deviation of 0.050 $e^-/Å^3$. On the basis of the final model, the calculated density was 1.294 g/cm^3 and F(000), 356 e^-.

Table 10. Crystal data and structure refinement for 14a.

Identification code	**14a**	
Empirical formula	$C_{20}H_{22}N_4O$	
Formula weight	334.41	
Temperature	100.0 K	
Wavelength	0.71073 Å	
Crystal system	Triclinic	
Space group	$P\bar{1}$	
Unit cell dimensions	a = 4.6972(3) Å	α= 96.978(2)°.
	b = 13.1750(8) Å	β= 92.318(2)°.
	c = 14.1022(9) Å	γ = 97.067(2)°.
Volume	858.32(9) $Å^3$	
Z	2	
Density (calculated)	1.294 Mg/m^3	
Absorption coefficient	0.083 mm^{-1}	
F(000)	356	
Crystal size	0.37 x 0.08 x 0.07 mm^3	
Theta range for data collection	2.915 to 26.550°.	
Index ranges	$-5 \leq h \leq 5$, $-16 \leq k \leq 16$, $-17 \leq l \leq 17$	
Reflections collected	20218	
Independent reflections	3545 [R_{int} = 0.0554]	
Completeness to theta = 25.242°	99.8 %	
Absorption correction	Semi-empirical from equivalents	
Max. and min. transmission	0.7454 and 0.6573	
Refinement method	Full-matrix least-squares on F^2	
Data / restraints / parameters	3545 / 1 / 233	
Goodness-of-fit on F^2	1.037	
Final R indices [I>2sigma(I)]	R1 = 0.0399, wR2 = 0.0921	
R indices (all data)	R1 = 0.0622, wR2 = 0.1024	
Extinction coefficient	n/a	
Largest diff. peak and hole	0.227 and -0.256 e.$Å^{-3}$	

Table 11. Atomic coordinates (x 10^4) and equivalent isotropic displacement parameters ($Å^2$x 10^3) for 14a. U(eq) is defined as one third of the trace of the orthogonalized U^{ij} tensor.

	x	y	z	U(eq)
O(1)	1681(2)	6083(1)	4611(1)	22(1)
N(1)	2915(3)	8255(1)	2441(1)	21(1)
C(1)	453(3)	5553(1)	1701(1)	22(1)
N(2)	3923(2)	6754(1)	2882(1)	17(1)
C(2)	-1628(3)	5591(1)	987(1)	26(1)
N(3)	2173(3)	4497(1)	3916(1)	19(1)
C(3)	-2322(3)	6528(1)	735(1)	27(1)
C(4)	-942(3)	7464(1)	1172(1)	24(1)
N(4)	3363(3)	3882(1)	3212(1)	19(1)
C(5)	1182(3)	7445(1)	1891(1)	20(1)
C(6)	1802(3)	6501(1)	2148(1)	18(1)
C(7)	4488(3)	7814(1)	3021(1)	18(1)
C(8)	6590(3)	8396(1)	3783(1)	20(1)
C(9)	5148(3)	8518(1)	4740(1)	24(1)
C(10)	7743(3)	9447(1)	3508(1)	24(1)
C(11)	5104(3)	5998(1)	3396(1)	18(1)
C(12)	2855(3)	5529(1)	4022(1)	18(1)
C(13)	2300(3)	2927(1)	3049(1)	18(1)
C(14)	-104(3)	2445(1)	3579(1)	22(1)
C(15)	3639(3)	2300(1)	2282(1)	18(1)
C(16)	5386(3)	2785(1)	1647(1)	22(1)
C(17)	6756(3)	2218(1)	961(1)	27(1)
C(18)	6403(4)	1151(1)	891(1)	27(1)
C(19)	4669(3)	655(1)	1510(1)	25(1)
C(20)	3288(3)	1229(1)	2200(1)	21(1)

Table 12. Bond lengths [Å] and angles [°] for 14a.

O(1)-C(12)	1.2305(17)
N(1)-C(5)	1.3922(19)
N(1)-C(7)	1.3129(19)
C(1)-H(1)	0.9500
C(1)-C(2)	1.384(2)
C(1)-C(6)	1.394(2)
N(2)-C(6)	1.3877(18)
N(2)-C(7)	1.3771(18)
N(2)-C(11)	1.4507(18)
C(2)-H(2)	0.9500
C(2)-C(3)	1.397(2)
N(3)-H(3)	0.888(9)
N(3)-N(4)	1.3842(16)
N(3)-C(12)	1.3470(19)
C(3)-H(3A)	0.9500
C(3)-C(4)	1.381(2)
C(4)-H(4)	0.9500
C(4)-C(5)	1.398(2)
N(4)-C(13)	1.2847(18)
C(5)-C(6)	1.398(2)
C(7)-C(8)	1.499(2)
C(8)-H(8)	1.0000
C(8)-C(9)	1.534(2)
C(8)-C(10)	1.524(2)
C(9)-H(9A)	0.9800
C(9)-H(9B)	0.9800
C(9)-H(9C)	0.9800
C(10)-H(10A)	0.9800
C(10)-H(10B)	0.9800
C(10)-H(10C)	0.9800
C(11)-H(11A)	0.9900
C(11)-H(11B)	0.9900
C(11)-C(12)	1.5251(19)
C(13)-C(14)	1.498(2)
C(13)-C(15)	1.487(2)
C(14)-H(14A)	0.9800
C(14)-H(14B)	0.9800

C(14)-H(14C)	0.9800
C(15)-C(16)	1.398(2)
C(15)-C(20)	1.390(2)
C(16)-H(16)	0.9500
C(16)-C(17)	1.379(2)
C(17)-H(17)	0.9500
C(17)-C(18)	1.386(2)
C(18)-H(18)	0.9500
C(18)-C(19)	1.385(2)
C(19)-H(19)	0.9500
C(19)-C(20)	1.391(2)
C(20)-H(20)	0.9500

C(7)-N(1)-C(5)	105.09(12)
C(2)-C(1)-H(1)	122.0
C(2)-C(1)-C(6)	115.96(14)
C(6)-C(1)-H(1)	122.0
C(6)-N(2)-C(11)	123.58(12)
C(7)-N(2)-C(6)	106.69(12)
C(7)-N(2)-C(11)	129.67(12)
C(1)-C(2)-H(2)	119.2
C(1)-C(2)-C(3)	121.68(15)
C(3)-C(2)-H(2)	119.2
N(4)-N(3)-H(3)	122.9(12)
C(12)-N(3)-H(3)	116.6(12)
C(12)-N(3)-N(4)	120.50(12)
C(2)-C(3)-H(3A)	119.0
C(4)-C(3)-C(2)	121.94(15)
C(4)-C(3)-H(3A)	119.0
C(3)-C(4)-H(4)	121.3
C(3)-C(4)-C(5)	117.46(15)
C(5)-C(4)-H(4)	121.3
C(13)-N(4)-N(3)	117.33(12)
N(1)-C(5)-C(4)	129.98(14)
N(1)-C(5)-C(6)	110.24(13)
C(6)-C(5)-C(4)	119.79(14)
C(1)-C(6)-C(5)	123.14(14)
N(2)-C(6)-C(1)	131.73(14)
N(2)-C(6)-C(5)	105.13(13)

N(1)-C(7)-N(2)	112.85(13)
N(1)-C(7)-C(8)	123.93(13)
N(2)-C(7)-C(8)	123.18(13)
C(7)-C(8)-H(8)	108.6
C(7)-C(8)-C(9)	109.77(12)
C(7)-C(8)-C(10)	110.67(13)
C(9)-C(8)-H(8)	108.6
C(10)-C(8)-H(8)	108.6
C(10)-C(8)-C(9)	110.65(12)
C(8)-C(9)-H(9A)	109.5
C(8)-C(9)-H(9B)	109.5
C(8)-C(9)-H(9C)	109.5
H(9A)-C(9)-H(9B)	109.5
H(9A)-C(9)-H(9C)	109.5
H(9B)-C(9)-H(9C)	109.5
C(8)-C(10)-H(10A)	109.5
C(8)-C(10)-H(10B)	109.5
C(8)-C(10)-H(10C)	109.5
H(10A)-C(10)-H(10B)	109.5
H(10A)-C(10)-H(10C)	109.5
H(10B)-C(10)-H(10C)	109.5
N(2)-C(11)-H(11A)	109.7
N(2)-C(11)-H(11B)	109.7
N(2)-C(11)-C(12)	109.93(11)
H(11A)-C(11)-H(11B)	108.2
C(12)-C(11)-H(11A)	109.7
C(12)-C(11)-H(11B)	109.7
O(1)-C(12)-N(3)	120.66(13)
O(1)-C(12)-C(11)	120.73(13)
N(3)-C(12)-C(11)	118.61(12)
N(4)-C(13)-C(14)	124.07(13)
N(4)-C(13)-C(15)	115.26(13)
C(15)-C(13)-C(14)	120.67(13)
C(13)-C(14)-H(14A)	109.5
C(13)-C(14)-H(14B)	109.5
C(13)-C(14)-H(14C)	109.5
H(14A)-C(14)-H(14B)	109.5
H(14A)-C(14)-H(14C)	109.5
H(14B)-C(14)-H(14C)	109.5

C(16)-C(15)-C(13)	120.15(13)
C(20)-C(15)-C(13)	121.41(13)
C(20)-C(15)-C(16)	118.39(13)
C(15)-C(16)-H(16)	119.5
C(17)-C(16)-C(15)	120.95(14)
C(17)-C(16)-H(16)	119.5
C(16)-C(17)-H(17)	120.0
C(16)-C(17)-C(18)	120.07(15)
C(18)-C(17)-H(17)	120.0
C(17)-C(18)-H(18)	120.0
C(19)-C(18)-C(17)	119.91(14)
C(19)-C(18)-H(18)	120.0
C(18)-C(19)-H(19)	120.1
C(18)-C(19)-C(20)	119.89(14)
C(20)-C(19)-H(19)	120.1
C(15)-C(20)-C(19)	120.79(14)
C(15)-C(20)-H(20)	119.6
C(19)-C(20)-H(20)	119.6

Symmetry transformations used to generate equivalent atoms:

Table 13. Anisotropic displacement parameters ($Å^2 x 10^3$) for tcd870. The anisotropic displacement factor exponent takes the form: $-2\pi^2[h^2 a^{*2}U^{11} + ... + 2 h k a^* b^* U^{12}]$

	U^{11}	U^{22}	U^{33}	U^{23}	U^{13}	U^{12}
O(1)	25(1)	20(1)	23(1)	1(1)	9(1)	2(1)
N(1)	17(1)	22(1)	22(1)	3(1)	3(1)	1(1)
C(1)	21(1)	23(1)	19(1)	1(1)	5(1)	-2(1)
N(2)	16(1)	17(1)	17(1)	3(1)	2(1)	-1(1)
C(2)	23(1)	32(1)	19(1)	-1(1)	3(1)	-7(1)
N(3)	19(1)	18(1)	21(1)	2(1)	8(1)	1(1)
C(3)	20(1)	41(1)	19(1)	5(1)	0(1)	-2(1)
C(4)	22(1)	31(1)	22(1)	7(1)	5(1)	4(1)
N(4)	20(1)	19(1)	19(1)	1(1)	5(1)	3(1)
C(5)	17(1)	24(1)	18(1)	3(1)	6(1)	1(1)
C(6)	15(1)	24(1)	16(1)	2(1)	5(1)	-1(1)
C(7)	14(1)	18(1)	21(1)	2(1)	7(1)	1(1)
C(8)	16(1)	18(1)	23(1)	1(1)	1(1)	0(1)
C(9)	24(1)	24(1)	22(1)	0(1)	0(1)	-1(1)
C(10)	22(1)	20(1)	28(1)	1(1)	3(1)	-2(1)
C(11)	16(1)	17(1)	21(1)	2(1)	3(1)	1(1)
C(12)	17(1)	18(1)	18(1)	3(1)	2(1)	2(1)
C(13)	16(1)	19(1)	18(1)	4(1)	0(1)	2(1)
C(14)	19(1)	21(1)	26(1)	2(1)	5(1)	0(1)
C(15)	17(1)	20(1)	17(1)	1(1)	-1(1)	2(1)
C(16)	26(1)	20(1)	21(1)	4(1)	3(1)	2(1)
C(17)	28(1)	30(1)	23(1)	5(1)	8(1)	4(1)
C(18)	30(1)	29(1)	21(1)	-4(1)	3(1)	9(1)
C(19)	29(1)	18(1)	28(1)	-2(1)	-1(1)	3(1)
C(20)	20(1)	20(1)	22(1)	2(1)	1(1)	-1(1)

Table 14. Hydrogen coordinates (x 10^4) and isotropic displacement parameters (Å^{2}x 10^3) for 14a.

_	x	y	z	U(eq)
H(1)	931	4918	1875	26
H(3)	920(30)	4245(13)	4308(11)	32(5)
H(2)	-2609	4965	660	31
H(3A)	-3785	6521	248	33
H(4)	-1419	8096	992	29
H(8)	8237	7992	3854	23
H(9A)	3484	8891	4675	36
H(9B)	6524	8906	5235	36
H(9C)	4515	7835	4923	36
H(10A)	8691	9353	2902	36
H(10B)	9131	9812	4010	36
H(10C)	6150	9851	3433	36
H(11A)	6817	6332	3800	22
H(11B)	5708	5448	2934	22
H(14A)	611	2370	4226	33
H(14B)	-850	1764	3240	33
H(14C)	-1644	2884	3618	33
H(16)	5633	3516	1688	27
H(17)	7943	2559	536	32
H(18)	7349	760	419	32
H(19)	4423	-76	1463	30
H(20)	2092	886	2621	25

Table 15. Torsion angles [°] for 14a.

N(1)-C(5)-C(6)-C(1)	-178.33(13)
N(1)-C(5)-C(6)-N(2)	1.02(15)
N(1)-C(7)-C(8)-C(9)	94.66(16)
N(1)-C(7)-C(8)-C(10)	-27.77(19)
C(1)-C(2)-C(3)-C(4)	1.0(2)
N(2)-C(7)-C(8)-C(9)	-82.67(16)
N(2)-C(7)-C(8)-C(10)	154.90(13)
N(2)-C(11)-C(12)-O(1)	-55.15(18)
N(2)-C(11)-C(12)-N(3)	124.66(14)
C(2)-C(1)-C(6)-N(2)	179.40(14)
C(2)-C(1)-C(6)-C(5)	-1.4(2)
C(2)-C(3)-C(4)-C(5)	-0.5(2)
N(3)-N(4)-C(13)-C(14)	-0.7(2)
N(3)-N(4)-C(13)-C(15)	179.64(12)
C(3)-C(4)-C(5)-N(1)	179.45(14)
C(3)-C(4)-C(5)-C(6)	-0.9(2)
C(4)-C(5)-C(6)-C(1)	2.0(2)
C(4)-C(5)-C(6)-N(2)	-178.70(12)
N(4)-N(3)-C(12)-O(1)	175.91(12)
N(4)-N(3)-C(12)-C(11)	-3.9(2)
N(4)-C(13)-C(15)-C(16)	-15.2(2)
N(4)-C(13)-C(15)-C(20)	162.30(13)
C(5)-N(1)-C(7)-N(2)	0.63(15)
C(5)-N(1)-C(7)-C(8)	-176.94(13)
C(6)-C(1)-C(2)-C(3)	0.0(2)
C(6)-N(2)-C(7)-N(1)	-0.01(15)
C(6)-N(2)-C(7)-C(8)	177.59(12)
C(6)-N(2)-C(11)-C(12)	-69.75(16)
C(7)-N(1)-C(5)-C(4)	178.66(15)
C(7)-N(1)-C(5)-C(6)	-1.03(15)
C(7)-N(2)-C(6)-C(1)	178.65(15)
C(7)-N(2)-C(6)-C(5)	-0.62(14)

C(7)-N(2)-C(11)-C(12)	107.06(15)
C(11)-N(2)-C(6)-C(1)	-3.9(2)
C(11)-N(2)-C(6)-C(5)	176.82(12)
C(11)-N(2)-C(7)-N(1)	-177.23(13)
C(11)-N(2)-C(7)-C(8)	0.4(2)
C(12)-N(3)-N(4)-C(13)	-169.42(13)
C(13)-C(15)-C(16)-C(17)	176.88(14)
C(13)-C(15)-C(20)-C(19)	-176.80(14)
C(14)-C(13)-C(15)-C(16)	165.18(13)
C(14)-C(13)-C(15)-C(20)	-17.3(2)
C(15)-C(16)-C(17)-C(18)	0.3(2)
C(16)-C(15)-C(20)-C(19)	0.7(2)
C(16)-C(17)-C(18)-C(19)	0.1(2)
C(17)-C(18)-C(19)-C(20)	0.0(2)
C(18)-C(19)-C(20)-C(15)	-0.4(2)
C(20)-C(15)-C(16)-C(17)	-0.7(2)

Symmetry transformations used to generate equivalent atoms:

Table 16. Hydrogen bonds for 14a [Å and °].

D-H...A	d(D-H)	d(H...A)	d(D...A)	<(DHA)
N(3)-H(3)...O(1)#1	0.888(9)	2.044(10)	2.9197(16)	168.8(17)
C(11)-H(11A)...O(1)#2	0.99	2.59	3.4546(18)	146.0

Symmetry transformations used to generate equivalent atoms:
#1 -x,-y+1,-z+1 #2 x+1,y,z

3. Docking validation of sorafenib, the co-crystalized ligand, in the VEGFR-2 active site (Fig. 57)

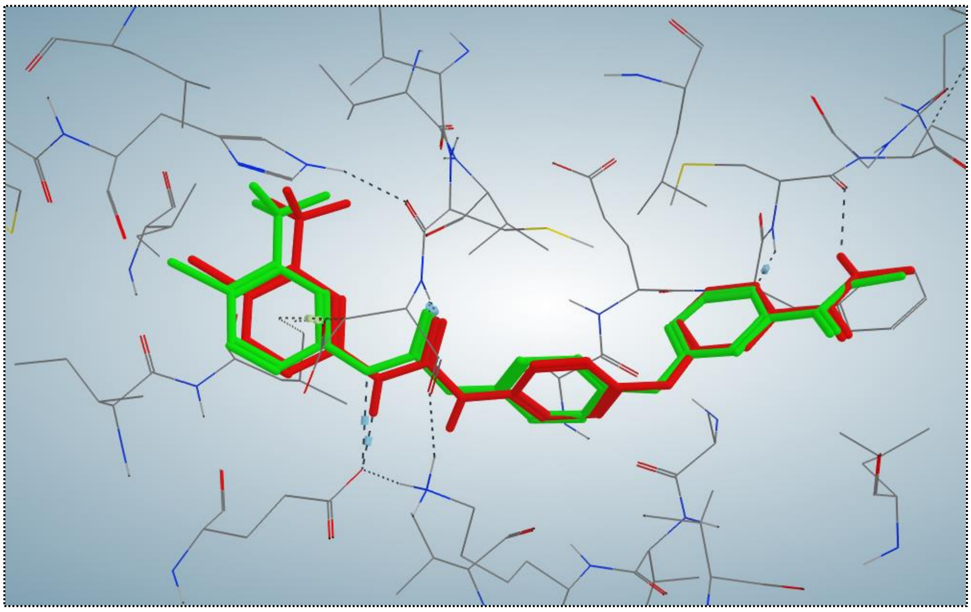

(A)

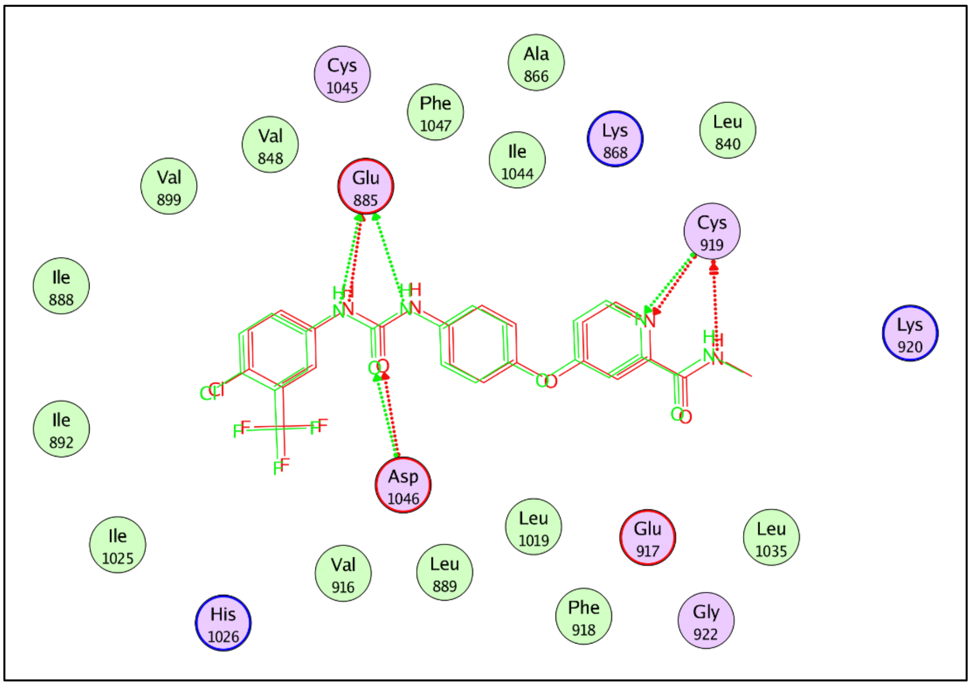

(B)

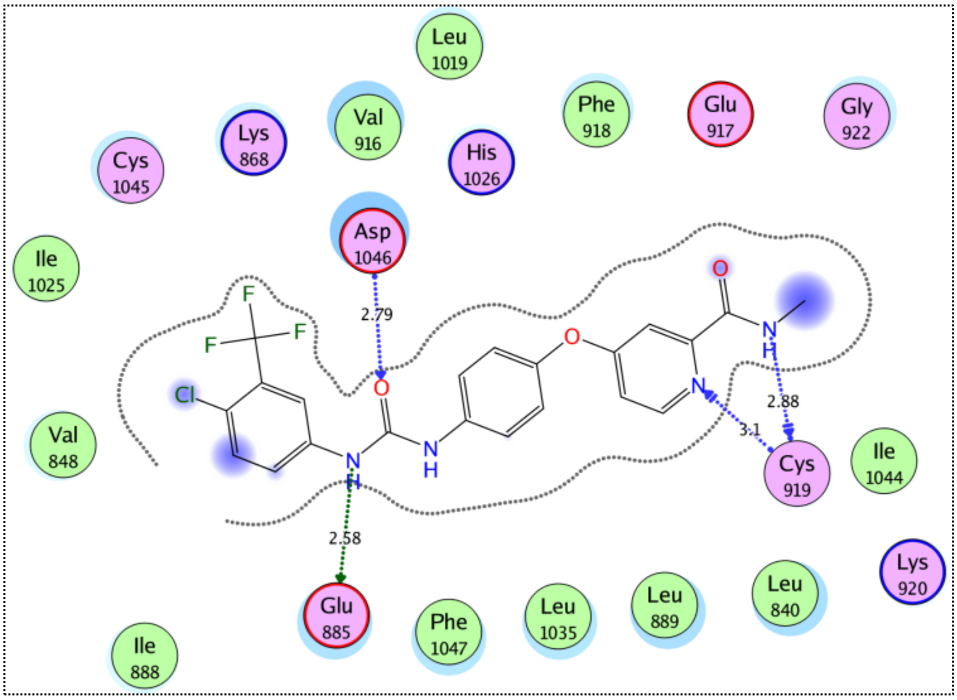

(C)

Fig. 57. 2D diagram (A) and 3D representation (B) of the superimposition of docking pose (green) and the co-crystallized (red) of sorafenib in the VEGFR-2 active site with RMSD of 0.470Å. (C) 2D interaction diagram showing sorafenib docking pose interactions with the key amino acids (hot spots) in the VEGFR-2 active site. (Distances in Å).

4. **2D diagrams of the newly synthesized 1,2-disubstituted benzimidazole showing their interaction with the VEGFR-2 active site; distances in Å; (Fig. 58-85)**

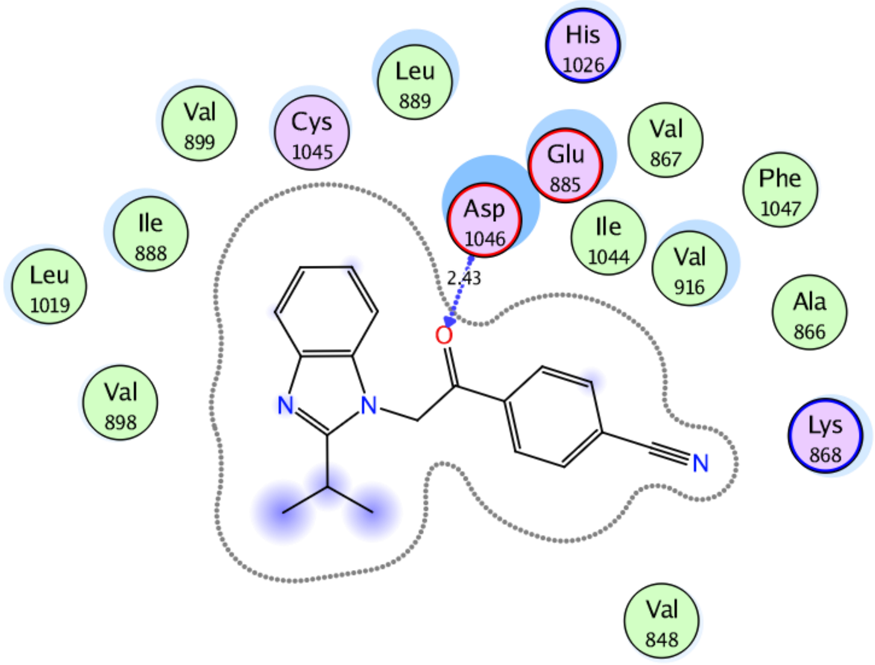

Fig. 58. 2D diagram of **6** showing its interaction with the VEGFR-2 active site. (Distances in Å)

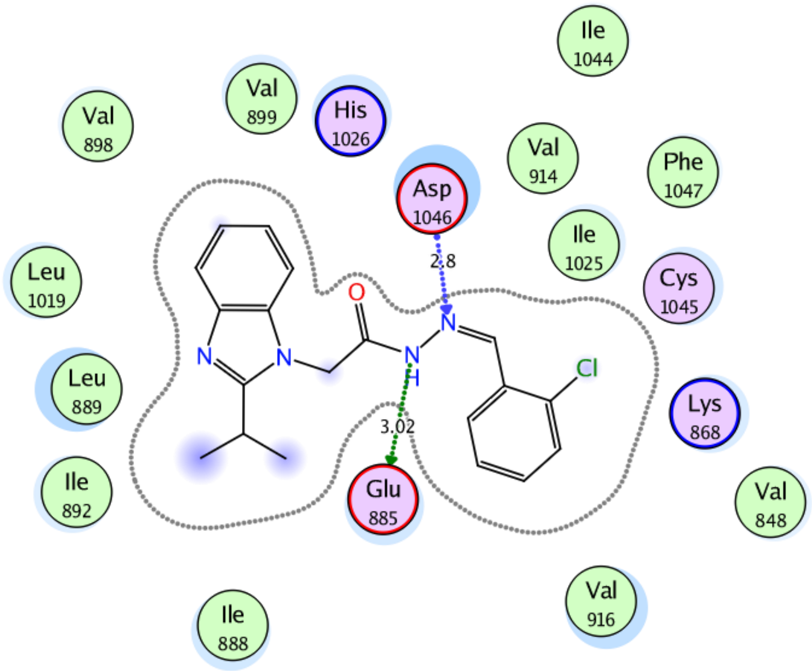

Fig. 59. 2D diagram of **13a** showing its interaction with the VEGFR-2 active site. (Distances in Å)

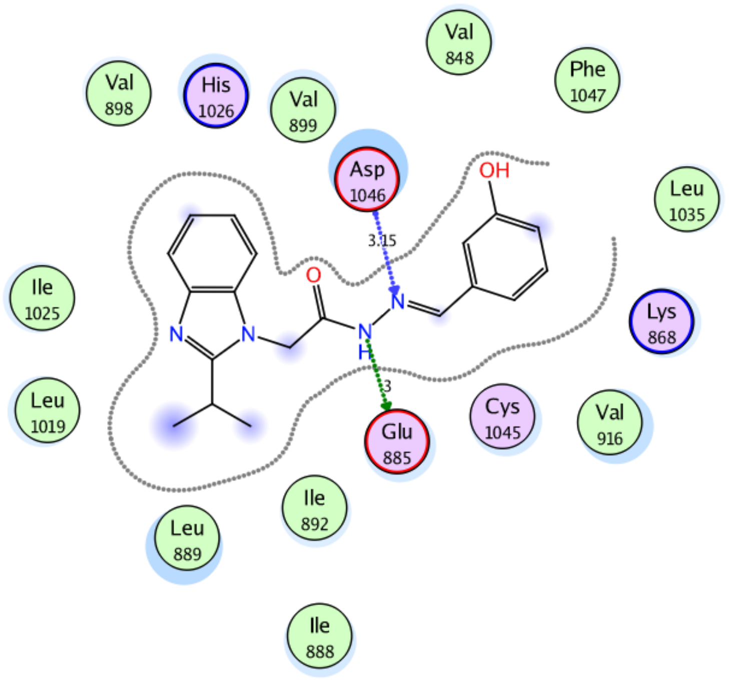

Fig. 60. 2D diagram of **13b** showing its interaction with the VEGFR-2 active site. (Distances in Å)

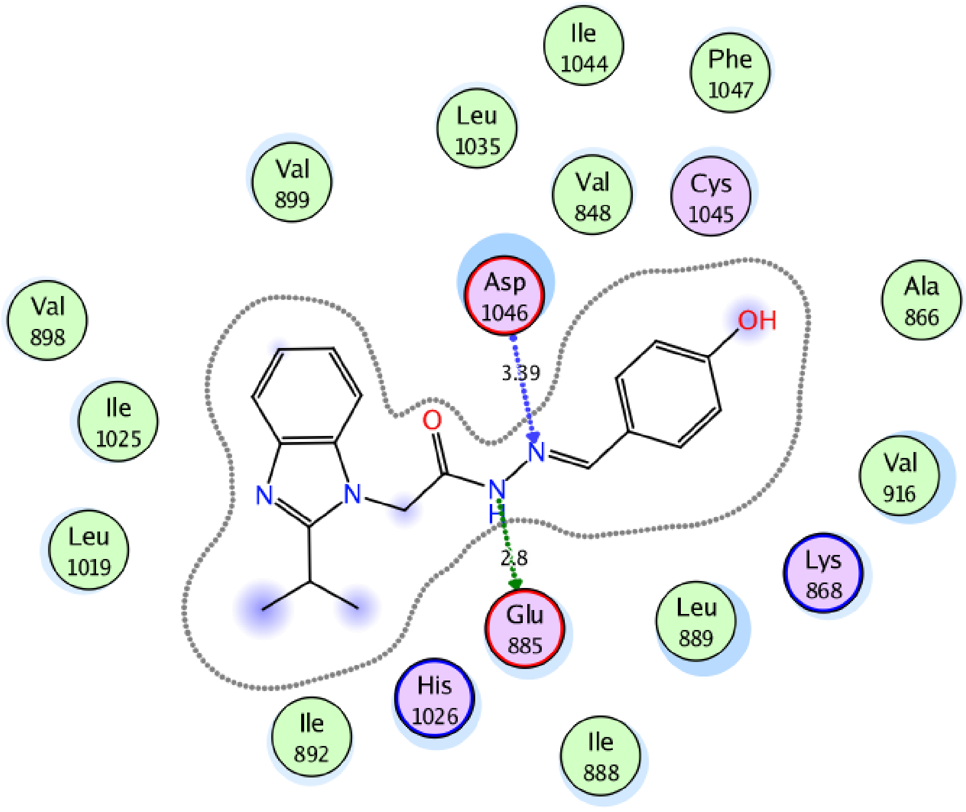

Fig. 61. 2D diagram of **13c** showing its interaction with the VEGFR-2 active site. (Distances in Å)

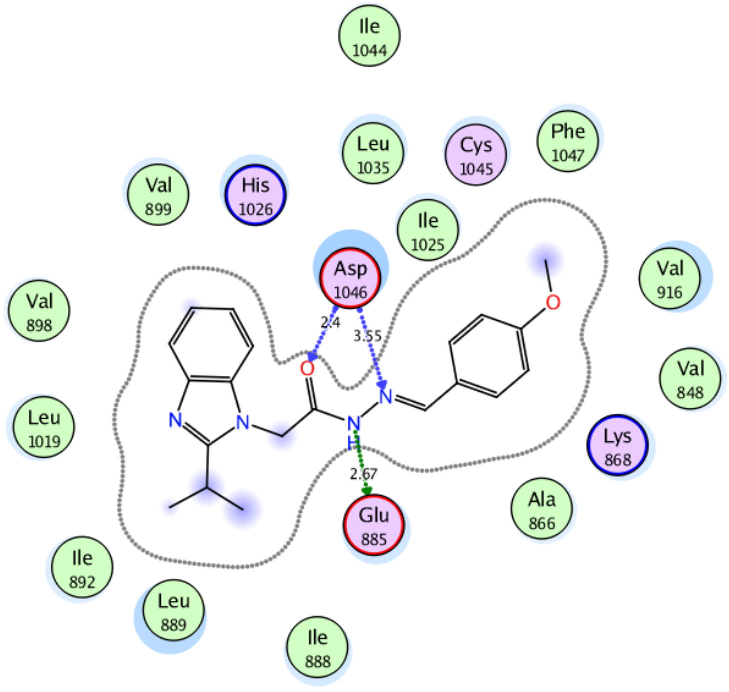

Fig. 62. 2D diagram of **13d** showing its interaction with the VEGFR-2 active site. (Distances in Å)

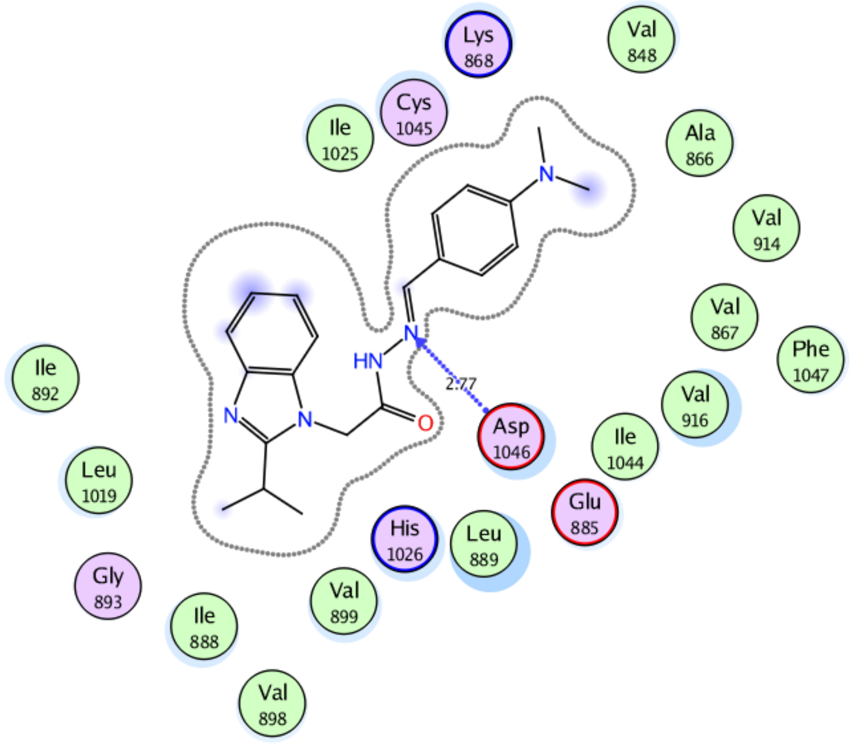

Fig. 63. 2D diagram of **13e** showing its interaction with the VEGFR-2 active site. (Distances in Å)

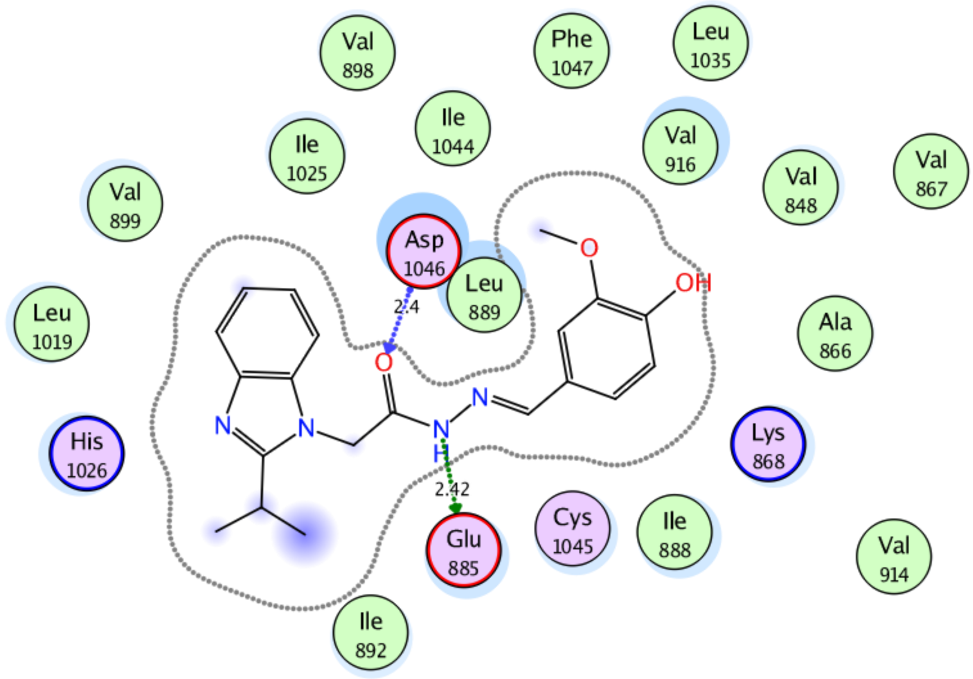

Fig. 64. 2D diagram of **13f** showing its interaction with the VEGFR-2 active site. (Distances in Å)

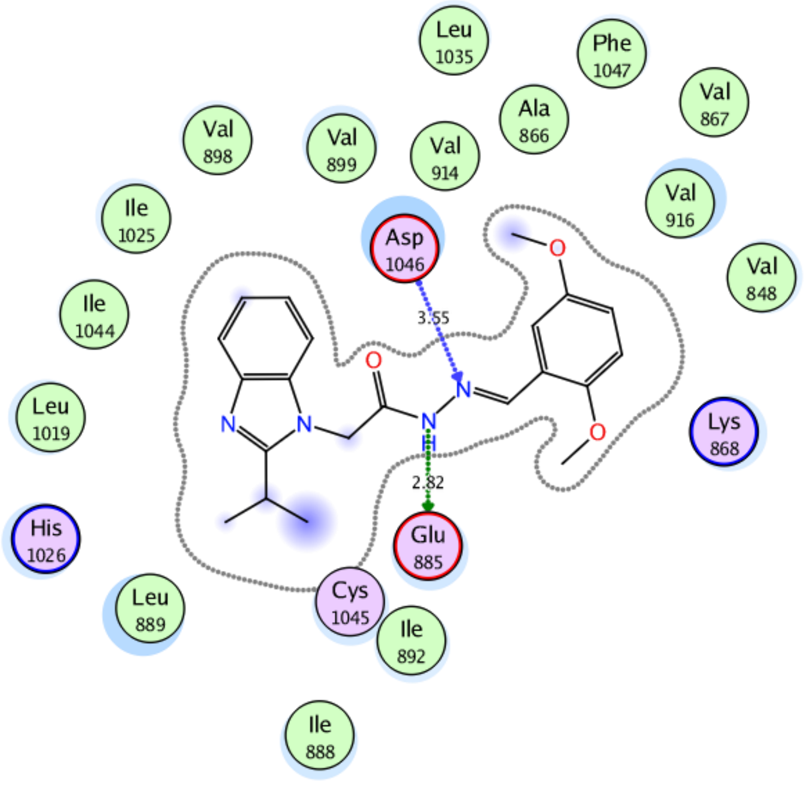

Fig. 65. 2D diagram of **13g** showing its interaction with the VEGFR-2 active site. (Distances in Å)

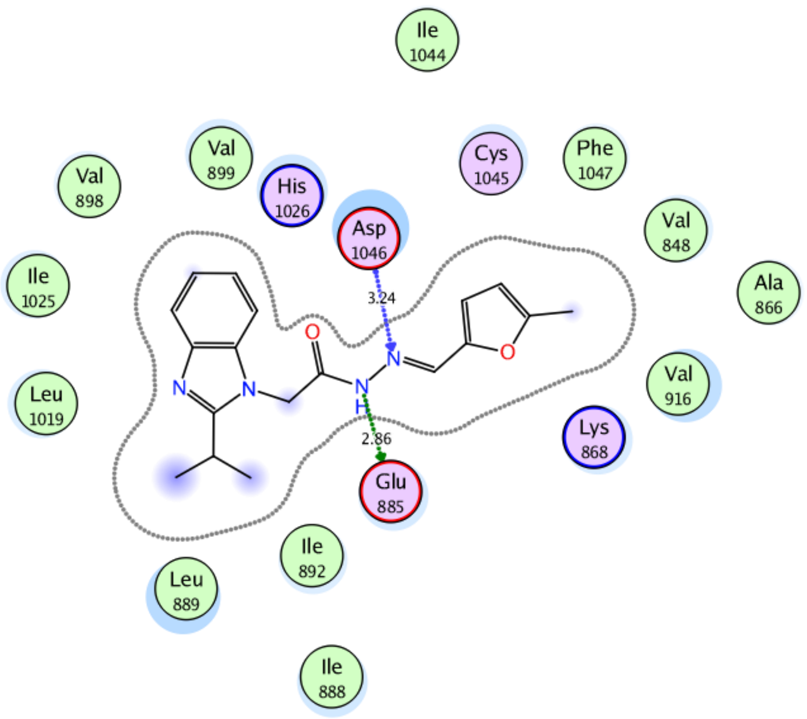

Fig. 66. 2D diagram of **13h** showing its interaction with the VEGFR-2 active site. (Distances in Å)

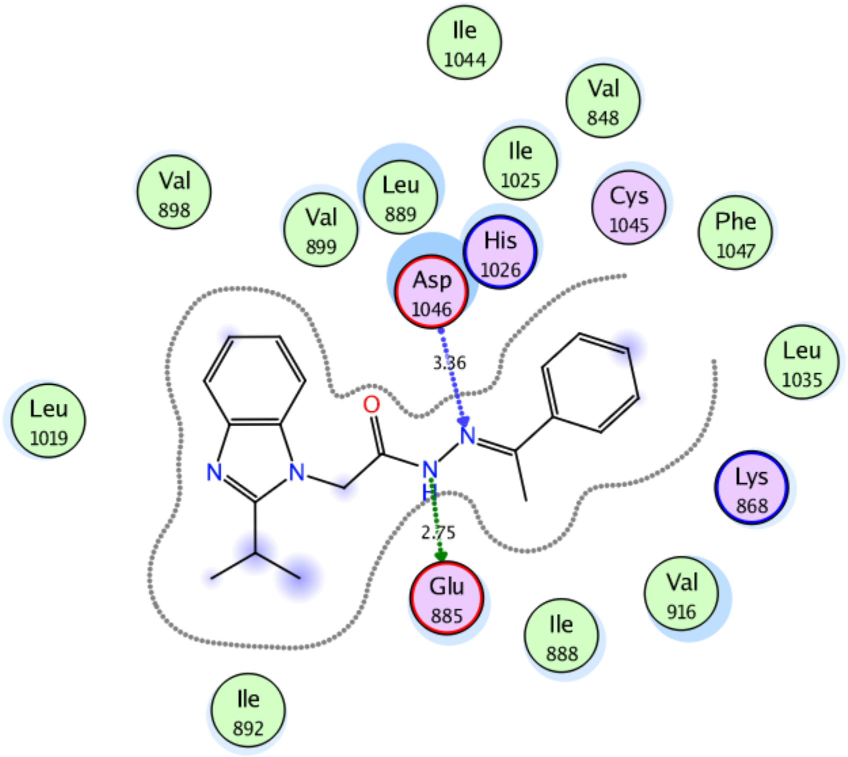

Fig. 67. 2D diagram of **14a** showing its interaction with the VEGFR-2 active site. (Distances in Å)

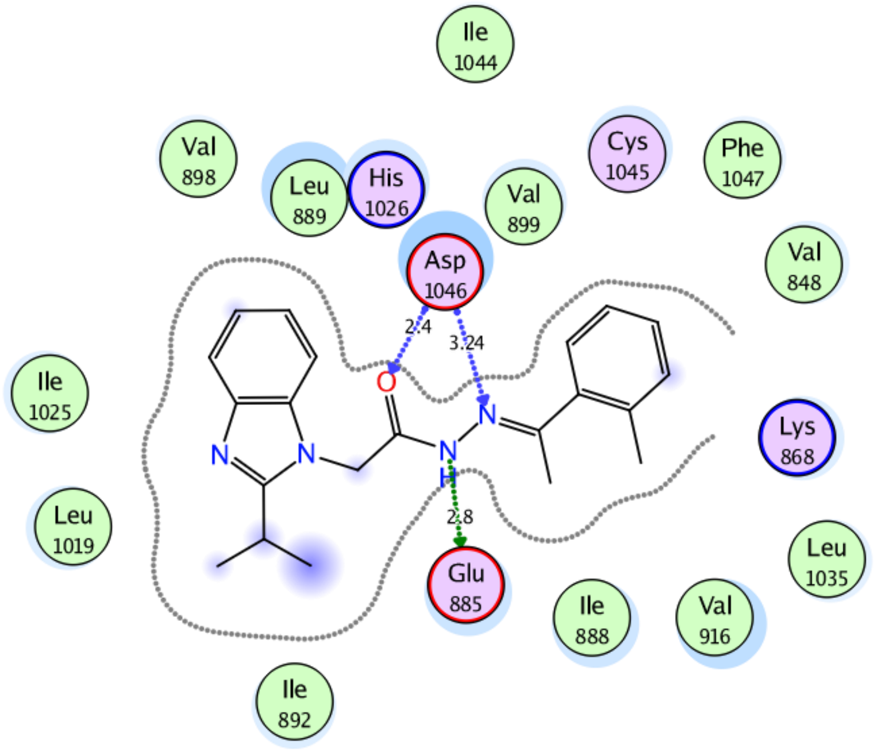

Fig. 68. 2D diagram of **14b** showing its interaction with the VEGFR-2 active site. (Distances in Å)

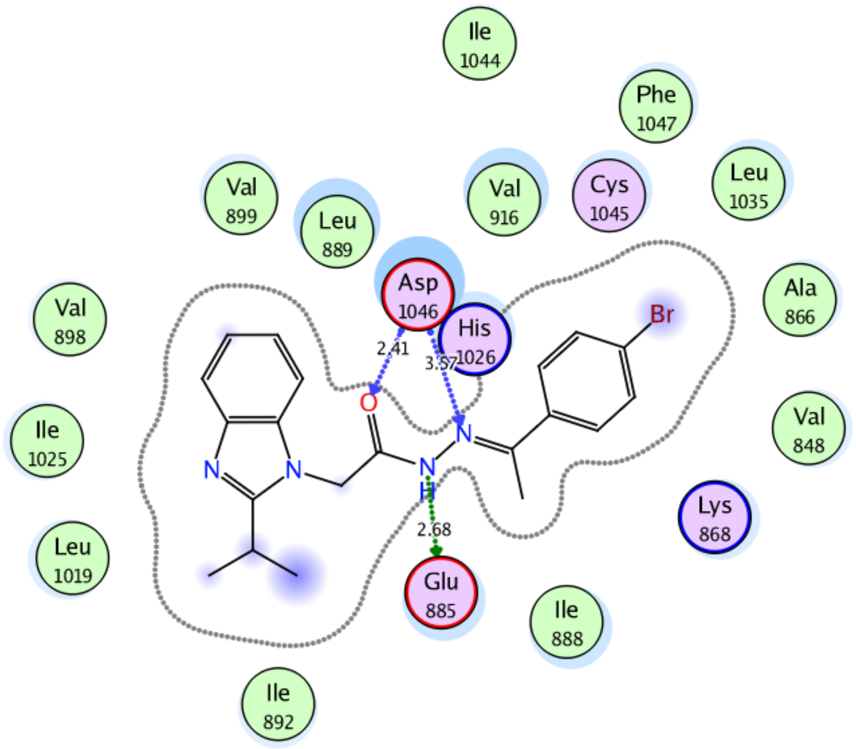

Fig. 69. 2D diagram of **14c** showing its interaction with the VEGFR-2 active site. (Distances in Å)

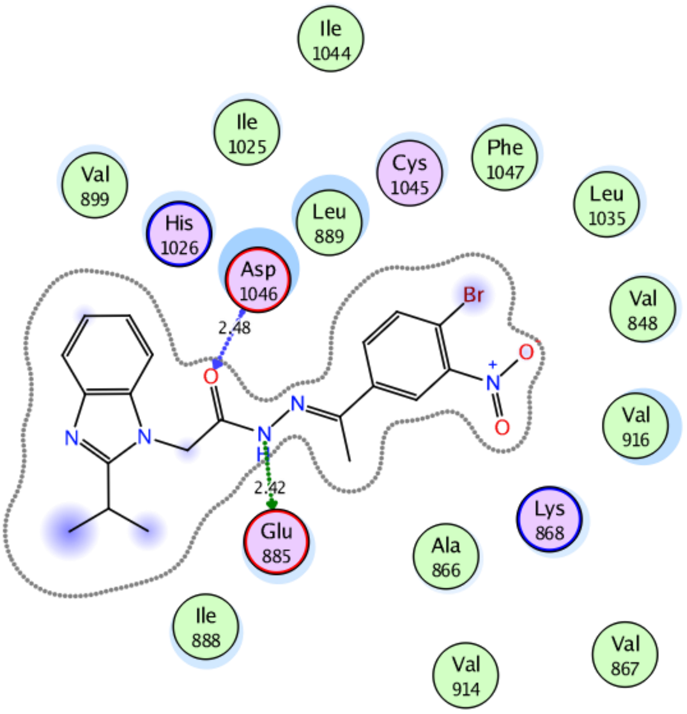

Fig. 70. 2D diagram of **14d** showing its interaction with the VEGFR-2 active site. (Distances in Å)

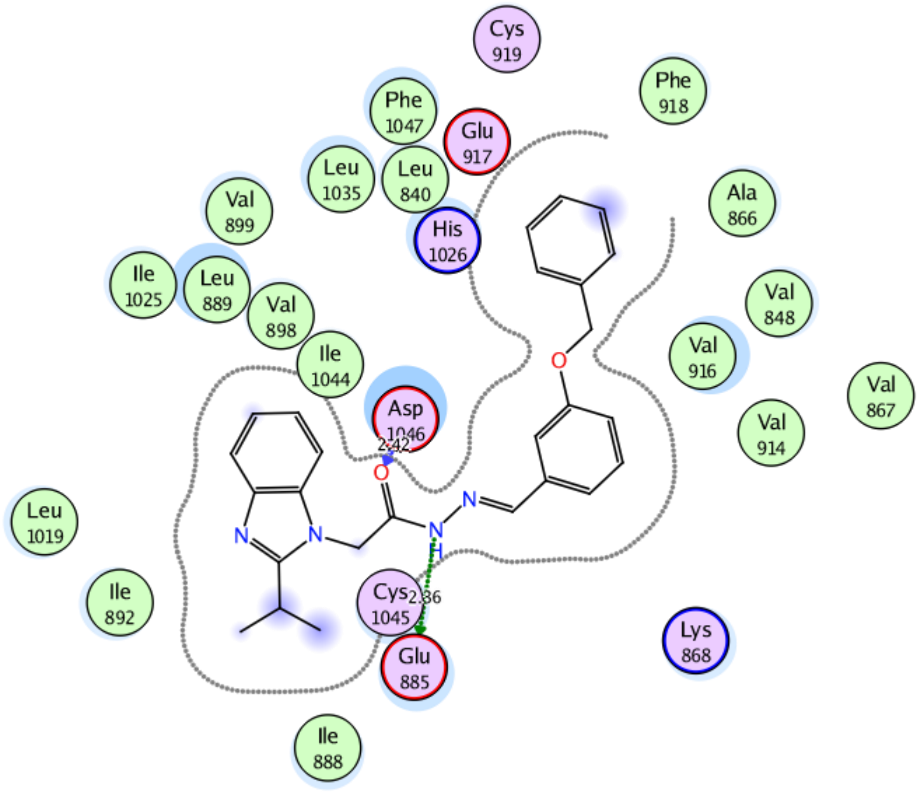

Fig. 71. 2D diagram of **17a** showing its interaction with the VEGFR-2 active site. (Distances in Å)

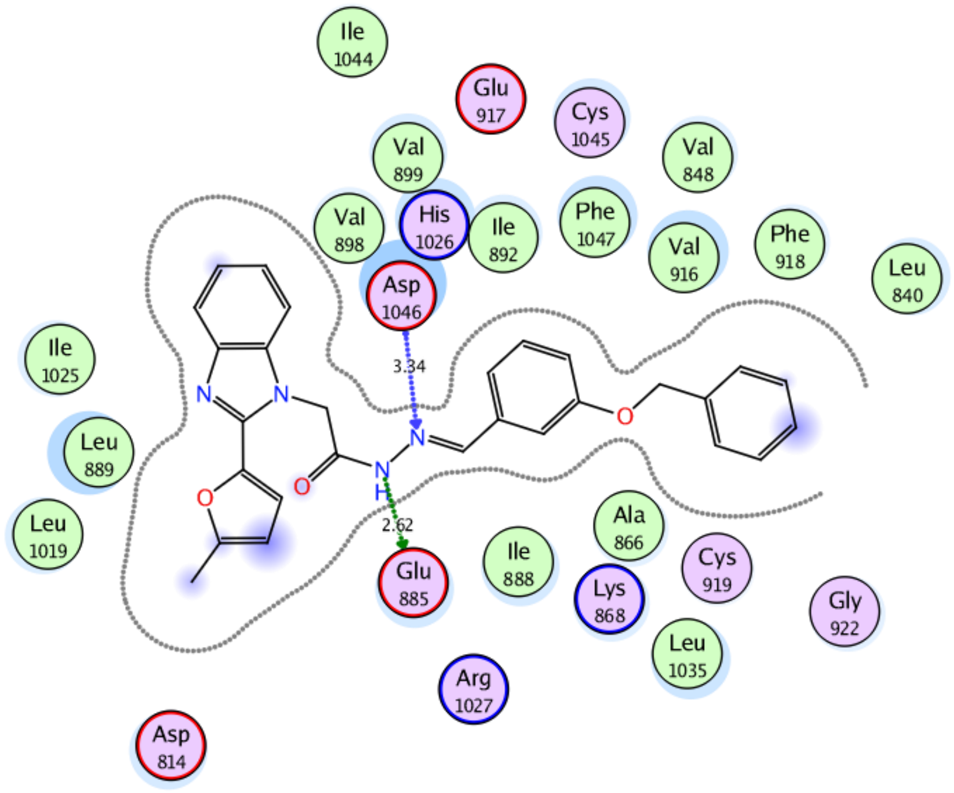

Fig. 72. 2D diagram of **17b** showing its interaction with the VEGFR-2 active site. (Distances in Å)

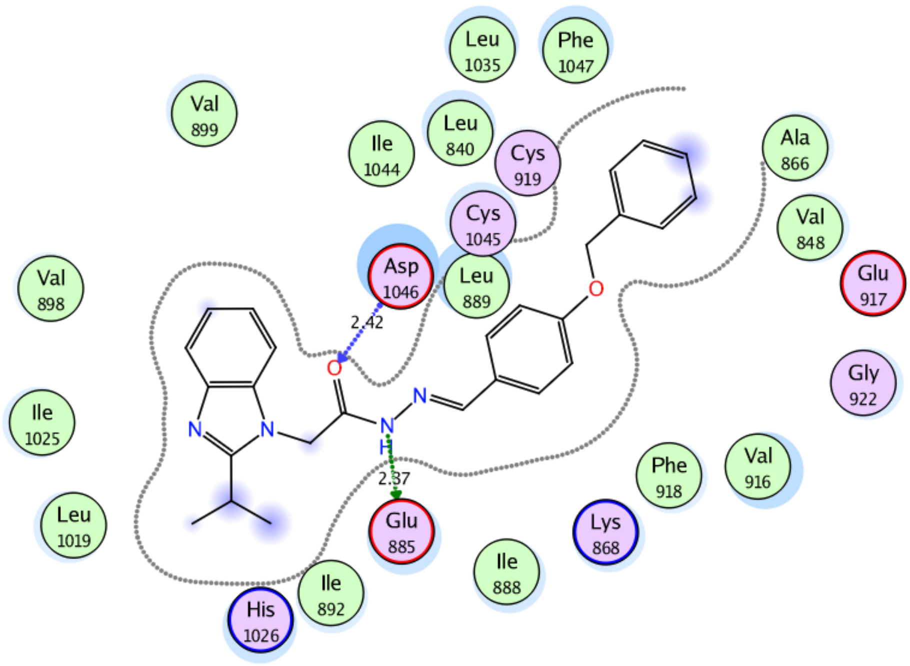

Fig. 73. 2D diagram of **17c** showing its interaction with the VEGFR-2 active site. (Distances in Å)

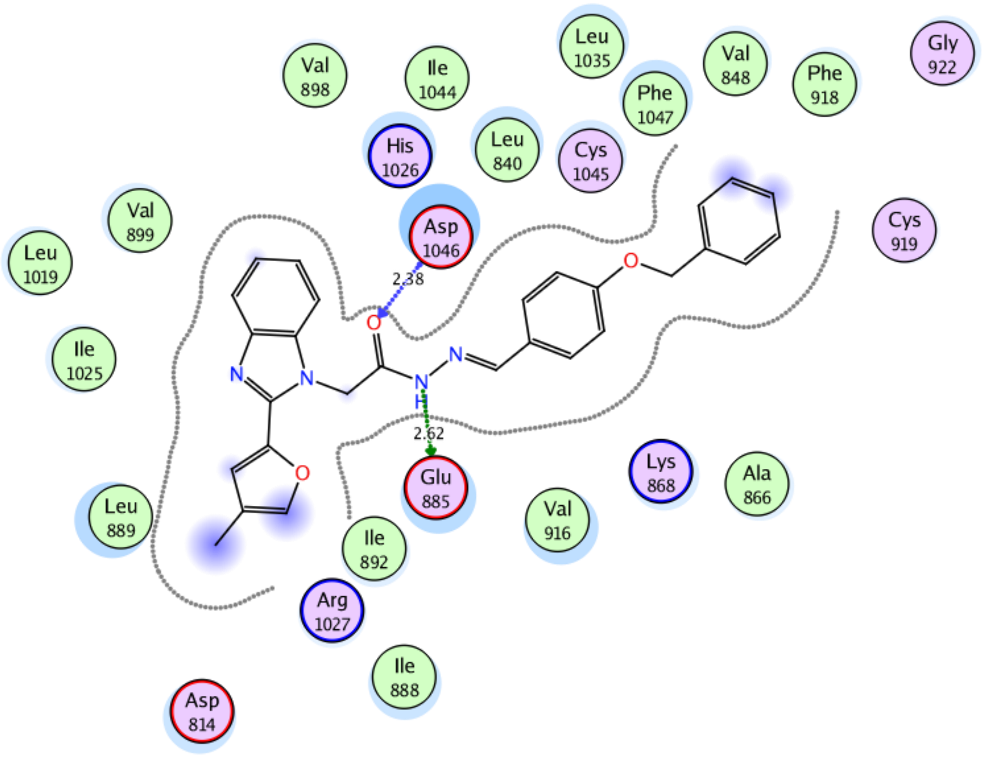

Fig. 74. 2D diagram of **17d** showing its interaction with the VEGFR-2 active site. (Distances in Å)

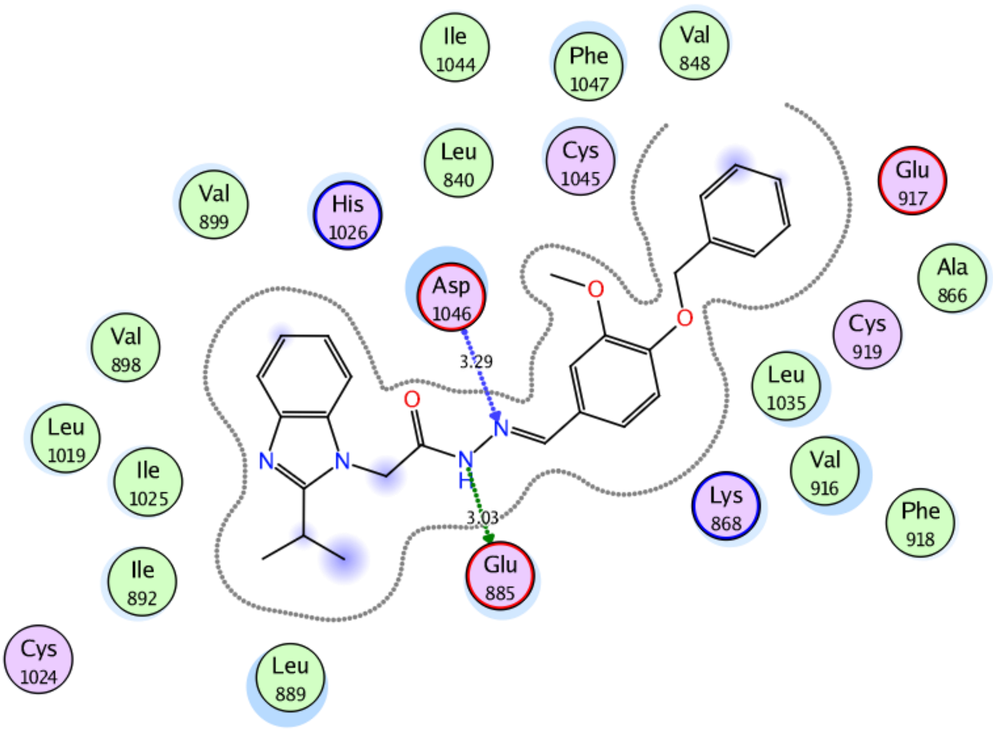

Fig. 75. 2D diagram of **17e** showing its interaction with the VEGFR-2 active site. (Distances in Å)

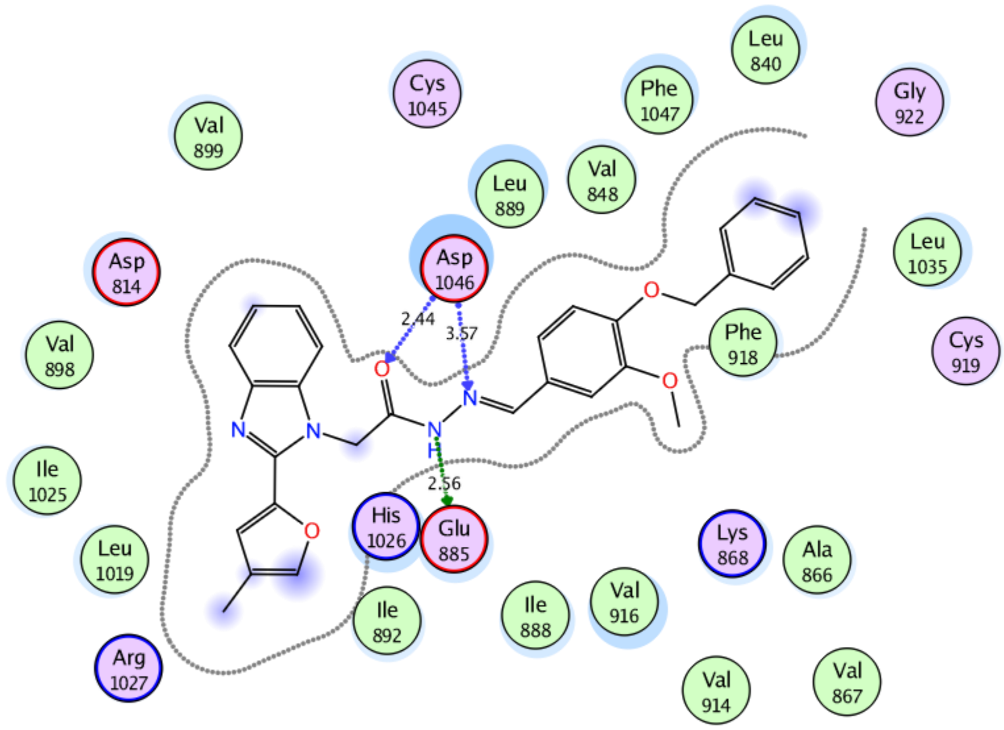

Fig. 76. 2D diagram of **17f** showing its interaction with the VEGFR-2 active site. (Distances in Å)

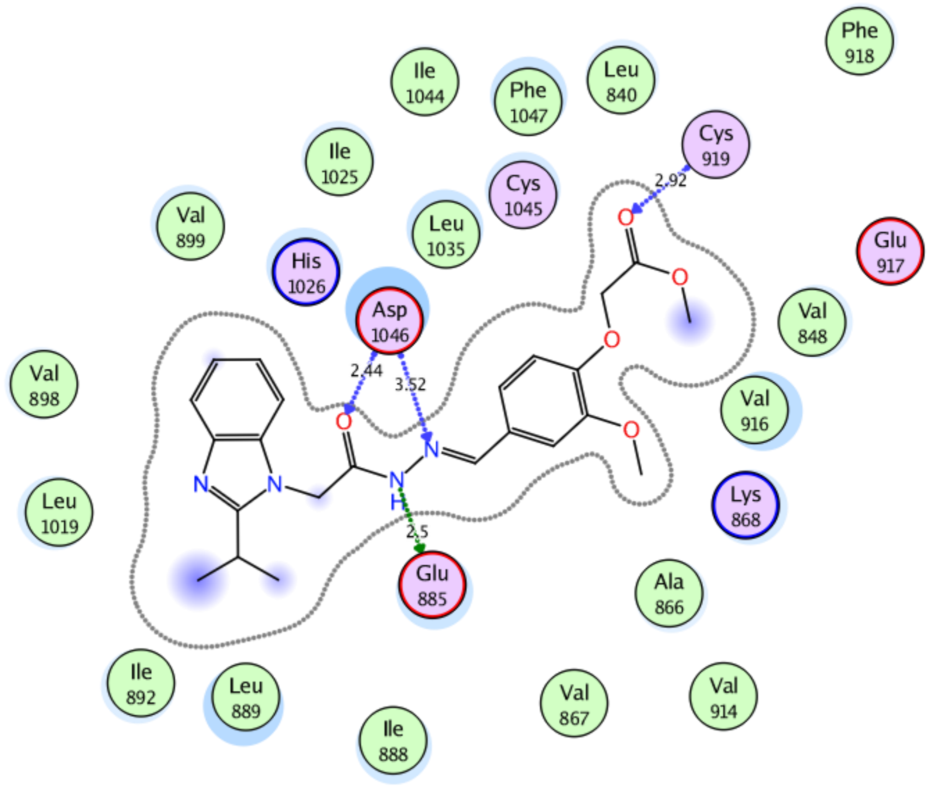

Fig. 77. 2D diagram of **19a** showing its interaction with the VEGFR-2 active site. (Distances in Å)

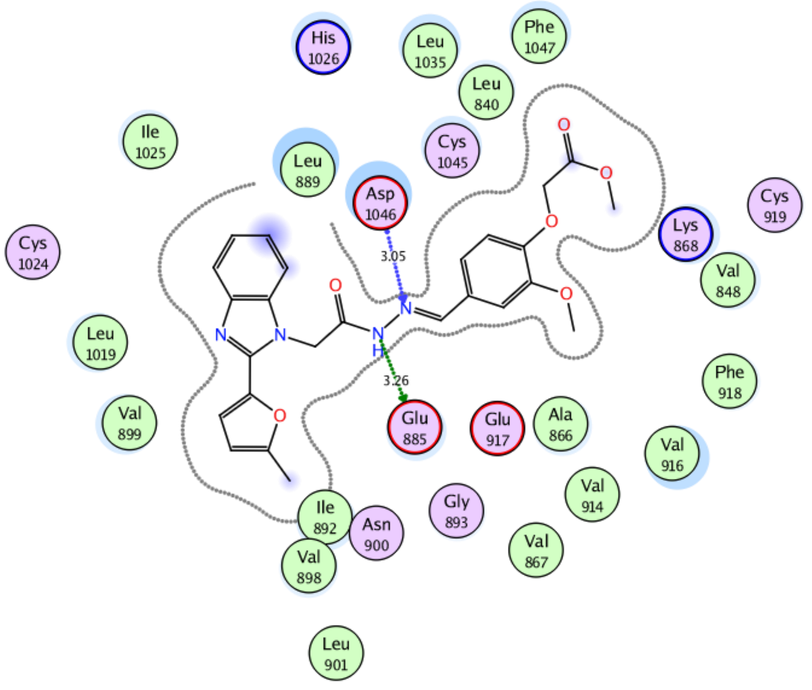

Fig. 78. 2D diagram of **19b** showing its interaction with the VEGFR-2 active site. (Distances in Å)

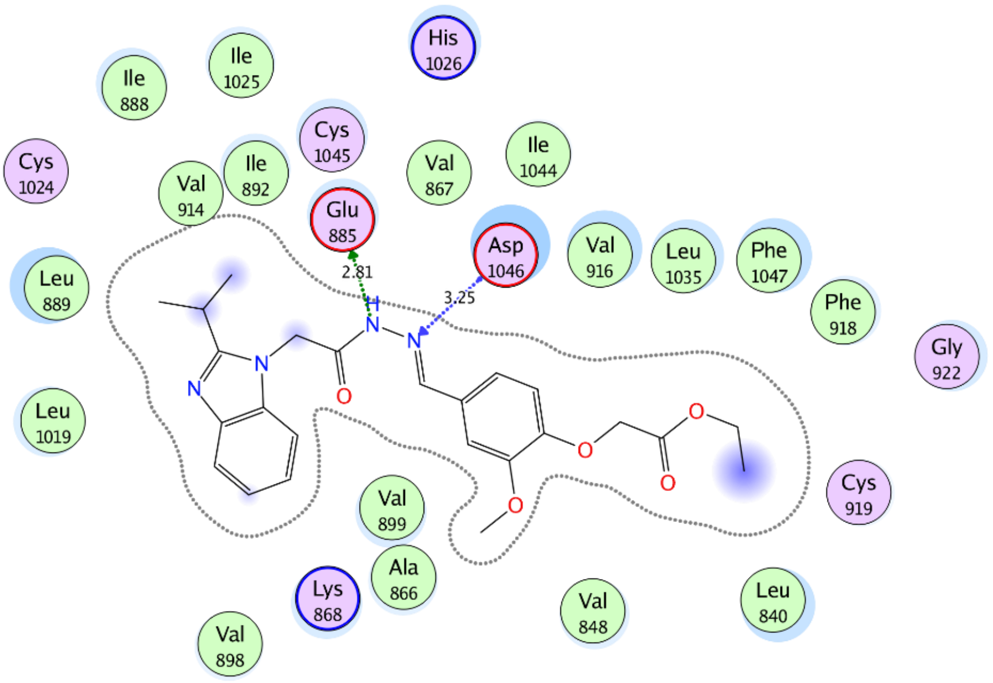

Fig. 79. 2D diagram of **19c** showing its interaction with the VEGFR-2 active site. (Distances in Å)

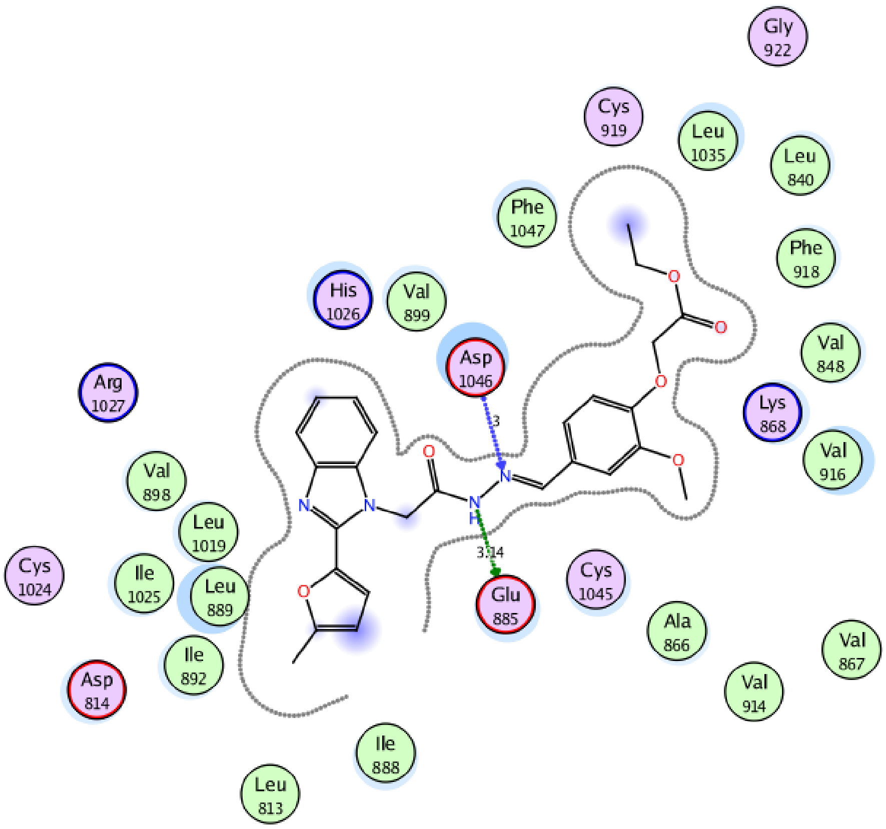

Fig. 80. 2D diagram of **19d** showing its interaction with the VEGFR-2 active site. (Distances in Å)

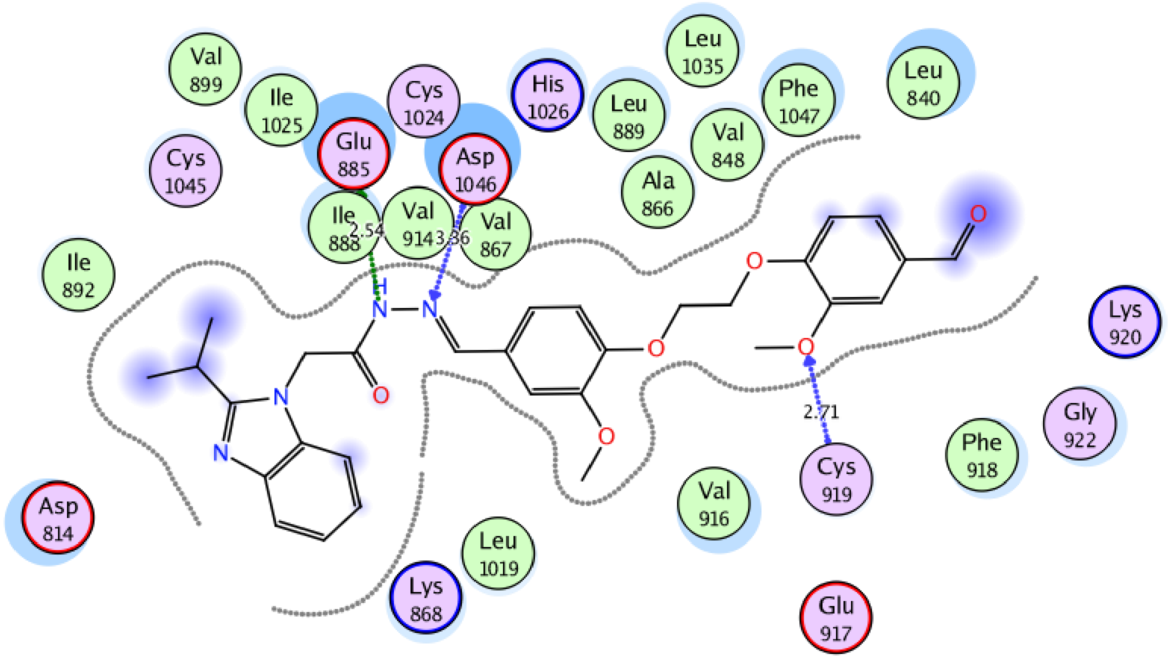

Fig. 81. 2D diagram of **22a** showing its interaction with the VEGFR-2 active site. (Distances in Å)

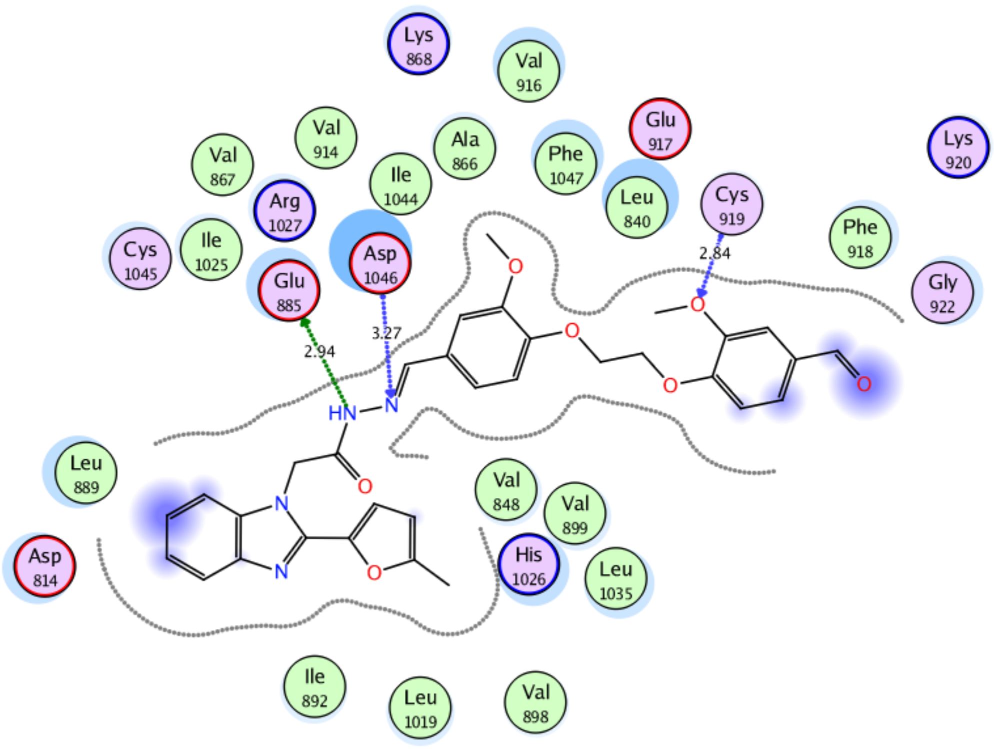

Fig. 82. 2D diagram of **22b** showing its interaction with the VEGFR-2 active site. (Distances in Å)

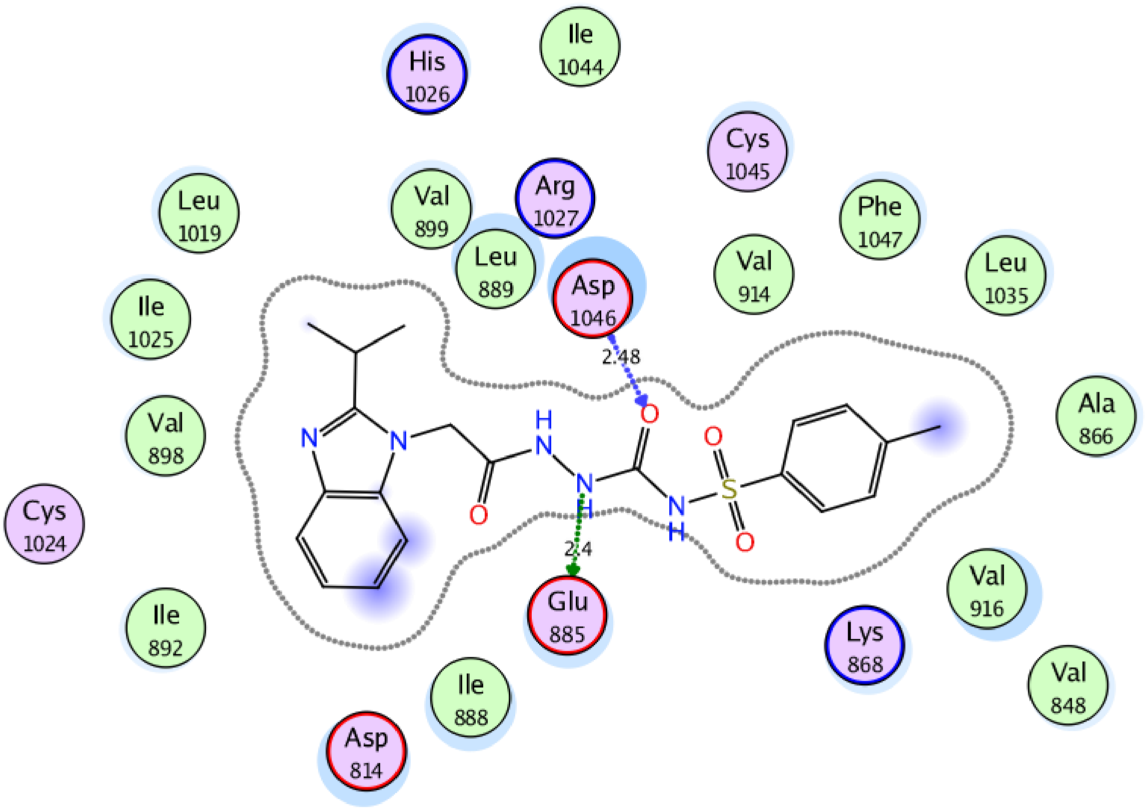

Fig. 83. 2D diagram of **24a** showing its interaction with the VEGFR-2 active site. (Distances in Å)

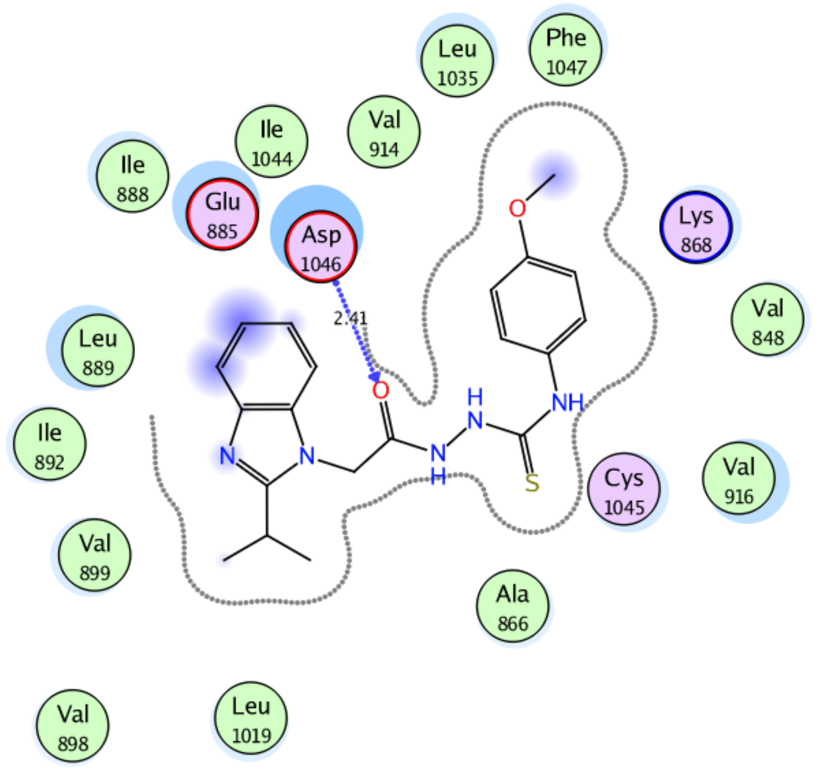

Fig. 84. 2D diagram of **24b** showing its interaction with the VEGFR-2 active site. (Distances in Å)

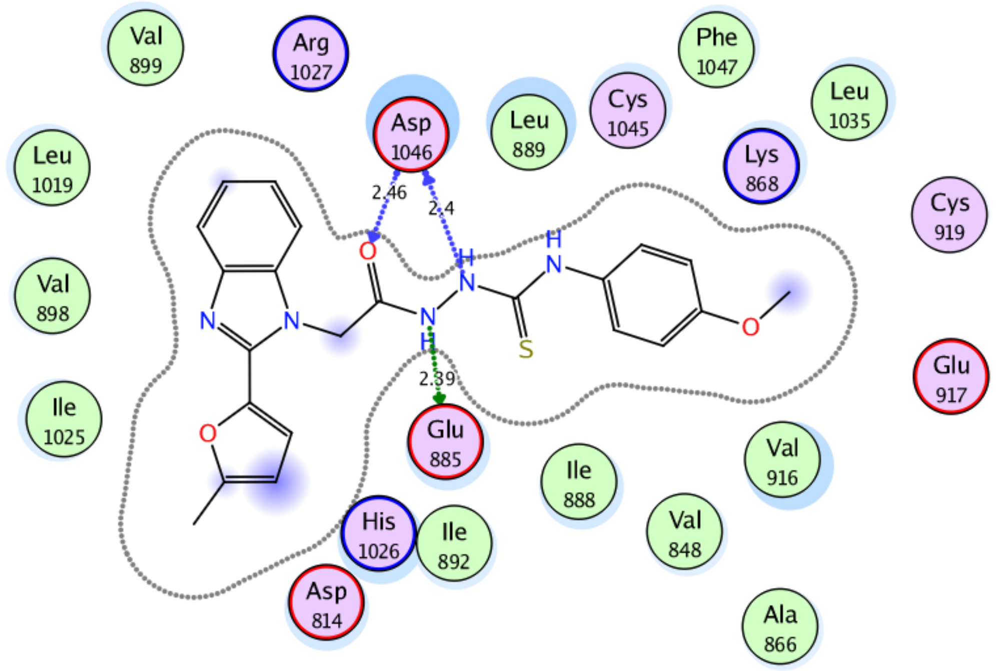

Fig. 85. 2D diagram of **24c** showing its interaction with the VEGFR-2 active site. (Distances in Å
